# Obtaining Pure ^1^H NMR Spectra of Individual Pyranose and Furanose Anomers of Reducing Deoxyfluorinated Sugars

**DOI:** 10.1021/acs.joc.3c01503

**Published:** 2023-09-27

**Authors:** Gabija Poškaitė, David E. Wheatley, Neil Wells, Bruno Linclau, Davy Sinnaeve

**Affiliations:** † School of Chemistry, University of Southampton, Highfield, Southampton SO17 1BJ, United Kingdom; ‡ Department of Organic and Macromolecular Chemistry, Ghent University, Campus Sterre, Krijgslaan 281-S4, Ghent 9000, Belgium; § Univ. Lille, Inserm, CHU Lille, Institut Pasteur de Lille, U1167 RID-AGE - Risk Factors and Molecular Determinants of Aging-Related Diseases, F-59000 Lille, France; ∥ CNRS, EMR9002 Integrative Structural Biology, F-59000 Lille, France

## Abstract

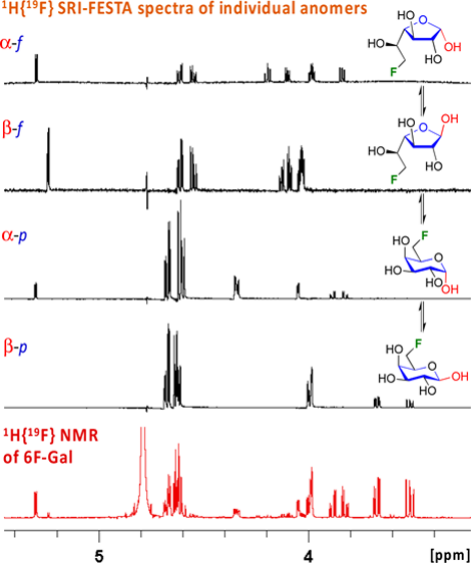

Due to tautomeric equilibria, NMR spectra of reducing sugars can be complex with many overlapping resonances. This hampers coupling constant determination, which is required for conformational analysis and configurational assignment of substituents. Given that mixtures of interconverting species are physically inseparable, easy-to-use techniques that enable facile full ^1^H NMR characterization of sugars are of interest. Here, we show that individual spectra of both pyranoside and furanoside forms of reducing fluorosugars can be obtained using 1D FESTA. We discuss the unique opportunities offered by FESTA over standard sel-TOCSY and show how it allows a more complete characterization. We illustrate the power of FESTA by presenting the first full NMR characterization of many fluorosugars, including of the important fluorosugar 2-deoxy-2-fluoroglucose. We discuss in detail all practical considerations for setting up FESTA experiments for fluorosugars, which can be extended to any mixture of fluorine-containing species interconverting slowly on the NMR frequency–time scale.

## Introduction

Fluorinated carbohydrates are a much employed class of compounds for a wide range of applications, including spectroscopic probes for protein–carbohydrate interactions,^1^ mechanism-based inhibitors,^2,3^ and as a means to modify or investigate the properties of biologically active sugar-based compounds^4−7^ or carbohydrate-based materials.^8^ Fluorination of sugars is also of interest to modify physical properties such as lipophilicity, with application in the design of carbohydrate mimetics.^9−13^ Many ^19^F NMR-based experiments have been designed to investigate the binding of fluorinated sugars,^14−17^ including for individual reducing sugar anomers.^18^ A very important application in medicine involves their use as imaging agents,^19−21^ and many nucleoside-based drugs contain a fluorinated sugar subunit.^22−26^ Most of the monodeoxyfluorinated analogues of the important sugars have been described, as are the syntheses of a great many polyfluorinated sugars.^27^ The characterization of fluorinated sugars is facilitated by the presence of the fluorine atom(s) due to a characteristic downfield chemical shift of adjacent hydrogen atoms and by H–F coupling constants—often with many long-range couplings, although these additional couplings cause increased signal overlap. While ^19^F decoupling can be employed to reduce signal overlap, heavily overlapping multiplets due to homonuclear couplings can still preclude complete spectral characterization, just as for nonfluorinated sugars.

**Scheme 1:**
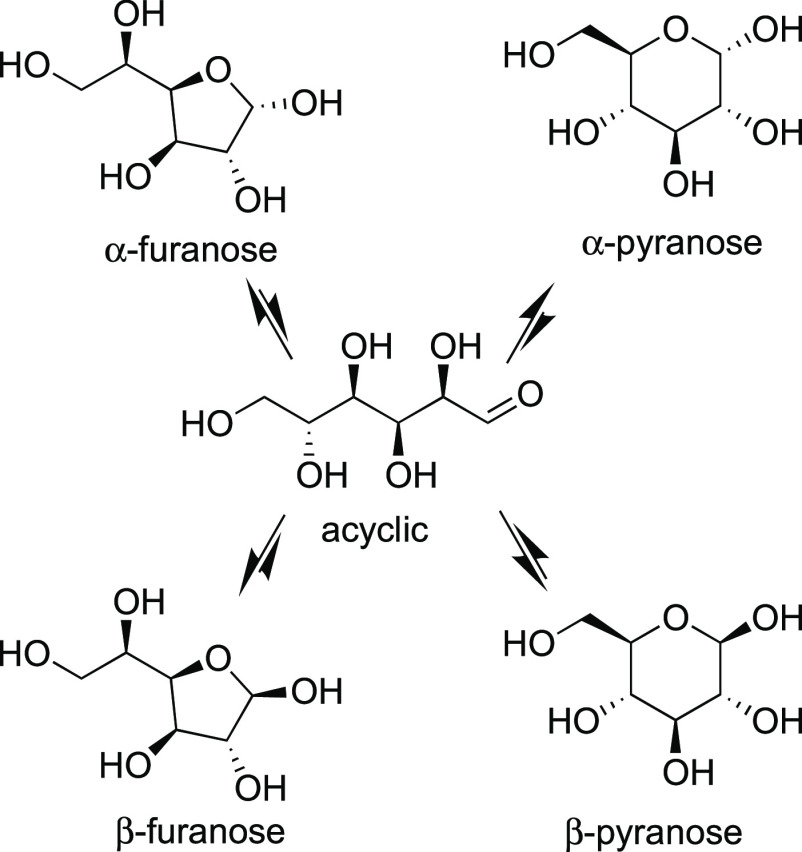
Mutarotation and Tautomerization Equilibria of d-Glucose

Reducing sugars typically consist of a mixture of anomers with an interconversion rate that is slow on the NMR frequency–time scale. Hence, for hexose sugars, both the α- and the β-pyranose anomers are visible in the NMR spectra as a mixture of compounds. For certain sugars, the anomers of the corresponding furanose sugars are sufficiently populated to be detected by NMR, though these are typically present in small amounts (Scheme 1). This can considerably complicate NMR spectra as resonances of protons on a given position in the sugar ring often have close chemical shift values, leading to overlapping signals, which can severely hamper spectral characterization. For example, even for common fluorinated sugars such as 2-deoxy-2-fluoroglucopyranose and 2-deoxy-2-fluoromannopyranose, complete characterization (in which all *J*_HH_ and *J*_HF_ values for all detectable anomers have been determined) is still not reported. The situation is exacerbated for furanoses given their low abundance. The complete spectral assignment of the furanose anomers of glucose, which was achieved using a combination of selective and nonselective 1D and 2D NMR experiments, complemented by spin simulations and iterative spectral analysis, was only published in 2022.^28^

**Figure 1:**
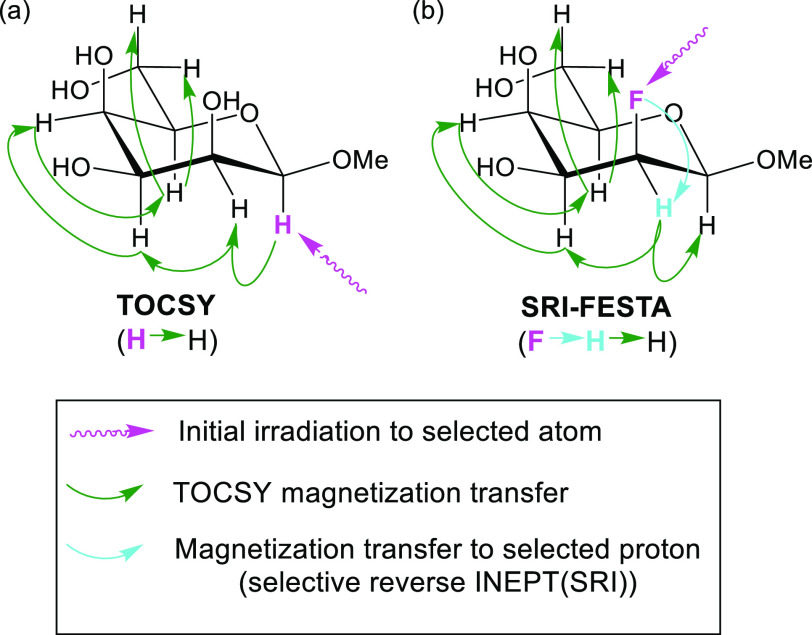
Principle of selective TOCSY (starting from the anomer H1 proton) and SRI-FESTA (starting from the fluorine and selectively transferred to any selected proton coupling partner) NMR experiments.

NMR techniques that are able to separate spectra of individual compounds from mixtures without the need for their physical separation have attracted wide interest, as they pay dividends in terms of time and effort needed for analysis. Such “virtual” spectroscopic separation techniques can occur by correlating the signals with molecular diffusion, as done in diffusion-ordered spectroscopy (DOSY).^29,30^ However, these experiments are severely limited in the case of signal overlap with other anomers or compounds. DOSY requires sufficiently large differences in diffusion coefficients, especially when there is signal overlap, which is not straightforward for anomers. Nevertheless, there exists some precedence for separation of the pyranose anomer spectra of glucose and mannose.^31,32^ The resolving power can be improved upon by including relaxation encoding and parallel factor analysis^33,34^ or by avoiding overlap by including pure shift resolution,^35,36^ but limitations remain in cases of compounds with similar chemical shifts and also with large concentration differences. Alternatively, well-resolved and recognizable individual proton signals of the various compounds can be selectively excited, followed by a TOCSY (total correlation spectroscopy) mixing step to spread its polarization to the rest of the spin system (Figure 1a);^37^ if needed, it can be augmented with relaxation or diffusion encoding and/or pure shift resolution.^38−40^

Selective TOCSY (sel-TOCSY) is often employed to characterize oligo- and polysaccharides.^41−43^ In TOCSY, a ^1^H isotropic mixing scheme^37^ (such as a DIPSI2 spinlock)^44^ of duration τ_m_ is applied, during which magnetization from one proton spreads out to other protons in the same spin system, mediated by the ^1^H–^1^H scalar coupling network that exists between these protons. The resulting peak intensities relative to the initial ^1^H magnetization depend on the magnitudes of these *J*_HH_ couplings, the number of protons in the coupling network, the number of relayed steps from the initial proton(s), and the chosen τ_m_. Next to the well-known 2D TOCSY experiment, also 1D sel-TOCSY spectra can be produced by selectively generating the initial ^1^H magnetization using frequency-selective pulses. Starting from a well-resolved signal, TOCSY propagation can thus be used to retrieve all signals from a particular spin system without interference of other spin systems. In sugars, the anomeric proton resonances are often well separated from the bulk of the saccharide signal. As the anomer signals of a reducing sugar often have distinct chemical shifts, TOCSY thus, in principle, allows one to obtain spectra of the individual anomers. In practice, a number of issues are encountered with this strategy. First, in some cases, anomeric protons do overlap, be it with signals from other anomeric forms or with those of impurities. Especially for minor furanose forms, this can be problematic. Chemical-shift-selective filtering^45,46^ or the recently introduced GEMSTONE technique^47,48^ can overcome the issue of selective excitation of overlapping multiplets, but these experiments require precise knowledge of chemical shifts and very long selective elements (resulting in excessive T_2_ relaxation losses) when chemical shift differences are very small. Second, since the architecture of the monosaccharide ^1^H spin system is unbranched, the transfer of magnetization during the TOCSY spinlock starting from the anomeric H1 to the exocyclic H6 only follows a single ^1^H → ^1^H pathway with many relay transfer steps. This means the transfer efficiency relies on the successive ^3^*J*_HH_ scalar coupling constants being sufficiently large, and longer spinlock times may be needed to obtain the full spin system in case small couplings are present.^43^ Each hexapyranose stereochemistry leads to a unique series of scalar coupling constants and hence different TOCSY propagation patterns. As reported by Martins et al.,^41^ when starting from the anomeric proton, TOCSY mixing does not propagate across the whole spin network for galactose and mannose hexapyranoses at spinlock times of 100 ms. Starting from protons halfway, the spin system would reduce the number of relayed steps and thus spinlock durations required, but these are typically part of overlapped regions and thus cannot be straightforwardly used in sel-TOCSY experiments.

Given the importance of fluorinated compounds in medicinal chemistry and chemical biology,^7,49−52^ there has been great interest in the development of NMR techniques that make good use of fluorine nuclei.^53−56^ Yet, there has only been limited precedence of exploiting ^19^F NMR to separate the anomer ^1^H spectra of fluorinated carbohydrates. For 2-fluoro-2-deoxy-d-glucose (FDGlc-2) metabolites, O’Connell and London proposed a 2D TOCSY NMR experiment with direct ^19^F detection,^57^ where ^1^H TOCSY traces can be obtained from the indirect dimension. This approach requires impractically long 2D acquisition schemes of at least several hours to obtain the digital resolution needed for multiplet analysis. Although not demonstrated on fluorosugars, Krishnamurthy and co-workers published a similar 3D ^19^F–^1^H heteronuclear TOCSY filtered experiment for mixture analysis, but this experiment requires very long experimental times (reported 60 h).^58^ During the course of our work, Uhrín and Bell published a set of 2D ^1^H–^19^F experiments for mixture analysis, and while this was not emphasized, it also featured a demonstration on FDGlc-3.^59^

Recently, the selective 1D FESTA (fluorine-edited selective TOCSY acquisition) experiment has been proposed for the analysis of mixtures of fluorinated compounds.^60−62^ As illustrated in Figure 1b, the experiment starts with the selective excitation of a chosen fluorine resonance. Next, a selective reverse INEPT (SRI) sequence^60^ transfers this coherence to a chosen proton coupling partner (^19^F → ^1^H), giving antiphase ^1^H magnetization. The subsequent ^1^H-selective modulated echo (SME)^63^ then lets this evolve into in-phase ^1^H magnetization followed by the TOCSY in-phase transfer (^1^H → ^1^H). An alternative for SRI is the use of a modulated echo (MODO) sequence, which uses difference spectroscopy.^62^ While MODO-FESTA provides the best signal-to-noise ratio, SRI-FESTA yields a higher signal-to-artifact ratio and thus the cleanest results. The latter is thus the preferred choice when looking for lower intensity components in mixtures.^62^ FESTA is particularly advantageous when ^1^H-selective pulses alone cannot select signals exclusively from a unique compound due to spectral overlap. Since the SRI element that generates the initial proton magnetization is doubly selective for ^1^H and ^19^F frequencies, it is limited only by overlap occurring with another anomer’s multiplets in the ^1^H and ^19^F spectrum *at the same time* and even then only when these are coupled to each other (see below). Given the very wide chemical shift dispersion of fluorine and the sparsity of signals in ^19^F NMR spectra, it is very likely that there will be at least one ^19^F/^1^H pair that does not suffer from this limitation. This offers an exceptionally high probability of discrimination between different fluorinated components in the mixture and allows obtaining clean ^1^H NMR subspectra for individual spin systems. FESTA compares to the 2D ^19^F–^1^H CP-DIPSI3-DIPSI2 experiment, which is part of the aforementioned set of experiments by Uhrín and Bell,^59^ in similar ways as does 1D sel-TOCSY to 2D TOCSY and can thus be seen as complementary. Just like sel-TOCSY, FESTA thus holds the advantage of short experimental times (from seconds to minutes depending on the amount of signal averaging needed) and, thanks to the selective ^19^F excitation and polarization transfer with gradient pulses, should show fewer spectral complications from intense signals coming from other anomers compared to nonselective 2D methods (cf. t_1_ noise). The latter is especially important for minor compounds in mixtures.

**Figure 2:**
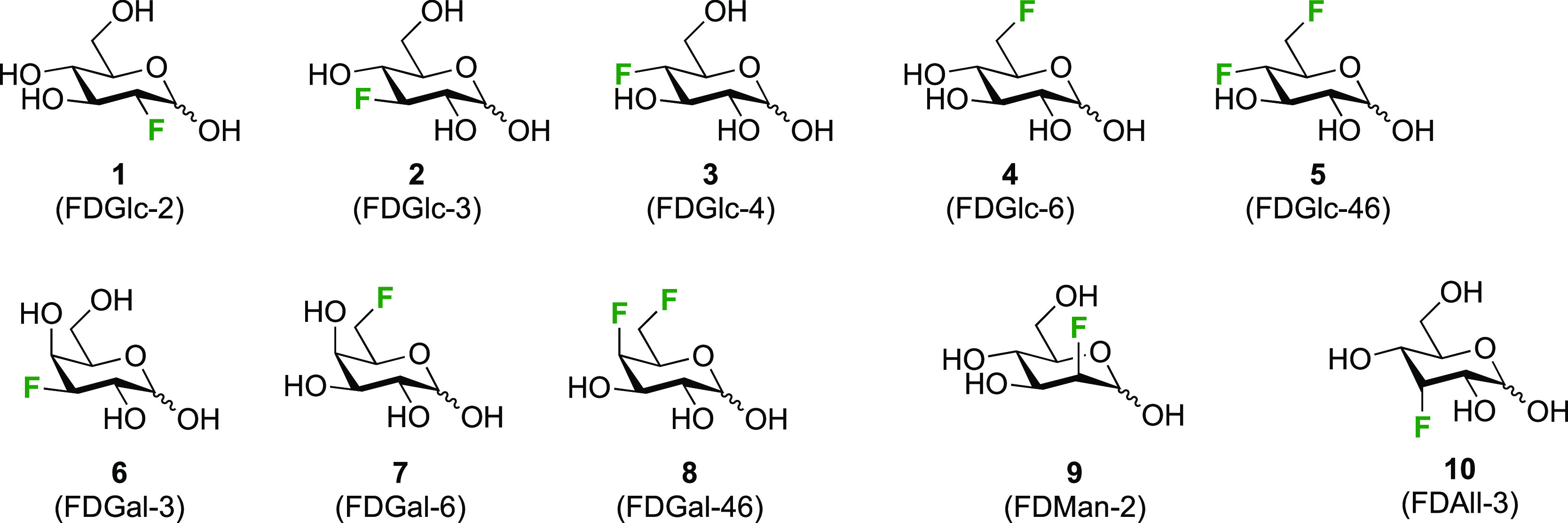
Fluorosugars studied with SRI-FESTA (only the pyranose anomers are shown).

We thus envisioned the SRI-FESTA technique as a very suitable methodology for obtaining individual anomer spectra (which we will term here “anomer subspectra”) of reducing fluorinated sugar derivatives, overcoming the above-described limitations of 1D sel-TOCSY experiments. We set out to perform a detailed study on the scope and limitations of FESTA in the context of fluorosugars and make the comparison with standard 1D sel-TOCSY experiments. In this contribution, we first discuss in detail the experimental considerations for setting up FESTA for fluorosugars (or other interconverting mixtures), serving as a practical guide. Using the set of examples shown in Figure 2, we show how pure anomer subspectra can be obtained, even of the low-abundance furanose tautomers. Second, we demonstrate how the FESTA spectra can facilitate both ^1^H NMR assignment and multiplet analysis and provide the first complete spectral characterization of several anomers shown in Figure 2. Finally, we pay special attention to the characterization of the minor furanose forms.

## Results and Discussion

### Decomposition into Anomer Subspectra by FESTA

**Figure 3:**
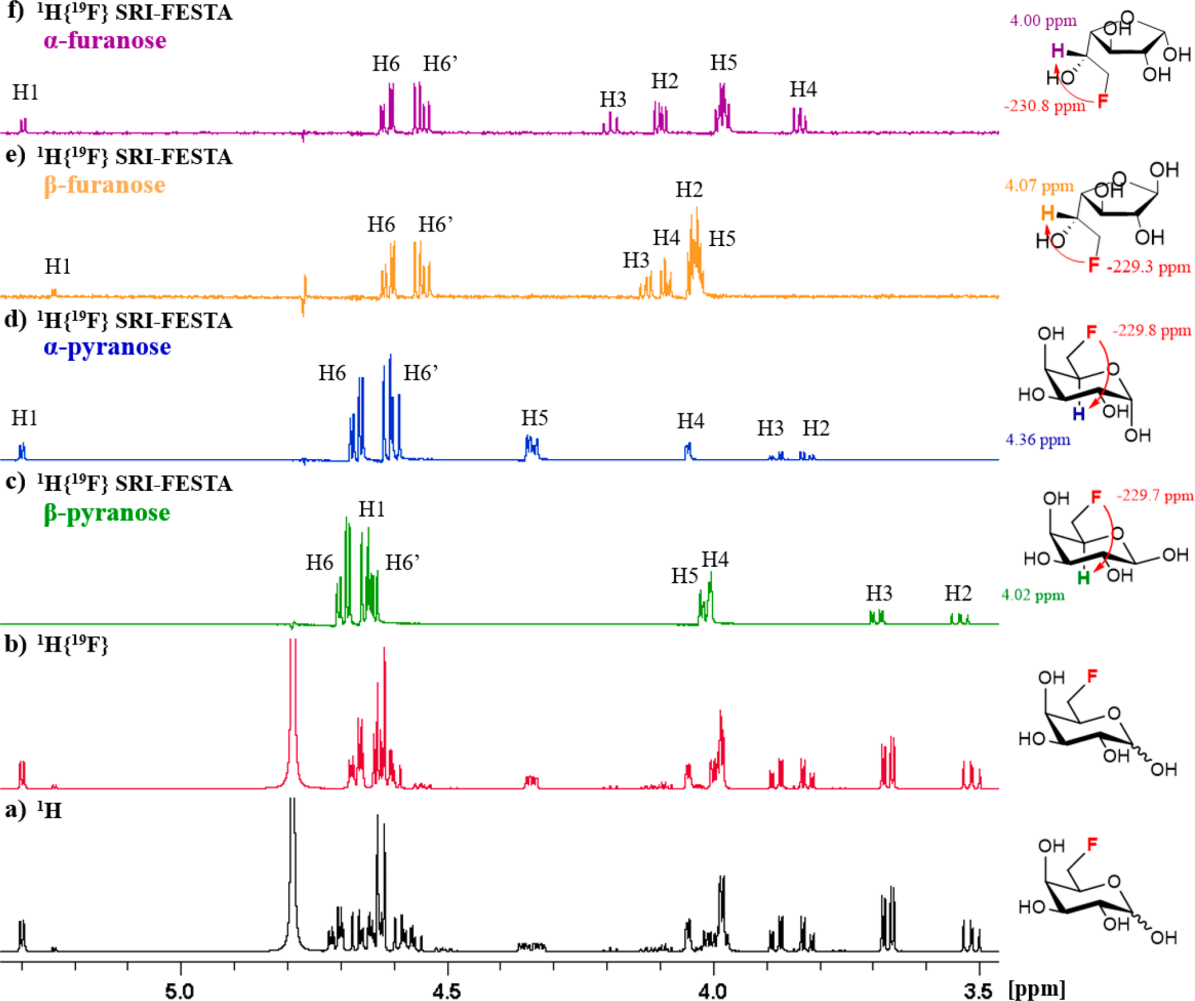
NMR spectra of FDGal-6 in D_2_O, 600 MHz: (a) ^1^H NMR spectrum; (b) ^1^H{^19^F} NMR spectrum; (c) ^1^H{^19^F} SRI-FESTA spectrum of β-pyranose (selection of ^1^H5: mixing time 300 ms); (d) ^1^H{^19^F} SRI-FESTA spectrum of α-pyranose (selection of ^1^H5: mixing time 300 ms); (e) ^1^H{^19^F} SRI-FESTA spectrum of β-furanose (selection of ^1^H5: mixing time 100 ms); (f) ^1^H{^19^F} SRI-FESTA spectrum of α-furanose (selection of ^1^H5: mixing time 100 ms). The following color code is used in this and all subsequent figures: 1D ^1^H NMR showing tautomeric mixtures, black; 1D ^1^H{^19^F} NMR showing tautomeric mixtures, red; β-pyranose subspectra, green; α-pyranose subspectra, blue; β-furanose subspectra, yellow; α-furanose subspectra, purple.

To illustrate the complexity of fluorosugar spectra, the 1D ^1^H spectra (with or without ^19^F decoupling) of FDGal-6 are shown in Figure 3a and 3b. These reveal many overlapped multiplets coming from the various anomers, each with different populations. Also, the characteristic anomeric protons turn out overlapped either with each other (α-pyranose and α-furanose) or with other multiplets of various anomers (β-pyranose). This complicates the use of standard sel-TOCSY experiments. The FESTA experiment (Figure 3c–f) does allow one to separate the subspectra of all anomers with remarkable spectral purity, i.e., absence of any residual signal from other compounds and clean baselines. By disentangling all four anomers, spectral characterization is greatly facilitated. Note that we will apply the color code used in Figure 3 consistently in all examples that follow to indicate the type of anomer subspectrum, irrespective of whether these are obtained via FESTA or sel-TOCSY experiments. Before discussing how FESTA can be exploited for spectral characterization, we first provide a detailed overview of how to obtain such spectra.

### Setting up FESTA for Fluorosugars

**Figure 4:**
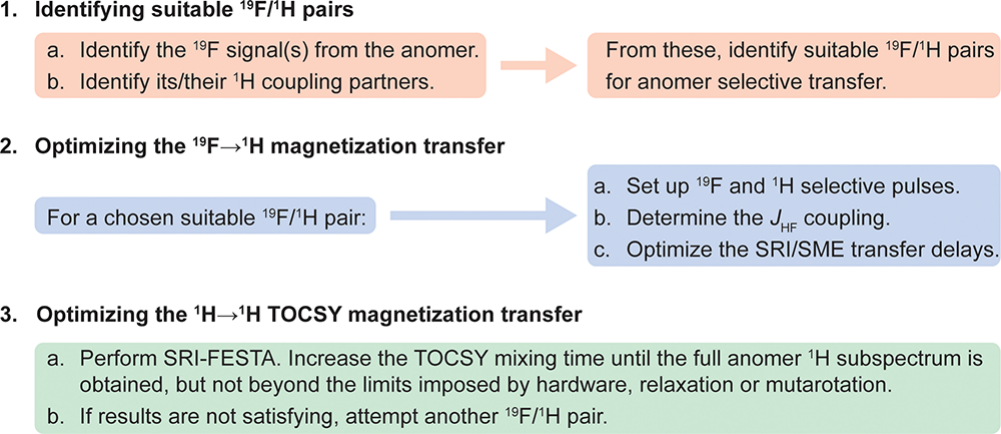
Individual steps for carrying out a FESTA experiment of a particular anomer.

A FESTA spectrum is obtained by applying the pulse sequence developed by Morris and co-workers, of which the Bruker pulse sequence code has been published.^60^ Several parameters need to be considered when setting up the experiments, depending on the spectral properties of the particular fluorosugar anomer. A workflow is shown in Figure 4. First, a suitable pair of fluorine and proton signals associated with a specific anomer needs to be identified for selective polarization transfer and as a starting point for TOCSY propagation. Second, for a chosen pair, the SRI and SME sequences have to be set up to ensure adequate ^19^F → ^1^H magnetization transfer. Lastly, a TOCSY mixing time should be chosen that is sufficiently long for the signal to propagate across the whole ^1^H spin system but also sufficiently short to avoid complications from relaxation or anomer interconversion. Each of these steps will be discussed below, while a detailed protocol is provided in the SI.

### Identifying Suitable ^19^F/^1^H Pairs

In sel-TOCSY experiments, a proton signal is excited by a frequency-selective pulse as a starting point for TOCSY magnetization transfer. In order to obtain a signal from just one anomer, the irradiation bandwidth of this pulse should be narrow enough to avoid hitting signals from other anomers. In other words, the sel-TOCSY experiment is not tolerant to signal overlap between anomers for the initially selected proton resonance. In contrast, the SRI-SME sequence in FESTA generates the starting point ^1^H signal by combining ^19^F- and ^1^H-selective pulses in such a way that magnetization is transferred from a fluorine to a proton if they both resonate in the respective pulse bandwidths and if an *J*_HF_ coupling exists between them. If these pulses are set to a coupled ^19^F/^1^H pair from one anomer, a signal from another anomer only will be generated in the very specific case when it also features a fluorine and a proton that (1) *each* resonates within these selective pulse bandwidths and (2) are coupled. This makes the experiment very tolerant to signal overlap between anomers, as it occurring in either the ^1^H or the ^19^F spectrum alone is insufficient to break down the selectivity. Typically, multiple combinations of ^19^F and ^1^H signals can be identified in one anomer that are not thwarted by such specific double overlap in the ^1^H and ^19^F spectrum (as summarized in Figure S2).

**Figure 5:**
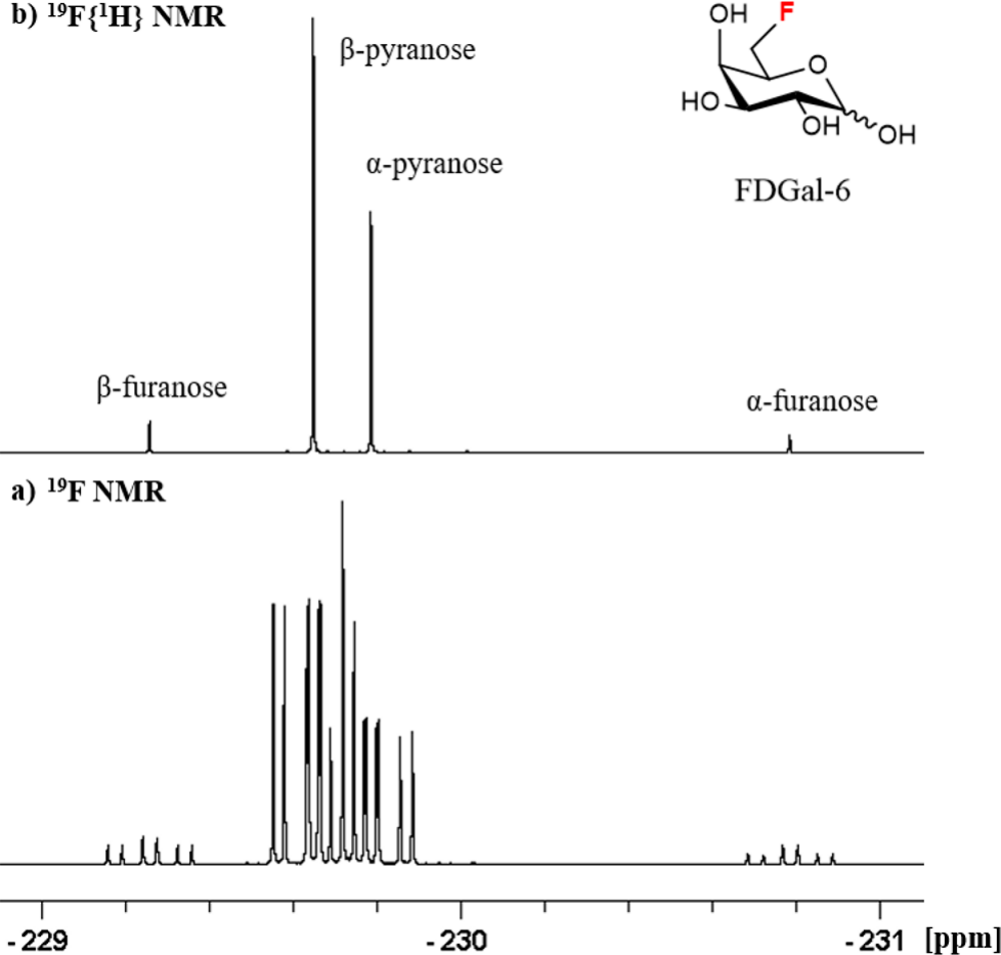
Overlapping ^19^F multiplets of the FDGal-6 pyranose anomers in D_2_O, 565 MHz.

The resolution in the ^19^F spectrum by itself already provides most of the selective power to single out anomers, as the wide dispersion of fluorine signals most of the time results in the absence of spectral overlap. For reducing fluorosugars, the individual anomers indeed typically feature baseline-separated ^19^F resonances. As an illustration, among all of the monosaccharides investigated, the smallest chemical shift differences between the α- and the β-pyranose ^19^F resonances were observed for FDGlc-2 (**1**) and FDGal-6 (**7**) (both 0.14 ppm, Table S1). Also, for monofluorinated reducing mannose and galactose not covered in our study, the ^19^F chemical shifts of the different anomers were reported as well separated.^64^ An important caveat to point out here is that the full multiplet of the ^19^F resonance must be taken into account, since ^1^H decoupling during the selective pulses of the SRI-SME sequence is impractical. This means that the difference in anomer ^19^F resonance frequencies must be more than the multiplet widths. The latter can be very broad given the large size of *J*_HF_ coupling constants, especially for sugars fluorinated at the 6 position (featuring two geminal ^1^H–^19^F couplings) or at an axial position. For all of the sugars investigated, ^19^F multiplet overlap was observed in just three cases (FDGlc-2 (**1**), FDGal-6 (**7**, Figure 5), and F6 in FDGal-46 (**8**)). In principle, off-resonance irradiation of part of the multiplet can be a way to select ^19^F magnetization of just one anomer.^60^ In practice, this was not needed for any of the aforementioned cases featuring fluorine multiplet overlap, since at least one ^1^H coupling partner without overlap to the same anomer could always be exploited for ^19^F → ^1^H transfer (see below). For multifluorinated compounds such as FDGal-46, it is of course also an option to just focus on the fluorine signal that does not overlap.

After choosing a fluorine signal of a particular anomer, a suitable proton coupling partner must be identified. As mentioned above, this choice is only further restricted when the chosen fluorine multiplet overlaps with that of another anomer, meaning that the chosen ^1^H multiplet also should not overlap with protons coupled to the fluorine from the interfering anomer. Given that a fluorine atom nearly always has several proton coupling partners, it should be extremely rare that none of these meet this requirement. The proton coupling partners can be most conveniently identified via a separate ^1^H SRI experiment^60^ that is nonselective in ^1^H (examples given in Figures S3–S5).

**Figure 6:**
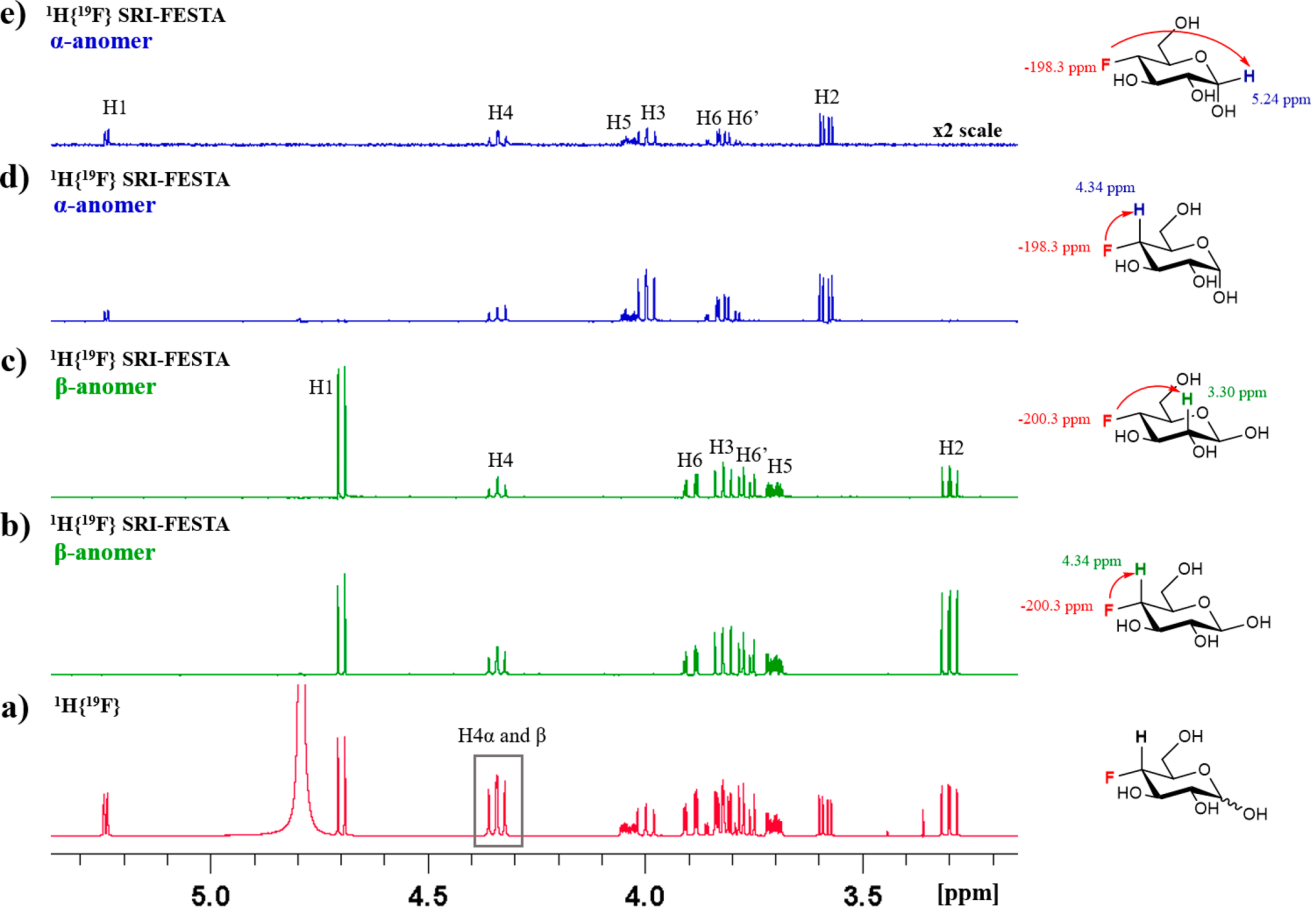
NMR spectra of FDGlc-4, all in D_2_O, 500 MHz: (a) ^1^H{^19^F} NMR spectrum; (b) ^1^H{^19^F} SRI-FESTA spectrum of the β-anomer (selection of H4: mixing time 100 ms); (c) ^1^H{^19^F} SRI-FESTA spectrum of the β-anomer (selection of H2: mixing time 160 ms); (d) ^1^H{^19^F} SRI-FESTA spectrum of the α-anomer (selection of H4: mixing time 100 ms); (e) ^1^H{^19^F} SRI-FESTA spectrum of the α-anomer (selection of H1: mixing time 160 ms).

FDGlc-4 is an example where ^19^F multiplets are resolved, and anomer selectivity can indeed be fully obtained through the ^19^F spectrum. Using H4 selection, clean subspectra can be obtained for all anomers with FESTA (Figure 6b and 6d), despite the H4 protons of both pyranose anomers overlapping at 4.34 ppm (see Figure 6a), meaning these would not have been a viable route for 1D sel-TOCSY. FDGal-6 is an example where there is ^19^F multiplet overlap between both pyranose anomers. Since the H6,H6′ protons of these anomers are also overlapping (Figure 3a and 3b), selecting those would not produce clean anomer subspectra. The H5 protons, with a vicinal coupling to fluorine, can be chosen instead, as those multiplets from both pyranoses are well separated, leading to the pure anomer subspectra shown in Figure 3c and 3d.

### Optimizing the ^19^F → ^1^H Magnetization Transfer

Once suitable coupling partners have been identified, the efficiency of the ^19^F → ^1^H transfer must be considered. There is a preference for proton coupling partners with larger *J*_HF_ coupling sizes as these will shorten the delays needed in the SRI and SME sequences (see the workflow in the Supporting Information for detail). If present, a proton geminal to the fluorine is an obvious choice as their coupling constant is always close to 50 Hz. Also, vicinal protons usually show sizable coupling constants with the fluorine atom, making them viable options. We even found that smaller long-range couplings on the order of 1 Hz can be exploited, although in such cases the sensitivity and optimal transfer delays may be significantly influenced by ^19^F and ^1^H transverse relaxation (T_2_). As an example, we acquired SRI-FESTA spectra of both FDGlc-4 pyranoses using long-range ^4^*J*_HF_ or ^5^*J*_HF_ couplings (Figure 6c and 6e). For the β-anomer, H2 could be selected, which features a ^4^*J*_H2F4_ coupling constant of just 0.9 Hz. This delivered a subspectrum of very similar quality as when selecting the geminal H4 proton (compare Figure 6c and 6d). Similarly, for the α-anomer, H1 was selected with a ^5^*J*_H1F4_ coupling constant of 3.5 Hz (Figure 6e), delivering a spectrum comparable to the F4 → H4 FESTA spectrum (compare Figure 6d and 6e). Having such variety of options available for the starting point of ^1^H magnetization is important when considering limitations to the TOCSY transfer step, which is discussed below.

The efficiency of the initial ^19^F → ^1^H transfer is an important factor determining the sensitivity of the subspectrum. This requires a judicious choice of the SRI/SME transfer delays, which is primarily determined by the *J*_HF_ coupling size. When unknown, this coupling can be conveniently established using the 1D SRI experiment.^60^ For large *J*_HF_ couplings, the optimal SRI/SME transfer delays can then simply be calculated as the delays will typically be short enough so that the effects of T_2_ relaxation can be ignored (see Supporting Information). For small *J*_HF_ couplings, ^1^H and ^19^F T_2_ relaxation during the longer delays can lead to significant signal loss, and shorter delays may be chosen as a compromise. In some cases, multiple proton coupling partners to a single fluorine are simultaneously irradiated with the selective pulse, for instance, when their multiplets overlap. In such case, multiple *J*_HF_ couplings will evolve at the same time. The consequence of this is that ^19^F polarization can never be fully transferred to the ^1^H spins as it is partially lost to the formation of double antiphase ^1^H–^19^F magnetization during the SRI sequence. Furthermore, if these selected protons are also coupled among each other, ^1^H–^1^H coupling evolution during the SME sequence results in further losses to the final in-phase ^1^H magnetization. Although these complications are not critical, it is clear that selection of a single ^1^H/^19^F pair is preferable for obtaining optimal sensitivity. In the case where such complications, or T_2_ relaxation, come into play, the optimal transfer delay can be best obtained by experimental optimization using an SRI-FESTA sequence with zero TOCSY mixing time rather than by calculation.

### Optimizing the ^1^H → ^1^H TOCSY Magnetization Transfer

The initial ^1^H magnetization is transferred to the rest of the spin system via a TOCSY mixing step. This requires the application of a radio frequency spinlock of a certain duration τ_m_ (in our work typically ranging between 60 and 300 ms). The longer the τ_m_, the greater the likelihood that the initial proton’s magnetization will have propagated across the whole spin system. However, this competes with relaxation during the spinlock (spin relaxation in the rotating frame under an applied radio frequency field, T_1_ρ), which decreases the overall amount of signal detected. In addition, when applied for too long, the radio frequency power continuously applied during the spinlock may cause damage to the NMR probe head as well as sample heating. These factors thus impose a practical limit on τ_m_.

**Figure 7:**
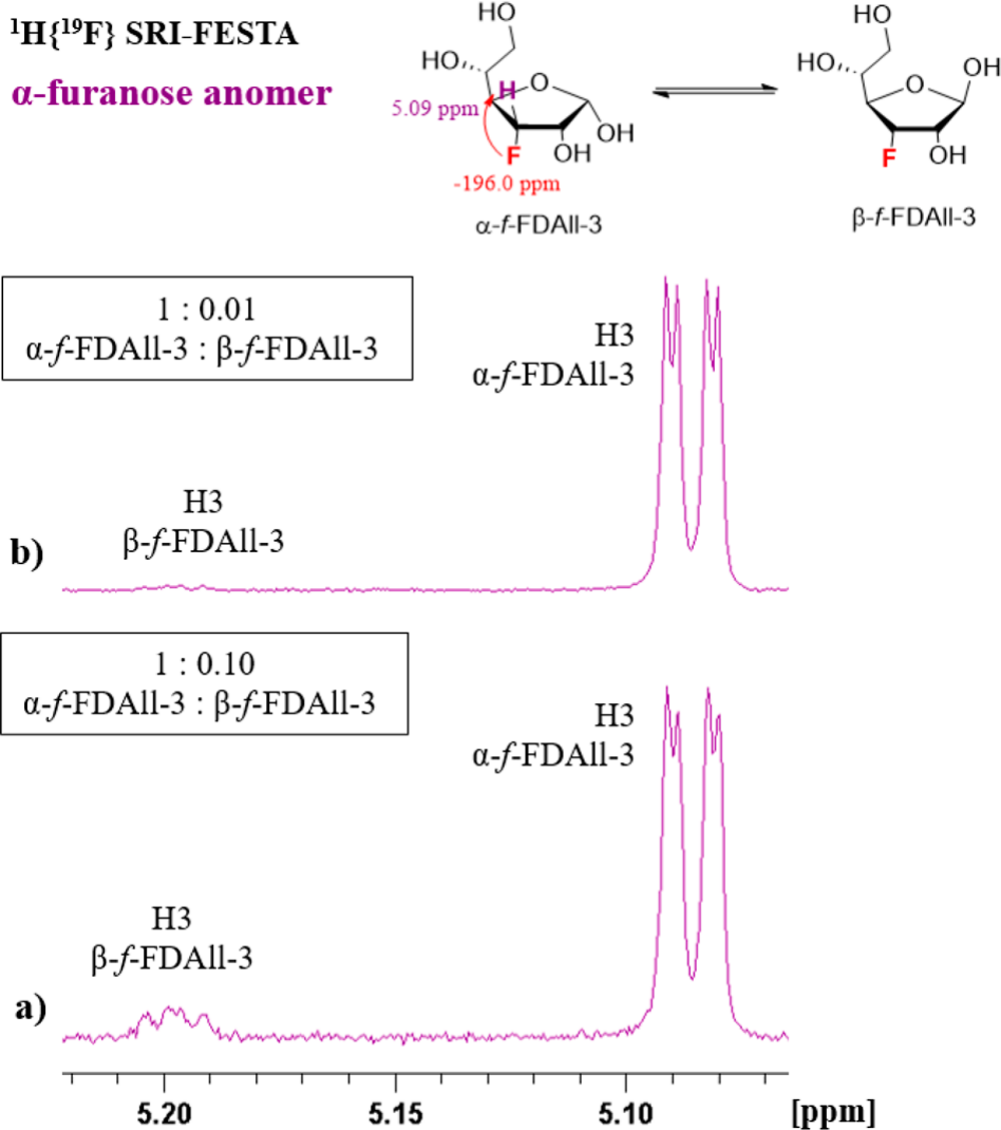
^1^H{^19^F} SRI-FESTA spectra expansion at H3 of α-*f*-FDAll-3 in D_2_O, 600 MHz: (a) selection of ^1^H3: mixing time τ_m_ = 0 ms and a total ZQS pulse duration of 85 ms; (b) selection of ^1^H3: mixing time τ_m_ = 0 ms and total ZQS pulse duration of 20 ms.

An additional complication is present specifically for mixtures of compounds in equilibrium: interconversions taking place during the long TOCSY mixing step. In FESTA, the spinlock is applied during a *z*-filter element of duration Δ (Δ > τ_m_), which also contains zero-quantum coherence suppression (ZQS) frequency-swept pulses^65^ flanking the spinlock, each typically several tens of milliseconds long. If the anomer interconversion rate becomes comparable to the inverse of Δ, ^1^H magnetization also transfers, thus resulting in loss of purity of the resulting subspectrum. For interconverting carbohydrate anomers, this was initially not expected to be a practical limitation given the anomerization rates are relatively slow. For all fluorosugars investigated in this work, the pyranose subspectra indeed showed no signal spillover due to mutarotation when applying TOCSY mixing times from 60 to 300 ms (examples in Figures S6–S13). In contrast, the furanose subspectra were contaminated with signals coming from the other furanose anomer in a number of cases (FDAll-3 and FDGal-3), indicating faster anomerization rates. Incidentally, these observations are consistent with investigations showing that for aldohexoses, α-furanose to β-furanose exchange is kinetically favored compared to α-furanose−β-pyranose and α-pyranose−β-pyranose exchange.^66,67^ The faster interconversion kinetics of the furanoses compared to the pyranoses are caused by the higher angular strain, as evidenced by the former’s inherent flexibility.^68^ The most effective countermeasure against rapid mutarotation is to reduce the exchange rate by lowering the sample temperature. Other options are keeping the *z*-filter time Δ to a minimum, either by keeping *τ*_m_ low if spinlock magnetization transfer is sufficiently efficient or by keeping the duration of the ZQS pulses as short as possible while still having adequate ZQ-artifact suppression. This is illustrated in Figure 7a, which shows an SRI-FESTA spectrum of the α-furanose anomer of FDAll-3 (F3 → H3) without spinlock (*τ*_m_ = 0) with a total ZQS pulse duration of 85 ms (which was the default duration used in this work). Besides the expected H3 signal of the α-furanose, the signal of the β-furanose is present in a 10:1 α:β ratio. By reducing the total ZQS pulse duration to a bare minimum of 20 ms, this ratio is reduced to 100:1 (Figure 7b, see Figure S14 for a similar example with the corresponding β-anomer).

**Figure 8:**
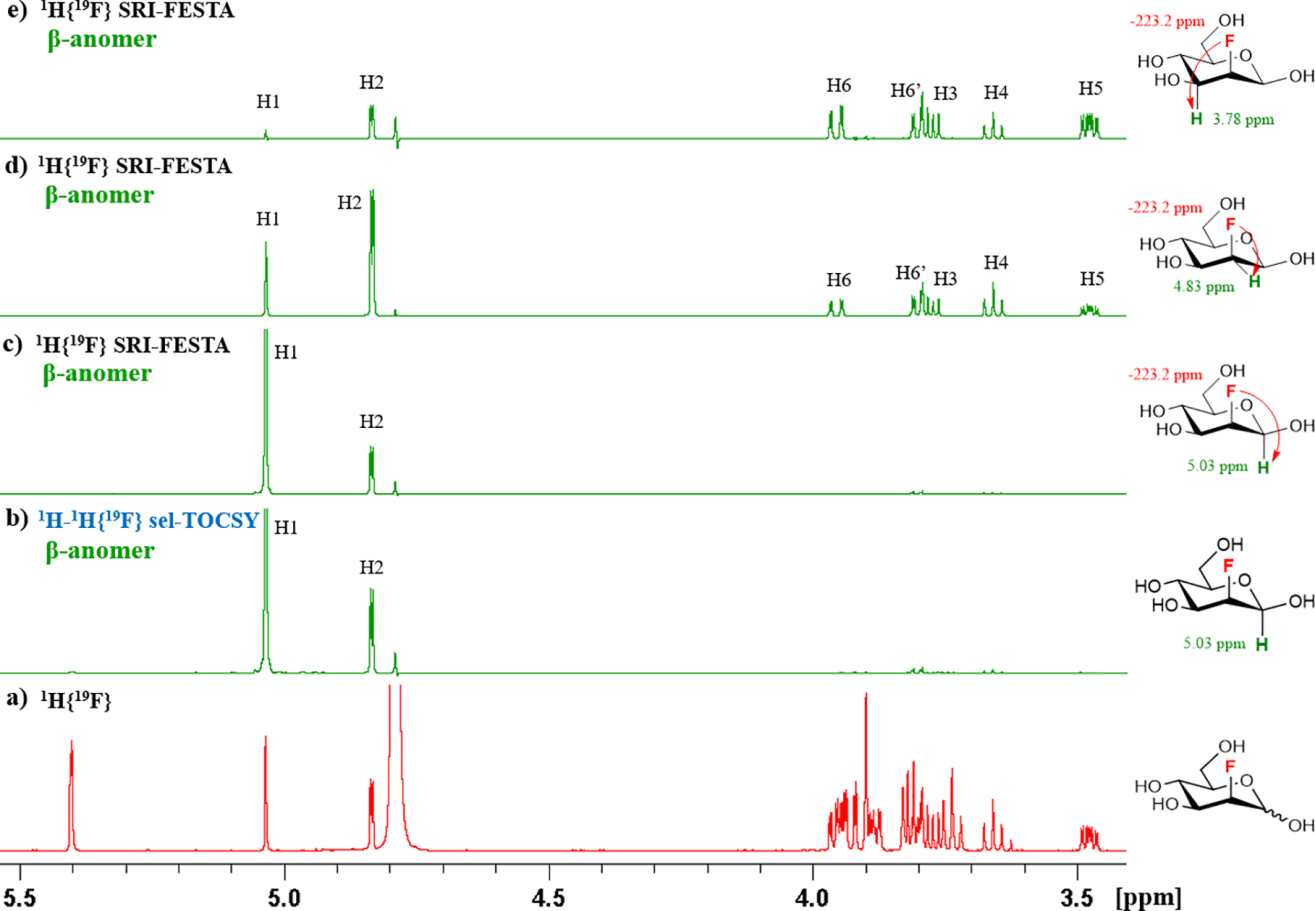
NMR spectra of FDMan-2, all in D_2_O, 600 MHz: (a) ^1^H{^19^F} NMR spectrum; (b) ^1^H–^1^H{^19^F} sel-TOCSY NMR spectrum of the β-anomer (selection of ^1^H1); (c–e) ^1^H{^19^F} SRI-FESTA spectrum of the β-anomer ((c) selection of ^1^H1; (d) selection of ^1^H2; (e) selection of ^1^H3). All mixing times 200 ms.

Although mixing times are preferably kept short for all of the aforementioned reasons, the minimum duration needed is imposed by the ^1^H–^1^H coupling network of the fluorosugar. The efficiency of TOCSY depends on the sizes of the *J*_HH_ couplings that mediate the magnetization transfer across the spin system. For certain carbohydrates featuring small *J*_HH_ couplings, this can be problematic. Martins et al. have reported that sel-TOCSY experiments starting from H1 resulted in incomplete magnetization propagation beyond H4 and H2 for d-galactose and d-mannose pyranoses, respectively, even with long mixing times (i.e., up to 100 ms investigated).^41^ Also, Bax et al. have reported incomplete magnetization using sel-TOCSY experiments for d-galactose even at τ_m_ = 200 ms.^43^ This is due to the presence of axial hydroxyl groups, with the corresponding geminal protons thus residing in equatorial position, leading to small successive ^3^*J*_eq–eq_ and ^3^*J*_ax–eq_ couplings in these sugars. When OH groups are substituted with the more electron-withdrawing fluorine, a further reduction of the magnitude of ^3^*J*_HH_ is expected, depending on the fluorine-bearing carbon stereochemistry, as described by the Haasnoot–Altona equation.^69^ The ^3^*J*_eq–eq_ and ^3^*J*_ax–eq_ coupling constants present in the pyranose anomers of mannose-, galactose-, and allose-configured deoxyfluorinated monosaccharides are given in Table 1. In sel-TOCSY experiments, the initially selected proton is typically the well-resolved anomeric H1 proton, meaning the magnetization has to propagate across the maximum number of relayed steps. This maximizes the chance that such successive small couplings create critical bottlenecks for the TOCSY propagation. FDMan-2 provides a clear illustration of this issue. Both a sel-TOCSY experiment starting from H1 and an SRI-FESTA experiment starting from a F2 → H1 transfer, each with a 100 ms mixing time, produced no signals beyond H2 in either experiment for the β-anomer (Figure 8b and 8c). The successive small ^3^*J*_H1ax–H2eq_ and ^3^*J*_H2eq–H3ax_ couplings thus completely block the TOCSY transfer to the rest of the spin system. In contrast, full anomer subspectra for the α-anomer were obtained, in line with the slightly higher ^3^*J*_H1eq–H2eq_ coupling (Table 1), albeit with low signal intensities as shown in Figure S17.

**Table 1:** ^3^*J*_HH_ Coupling Constants (in Hz) Involving Equatorial C–H Bonds Hampering TOCSY Transfer

entry	sugar	pyranose anomer	^3^*J*_H1–H2eq_	^3^*J*_H2eq–H3ax_
1	FDMan-2	α	1.8	2.6
		β	a	2.5
			^3^*J*_H2ax–H3eq_	^3^*J*_H3eq–H4ax_
2	FDAll-3	α	2.6	2.4
		β	2.2	2.1
			^3^*J*_H3ax–H4eq_	^3^*J*_H4eq–H5ax_
3	FDGal-3	α	3.5	1.1
		β	3.6	0.9
4	FDGal-6	α	3.3	1.0
		β	3.6	1.1
5	FDGal-46	α	2.7	a
		β	2.8	a

a Broad singlet was observed.

The high selective power of the SRI pulse sequence in FESTA makes it straightforward to start from protons halfway in the fluorosugar spin system, which more frequently resonate in overlapped regions. The resulting reduced number of relayed steps should make it easier for the magnetization to spread across the full spin system. For β-*p*-FDMan-2, either F2 → H2 or F2 → H3 transfer can be used. This completely changes the dynamics of the TOCSY propagation compared to the F2 → H1 transfer, as only the H1 proton’s signal intensity depends on the encumbering ^3^*J*_H1–H2_ coupling. Both experiments now indeed do reveal the full subspectrum (Figure 8d and 8e), including the H1 proton.

**Figure 9:**
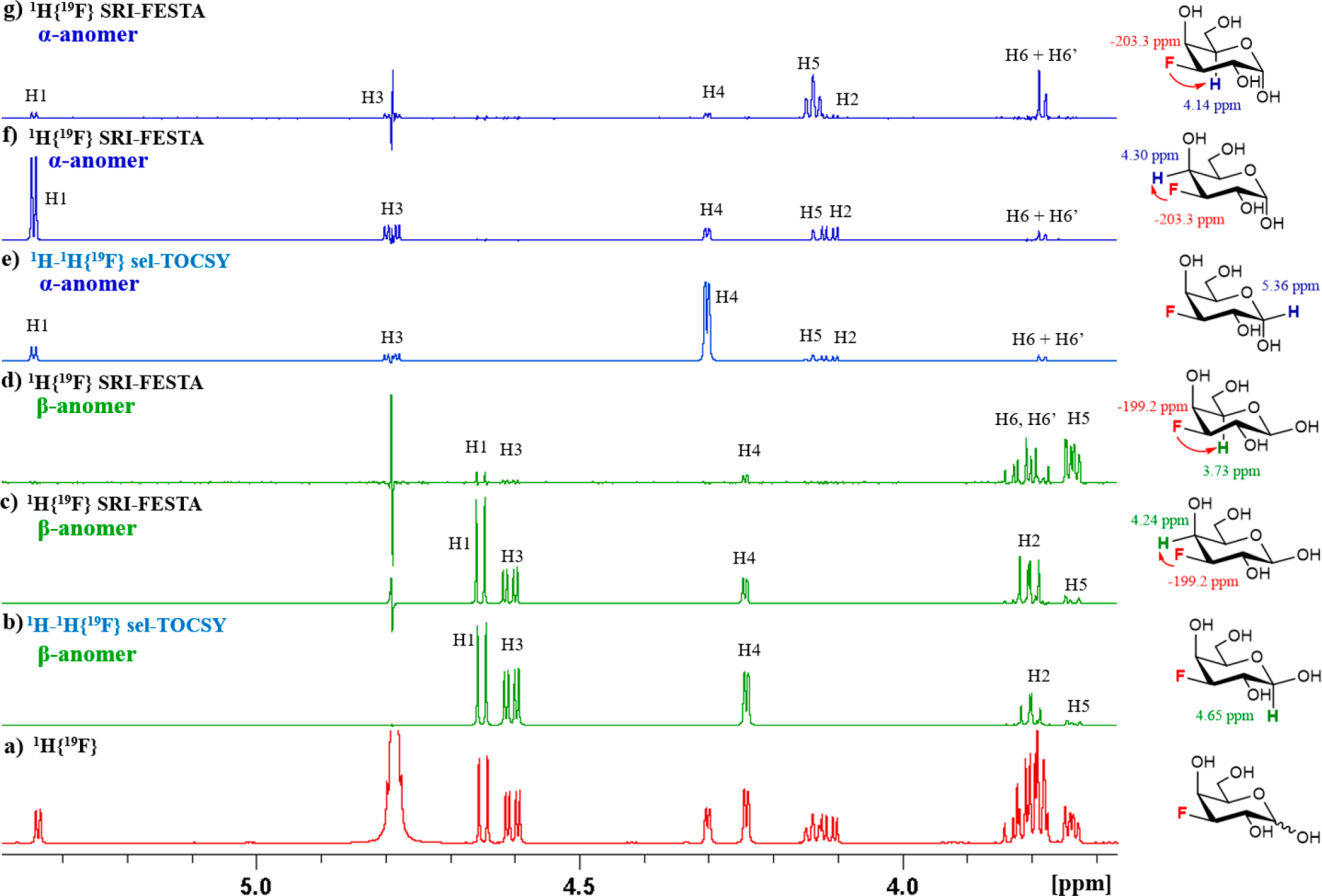
NMR spectra of FDGal-3 in D_2_O, 600 MHz: (a) ^1^H{^19^F} NMR spectrum; (b) ^1^H–^1^H{^19^F} sel-TOCSY NMR spectrum of the β-anomer (selection of ^1^H1); (c) ^1^H{^19^F} SRI-FESTA spectrum of the β-anomer (selection of ^1^H4); (d) ^1^H{^19^F} SRI-FESTA spectrum of the β-anomer (selection of ^1^H5); (e) ^1^H–^1^H{^19^F} sel-TOCSY NMR spectrum of the α-anomer (selection of ^1^H1); (f) ^1^H{^19^F} SRI-FESTA spectrum of the α-anomer (selection of ^1^H4: mixing time 200 ms); (g) ^1^H{^19^F} SRI-FESTA spectrum of the α-anomer (selection of ^1^H5). All mixing times 300 ms except where indicated.

A similar issue arises for galactose-configured pyranoses, which also feature two successively small couplings, ^3^*J*_H3ax–H4eq_ and ^3^*J*_H4eq–H5ax_ (Table 1). As mentioned above, fluorination generally exacerbates this situation, with FDGal-46 having the smallest couplings of the three fluorogalactoses investigated here due to the axial C4–F bond, as explained by the β-effect described by Haasnoot and Altona.^70,71^ For 3-deoxy-3-fluorogalactose, sel-TOCSY experiments starting from H1 yielded incomplete subspectra for both pyranose anomers (Figure 9b and 9e). Both anomers showed only clear signals for the H1–H4 protons, while the H5 and H6 protons were either absent (α-pyranose) or very weak (β-pyranose), even for mixing times up 300 ms. Also, in SRI-FESTA spectra starting from the geminal F3 → H3 transfers, only faint responses beyond H4 were obtained (not shown), showing that indeed the successive ^3^*J*_H3ax–H4eq_ and ^3^*J*_H4eq–H5ax_ couplings create a bottleneck that is too narrow for effective magnetization transfer. For the α-anomer, the vicinal F3 → H4 SRI transfer circumvents the need for the TOCSY transfer to pass through both small couplings. A 200 ms spinlock indeed now did result in sufficient signal for H5 and H6/6′ as well as the rest of the spin system (Figure 9f) (Note that the H6, H6′ chemical shifts of the α-pyranose form are degenerate, leading to a distorted multiplet). By exploiting a long-range ^4^*J*_H5–F3_ coupling, an SRI-FESTA spectrum with F3 → H5 transfer was also measured. This only delivered the H5 and H6 responses with good signal intensity even at 300 ms spinlock (Figure 9g), again due to the successive ^3^*J*_H3ax–H4eq_ and ^3^*J*_H4eq–H5ax_ couplings blocking efficient TOCSY transfer. For the β-anomer, the F3 → H4 SRI-FESTA spectrum did not deliver sufficient signal for the H5 and H6 protons (Figure 9c), in contrast to the F3 → H5 transfer (Figure 9d). For FDGal-46, sel-TOCSY and SRI-FESTA spectra yielded similar results (see Figure S18).

In allopyranose sugars, there are also two successive vicinal H_ax_–H_eq_ couplings (^3^*J*_H2–H3_ and ^3^*J*_H3–H4_). In the case of FDAll-3 (**10**), SRI-FESTA (F3 → H3) leads to full TOCSY propagation as the transfer to H3 avoids magnetization over the two small coupling constants. Even with a 100 ms mixing time, clean anomer subspectra were obtained (see Figure S19).

To summarize, practical limits such as T_2_ relaxation and anomer interconversion impose a preference for mixing times that remain as short as possible. In this respect, the anomeric H1 proton, which is typically exploited in sel-TOCSY, is the least favorable starting point, especially in the presence of successive small coupling constants. A key advantage of SRI-FESTA is that protons halfway in the fluorosugar spin system can easily be selected, decreasing the chance that small *J*_HH_ couplings clog TOCSY propagation. If needed, multiple F → H transfers can be obtained to reveal the full ^1^H spin system, or for polyfluorinated sugars, different fluorines can be used (example in FDGlc-46, Figure S20).

### SRI-FESTA as an Aid for ^1^H NMR Spectral Assignment

**Figure 10:**
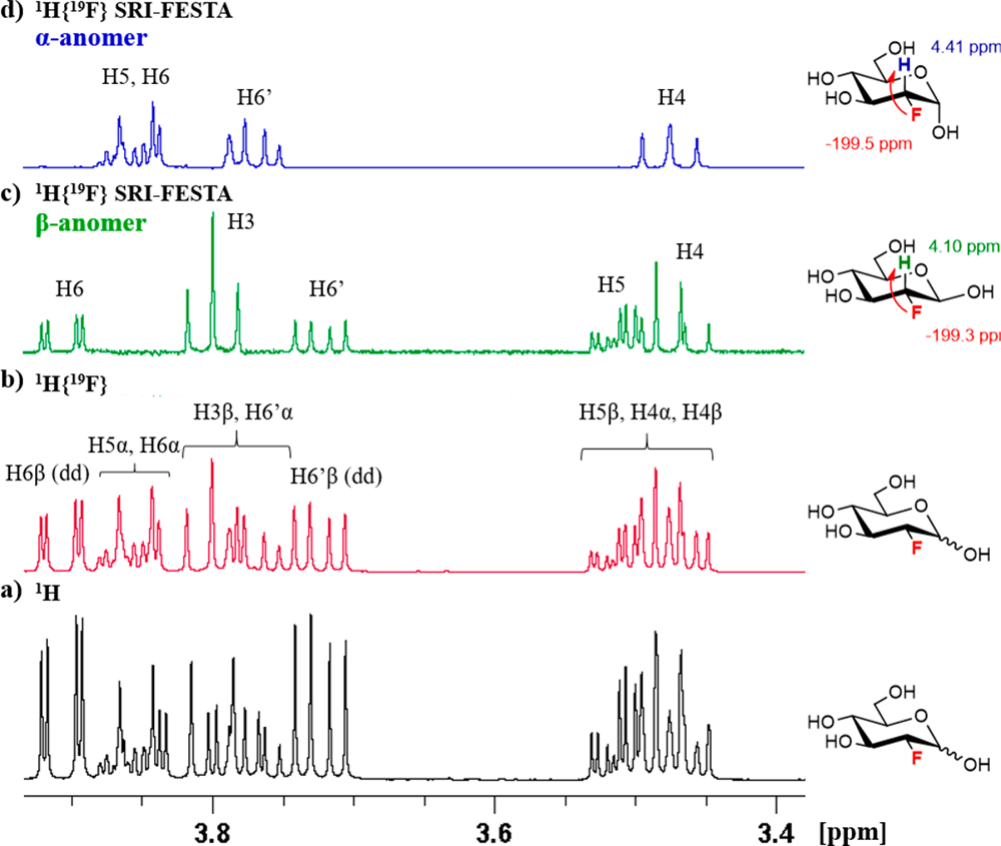
NMR spectra expansion at 3.90–3.40 ppm of FDGlc-2, all in D_2_O, 500 MHz: (a) ^1^H NMR spectrum; (b) ^1^H{^19^F} NMR spectrum; (c) ^1^H{^19^F} SRI-FESTA spectrum of the β-anomer (selection of ^1^H2); (d) ^1^H{^19^F} SRI-FESTA spectrum of the α-anomer (selection of ^1^H2). All mixing times 100 ms.

The ability to separate the ^1^H NMR spectra of reducing sugar anomers provides new opportunities for characterization, especially when overlapping multiplets prevent coupling constant determination. This is illustrated with the full characterization of fluorosugar 2-deoxy-2-fluoro-d-glucose, which exists as a 45/55 α/β-pyranose anomeric mixture. The acquired ^1^H and ^1^H{^19^F} spectra of FDGlc-2 feature overlapping resonances in the regions 3.92–3.70 and 3.53–3.46 ppm (see Figure 10a and 10b). Although these regions can be assigned via 2D COSY, the spectral overlap prevents measurements of the coupling constants (*J*_HH_ and *J*_HF_). In contrast, the acquired ^1^H{^19^F} SRI-FESTA subspectra for the pyranose anomers (shown in Figure 10c and 10d) exhibit almost fully resolved multiplets. This allowed full ^1^H NMR characterization (chemical shifts and *J* couplings), which filled various gaps in the existing literature of this important fluorosugar (Table S3).^72−77^ Similarly, the ^1^H spectral assignments reported for the pyranose forms of FDMan-2,^73,74,77^ FDGal-3,^75,78^ FDGal-46,^11,79^ and FDAll-3^80^ could also be completed with minor corrections (summarized in Table S3).

**Figure 11:**
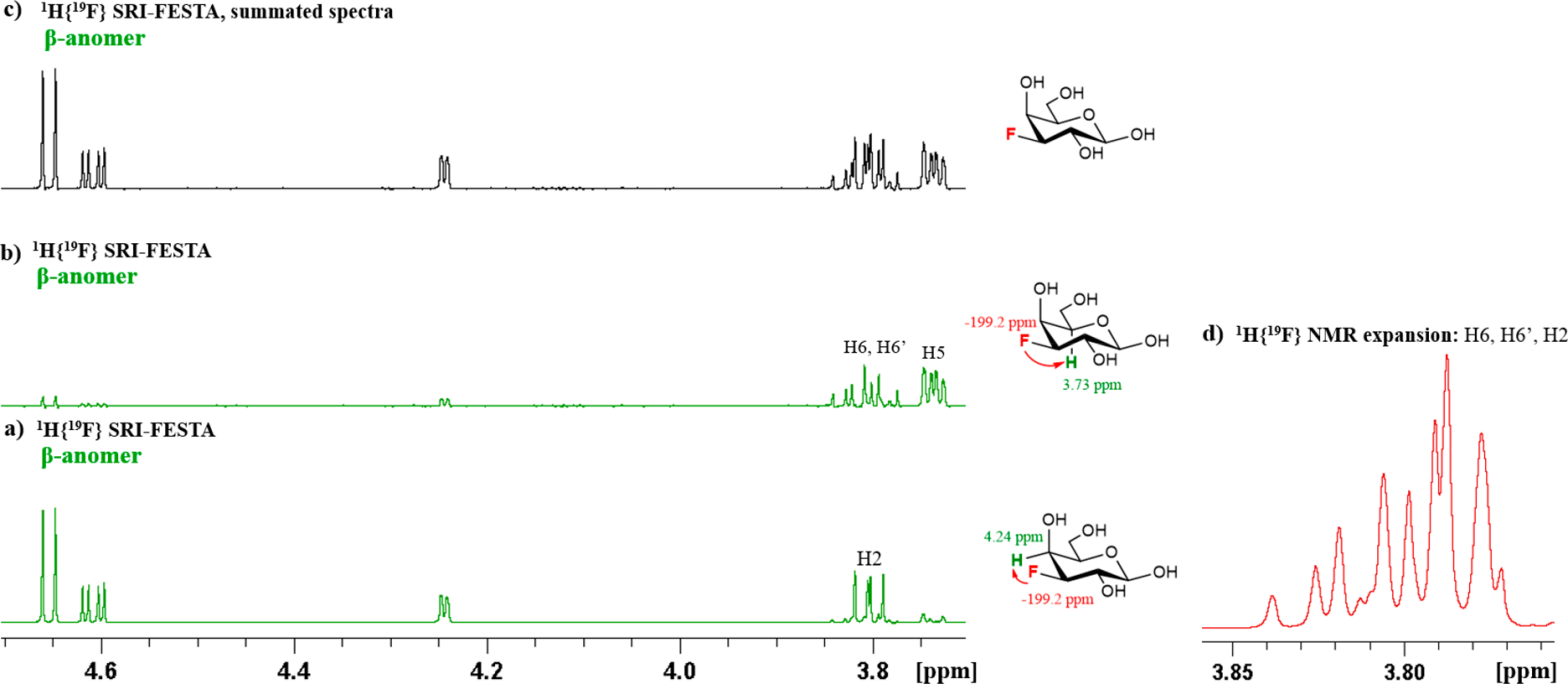
Obtaining all *J* values for the three-proton multiplets by selecting different F3 → H proton magnetization transfer partners: (a) ^1^H{^19^F} SRI-FESTA spectrum of β-*p*-FDGal-3 (selection of ^1^H4); (b) ^1^H{^19^F} SRI-FESTA spectrum of β-*p*-FDGal-3 (selection of ^1^H5); (c) summed spectra of a and b; (d) ^1^H{^19^F} spectrum expansion at 3.83–3.75 ppm. All in D_2_O, 600 MHz. All mixing times 300 ms.

Within pure anomer subspectra, multiplet overlap remains possible. This is the case for FDGal-3, where there is overlap between H2 and H6/H6′ for the β-anomer. In this case, the small galactose ^3^*J*_H4–H5_ coupling constant that impedes TOCSY transfer (see above) can be exploited for characterization purposes combined with the ability of FESTA to start TOCSY mixing from different protons. ^1^H{^19^F} SRI-FESTA subspectra starting from F3 → H4 and F3 → H5 transfers lead to only partial anomeric subspectra with just H2 and H6/H6′ visible, respectively (Figure 11a and 11b). This allowed facile extraction of coupling constants from these multiplets. If so desired, the sum spectrum can be obtained from these two complementary subspectra, revealing the full pure β-anomer subspectrum (Figure 11c).

**Figure 12:**
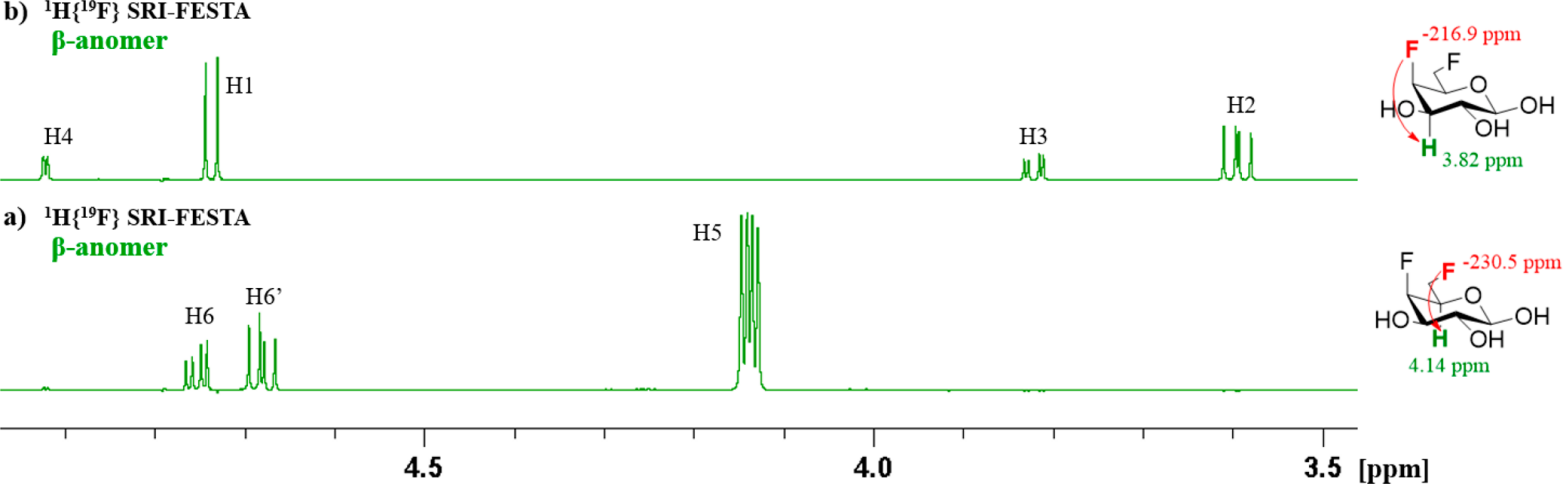
Obtaining all multiplets of β-*p*-FDGal-46 by utilizing both fluorines. ^1^H{^19^F} SRI-FESTA spectra in D_2_O, 600 MHz: (a) selection of F6 → H5; (b) selection of F4 → H3. Both mixing times 100 ms.

Similarly, the TOCSY magnetization propagation is hindered in 4,6-difluorinated galactose by the small ^3^*J*_H3ax–H4eq_ and ^3^*J*_H4eq–H5ax_ couplings (Table 1). An obvious solution is to make good use of both fluorine atoms. SRI-FESTA using the F6 → H5 transfer yields just the H5 and the H6 protons (Figure 12a), while the SRI-FESTA using the F4 → H3 transfer provides all signals from H1 to H4 (Figure 12b, see Figure S18 for the full data set).

### SRI-FESTA Spectra of Minor Furanose Tautomers

Another illustration of the power of FESTA for the full characterization of mixtures is that clean spectra of the minor furanose tautomers can be obtained and characterized. Out of the 10 fluorosugars investigated here, three featured detectable furanose forms. The populations of furanose forms are generally very low (FDGal-3 0.4% and 0.9% for α- and β-furanose, FDGal-6 3.5% and 5.0% for α- and β-furanose, and FDAll-3 0.6% and 0.5% for α- and β-furanose), meaning that they are overshadowed by the intense pyranose signals in regular 1D ^1^H spectra. Indeed, the furanose signal intensities can be on the same order of magnitude as the ^13^C satellites of the pyranose signals and of impurities or residual solvent signals, exacerbating the chance of overlap and complicating the use of sel-TOCSY experiments. Thanks to the double selectivity of the SRI-FESTA experiment, clean pure furanose ^1^H subspectra can be provided. Once the ^19^F signal is identified, an SRI experiment can be performed as usual to detect the location of the proton coupling partners, which due to overlap might not be obvious from the standard 1D ^1^H NMR spectrum. We will discuss here all three fluorosugars with detectable furanose forms. To the best of our knowledge, this is the first report of ^1^H spectral assignments for FDGal-6 and FDAll-3 furanoses.

Unlike the pyranose forms, which possess well-defined chair conformations, it is less obvious how to distinguish the furanose anomers. Some general trends exist that can be used.^81−83^ The chemical shifts of anomeric protons of the 1,2-cis anomers (α-furanoses) are usually shifted downfield relative to those of the 1,2-trans anomers (β-furanoses) and that α-furanoses usually show a doublet splitting with a ^3^*J*_H1–H2_ coupling constant of around 3–5 Hz with a lower coupling constant (0–2 Hz) for β-furanoses. This assignment approach based on ^1^H NMR anomeric spectral data was also recently used for the NMR assignment of d-glucofuranoses.^28^ Based on this, in the case of FDGal-6 furanose, the more deshielded galactofuranose H1 resonance was assigned as that of the α-furanose. This assignment corroborated with its slightly higher ^3^*J*_H1–H2_ coupling constant compared to that of the other furanose (3.9 vs 2.7 Hz). This leads to the α-furanose having a ^19^F chemical shift of −230.8 ppm and the β-furanose of −229.3 ppm. According to this assignment, the β-furanose is the more abundant furanose tautomer, which is also the case for nonfluorinated galactose.^84^

**Figure 13:**
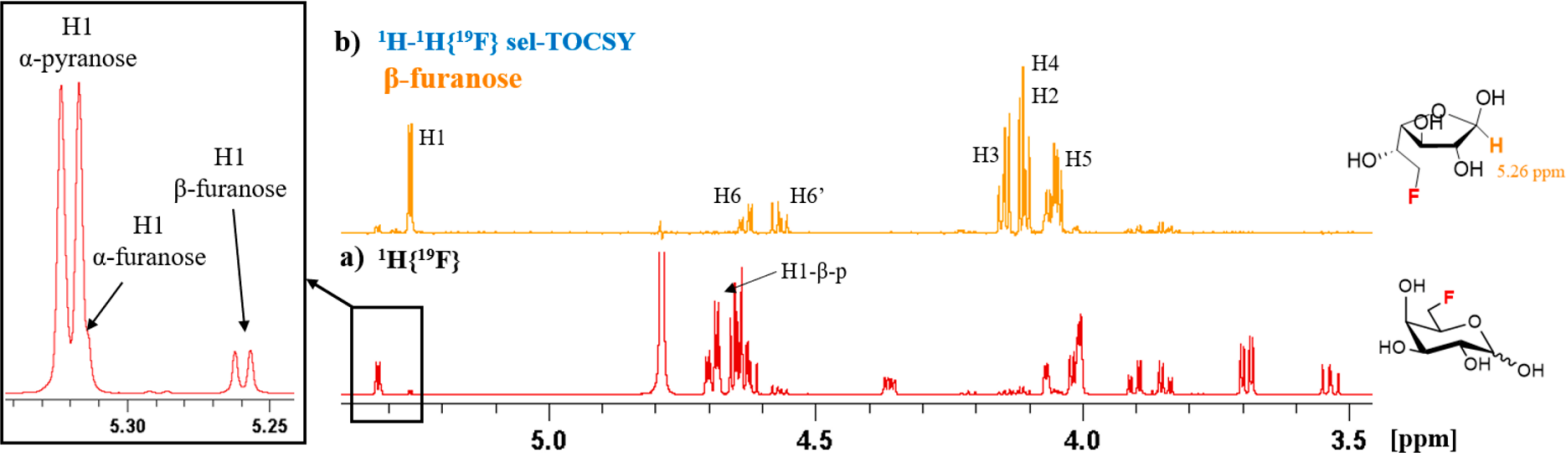
(a) ^1^H{^19^F} NMR spectrum of FDGal-6, and the expansion showcasing the overlap of α-*p*-FDGal-6 and α-*f*-FDGal-6 anomeric protons; (b) ^1^H–^1^H{^19^F} sel-TOCSY NMR spectrum of β-*f*-FDGal-6 (selection of ^1^H1: mixing time 200 ms). All in D_2_O, 600 MHz.

In the ^1^H{^19^F} NMR spectrum of FDGal-6 (Figure 13a), the signals of the anomeric protons of α-furanose and α-pyranose overlap. Conventional sel-TOCSY experiments starting from the H1 protons therefore cannot provide the β-furanose subspectrum (see Figure 13b). The SRI-FESTA spectra (using F6 → H5 transfers) easily yielded clean and pure anomer subspectra for all FDGal-6 pyranoses and furanoses (Figure 3). While 300 ms mixing times were needed for the pyranose forms due to the small ^3^*J*_H3ax–H4eq_ and ^3^*J*_H4eq–H5ax_ couplings, 100 ms suffices for the furanose forms to obtain all very well resolved proton environments (see Figures S10–S13 for experiments at varying mixing times for all FDGal-6 mixture components). Indeed, given the very different set of dihedral angles, the galactofuranoses feature no TOCSY propagation bottleneck due to successive small coupling constants with ^3^*J*_H4–H5_ couplings of 4.5 and 3.2 Hz for the α- and β furanoses, respectively.

**Figure 14:**
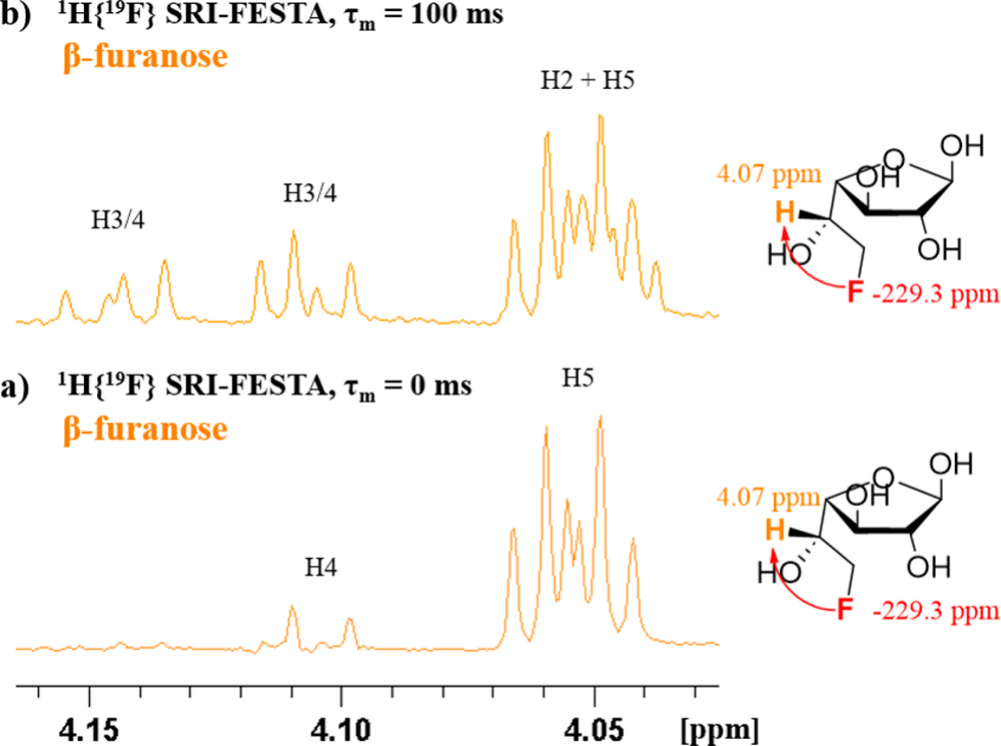
^1^H{^19^F} SRI-FESTA spectra of β-*f*-FDGal-6 in D_2_O, 600 MHz: (a) selection of ^1^H5: mixing time 0 ms; (b) selection of ^1^H5: mixing time 100 ms.

Regarding the analysis of the ^1^H NMR spectra, while the ^1^H NMR chemical shift assignment of the α-furanose anomer of FDGal-6 was straightforward from multiplet analysis in the SRI-FESTA spectrum, this was not the case for the β-furanose due to overlap of the H2 and H5 protons. This overlap initially made it impossible to extract couplings between these protons and the H3β and H4β protons (Figure 14b), making the assignment of the latter two ambiguous. Two-dimensional COSY experiments did not help due to the low-intensity cross-peaks of furanoses overlapping with the intense cross-peaks of pyranoses. As discussed above, SRI-FESTA spectra with incomplete TOCSY propagation can be a useful tool to obtain subspectra with only a limited number of multiplets. Here, an SRI-FESTA of the β-furanose using the F6 → H5 transfer with a TOCSY mixing time set to 0 ms (Figure 14a) was recorded, revealing only the H5 multiplet and a slight residual appearance of the multiplet at 4.13 ppm. The latter observation already suggested that this resonance corresponds to the H4 proton. This spectrum thus allowed extraction of the coupling constants involving H5, confirming the assignments of H4.

**Figure 15:**
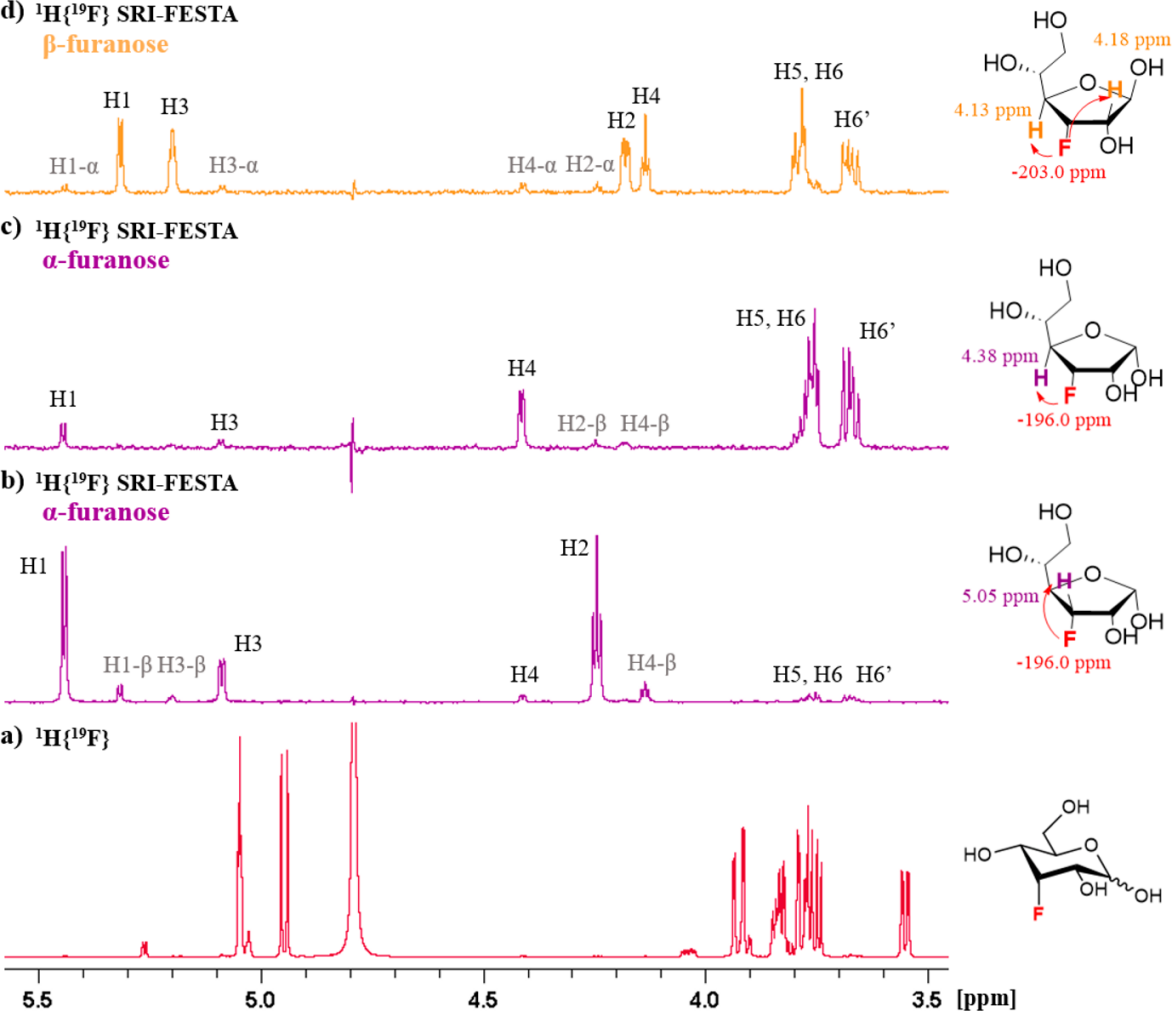
NMR spectra of FDAll-3 furanoses in D_2_O, 600 MHz: (a) ^1^H{^19^F} NMR spectrum of FDAll-3; (b) ^1^H{^19^F} SRI-FESTA spectrum of α-furanose (selection of ^1^H3); (c) ^1^H{^19^F} SRI-FESTA spectrum of α-furanose (selection of ^1^H4); (d) ^1^H{^19^F} SRI-FESTA spectrum of β-furanose (selection of ^1^H2 + ^1^H4). (b–d) Mixing time 200 ms, total ZQS pulse duration of 20 ms.

Similarly, SRI-FESTA furanose subspectra for FDAll-3 were extracted (Figure 15). As already discussed above (Figure 7), fast interconversion between its two furanose forms causes signal exchange between both subspectra. For the α-furanose form of FDAll-3, TOCSY propagation starting from an F3 → H3 SRI transfer proved incomplete, even at τ_m_ = 300 ms (see Figure S15). This is due to the small ^3^*J*_H4–H3_ coupling (1.4 Hz), providing only H1 to H3 protons adequately visible (Figure 15b, using a 200 ms mixing time). Such a long mixing time did result in a significant contamination with β-furanose signals, despite the minimal ZQS pulse duration. The missing α-furanose peaks could be obtained from an SRI-FESTA F3 → H4 experiment (Figure 15c). If so desired, the full spectrum of α-*f*-FDAll-3 can then be obtained by simply summing the two spectra (not shown). For the β-anomer, for which ^3^*J*_H4–H3_ is larger (2.9 Hz), the full anomer subspectrum could be obtained with an F3 → H3 transfer even at more modest mixing times (τ_m_ = 100 ms, see Figures S21a and S16). Nevertheless, the F3 → H2 + H4 transfer (with H2 and H4 multiplets overlapped) provided better quality spectra (τ_m_ = 100 ms, see Figure 15d). Full analysis of all multiplets was achieved apart from those at 3.80–3.77 ppm (H6β and H5β) and the one at 3.74–3.71 ppm (H5α).

Furanose anomer assignment for FDAll-3 was initially based on the chemical shifts of the anomeric protons (5.41 and 5.31 ppm), as the ^3^*J*_H1–H2_ values of both furanose anomers were too close (4.7 and 4.4 Hz). The unusually elevated ^3^*J*_H1–H2_ = 4.4 Hz for β-*f*-FDAll-3 was consistent with the NMR assignment of 2-acetamido-2-deoxy-d-allose (AllNAc) furanoses, where both α-*f*-d-AllNAc and β-*f*-d-AllNAc showed ^3^*J*_H1–H2_ ≈ 4.85 Hz.^85^ The authors attributed this to a different degree of conformational averaging in the allo-configured furanoses compared to, e.g., galactofuranoses. The anomeric proton assignment was further confirmed by the chemical shifts of the anomeric carbons, which were obtained from a ^1^H–^13^C HSQC spectrum. The ^13^C NMR chemical shifts have been used previously for d-allose^86^ and d-AllNAc^85^ to distinguish between pyranoses and furanoses, with C4 resonating at ∼67 ppm for pyranoses vs at ∼85 ppm for furanoses. The C1 chemical shifts of the furanoses were also characteristic for the α- and β-tautomers (ca. 96 and 101 ppm, respectively). In the case of FDAll-3, C4 of the furanose tautomers indeed resonated at 82.8 and 80.8 ppm, with the anomers assigned as α- and β-furanoses having C1 chemical shifts of 95.1 and 100.2 ppm, respectively.

The furanose tautomer ^1^H spectral assignments for FDGal-3 have been reported by Blanchard et al.,^78^ including some of the coupling constants. For this fluorosugar, the anomeric protons of the furanose forms were only separated by 5 Hz (600 MHz spectrometer), often resulting in sel-TOCSY experiments from H1 leading to excitation of both species. Using FESTA, we have been able to expand and correct these assignments (Figure S22), arriving at a full chemical shift and *J*-coupling characterization (see Table S2).

## Conclusions

The large range of examples investigated in this work demonstrates that the recent SRI-FESTA experiment holds great promise for NMR analysis of reducing fluorinated carbohydrates and their derivatives. Despite the tautomeric species existing in an equilibrium, individual high-quality ^1^H NMR spectra become accessible, even for furanose forms if they are sufficiently populated for NMR observation. The conventional sel-TOCSY experiment has as the main limitation that it requires a cleanly resolved proton resonance in the spectrum of each anomer (typically the anomeric proton), which is not generally the case. This is especially true for furanose minor forms, which may overlap with impurity signals or ^13^C satellites of major compounds. If the fluorosugars are found in a more complex mixture with organic or biological compounds (e.g., molecular recognition studies) or form part of oligosaccharides then it is very likely that in sel-TOCSY no resolved proton signal can be identified. Thanks to the double ^1^H/^19^F selectivity of the SRI-FESTA method, clean ^1^H anomer subspectra will become achievable. Another advantage is that multiple protons at various positions in the fluorosugar can serve as a starting point for TOCSY mixing. We have shown that this ability is key in overcoming TOCSY mixing bottlenecks created by small ^3^*J*_HH_ coupling due to which sel-TOCSY experiments often fail to deliver the full anomer subspectrum.

Using SRI-FESTA, we achieved for the first time full chemical shift and *J*-coupling characterization for FDGlc-2, FDMan-2, FDGal-3, and FDAll-3 pyranoses. For FDGal-6, FDAll-3, and FDGal-3 furanoses, all chemical shifts and most of the *J* couplings were obtained. To the best of our knowledge, this is the first example of the utilization of FESTA for the analysis of a mixture of interconverting species. We envision that this technique will also be useful for the characterization of resonances from fluorosugar units that are part of larger glycans and for the characterization of other types of interconverting fluorinated species. While written with fluorinated carbohydrates in mind, the workflow should be equally applicable to any mixture of fluorinated organic compounds that interconvert slowly on the NMR frequency–time scale.

## Experimental Section

### NMR Assignments

Samples of 2-deoxy-2-fluoro-d-glucose (FDGlc-2, >98% pure), 3-deoxy-3-fluoro-d-glucose (FDGlc-3, >98% pure), 4-deoxy-4-fluoro-d-glucose (FDGlc-4, >98% pure), 6-deoxy-6-fluoro-d-glucose (FDGlc-6, >98% pure), 3-deoxy-3-fluoro-d-galactose (FDGal-3, >98% pure), 6-deoxy-6-fluoro-d-galactose (FDGal-6, >98% pure), 2-deoxy-2-fluoro-d-mannose (FDMan-2, >97% pure), and 3-deoxy-3-fluoro-d-allose (FDAll-3, purity not specified) were obtained from commercial suppliers and used as received. Compounds 4,6-dideoxy-4,6-difluoro-d-glucose (FDGlc-46)^11,79^ and 4,6-dideoxy-4,6-difluoro-d-galactose (FDGal-46)^79^ were prepared according to established procedures.

All NMR spectra were recorded on a Bruker Avance III HD 500 or 600 instrument. The SRI-FESTA and SRI experiments were acquired following the methodology published by Morris and co-workers.^60^ For NMR experimental details, please see the Supporting Information. In this section, assignment of ^1^H and ^19^F spectra is provided for each mixture component. The ^1^H and ^1^H{^19^F} NMR spectral assignments were performed using 2D COSY and 1D SRI-FESTA spectra. Data are reported as chemical shift (δ /ppm), number of protons, multiplicity, coupling constants (*J*) in Hertz (Hz), and attribution. ^1^H chemical shifts (δ) were quoted in ppm relative to D_2_O (4.79 ppm). ^19^F spectra were referenced to CFCl_3_. Signal multiplicities are described as singlet (s), doublet (d), triplet (t), quartet (q), multiplet (m), or a combination of those. Signals that are simplified because of fortuitously similar coupling constants are indicated as “apparent (app)”: a dd that simplifies to a t is indicated as app(t). Pyranose anomer ratios were obtained via integration of the ^1^H anomeric region; contents of furanose tautomers were determined via integration of the ^19^F or ^1^H spectra.

The tables below list the ^1^H and ^19^F resonances, their coupling constants, and their assignments based on the FESTA analysis.^87−89^ The values shown represent the splittings independently measured from the indicated multiplet. Small deviations for splittings found on different multiplets can be because of measurement error due to overlapping resonances or secondary order effects. Entries indicated in bold represent improvements to literature data. A summary of these improvements is given in Tables S2 and S3.

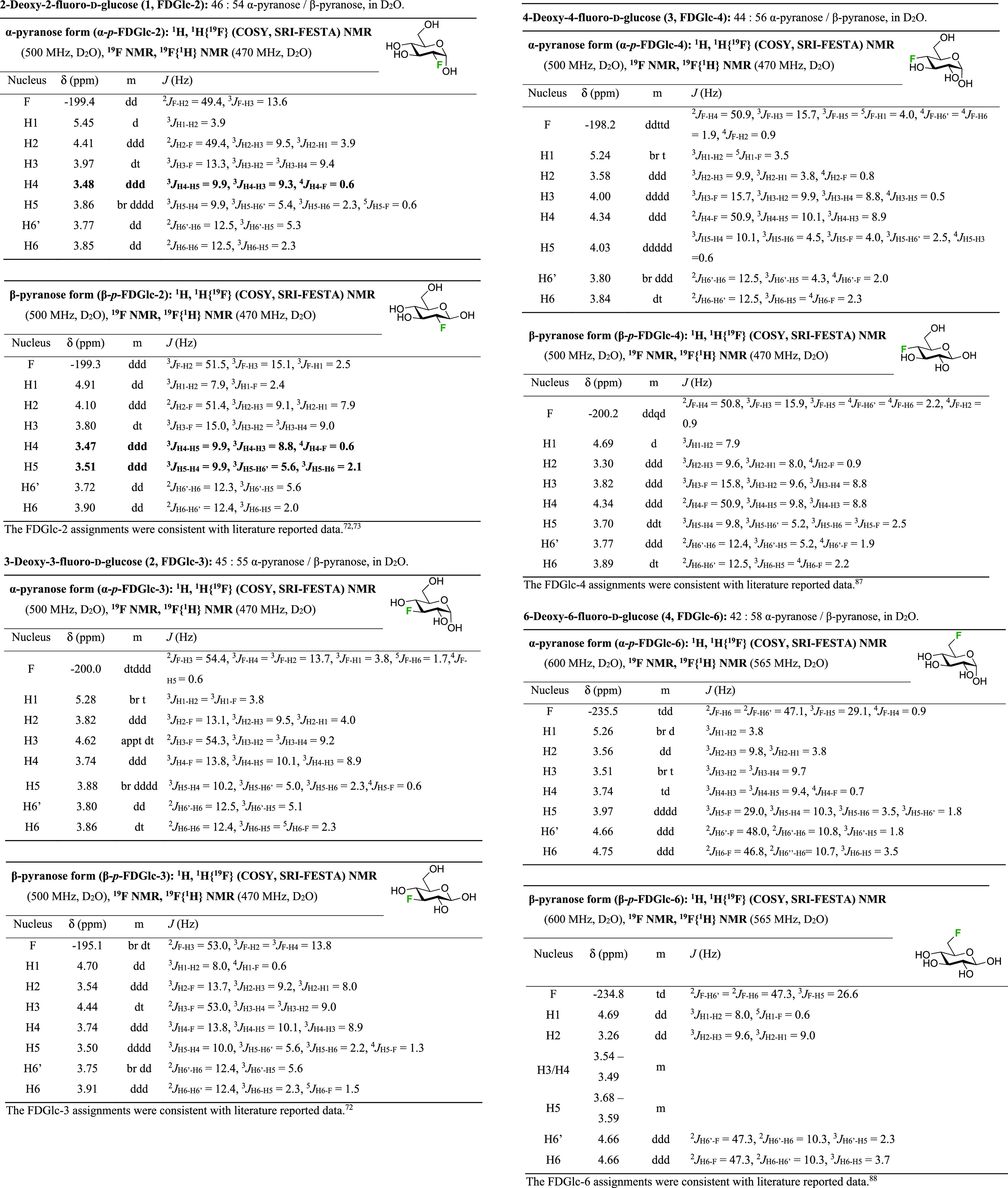

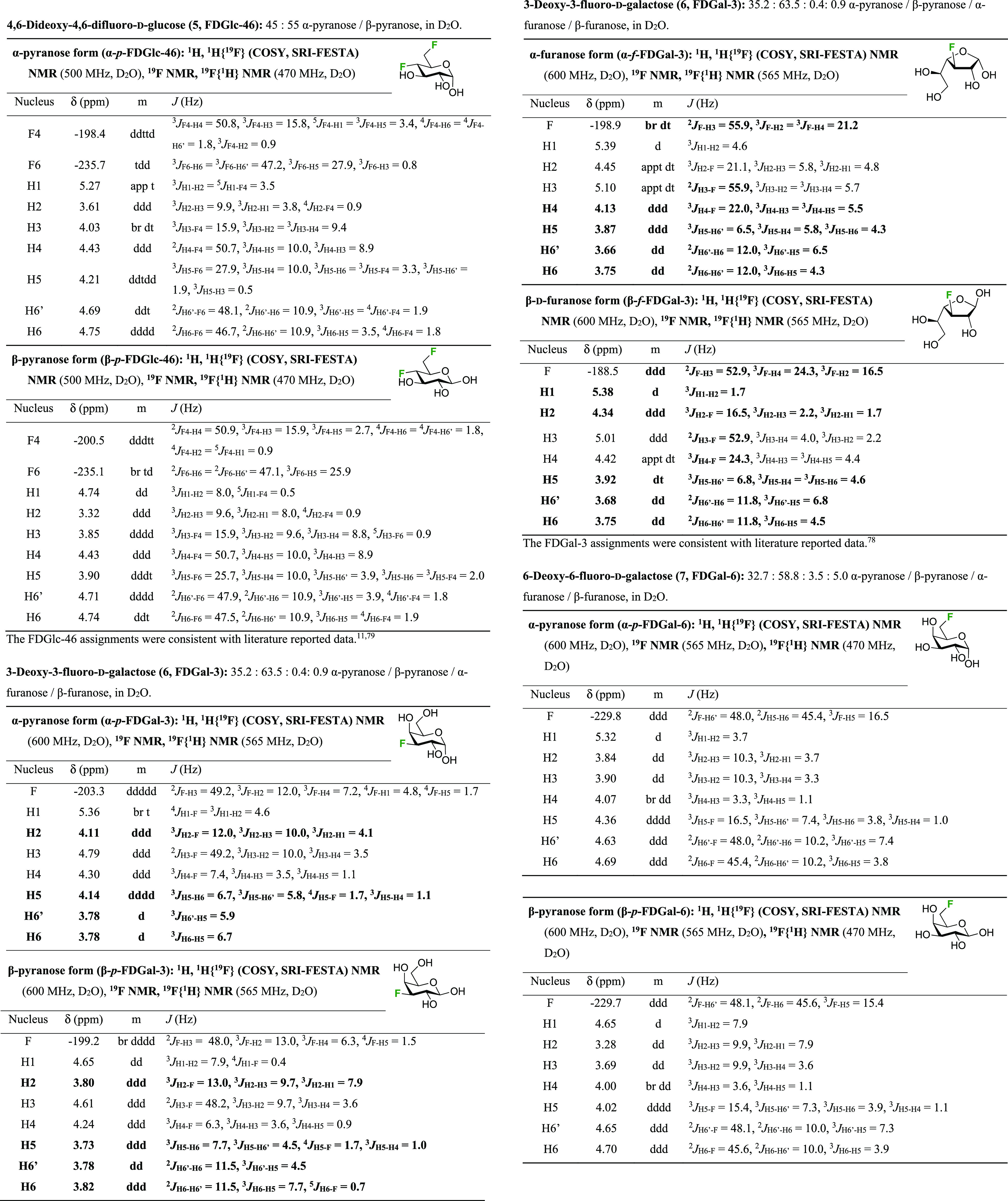

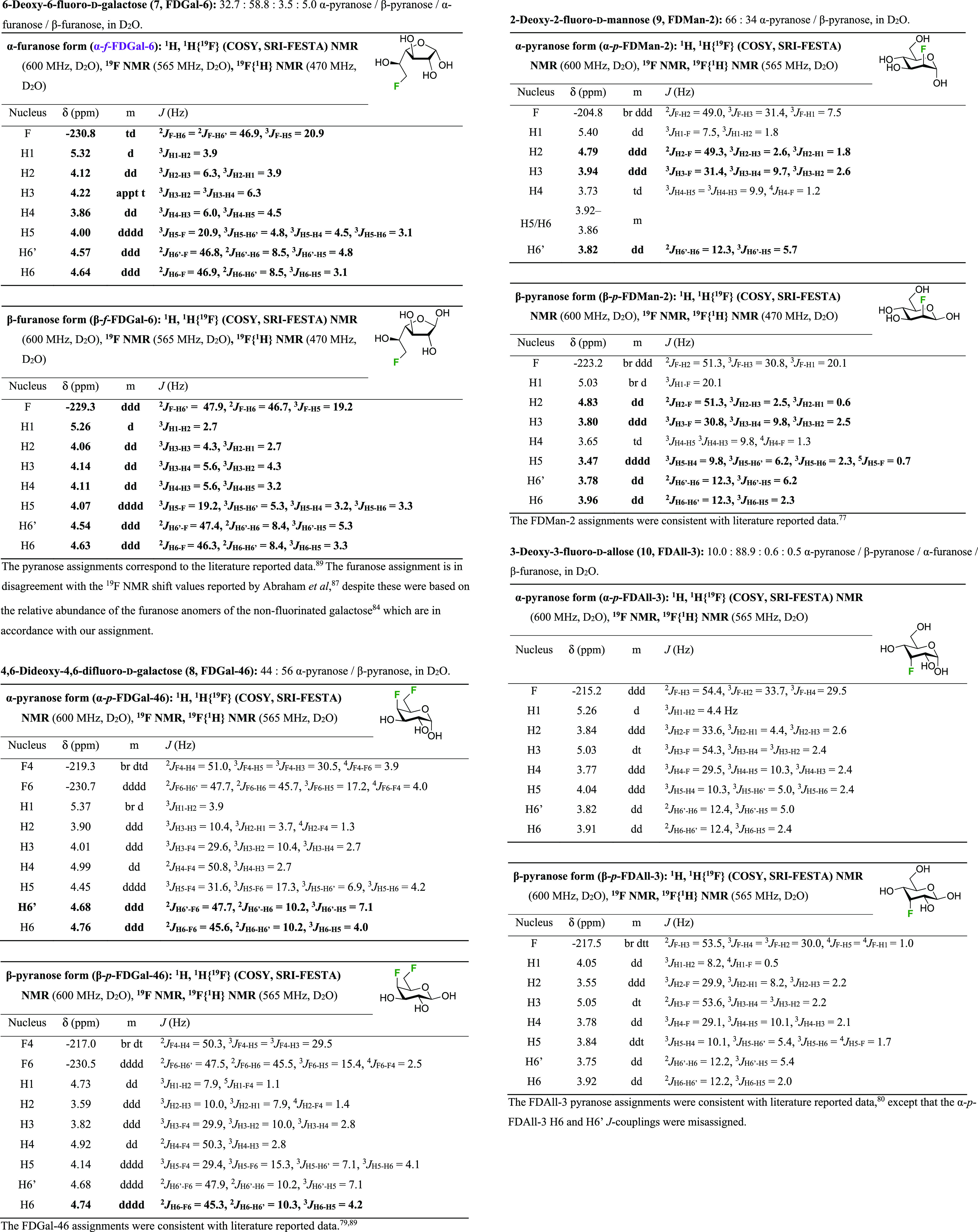

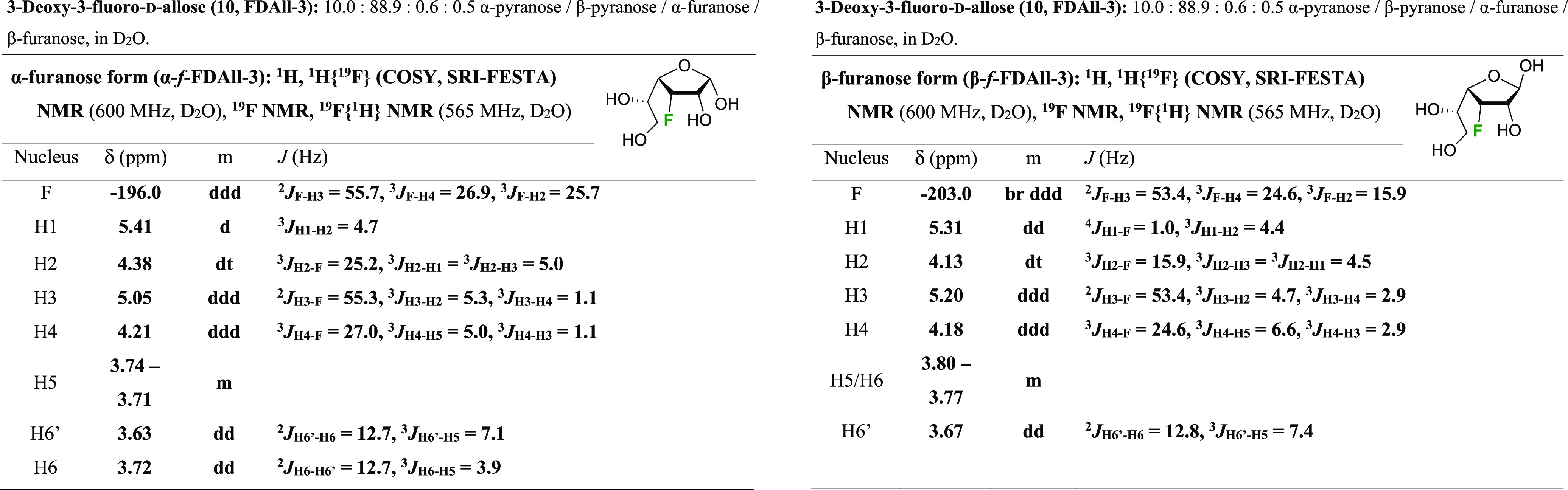









## Data Availability

The data underlying this study are available in the published article and its Supporting Information.

## Supporting Information Available

Recommended workflow on setting up the SRI-FESTA experiment, corrections, and additional multiplet characterization of fluorinated carbohydrates, full experimental details, additional figures and copies of spectra for all compounds (PDF)
FAIR data, including the primary NMR FID files, for compounds **1H–19F** (ZIP)
