# Supplementary material for: Obtaining Pure 1H NMR Spectra of Individual Pyranose and Furanose Anomers of Reducing Deoxyfluorinated Sugars
Source: J Org Chem. 2023 Sep 27;88(19):13908–25. doi: 10.1021/acs.joc.3c01503 (PMC10563139; doi:10.1021/acs.joc.3c01503)
Supplement: Supplementary file 1 — jo3c01503_si_001.pdf [file jo3c01503_si_001.pdf]

SUPPLEMENTARY INFORMATION

Obtaining pure ^{1}H NMR spectra of individual pyranose and furanose anomers of reducing deoxyfluorinated sugars

Gabija Poškaitė,[1] David Wheatley,[1] Neil Wells,[1] Bruno Linclau,[1,2]* and Davy Sinnaeve[3,4]*

bruno.linclau@ugent.be

davy.sinnaeve@univ-lille.fr

Contents

1 NMR experimental S8

1.1 General information S8

1.2 SRI-FESTA and SRI NMR experimental S8

1.3 Recommended workflow for SRI-FESTA experiments S9

1.4 Figure S1: ^{1}H-^{19}F HMQC NMR spectrum of FDGal-6. .... S11

1.5 Figure S2: Summary of possible resonance overlap scenario's S12

1.6 Table S1. ^{19}F resonance chemical shift difference of monosaccharides 1-10 pyranose forms at 500 MHz in D_2O. S12

2 Figures S3-S5: Exemplary SRI experiments (FDGlc-4, FDGlc-3 and FDGal-3) S13

3 Figures S6-S16: Selected delay experiments (FDGlc-2, FDGal-3, FDGal-6, FDAll-3) S15

4 Figures S17-S23: SRI-FESTA and sel-TOCSY experiments S22

4.1 Figure S17: ^{1}H-^{1}H{^{19}F} sel-TOCSY and ^{1}H{^{19}F} SRI-FESTA NMR subspectra of FDMan-2 (9) (F3 → H1 and F3 → H2) S22

4.2 Figure S18 (^{1}H-^{1}H{^{19}F} sel-TOCSY and ^{1}H{^{19}F} SRI-FESTA NMR subspectra of FDGal-46 (7) (F3 → H1 and F3 → H2) S23

4.3 Figure S19: ^{1}H{^{19}F} SRI-FESTA NMR subspectra of FDAll-3 (10) (F3 → H3) S24

4.4 Figure S20. ^{1}H{^{19}F} SRI-FESTA NMR subspectra of FDGlc-46 (5) (selection of either F4 and F6) S25

4.5 Figure S21: $^1H\{^{19}F\}$ SRI-FESTA NMR subspectra of β-*f*-FDAll-3 (3-deoxy-3-fluoro-β-D-allofuranose) (F3 → H3 and F3 → H2+H4) S26

4.6 Figure S22: $^1H\{^{19}F\}$ SRI-FESTA NMR subspectra of FDGal-3 furanoses (F3 → H3) S27

5 Tables S2 and S3: Corrections and additional multiplet characterization of fluorinated carbohydrates S28

5.1 Table S2. Detailed $^1H\{^{19}F\}$ spectra characterization of FDGal-3 furanoses in D_2O. S28

5.2 Table S3. More detailed $^1H\{^{19}F\}$ spectra characterization of pyranoses in D_2O at 300 K, 600 MHz. S28

6 Experimental details for figures in main manuscript S29

6.1 Table S4. Experimental details for Figure 3 .... S29

6.2 Table S5. Experimental details for Figure N6 .... S29

6.1 Table S6. Experimental details for Figure 7 .... S30

6.2 Table S7. Experimental details for Figure 8 .... S30

6.3 Table S8. Experimental details for Figures 9 and 11. .... S30

6.4 Table S9. Experimental details for Figure 10 .... S31

6.5 Table S10. Experimental details for Figure 12 .... S31

6.6 Table S11. Experimental details for Figure 13 .... S31

6.1 Table S12. Experimental details for Figure 14 .... S32

6.2 Table S13. Experimental details for Figure 15 .... S32

7 Copies of spectra of all compounds and anomers: S33

7.1 2-Deoxy-2-fluoro-D-glucose (1, FDGlc-2): 46 : 54 α-pyranose / β-pyranose, in D_2O. S33

7.1.1 FDGlc-2 (**1**): **^{1}H NMR** (500 MHz, D_2O) S33

7.1.2 FDGlc-2 (**1**): **$^1H\{^{19}F\}$ NMR** (500 MHz, D_2O) S34

7.1.3 FDGlc-2 (**1**): **$^{19}F\{^1H\}$ NMR** (470 MHz, D_2O) S35

7.1.4 FDGlc-2 (**1**): **^{19}F NMR** (470 MHz, D_2O) S35

7.1.5 FDGlc-2 (**1**): **^1H-^1H COSY** (500 MHz, D_2O) S36

7.1.6 FDGlc-2 (**1**): **^1H-$^1H\{^{19}F\}$ COSY** (500 MHz, D_2O) S36

7.1.7 FDGlc-2 (**1**): α-pyranose form (α-*p*-FDGlc-2): **^{1}H{^{19}F} SRI-FESTA NMR** (500 MHz, D_2O, δ^{19}F = -199.45 ppm, δ^1H = 4.41 ppm, τ_m = 120 ms) S37

7.1.8 FDGlc-2 (**1**): β-pyranose form (β-*p*-FDGlc-2): **^{1}H{^{19}F} SRI-FESTA NMR** (500 MHz, D_2O, δ^{19}F = -199.30 ppm, δ^1H = 4.10 ppm, τ_m = 120 ms) S38

7.2 3-Deoxy-3-fluoro-D-glucose (2, FDGlc-3): 45 : 55 α-pyranose / β-pyranose, in D_2O. S39

7.2.1 FDGlc-3 (**2**): **^{1}H NMR** (500 MHz, D_2O) S39

7.2.2 FDGlc-3 (**2**): **^{1}H{^{19}F} NMR** (500 MHz, D_2O) S40

7.2.3 FDGlc-3 (**2**): **^{19}F{^{1}H} NMR** (470 MHz, D_2O) S41

7.2.4 FDGlc-3 (**2**): **^{19}F NMR** (470 MHz, D_2O) S41

7.2.5 FDGlc-3 (**2**): **^{1}H-^{1}H COSY** (500 MHz, D_2O) S42

7.2.6 FDGlc-3 (**2**): α-pyranose form (α-*p*-FDGlc-3): **^{1}H{^{19}F} SRI-FESTA NMR** (500 MHz, D_2O, δ^{19}F = -200.01 ppm, δ^1H = 4.62 ppm, τ_m = 100 ms) S43

7.2.7 FDGlc-3 (**2**): β-pyranose form (β-*p*-FDGlc-3): **^{1}H{^{19}F} SRI-FESTA NMR** (500 MHz, D_2O, δ^{19}F = -195.11 ppm, δ^1H = 4.44 ppm, τ_m = 100 ms) S44

7.3 4-Deoxy-4-fluoro-D-glucose (3, FDGlc-4): 44 : 56 α-pyranose / β-pyranose, in D_2O. S45

7.3.1 FDGlc-4 (**3**): **^{1}H NMR** (500 MHz, D_2O) S45

7.3.2 FDGlc-4 (**3**): **^{1}H{^{19}F} NMR** (500 MHz, D_2O) S46

7.3.3 FDGlc-4 (**3**): **^{19}F{^{1}H} NMR** (470 MHz, D_2O) S47

7.3.4 FDGlc-4 (**3**): **^{19}F NMR** (470 MHz, D_2O) S47

7.3.5 FDGlc-4 (**3**): **^{1}H-^{1}H COSY** (500 MHz, D_2O) S48

7.3.6 FDGlc-4 (**3**): α-pyranose form (α-*p*-FDGlc-4): **^{1}H{^{19}F} SRI-FESTA NMR** (500 MHz, D_2O, δ^{19}F = -198.21 ppm, δ^1H = 5.24 ppm, τ_m = 120 ms) S49

7.3.7 FDGlc-4 (**3**): β-pyranose form (β-*p*-FDGlc-4): **^{1}H{^{19}F} SRI-FESTA NMR** (500 MHz, D_2O, δ^{19}F = -200.22 ppm, δ^1H = 3.30 ppm, τ_m = 80 ms) S50

7.4 6-Deoxy-6-fluoro-D-glucose (4, FDGlc-6): 42 : 58 α-pyranose / β-pyranose, in D_2O. S51

7.4.1 FDGlc-6 (**4**): **^{1}H NMR** (600 MHz, D_2O) S51

7.4.2 FDGlc-6 (**4**): **^{1}H{^{19}F} NMR** (600 MHz, D_2O) S52

7.4.3 FDGlc-6 (**4**): **^{19}F{^{1}H} NMR** (565 MHz, D_2O) S53

7.4.4 FDGlc-6 (**4**): **^{19}F NMR** (565 MHz, D_2O) S53

7.4.5 FDGlc-6 (**4**): **^{1}H-^{1}H{^{19}F} COSY** (600 MHz, D_2O) S54

7.4.6 FDGlc-6 (**4**): α-pyranose form (α-*p*-FDGlc-4): **^{1}H{^{19}F} SRI-FESTA NMR** (600 MHz, D_2O, δ^{19}F = -235.49 ppm, δ^{1}H = 4.75 ppm, τ_m = 200 ms) S55

7.4.7 FDGlc-6 (**4**): β-pyranose form (β-*p*-FDGlc-4): **^{1}H{^{19}F} SRI-FESTA NMR** (600 MHz, D_2O, δ^{19}F = -234.75 ppm, δ^{1}H = 3.66 ppm, τ_m = 200 ms) S56

7.5 4,6-Dideoxy-4,6-difluoro-D-glucose (5, FDGlc-46): 45 : 55 α-pyranose / β-pyranose, in D_2O. S57

7.5.1 FDGlc-46 (**5**): **^{1}H NMR** (500 MHz, D_2O) S57

7.5.2 FDGlc-46 (**5**): **^{1}H{^{19}F} NMR** (500 MHz, D_2O) S58

7.5.3 FDGlc-46 (**5**): **^{19}F NMR** (470 MHz, D_2O) S59

7.5.4 FDGlc-46 (**5**): **^{19}F{^{1}H} NMR** (470 MHz, D_2O) S59

7.5.5 FDGlc-46 (**5**): **^{1}H-^{1}H COSY** (500 MHz, D_2O) S60

7.5.6 FDGlc-46 (**5**): **^{1}H-^{1}H{^{19}F} COSY** (500 MHz, D_2O) S60

7.5.7 FDGlc-46 (**5**): α-pyranose form (α-*p*-FDGlc-46): **^{1}H{^{19}F} SRI-FESTA NMR** (500 MHz, D_2O, δ^{19}F = -198.43 ppm, δ^{1}H = 4.43 ppm, τ_m = 80 ms) S61

7.5.8 FDGlc-46 (**5**): β-pyranose form (β-*p*-FDGlc-46): **^{1}H{^{19}F} SRI-FESTA NMR** (500 MHz, D_2O, δ^{19}F = -200.50 ppm, δ^{1}H = 4.43 ppm, τ_m = 80 ms) S62

7.6 3-Deoxy-3-fluoro-D-galactose (6, FDGal-3): 35.2 : 63.5 : 0.4: 0.9 α-pyranose / β-pyranose / α-furanose / β-furanose, in D_2O. S63

7.6.1 FDGal-3 (**6**): **^{1}H NMR** (600 MHz, D_2O) S63

7.6.2 FDGal-3 (**6**): **^{1}H{^{19}F} NMR** (600 MHz, D_2O) S64

7.6.3 FDGal-3 (**6**): **^{19}F NMR** (565 MHz, D_2O) S65

7.6.4 FDGal-3 (**6**): **^{19}F{^{1}H} NMR** (565 MHz, D_2O) S66

7.6.5 FDGal-3 (**6**): **^{1}H-^{1}H{^{19}F} COSY** (600 MHz, D_2O) S66

7.6.6 FDGal-3 (**6**): α-pyranose form (α-*p*-FDGal-3): **^{1}H{^{19}F} SRI-FESTA NMR** (600 MHz, D_2O, δ^{19}F = -203.29 ppm, δ^{1}H = 4.17-4.06 ppm, τ_m = 300 ms) S67

7.6.7 FDGal-3 (**6**): β-pyranose form (β-*p*-FDGal-3): **^{1}H{^{19}F} SRI-FESTA NMR** (600 MHz, D_2O, δ^{19}F = -199.18 ppm, τ_m = 300 ms, a) δ^1H = 4.24 ppm; b) δ^1H = 3.73 ppm)........................ S68

7.6.8 FDGal-3 (**6**): α-furanose form (α-*f*-FDGal-3): **^{1}H{^{19}F} SRI-FESTA NMR** (600 MHz, D_2O, δ^{19}F = -198.88 ppm, δ^1H = 4.45 ppm, τ_m = 200 ms, 4096 ns) S70

7.6.9 FDGal-3 (**6**): β-furanose form (β-*f*-FDGal-3): **^{1}H{^{19}F} SRI-FESTA NMR** (600 MHz, D_2O, δ^{19}F = -188.55 ppm, δ^1H = 4.42 ppm, τ_m = 300 ms, 4096 ns) S71

7.7 6-Deoxy-6-fluoro-D-galactose (7, FDGal-6): 32.7 : 58.8 : 3.5 : 5.0 α-pyranose / β-pyranose / α-furanose / β-furanose, in D_2O. S72

7.7.1 FDGal-6 (**7**): **^{1}H NMR** (600 MHz, D_2O).. S72

7.7.2 FDGal-6 (**7**): **^{1}H{^{19}F} NMR** (600 MHz, D_2O) S73

7.7.3 FDGal-6 (**7**): **^{19}F NMR** (565 MHz, D_2O).. S74

7.7.4 FDGal-6 (**7**): **^{19}F{^{1}H} NMR** (470 MHz, D_2O) S75

7.7.5 FDGal-6 (**7**): **^{1}H-^{1}H{^{19}F} COSY** (600 MHz, D_2O)... S75

7.7.6 FDGal-6 (**7**): α-pyranose form (α-*p*-FDGal-6): **^{1}H{^{19}F} SRI-FESTA NMR** (600 MHz, D_2O, δ^{19}F = -229.79 ppm, δ^1H = 4.36 ppm, τ_m = 300 ms)... S76

7.7.7 FDGal-6 (**7**): β-pyranose form (β-*p*-FDGal-6): **^{1}H{^{19}F} SRI-FESTA NMR** (600 MHz, D_2O, δ^{19}F = -229.65 ppm, δ^1H = 4.02 ppm, τ_m = 300 ms)... S77

7.7.8 FDGal-6 (**7**): α-furanose form (α-*f*-FDGal-6): **^{1}H{^{19}F} SRI-FESTA NMR** (600 MHz, D_2O, δ^{19}F = -230.79 ppm, δ^1H = 4.00 ppm, τ_m = 100 ms)... S78

7.7.9 FDGal-6 (**7**): β-furanose form (β-*f*-FDGal-6): **^{1}H{^{19}F} SRI-FESTA NMR** (600 MHz, D_2O, δ^{19}F = -229.26 ppm, δ^1H = 4.07 ppm, τ_m = 200 ms)... S79

7.8 4,6-Dideoxy-4,6-difluoro-D-galactose (8, FDGal-46): 44 : 56 α-pyranose / β-pyranose, in D_2O. 80

7.8.1 FDGal-46 (**8**): **^{1}H NMR** (600 MHz, D_2O)... S80

7.8.2 FDGal-46 (**8**): **^{1}H{^{19}F} NMR** (600 MHz, D_2O) S81

7.8.3 FDGal-46 (**8**): **^{19}F NMR** (565 MHz, D_2O)... S82

7.8.4 FDGal-46 (**8**): **^{19}F{^{1}H} NMR** (565 MHz, D_2O) S82

7.8.5 FDGal-46 (**8**): **^{1}H-^{1}H COSY** (500 MHz, D_2O) S83

7.8.6 FDGal-46 (**8**): **^{1}H-^{1}H{^{19}F} COSY** (500 MHz, D_2O) S83

7.8.7 FDGal-46 (**8**): α-pyranose form (α-*p*-FDGal-46): **^{1}H{^{19}F} SRI-FESTA NMR** (600 MHz, D_2O, a) δ^{19}F = -219.27 ppm, δ^1H = 4.99 ppm, τ_m = 100 ms; b) δ^{19}F = -230.48 ppm, δ^1H = 4.45 ppm, τ_m = 100 ms) S84

7.8.8 FDGal-46 (**8**): β-pyranose form (β-*p*-FDGal-46): **^{1}H{^{19}F} SRI-FESTA NMR** (600 MHz, D_2O, a) δ^{19}F = -216.93 ppm, δ^1H = 3.82 ppm, τ_m = 100 ms; b) δ^{19}F = -230.48 ppm, δ^1H = 4.14 ppm, τ_m = 100 ms) S85

7.9 2-Deoxy-2-fluoro-D-mannose (9, FDMan-2): 66 : 34 α-pyranose / β-pyranose, in D_2O. S87

7.9.1 FDMan-2 (**9**): **^{1}H NMR** (600 MHz, D_2O) S87

7.9.2 FDMan-2 (**9**): **^{1}H{^{19}F} NMR** (600 MHz, D_2O) S88

7.9.3 FDMan-2 (**9**): **^{19}F NMR** (565 MHz, D_2O) S89

7.9.4 FDMan-2 (**9**): **^{19}F{^{1}H} NMR** (565 MHz, D_2O) S89

7.9.5 FDMan-2 (**9**): **^{1}H-^{1}H COSY** (500 MHz, D_2O) S90

7.9.6 FDMan-2 (**9**): α-pyranose form (α-*p*-FDGMan-2): **^{1}H{^{19}F} SRI-FESTA NMR** (600 MHz, D_2O, δ^{19}F = -204.75 ppm, δ^1H = 3.94 ppm, τ_m = 300 ms) S90

7.9.7 FDMan-2 (**9**): β-pyranose form (β-*p*-FDMan-2): **^{1}H{^{19}F} SRI-FESTA NMR** (600 MHz, D_2O, δ^{19}F = -223.15 ppm, δ^1H = 4.83, τ_m = 200 ms) S92

7.10 3-Deoxy-3-fluoro-D-allose (10, FDAll-3): 10.0 : 88.9 : 0.6 : 0.5 α-pyranose / β-pyranose / α-furanose / β-furanose, in D_2O. S93

7.10.1 FDAll-3 (**10**): **^{1}H NMR** (600 MHz, D_2O) S93

7.10.2 FDAll-3 (**10**): **^{1}H{^{19}F} NMR** (600 MHz, D_2O) S94

7.10.3 FDAll-3 (**10**): **^{19}F NMR** (565 MHz, D_2O) S95

7.10.4 FDAll-3 (**10**): **^{19}F{^{1}H} NMR** (565 MHz, D_2O) S96

7.10.5 FDAll-3 (**10**): **^{1}H-^{1}H{^{19}F} COSY** (600 MHz, D_2O) S96

7.10.6 FDAll-3 (**10**): **^{1}H-^{13}C HSQC** (600 MHz, D_2O) S97

7.10.7 FDAll-3 (**10**): α-pyranose form (α-*p*-FDAll-3): **^{1}H{^{19}F} SRI-FESTA NMR** (600 MHz, D_2O, δ^{19}F = -215.18 ppm, δ^1H = 5.03 ppm, τ_m = 100 ms) S98

7.10.8 FDAll-3 (**10**): β-pyranose form (β-*p*-FDAll-3): **^{1}H{^{19}F} SRI-FESTA NMR** (600 MHz, D_2O, δ^{19}F = -217.46 ppm, δ^1H = 5.05 ppm, τ_m = 200 ms) S99

7.10.9 FDAll-3 (**10**): α-furanose form (α-*f*-FDAll-3): **^{1}H{^{19}F} SRI-FESTA NMR** (600 MHz, D_2O, δ^{19}F = -195.98 ppm, ZQS = 20 ms, a) δ^1H = 5.05 ppm, τ_m = 200 ms; b) δ^1H = 4.38 ppm, τ_m = 200 ms) S100

7.10.10 FDAll-3 (**10**): β-furanose form (β-*f*-FDAll-3): **^{1}H{^{19}F} SRI-FESTA NMR** (600 MHz, D_2O, δ^{19}F = -202.98 ppm, δ^1H = 3.89-4.59 ppm, τ_m = 100 ms, ZQS = 20 ms) S102

8 References S103

1 NMR experimental

1.1 General information

The samples were left to equilibrate for at least 3 days before the NMR acquisition. ^{1}H and ^{19}F NMR and COSY 2D NMR were acquired in addition to SRI-FESTA and selective ^{1}H-$^{1}H\{^{19}F\}$ TOCSY. Spectra were recorded on either a Bruker Avance III HD NMR spectrometer operating at 500.13 and 470.50 MHz ^{1}H and ^{19}F frequencies respectively equipped with a 5mm SMART probe, or a Bruker Avance III HD NMR spectrometer operating at 600.13 and 564.69 MHz ^{1}H and ^{19}F frequencies respectively equipped with a 5mm ^{1}H-^{13}C-^{15}N-^{19}F QCI cryoprobe. Sample temperature was set to 300 K throughout. ^{1}H chemical shifts (δ) were quoted in ppm relative to D_2O (4.79 ppm) if not otherwise stated. ^{19}F spectra were externally referenced to $CFCl_3$. TopSpin 4.0.7 was used for processing spectral data. Unless stated otherwise, ^{1}H and $^{1}H\{^{19}F\}$ were generally acquired with *ns* = 16 and *ds* = 4, and SRI-FESTA and sel-TOCSY experiments were acquired with *ns* = 32 and *ds* = 4, where *ns* is 'number of scans' and *ds* is 'dummy scans'.

1.2 SRI-FESTA and SRI NMR experimental

The SRI-FESTA and SRI experiments were acquired using the pulse sequence published by Castañar and co-workers.[1] Typically, ^{19}F-selective 90° pulses used either Gaussian[r2]or ESnob[3] shapes, while ^{1}H- and ^{19}F-selective 180° pulses used RSnob[3] or ReBurp[4] shapes. TOCSY transfer was achieved using the DIPSI-2 mixing scheme[5] with mixing times varying between 60 and 300 ms. At 600 MHz, the TOCSY spinlock pulses were applied with a power level corresponding to a 35 µs 90° pulse, while for the spectra recorded at 500 MHz this was a power level corresponding to a 30 µs 90° pulse. The two zero-quantum suppression (ZQS) elements[6] flanking the mixing scheme used Chirp pulses with a total 20% smoothening at the start and end of the pulse, sweeping 20 kHz bandwidth over a duration of typically 35 and 50 ms, or twice 10 ms in case of significant exchange between anomers (see Figure S10). Gradients applied during these pulses were at 1.6 G/cm. All coherence transfer pathway (CTP) gradient pulses had a duration of 1 ms, used a smoothed square shape, and had amplitudes as recommended by Castañar *et al.*[1] All gradient pulses were followed by a recovery delay of 200 µs. The SRI and SME transfer delays (Δ_1 and Δ_2 as defined in Figure 1 of Castañar *et al.*[1]) were set to $1/(4J_{HF}n_{\mathrm{H}})$, and $1/(4J_{HF}n_{\mathrm{F}})$, respectively, where J_{HF} is the heteronuclear coupling constant, n_{H} is the number of selected protons coupled to the selected fluorine, and n_{F} is the number of equivalent fluorines coupled to the selected proton. The required J_{HF} coupling constants could be obtained from ^{1}H and ^{19}F 1D NMR spectra. For more complex spectra with substantial overlap, an SRI experiment may firstly be acquired

(see Figure S1a) to determine which protons are coupled to the selected ^{19}F. In cases where the delays turned out shorter than the duration of the ^{1}H and/or ^{19}F selective 180° pulses, they were increased by a factor of 3 (see below, section 1.3, point 5), or were stepwise optimized for maximal signal output using standard procedures in Topspin (the 'POPT' command) in an SRI-FESTA experiment with zero TOCSY mixing time. An interscan relaxation delay of 1 s was used for the spectra recorded at 600 MHz and 2 s for those at 500 MHz. For ^{19}F decoupling, either a waltz16 or a bilevel adiabatic decoupling scheme were used. At 500 MHz, conventional ^{1}H NMR spectra and selective TOCSY were recorded with 15 kHz spectral width and 131072 complex points, ^{1}H{^{19}F} and SRI FESTA were recorded with 7 kHz spectral width and 65536 complex points, while SRI-FESTA was recorded with 7 kHz spectral width and 32768 complex points. At 600 MHz, all these spectra were typically recorded with a 9615.385 Hz spectral window and 32768 time domain points. All spectra were zero filled so that either up to 32768 or 65536 real data points were obtained in the spectrum.

1.3 Recommended workflow for SRI-FESTA experiments

We used the following workflow for SRI-FESTA experiments. The SRI and SRI-FESTA Bruker pulse sequences are available in the supporting information of the original paper of Castañar *et al.*[1] and on these author's website.

1) Calibrate both the ^{1}H and ^{19}F hard pulse power level, in order to properly calibrate the power levels of the TOCSY spinlock and ^{1}H and ^{19}F selective pulses. Acquire ^{1}H, ^{1}H{^{19}F}, ^{19}F, and ^{19}F{^{1}H} spectra, as well as standard 2D NMR spectra to aid in spectral assignment (^{1}H-^{1}H COSY, ^{1}H-^{13}C HSQC).
2) Acquire ^{1}H SRI (optional) to identify the protons coupled with a selected ^{19}F and to estimate the $^{n}J_{HF}$ couplings (see examples in Figures S14-S16). To identify the ^{1}H coupling partners, an alternative is to run a standard ^{1}H-^{19}F 2D HMQC experiment, which requires no ^{19}F selective pulse and can thus be useful in case of overlapping fluorine multiplets. It thus also reveals information for fluorine signals of all the mixture's components (furanoses and pyranoses) at once. For an example, see the 2D ^{1}H-^{19}F HMQC NMR spectrum of FDGal-6 (in Figure S1).
3) Identify a suitable ^{1}H coupling partner of the ^{19}F nucleus of the anomer of interest. As explained in the main manuscript, a number of factors determine the choice of an initial candidate proton, though none of them are crucial. Firstly, a sufficiently large J_{HF} coupling constant is preferred, resulting in short SRI-SME delays and thus lower T_2 relaxation losses. Geminal $^{2}J_{HF}$ and vicinal $^{3}J_{HF}$ couplings are the most obvious candidates. Preferably, but not crucially, an individual

proton is chosen that can be excited by the selective pulse in such a way that no other protons coupled to the same fluorine are excited. This to avoid signal losses from multiple J_{HF} couplings evolving at once. Equally, the proton selective pulse preferably (but again, not crucially) should not also select homonuclear coupling partners of the chosen proton, to avoid signal loss from unwanted J_{HH} coupling evolution during the SME element.

4) Set up the ^{19}F and ^{1}H selective pulses (pulse shapes, offsets, pulse durations as a function of bandwidths, power levels). Note that for the ^{1}H and ^{19}F selective pulses in FESTA, respectively the full ^{1}H and ^{19}F multiplet should be taken into account, *i.e.*, the multiplets in absence of heteronuclear decoupling. In case of ^{19}F multiplet overlap, either a ^{1}H coupling partner can be selected that does not overlap with a ^{1}H multiplet of the same anomer (see Figure S2), or the ^{19}F selective pulses can be given somewhat off-resonance to only cover the resonance of the intended ^{19}F multiplet.
5) When only one ^{1}H-^{19}F coupling evolves and no ^{1}H-^{1}H couplings evolve during the SRI and SME sequences (see above), the theoretical optimal transfer delays (Δ_1 and Δ_2 in the originally published sequence)[1] correspond to $1/(4J_{HF})$, neglecting T_2 relaxation. If these delays turn out too short to fit in the ^{1}H and or ^{19}F selective pulses (*i.e.*, very selective pulses have to be used in combination with large J_{HF} couplings), then longer delays can be chosen that in theory provide optimal transfer. These correspond to $(1+2n)/(4J_{HF})$, with n = 1,2,3… In cases where T_2 relaxation is significant (*i.e.*, in case of small J_{HF} couplings), or the evolution of multiple ^{1}H-^{19}F couplings or ^{1}H-^{1}H couplings during the SRI and SME sequences take place (*i.e.*, multiple protons coupled to fluorine or multiple protons coupled to each other are selected by the ^{1}H selective pulse), the optimal transfer delays are more difficult to estimate beforehand. Both T_2 relaxation and the evolution of other couplings will decrease the optimal transfer delays compared to the $1/(4J_{HF})$ value. For optimal sensitivity, these delays can then be optimized using an SRI-FESTA experiment with a 0 ms TOCSY mixing time. For this, the POPT function in Topspin can be used to do this in a stepwise fashion.
6) Acquire ^{1}H{^{19}F} SRI-FESTA. An initial TOCSY mixing time of 100-200 ms is recommended. Given the lower chemical shift dispersion of fluorosugar protons, a lower spinlock power level can be used if need be, in order to avoid sample heating, for instance a power level corresponding to a 35 μs hard pulse. In case small $^{3}J_{HH}$ couplings impede TOCSY transfer to the whole spin system, either longer mixing times can be used (*i.e.,* 300 ms), or another ^{1}H coupling partner of the fluorine can be chosen. For components interconverting at the NMR timescale (*i.e.,* furanoses) reduced duration of ZQS pulses (20 ms in total) are recommended.

1.4 Figure S1: ^{1}H-^{19}F HMQC NMR spectrum of FDGal-6.

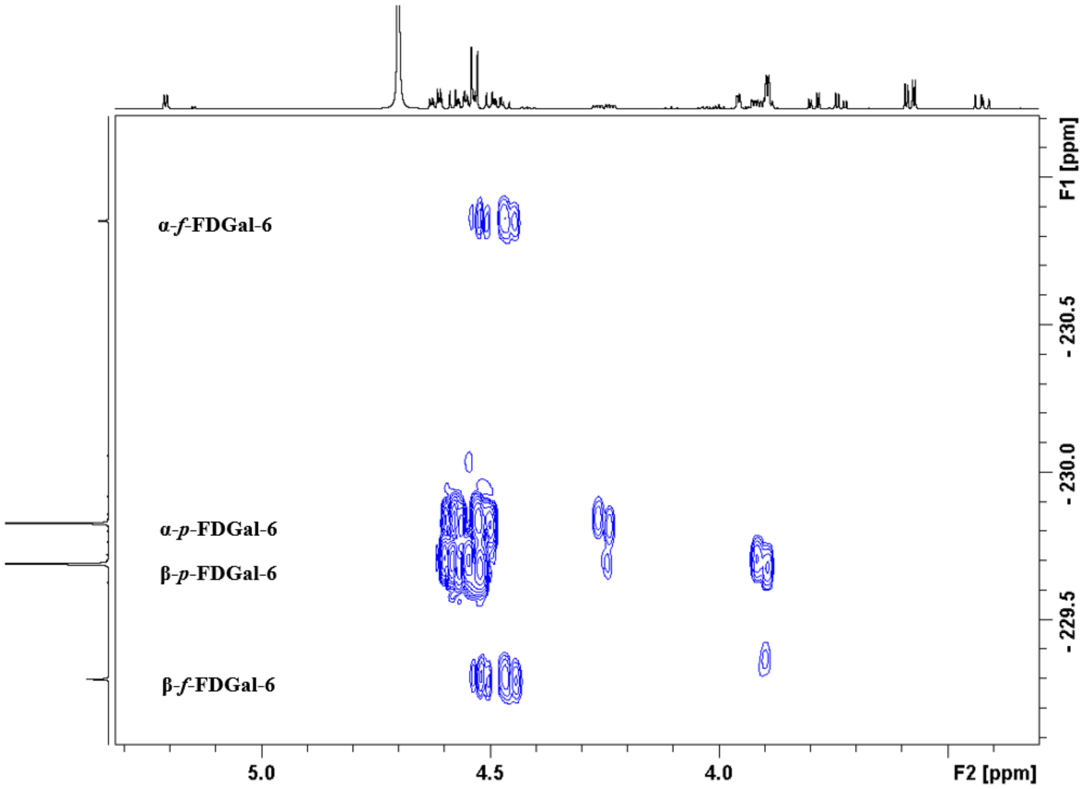

.

1.5 Figure S2: Summary of possible resonance overlap scenario's

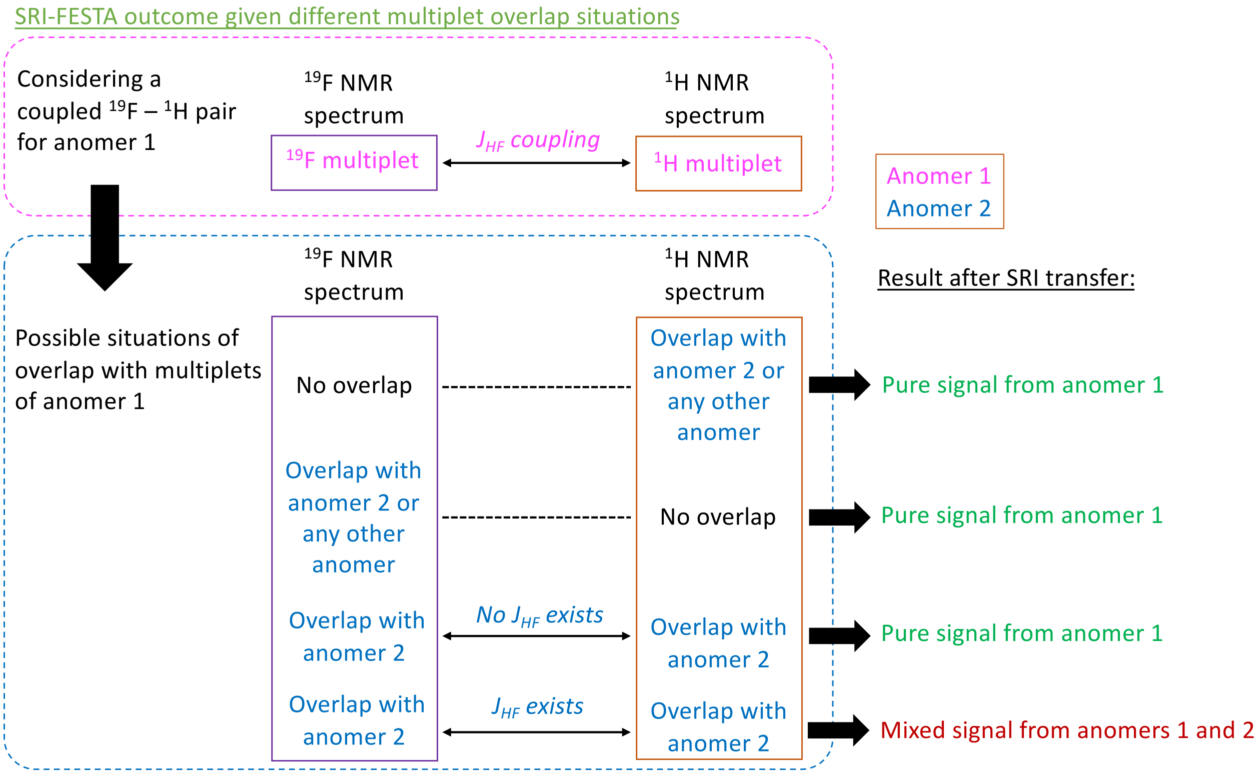

1.6 Table S1. ^{19}F resonance chemical shift difference of monosaccharides 1-10 pyranose forms at 500 MHz in D_2O.

Sugar	**1**	**2**	**3**	**4**	**5** (F4)	**5** (F6)	**6**	**7**	**8** (F4)	**8** (F6)	**9**	**10**
Δδ ^{19}F / ppm	0.1	4.90	2.01	0.74	2.07	0.61	4.10	0.14	2.34	0.23	18.40	2.29[a]
Δδ ^{19}F / Hz	67	2305	946	348	974	287	1929	66	1101	108	8657	1295[a]
Multiplet width (α, β) / Hz	69 63	88, 84	79, 74	125, 121	78, 74	120, 123	75, 69	110, 110	118, 113	114, 111	90, 114	117[a], 116[a]
Separation of multiplets / Hz	1	2219	870	225	902	166	1857	-44	987	-3	8539	1170[a]
Overlap	Y[b]	N	N	N	N	N	N	Y	N	Y	N	N

[a] Value at 600 MHz; [b] Borderline overlap at 500 MHz.

2 Figures S3-S5: Exemplary SRI experiments (FDGlc-4, FDGlc-3 and FDGal-3)

SRI experiments were routinely performed for all the samples (**1-10**) investigated for all of the ^{19}F resonances. Relevant to the manuscript SRI spectra of FDGlc-4, FDGlc-3 and FDGal-3 are depicted below (Figures S3-S5 respectively).

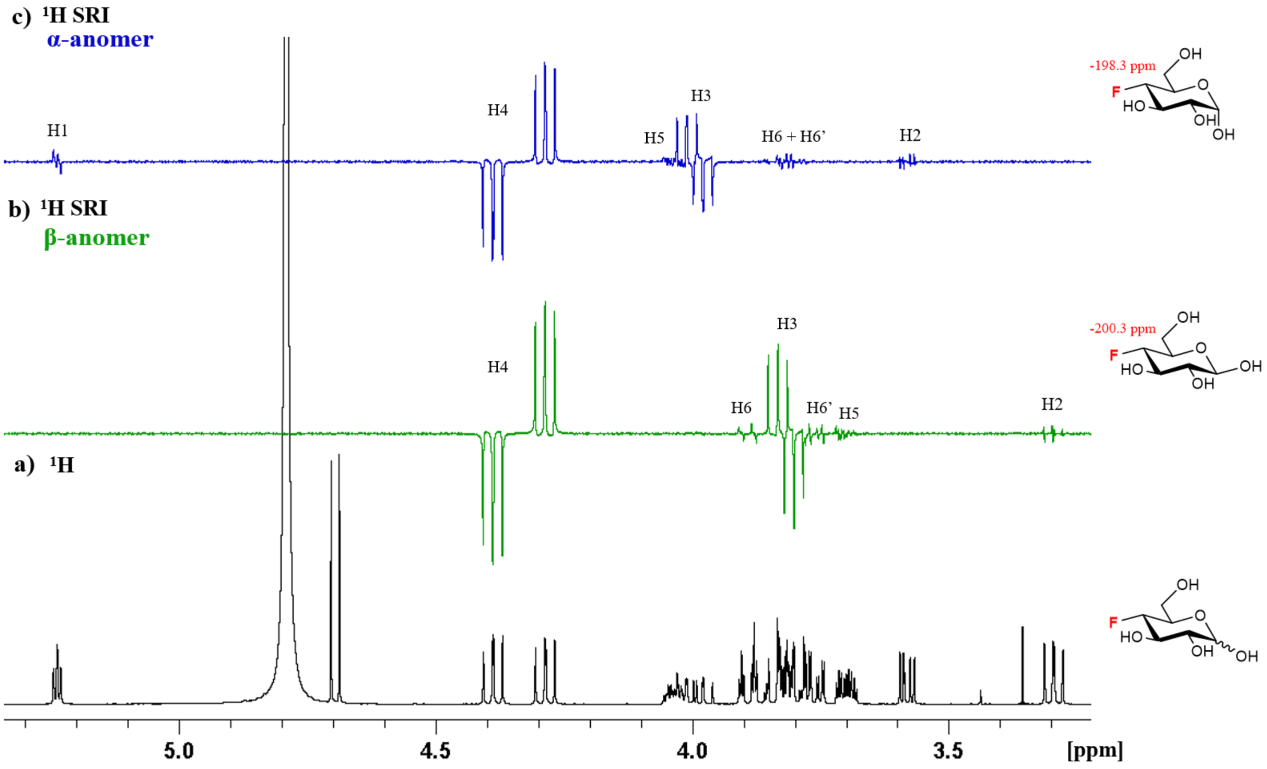

Figure S3. ^{1}H SRI NMR spectra of FDGlc-4 in D_2O, 500 MHz. a) ^{1}H NMR spectrum; b) ^{1}H SRI NMR spectrum of the β-anomer (Δ_1 = 4.90 ms, ns = 64, ds = 4); c) ^{1}H SRI NMR spectrum of the α-anomer (Δ_1 = 4.90 ms, ns = 64, ds = 4).

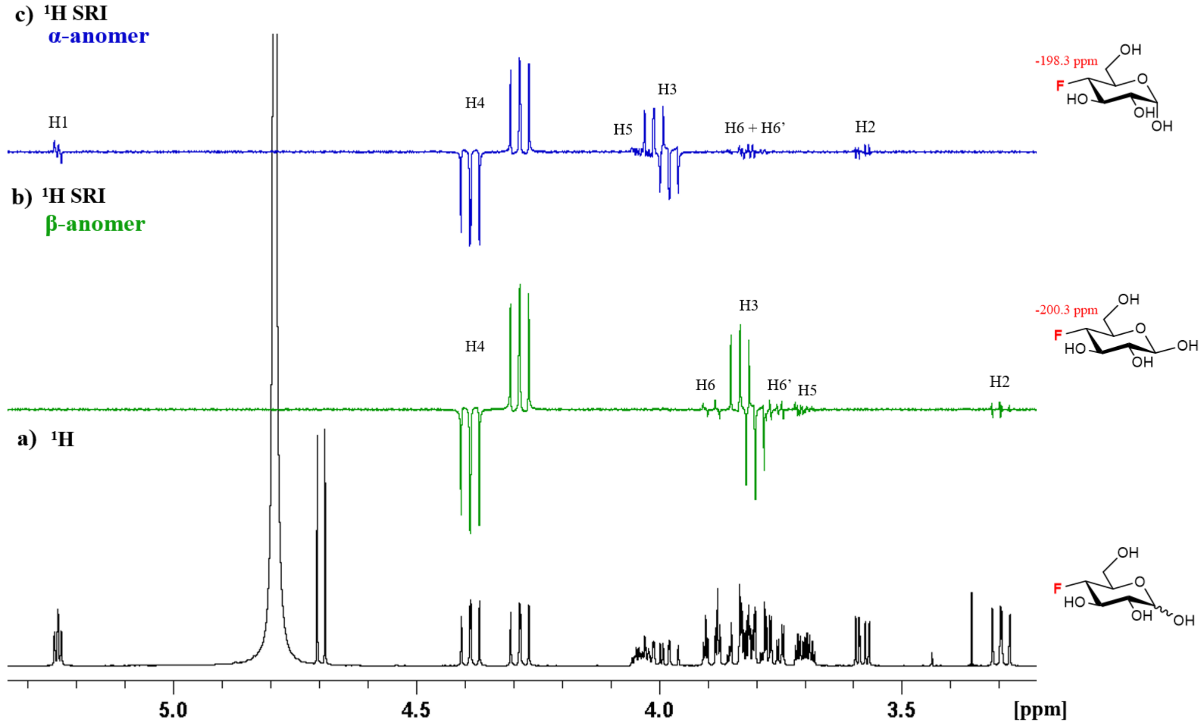

Figure S4. ^{1}H SRI NMR spectra of FDGlc-3 in D_2O, 500 MHz. a) ^{1}H NMR spectrum; b) ^{1}H SRI NMR spectrum of the β-anomer (Δ_1 = 4.72 ms, ns = 64, ds = 4); c) ^{1}H SRI NMR spectrum of the α-anomer (Δ_1 = 4.72 ms, ns = 64, ds = 4).

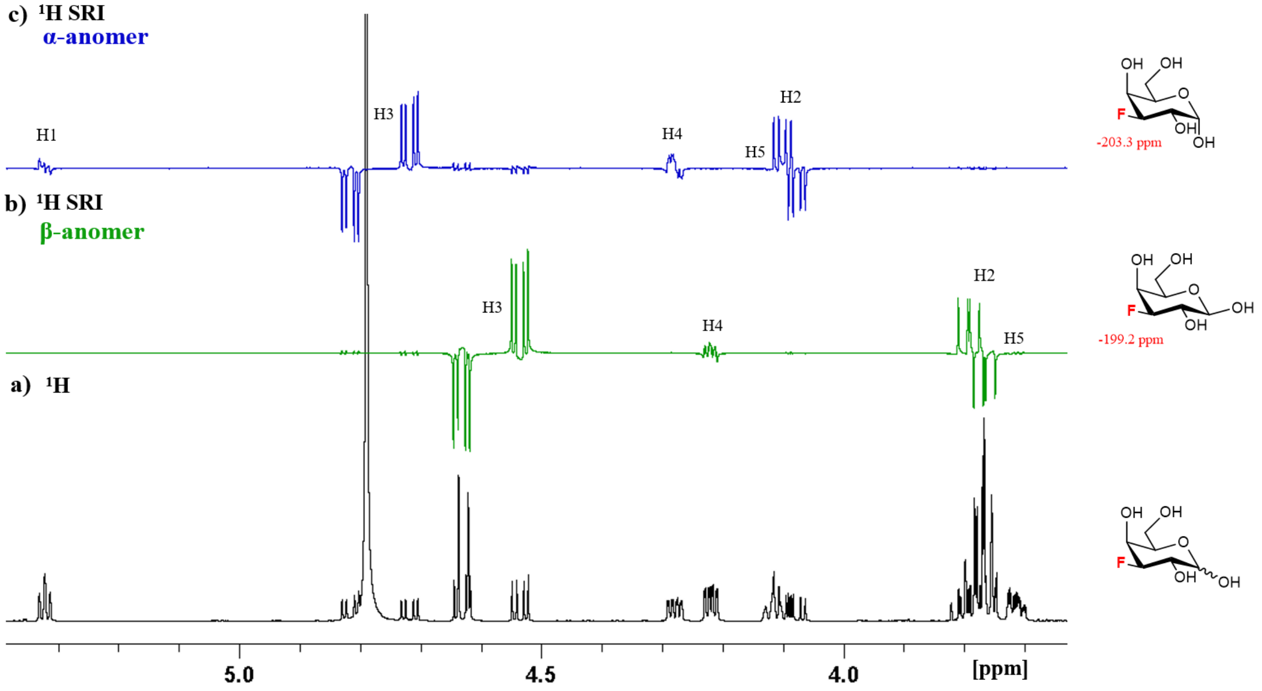

Figure S5. ^{1}H SRI NMR spectra of FDGal-3 in D_2O, 500 MHz. a) ^{1}H NMR spectrum; b) ^{1}H SRI NMR spectrum of the β-anomer (Δ_1 = 5.10 ms, ns = 64, ds = 4); c) ^{1}H SRI NMR spectrum of the α-anomer (Δ_1 = 5.10 ms, ns = 64, ds = 4).

3 Figures S6-S16: Selected delay experiments (FDGlc-2, FDGal-3, FDGal-6, FDAll-3)

SRI-FESTA spinlock times were screened in the range of 60 to 200 ms in 20 ms increments for pyranose components of samples **1-8** and in a range of 0-300 ms in 100 ms increments for samples **9-10** and furanoses investigated. See FID files supplied. Below exemplary delay screening spectra for α-*p*-FDGlc-2, β-*p*-FDGlc-2, α-*p*-FDGal-3, β-*p*-FDGal-3, α-*p*-FDGal-6, β-*p*-FDGal-6, α-*f*-FDGal-6 and β-*f*-FDGal-6 are provided (Figures S6-S13 respectively).

As discussed in the 'Optimizing the $^1H \rightarrow {}^1H$ TOCSY magnetization transfer' section of the main manuscript, the FDAll-3 furanose anomers were interconverting even at $\tau_m = 0$ ms. We proposed a strategy to reduce the duration of the ZQS pulses from 85 ms to 20 ms; here Figure S14 shows the SRI-FESTA spectra of β-*f*-FDAll-3 (F3→H3) with $\tau_m = 0$ ms with a total ZQS pulse duration of 85 ms and 20 ms. Also, the delay screenings with reduced ZQS pulses to 20 ms are provided for α-*f*-FDAll-3 and β-*f*-FDAll-3 (Figures S15-16 respectively).

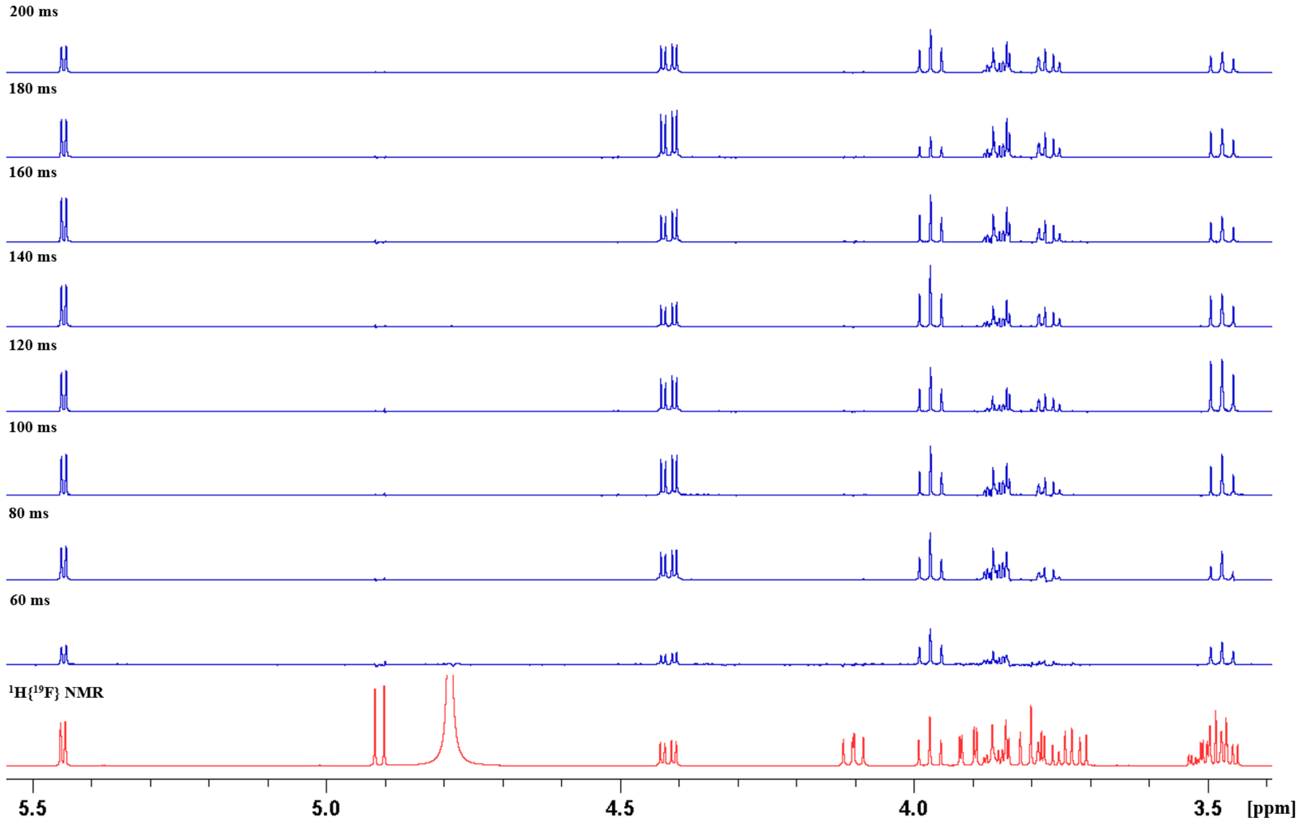

Figure S6. $^1H\{^{19}F\}$ SRI-FESTA NMR (500 MHz, D_2O, ^{19}F at -199.45 ppm, ^1H at 4.34 ppm, Δ_1, Δ_2 = 5.00 ms, varying τ_m, ns = 256, ds = 2) spectra of α-*p*-FDGlc-2.

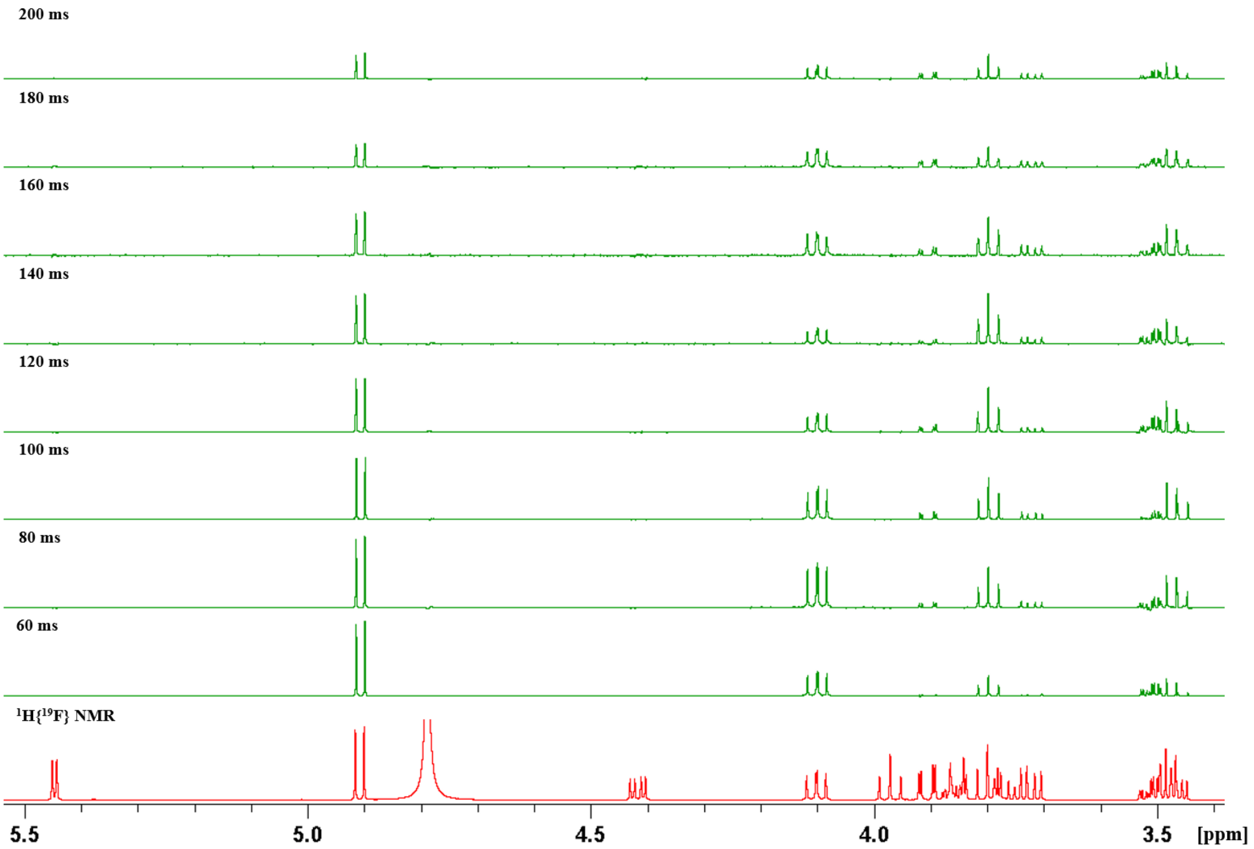

Figure S7. $^{1}H\{^{19}F\}$ SRI-FESTA NMR (500 MHz, D_2O, ^{19}F at -199.34 ppm, ^{1}H at 4.02 ppm, Δ_1, Δ_2 = 5.00 ms, varying τ_m, ns = 256, ds =2) of β-*p*-FDGlc-2.

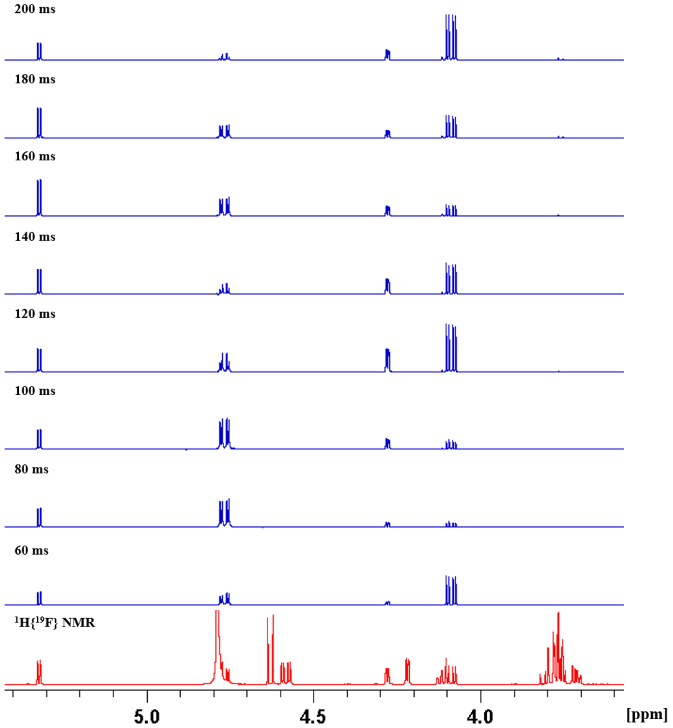

Figure S8. ^{1}H{^{19}F} SRI-FESTA NMR (500 MHz, D_2O, ^{19}F at -203.32 ppm, ^{1}H at 4.68 ppm, Δ_1, Δ_2 = 5.10 ms, varying τ_m, ns = 256, ds =2) of α-*p*-FDGal-3.

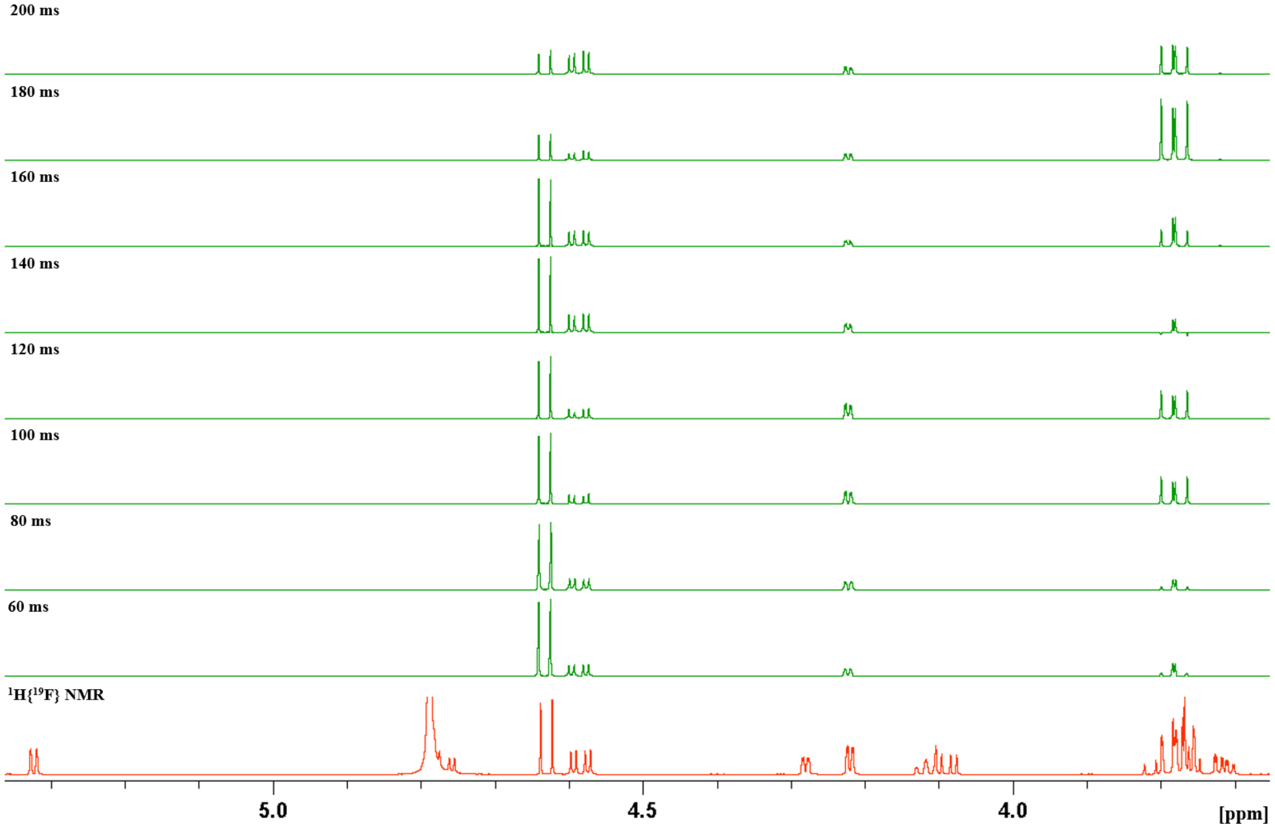

Figure S9. ^{1}H{^{19}F} SRI-FESTA NMR (500 MHz, D_2O, ^{19}F at -199.22 ppm, ^{1}H at 4.50 ppm, Δ_1, Δ_2 = 5.10 ms, varying τ_m, ns = 256, ds = 2) of β-*p*-FDGal-3.

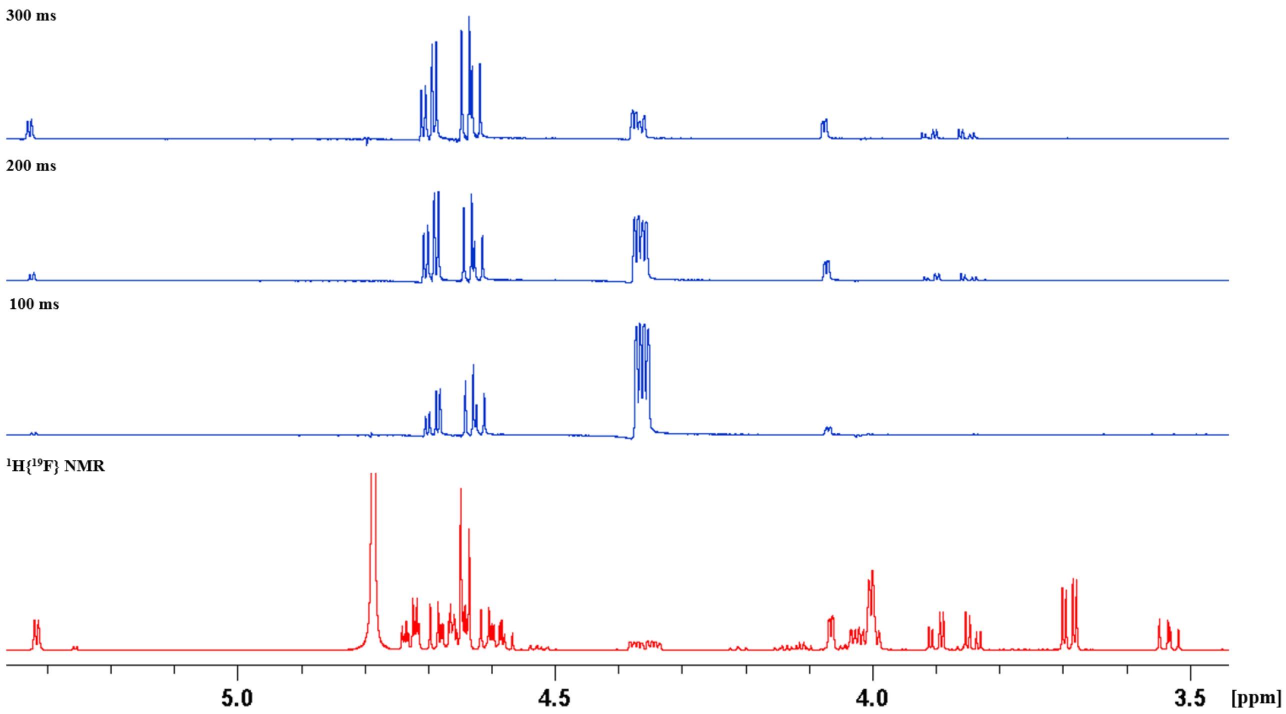

Figure S10. $^{1}H\{^{19}F\}$ SRI-FESTA NMR (600 MHz, D_2O, ^{19}F at -229.73 ppm, ^{1}H at 4.28 ppm, Δ_1, Δ_2 = 15.63 ms, varying τ_m) of α-*p*-FDGal-6.

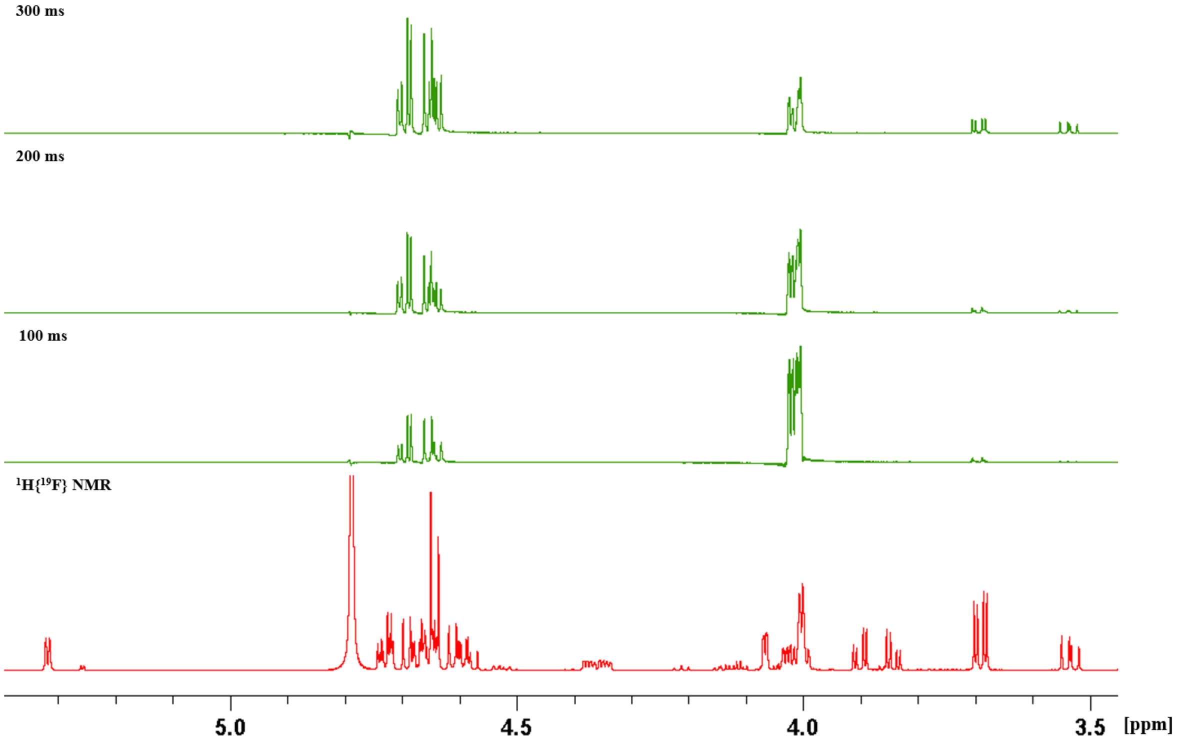

Figure S11. $^{1}H\{^{19}F\}$ SRI-FESTA NMR (600 MHz, D_2O, ^{19}F at -229.73 ppm, ^{1}H at 3.93 ppm, Δ_1, Δ_2 = 15.63 ms, varying τ_m) of β-*p*-FDGal-6.

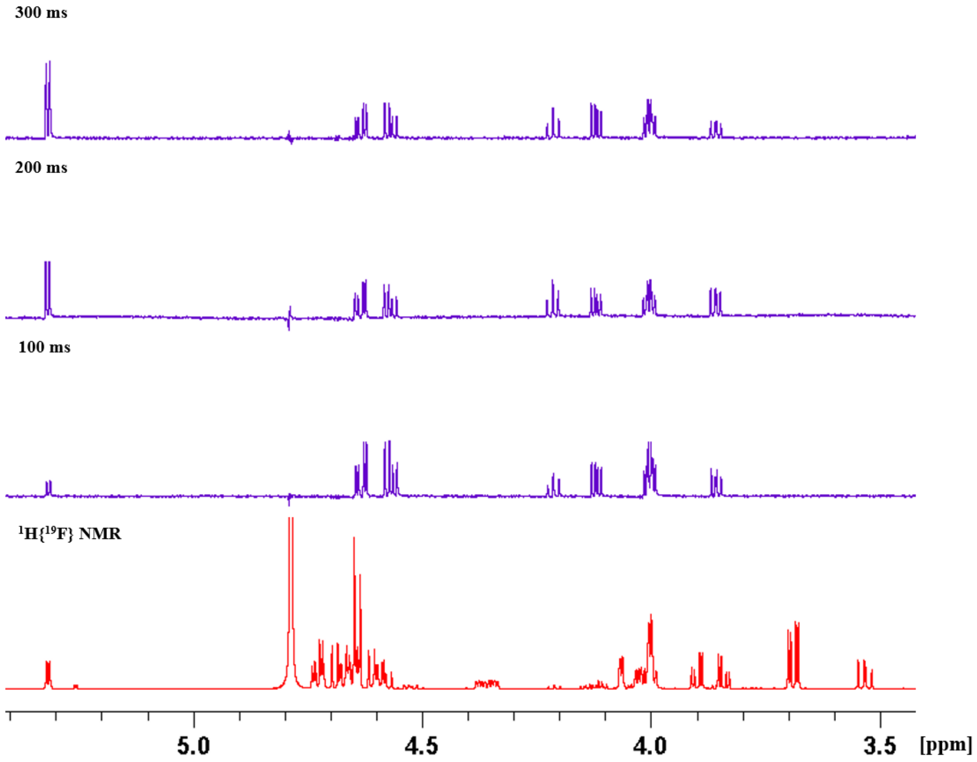

Figure S12. $^{1}H\{^{19}F\}$ SRI-FESTA NMR (600 MHz, D_2O, ^{19}F at -230.79 ppm, ^{1}H at 3.93 ppm, Δ_1 = 6.94 ms, Δ_2 = 15.63 ms, varying τ_m, ns = 128, ds = 4) of α-*f*-FDGal-6.

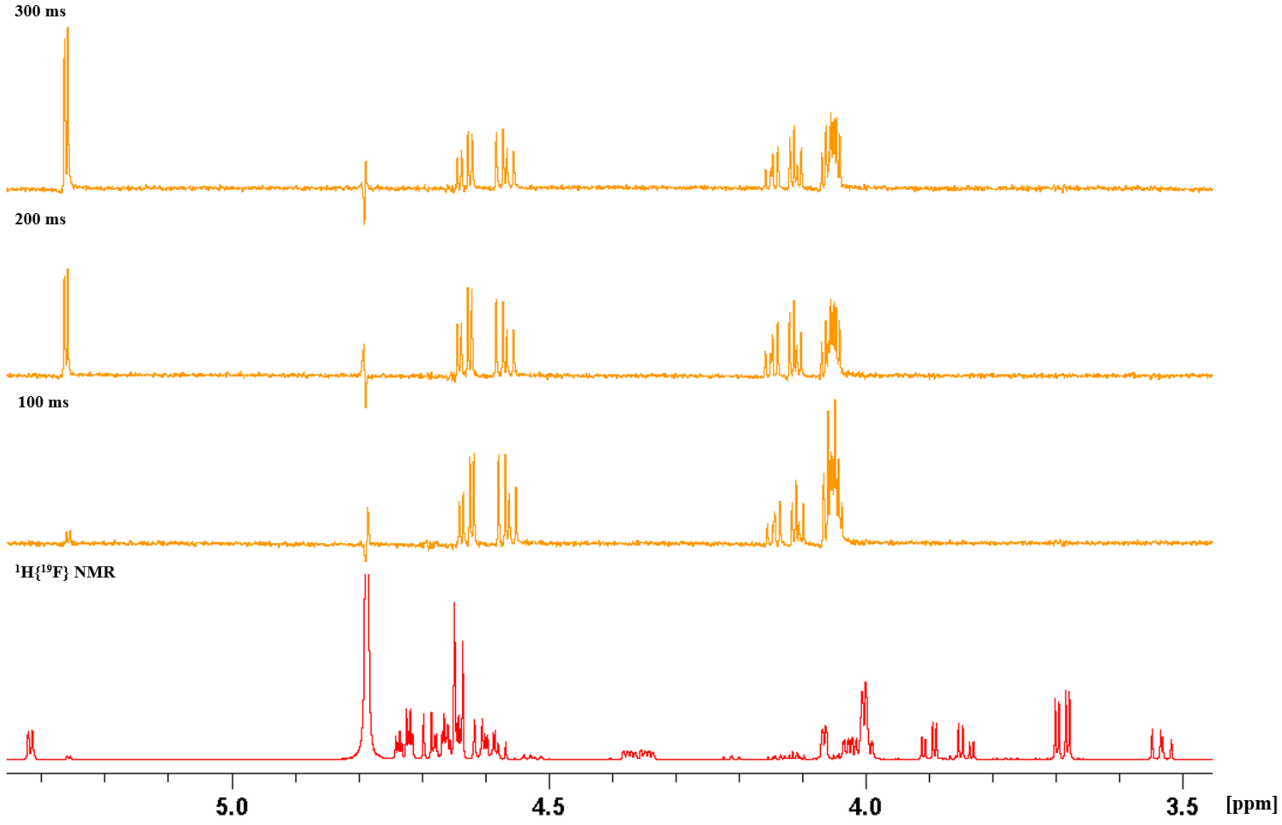

Figure S13. $^{1}H\{^{19}F\}$ SRI-FESTA NMR (600 MHz, D_2O, ^{19}F at -229.16 ppm, ^{1}H at 4.66 ppm, Δ_1, Δ_2 = 15.63 ms, varying τ_m, ns = 128, ds = 4) of β-*f*-FDGal-6.

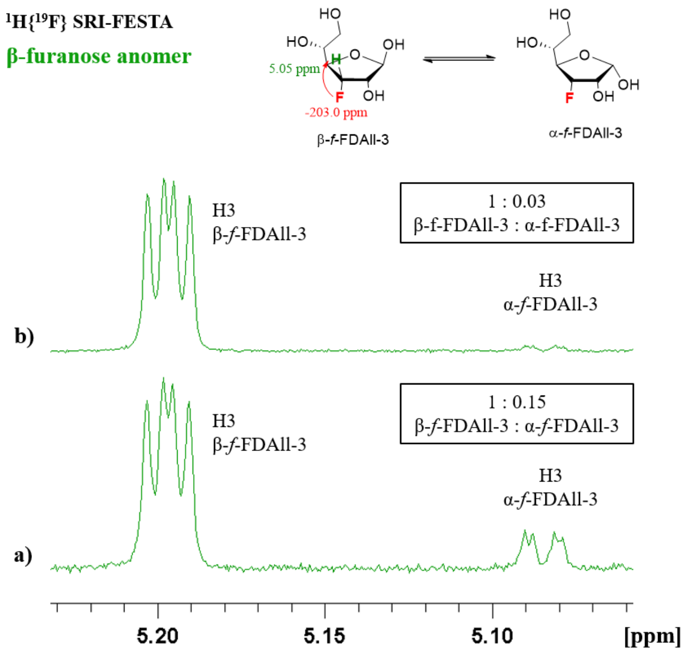

Figure S14. $^1H\{^{19}F\}$ SRI-FESTA NMR spectra expansion at H3 of β-*f*-FDAll-3 in D_2O, 600 MHz (Δ_1, Δ_2 = 4.63 ms, ns = 1024, ds = 4). a) *selection of* ^{1}H*3: mixing time* τ_m = 0 ms and a total ZQS pulse duration of 85 ms, b) *selection of* ^{1}H*3: mixing time* τ_m = 0 ms and total ZQS pulse duration of 20 ms.

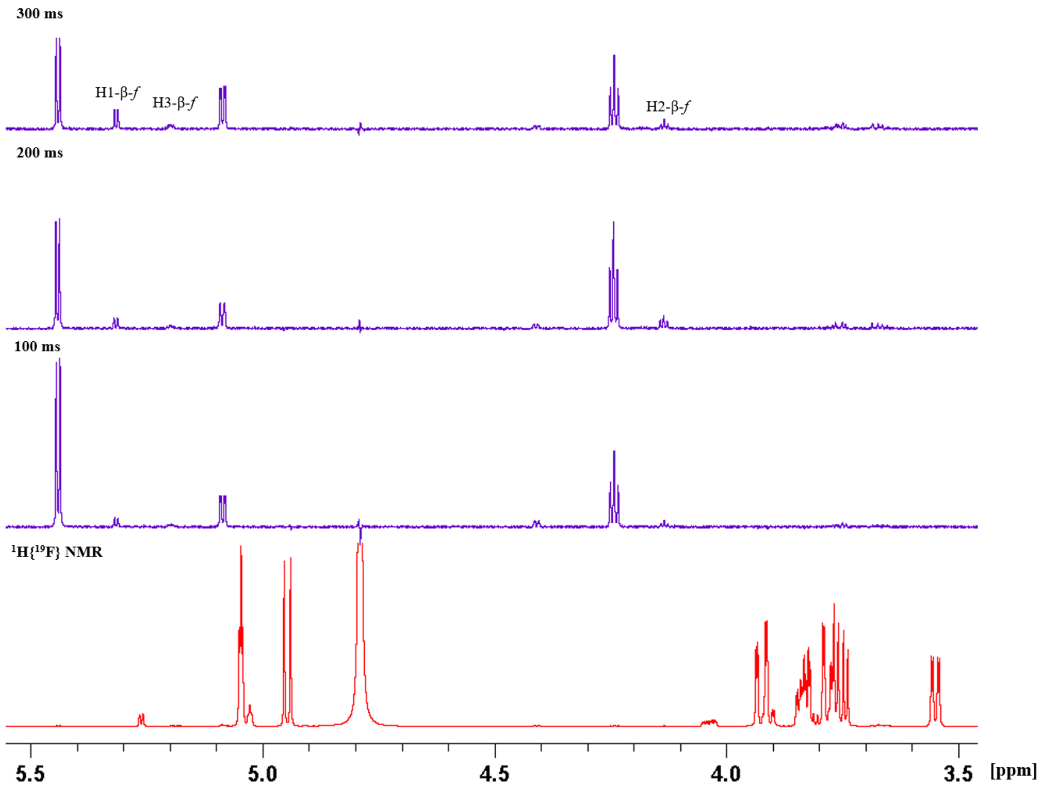

Figure S15. $^1H\{^{19}F\}$ SRI-FESTA NMR (600 MHz, D_2O, ^{19}F at -196.00 ppm, ^1H at 5.05 ppm, ZQS pulse duration of 20 ms, varying τ_m, Δ_1, Δ_2 = 4.63 ms, ns = 1024, ds = 4) of α-*f*-FDAll-3.

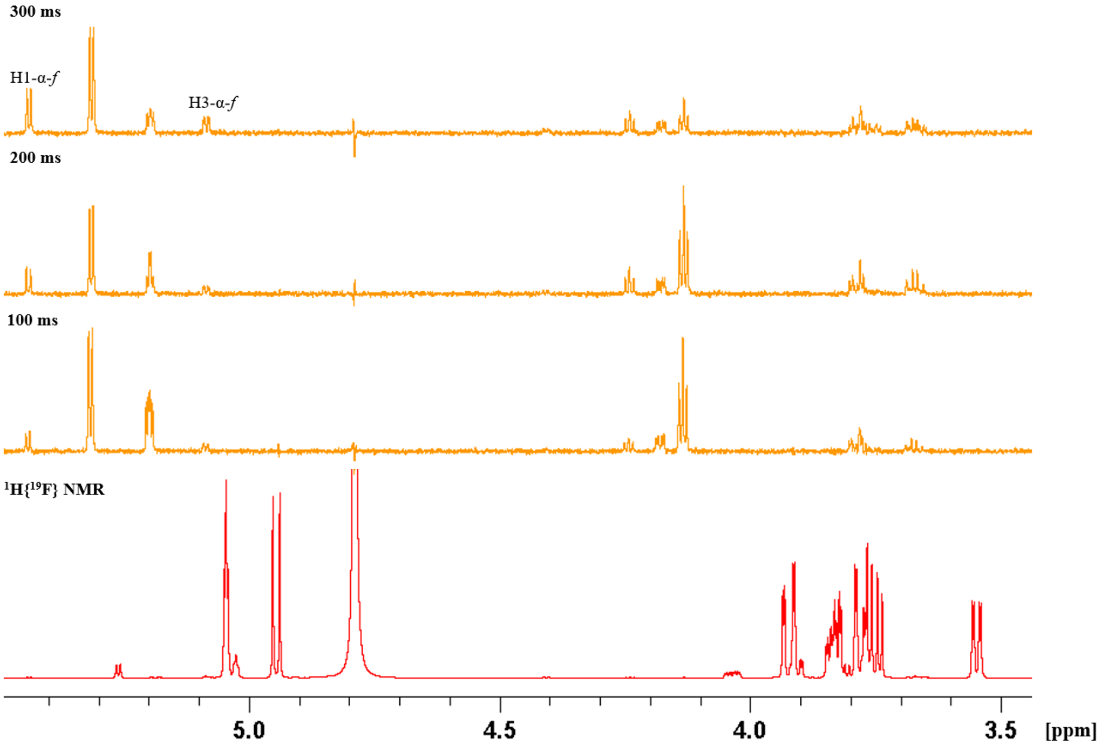

Figure S16. $^1H\{^{19}F\}$ SRI-FESTA NMR (600 MHz, D_2O, ^{19}F at -203.00 ppm, ^1H at 5.05 ppm, ZQS pulse duration of 20 ms*,* varying τ_m, Δ_1, Δ_2 = 4.63 ms, ns = 1024, ds = 4) of β-*f*-FDAll-3.

4 Figures S17-S23: SRI-FESTA and sel-TOCSY experiments

4.1 Figure S17: ^{1}H-$^{1}H\{^{19}F\}$ sel-TOCSY and $^{1}H\{^{19}F\}$ SRI-FESTA NMR subspectra of FDMan-2 (9) (F3 → H1 and F3 → H2)

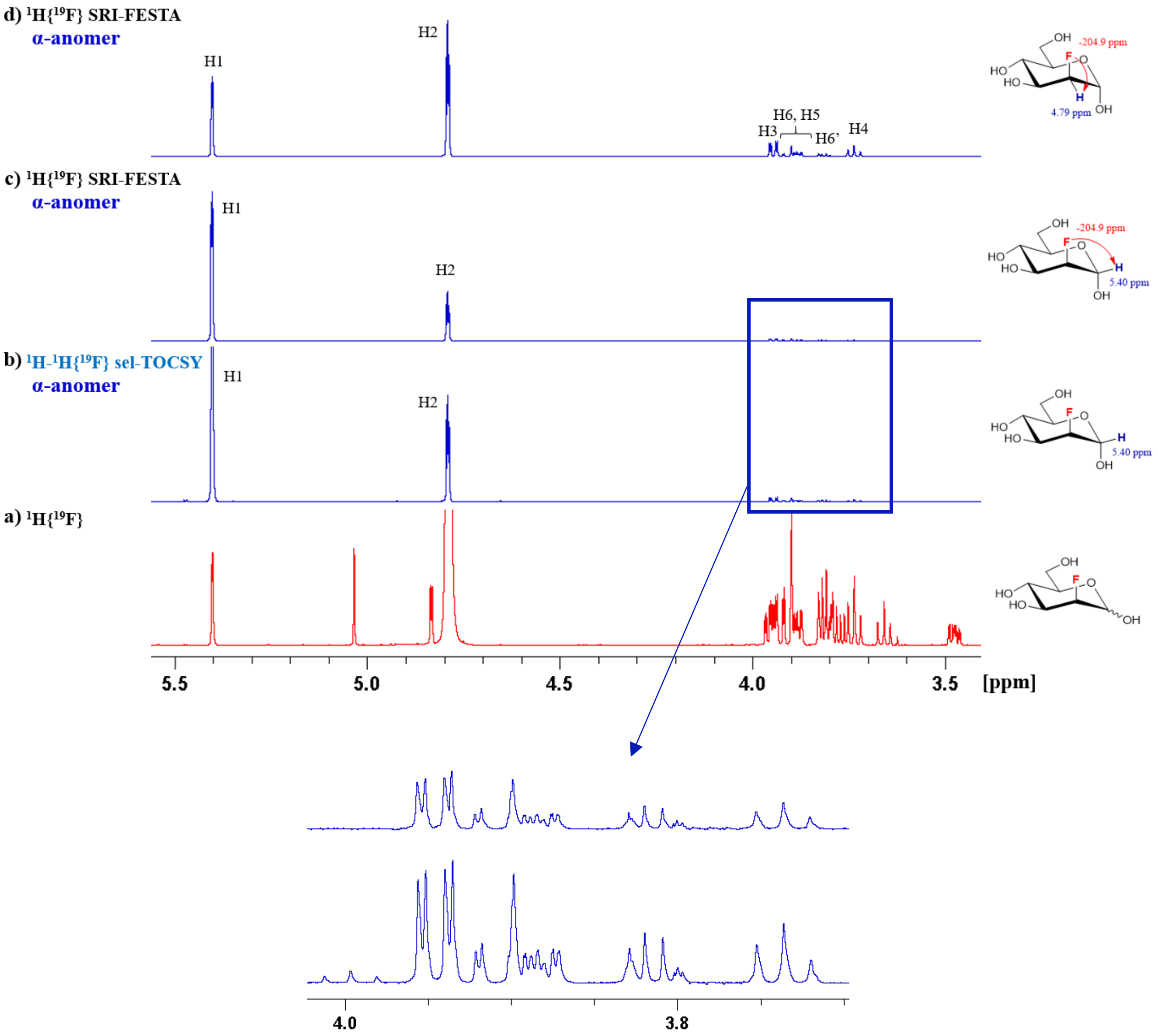

Figure S17. NMR spectra of FDMan-2, all in D_2O, 600 MHz. a) $^{1}H\{^{19}F\}$ NMR spectrum; b) ^{1}H-$^{1}H\{^{19}F\}$ sel-TOCSY NMR spectrum of the α-anomer (*selection of* ^{1}H*1: mixing time* 100 ms); c-d) $^{1}H\{^{19}F\}$ SRI-FESTA NMR spectrum of the α-anomer (*mixing time* 100 ms, ds = 8); c) *selection of* ^{1}H1 (Δ_1, Δ_2 = 33.33 ms); d) *selection of* ^{1}H2 (Δ_1, Δ_2 = 15.15 ms).

4.2 Figure S18 (^{1}H-^{1}H{^{19}F} sel-TOCSY and ^{1}H{^{19}F} SRI-FESTA NMR subspectra of FDGal-46 (7) (F3 → H1 and F3 → H2)

Just as for FDGal-3, the sel-TOCSY spectrum (selection of H1) of α-*p*-FDGal-46 yields only weak responses for H5 and H6 at 200 ms (Figure S18d). For the β-anomer, the H1 proton overlaps with the H6 protons of both anomers, meaning the TOCSY transfer starts from both ends of the spin system and the subspectrum is contaminated with signals from the α-anomer. SRI-FESTA employing a F4 → H4 transfer yields clear H5 and H6 signals for the α-anomer (Figure S18e), but for the β-anomer (Figure S18c), only the signals up to H5 are visible, a situation similar to FDGal-3.

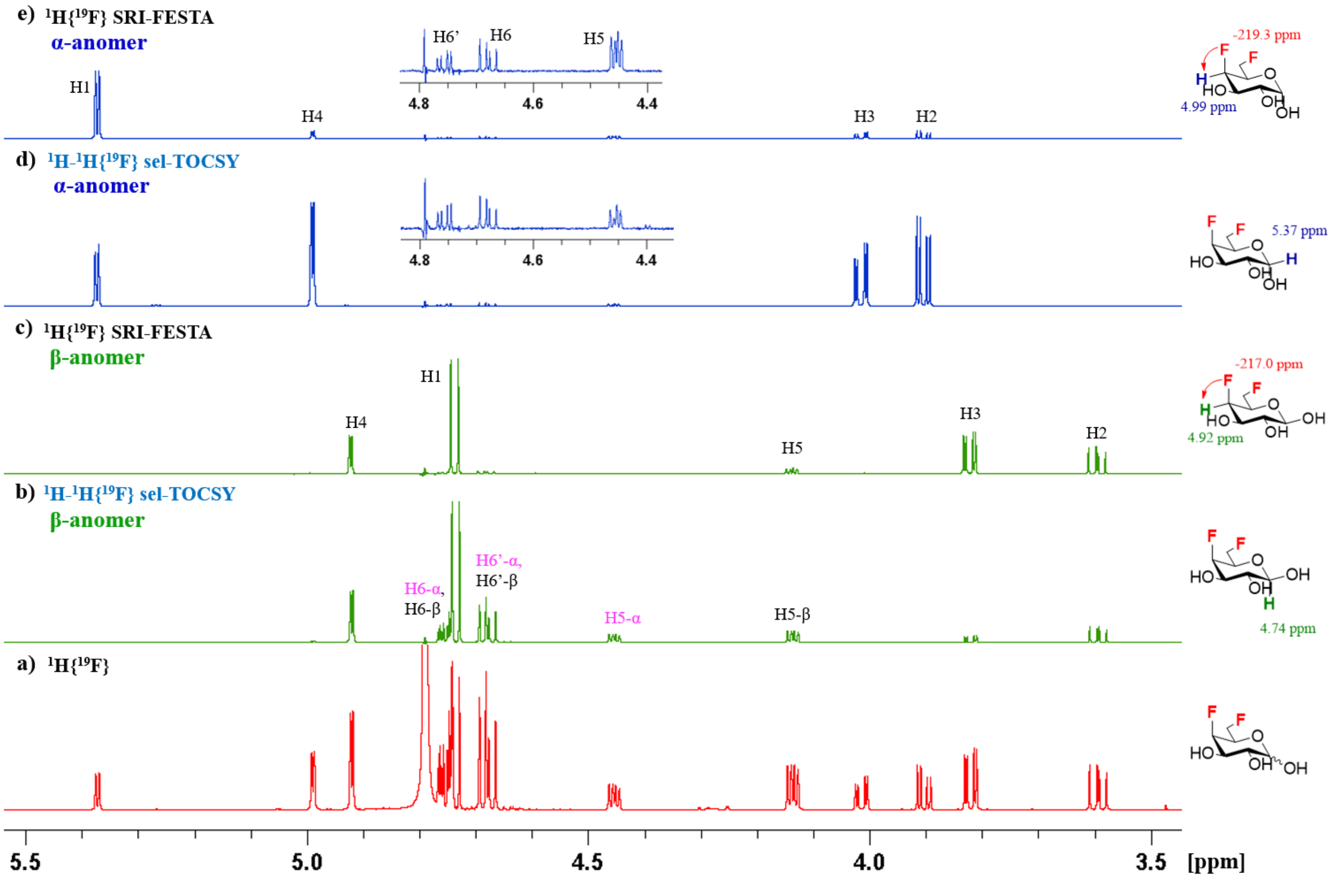

NMR spectra of FDGal-46 in D_2O, 600 MHz. a) ^{1}H{^{19}F} NMR spectrum; b) ^{1}H-^{1}H{^{19}F} sel-TOCSY NMR spectrum of the β-anomer (*selection of* ^{1}H*1*: *mixing time* 200 ms); c) ^{1}H{^{19}F} SRI-FESTA spectrum of the β-anomer (*selection of* ^{1}H*4*: *mixing time* 300 ms, Δ_1, Δ_2 = 15.00 ms); d) ^{1}H-^{1}H{^{19}F} sel-TOCSY NMR spectrum of the α-anomer (*selection of* ^{1}H*1: mixing time* 200 ms); e) ^{1}H{^{19}F} SRI-FESTA spectrum of the α-anomer (*selection of* ^{1}H*4*: *mixing time* 300 ms, Δ_1, Δ_2 = 15.00 ms).

4.3 Figure S19: ^{1}H{^{19}F} SRI-FESTA NMR subspectra of FDAll-3 (10) (F3 → H3)

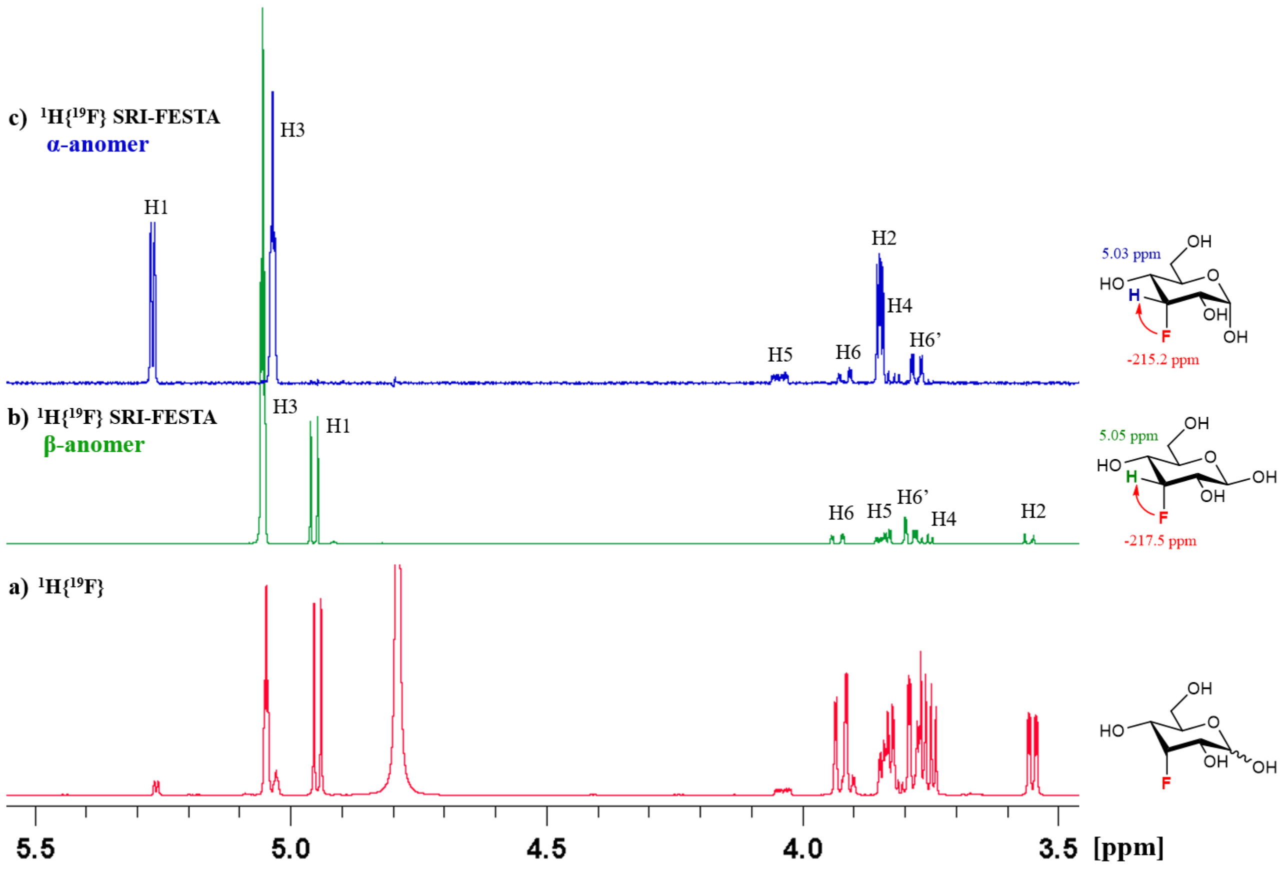

a) ^{1}H{^{19}F} NMR spectrum of FDAll-3; b) ^{1}H{^{19}F} SRI-FESTA NMR spectrum of β-*p*-FDAll-3 (*selection of* ^{1}H*3*+^{1}H*1: mixing time* 100 ms, Δ_1, Δ_2 = 4.63 ms); c) ^{1}H{^{19}F} SRI-FESTA NMR spectrum of α-*p*-FDAll-3 (*selection of* ^{1}H*3*+^{1}H*1: mixing time* 100 ms, Δ_1, Δ_2 = 4.63 ms). All in D_2O, 600 MHz.

The sel-TOCSY spectrum of α-*p*-FDAll-3 starting from H1 is of lesser use as it shows contamination with the minor furanose form, since the furanose H3 multiplet overlaps with the H1 of the pyranose.

4.4 Figure S20. $^{1}H\{^{19}F\}$ SRI-FESTA NMR subspectra of FDGlc-46 (5) (selection of either F4 and F6)

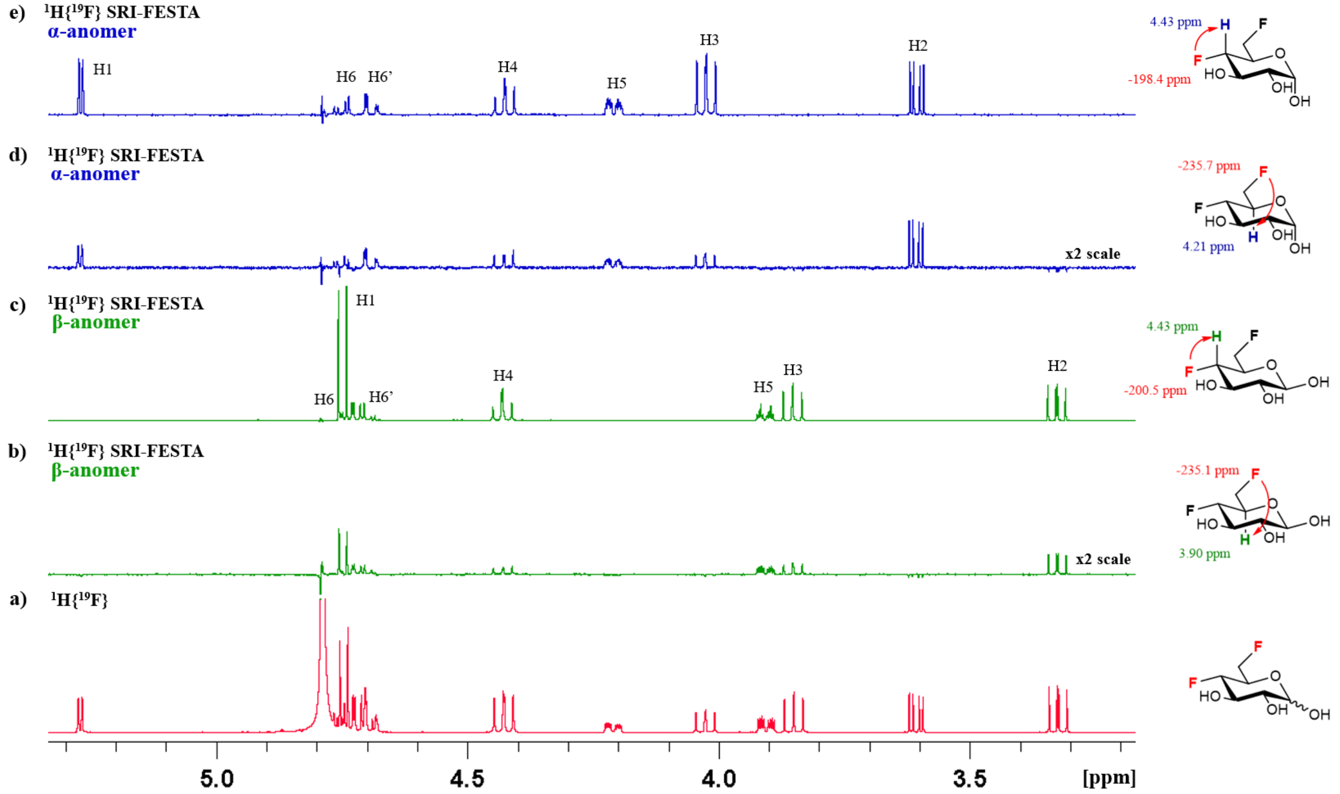

$^{1}H\{^{19}F\}$ SRI-FESTA of FDGlc-46 in D_2O, 500 MHz. a) $^{1}H\{^{19}F\}$ NMR spectrum; b) $^{1}H\{^{19}F\}$ SRI-FESTA NMR spectrum of the β-anomer (*selection of* ^{19}F*6 to* ^{1}H*5: mixing time* 160 ms, ds = 2, Δ_1, Δ_2 = 5.00 ms); c) $^{1}H\{^{19}F\}$ SRI-FESTA NMR spectrum of the β-anomer (*selection of* ^{19}F*4 to* ^{1}H*4: mixing time* 160 *ms,* ns = 256, ds = 2, Δ_1, Δ_2 = 5.00 ms); d) $^{1}H\{^{19}F\}$ SRI-FESTA NMR spectrum of the α-anomer (*selection of* ^{19}F*6 to* ^{1}H*5: mixing time* 160 ms, ns = 256, ds = 2, Δ_1, Δ_2 = 5.00 ms); e) $^{1}H\{^{19}F\}$ SRI-FESTA NMR spectrum of the α-anomer (*selection of* ^{19}F*4 to* ^{1}H*4: mixing time* 160 ms, ns = 256, ds = 2, Δ_1, Δ_2 = 5.00 ms). *b-e) delays* Δ_1*,* Δ_2 *not optimised.*

4.5 Figure S21: ^{1}H{^{19}F} SRI-FESTA NMR subspectra of β-*f*-FDAll-3 (3-deoxy-3-fluoro-β-D-allofuranose) (F3 → H3 and F3 → H2+H4)

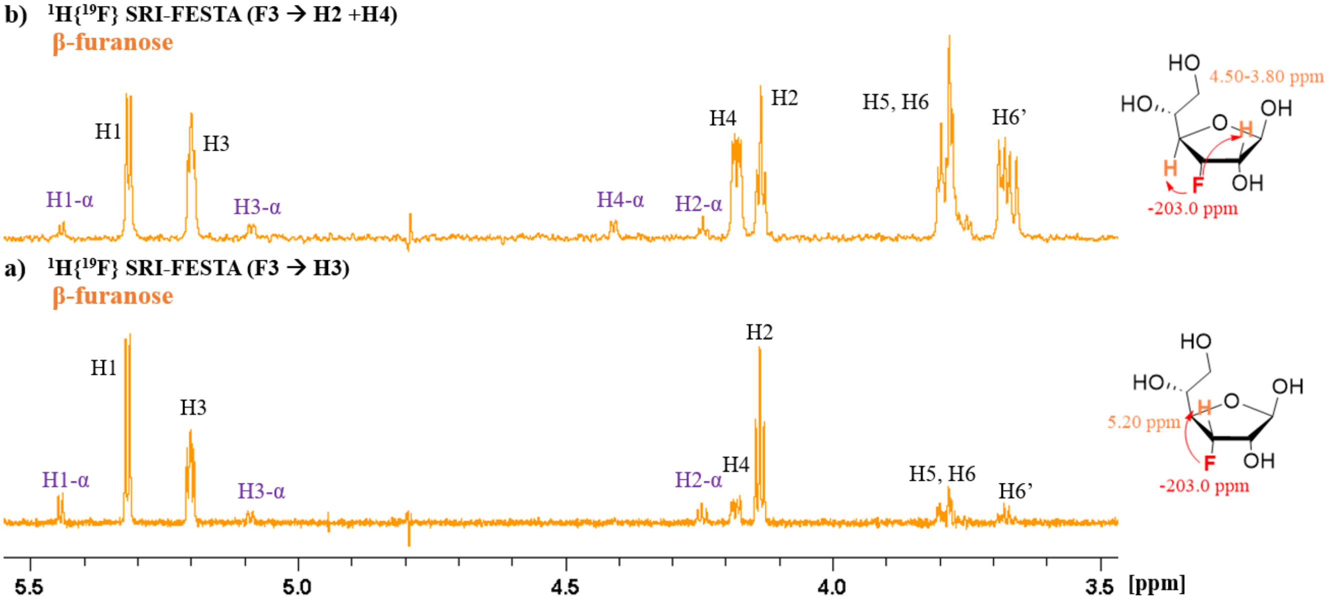

^{1}H{^{19}F} SRI-FESTA NMR spectrum of β-*f*-FDAll-3 in D_2O, 600 MHz. a) *selection of* ^{1}H*3: mixing time* 100 ms, Δ_1, Δ_2 = 4.63 ms, total ZQS pulse duration of 20 ms, ns = 1024; b) *selection of* ^{1}H*4*+^{1}H*2: mixing time* 100 ms, Δ_1 = 5.21 ms, Δ_2 = 10.42 ms, total ZQS pulse duration of 20 ms, ns = 1024.

4.6 Figure S22: $^1H\{^{19}F\}$ SRI-FESTA NMR subspectra of FDGal-3 furanoses (F3 → H3)

In the case of FDGal-3, similarly as for FDGal-6, even though pyranose tautomers exhibited small $^3J_{H4-H5}$ scalar couplings (1.1 and 1.0 Hz for α- and β-pyranoses respectively, Table 1) impeding TOCSY transfer, this was not the case for furanoses (5.6 and 4.5 Hz for α- and β-furanose anomers respectively). The H6 environments were already observed at 200 ms, however the H5 environments for both furanose anomers were too weak to extract *J*-coupling constants which was further worsened by the more complex splitting pattern (*ddd*) compared to other environments. In general, the signal-to-noise ratio was considerably low for both α and β-furanoses, which was exacerbated for α-furanose due to its low concentration. Notably, to extract *J*-coupling constants for H5 resonances *via* SRI-FESTA experiments, required 4096 number of scans and mixing time were extended to 300 ms for the β-tautomer (see Figure S22). At spinlock times of 300 ms, a negligible spill-over of the other anomer were observed for both furanoses.

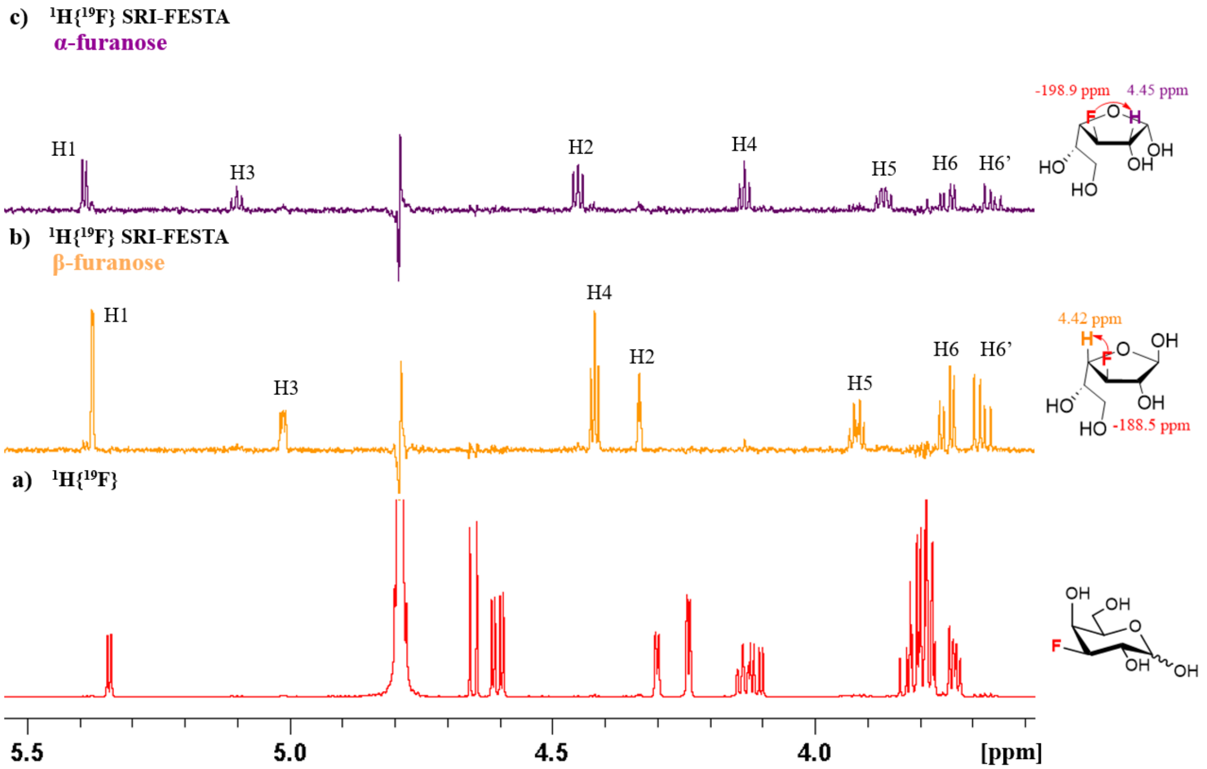

NMR spectra of FDGal-3 furanoses in D_2O, 600 MHz. a) $^1H\{^{19}F\}$ NMR of FDGal-3; b) $^1H\{^{19}F\}$ SRI-FESTA NMR spectrum of β-furanose (*selection of* ^{1}H*4: mixing time* 300 ms, $\Delta_1 = \Delta_2 = 30.86$ ms, 4096 ns); c) $^1H\{^{19}F\}$ SRI-FESTA NMR spectrum of α-furanose (*selection of* ^{1}H*2: mixing time* 200 ms, $\Delta_1 = \Delta_2 = 39.47$ ms, 4096 ns).

5 Tables S2 and S3: Corrections and additional multiplet characterization of fluorinated carbohydrates

5.1 Table S2. Detailed $^1H\{^{19}F\}$ spectra characterization of FDGal-3 furanoses in D_2O.

As reported[a] by Blanchard *et al.* [7]	Full characterization by SRI-FESTA analysis[b]
4.21 (H4α) ppm<br>3.80 (H6β, H6'β) ppm	H4α (4.13 ppm, appt dt, J_{HH} = 5.5 Hz, J_{HF} = 22.0 Hz)<br>H6β (3.75 ppm, dd, J_{HH} = 11.8, 4.5 Hz)<br>H6'β (3.68 ppm, dd, J_{HH} = 11.8, 6.8 Hz)
H5α, H6α, H6'α not assigned	H5α (3.87 ppm, ddd, J_{HH} = 6.5, 5.8, 4.3 Hz)<br>H6α (3.75 ppm, dd, J_{HH} = 12.0, 4.3 Hz)<br>H6'α (3.66 ppm, dd, J_{HH} = 12.0, 6.5 Hz)
5.49 (H1β, s) ppm<br>4.44 (H2β, d, J_{HF} = 16.7 Hz) ppm<br>4.02 (H5β, dd, J_{HF} = 24.1 Hz, J_{HH} = 9.0 Hz) ppm	H1β (5.38 ppm, d, J_{HH} = 1.7 Hz)<br>H2β (4.34 ppm, ddd, J_{HH} = 2.2, 1.7 Hz, J_{HF} = 16.5 Hz)<br>H5β (3.92 ppm, dt, J_{HH} = 6.8, 4.6 Hz)

[a] at 37 °C, 600 MHz; ^1H NMR assignments; D_2O reference shift not stated; [b] 4096 scans, 27 °C, 600 MHz.

5.2 Table S3. More detailed $^1H\{^{19}F\}$ spectra characterization of pyranoses in D_2O at 300 K, 600 MHz.

Sugar (ref)	Reported multiplet	Full characterization obtained by SRI-FESTA analysis
FDGlc-2 ([8])[a]	3.50–3.41 (H5β, H4α, H4β) ppm	H5β (3.51 ppm, ddd, J = 9.9, 5.6, 2.1 Hz)[f]<br>H4α (3.48 ppm, ddd, J = 9.9, 9.3, 0.6 Hz)[f]<br>H4β (3.47 ppm, ddd, J = 9.9, 8.8, 0.6 Hz)[f]
FDMan-2 ([9])[a,b]	4.69 (H2β) ppm[c]<br>4.65 (H2α) ppm[c]	H2β (4.83 ppm, dd, J = 51.3, 2.6, 0.6 Hz)<br>H2α (4.73 ppm, ddd, J = 49.3, 2.5, 1.9 Hz)
	3.90 (H3α) ppm	H3α (3.94 ppm, ddd, J = 31.4, 9.7, 2.6 Hz)
	3.82, 3.72 ppm (m)	H6β (3.96 ppm, dd, J = 12.3, 2.3, Hz)<br>H6'α (3.82 ppm, dd, J = 12.3, 5.7 Hz)<br>H3β (3.80 ppm, ddd, J = 30.8, 9.8, 2.5 Hz)<br>H6'β (3.78 ppm, dd, J = 12.3, 6.2 Hz)
	3.40 (H5β) ppm	H5β (3.47 ppm, dddd, J = 9.8, 6.2, 2.3, 0.7 Hz)

FDGal-3 (7)[d]	4.23 (H5α, H2α) ppm	H5α (4.14 ppm, dddd, J = 6.7, 5.8, 1.7, 1.1 Hz)<br>H2α (4.11 ppm, ddd, J = 12.0, 10.0, 4.1 Hz)
	3.91 (H2β) ppm<br>3.90 (H6β, H6'β) ppm	H6β (3.82, ddd, J = 11.5, 7.7, 0.7 Hz)<br>H2β (3.80 ppm, ddd, J = 13.0, 9.7, 7.9 Hz)<br>H6'β (3.78 ppm, dd, J = 11.5, 4.5 Hz)
	3.84 (H5β) ppm	H5β (3.74 ppm, ddd, J = 7.7, 4.5, 1.7, 1.0 Hz)
FDGal-46 (10)[f]	H6α, H6β not assigned[c]	H6α (4.76 ppm, ddd, J = 45.6, 10.2, 4.0 Hz)<br>H6β (4.74 ppm, ddd, J = 45.3, 10.3, 4.1 Hz)
FDAll-3 (11)[b,e,f]	3.67–3.56 (H4α) ppm	H4α (3.68 ppm, ddd, J = 29.5, 10.3, 2.4 Hz)

[a] 400 MHz; [b] D_2O referenced at 4.70 ppm; [c] resonances were under broad D_2O peak; [d] at 35 °C, 600 MHz; [e] from ^1H NMR; [f] 500 MHz;

6 Experimental details for figures in main manuscript

6.1 Table S4. Experimental details for Figure 3.

Label	Experiment[a]	Component	Transfer	δ ^{19}F [ppm]	δ ^1H[b] [ppm]	Δ_1, Δ_2 [ms]	τ_m [ms]
a)	^1H	FDGal-6	-	-	-	-	-
b)	$^1H\{^{19}F\}$	FDGal-6	-	-	-	-	-
c)	$^1H\{^{19}F\}$ SRI-FESTA	β-*p*-FDGal-6	F6→H5	-229.73	3.93	15.63	300
d)	$^1H\{^{19}F\}$ SRI-FESTA	α-*p*-FDGal-6	F6→H5	-229.73	4.28	15.63	300
e)	$^1H\{^{19}F\}$ SRI-FESTA	β-*f*-FDGal-6	F6→H5	-229.26	3.93	15.63	100
f)	$^1H\{^{19}F\}$ SRI-FESTA	α-*f*-FDGal-6	F6→H5	-230.79	3.93	15.63	100

[a] 600 MHz, D_2O referenced at 4.70 ppm, $^1H\{^{19}F\}$ SRI-FESTA for furanoses acquired with *ns* = 128 and *ds* = 4; [b] centre of the selected range.

6.2 Table S5. Experimental details for Figure N6.

Label	Experiment[a]	Component	Transfer	δ ^{19}F [ppm]	δ ^1H[b] [ppm]	Δ_1, Δ_2 [ms]	τ_m [ms]
a)	$^1H\{^{19}F\}$	FDGlc-4	-	-	-		-
b)	$^1H\{^{19}F\}$ SRI-FESTA	β-*p*-FDGlc-4	F4→H4	-198.21	4.25	5.00	100
c)	$^1H\{^{19}F\}$ SRI-FESTA	β-*p*-FDGlc-4	F4→H2	-198.21	5.15	4.90[c]	160

d)	^{1}H{^{19}F} SRI-FESTA	α-*p*-FDGlc-4	F4→H4	-200.23	4.25	4.90[c]	100
e)	^{1}H{^{19}F} SRI-FESTA	α-*p*-FDGlc-4	F4→H1	-200.23	3.20	5.00	160

[a] 500 MHz, D_2O referenced at 4.70 ppm, ^{1}H{^{19}F} SRI-FESTA acquired with *ns* = 256 and *ds* = 2; [b] centre of the selected range; [c] delays not optimised.

6.1 Table S6. Experimental details for Figure 7.

Label	Experiment[a]	Component	Transfer	δ ^{19}F [ppm]	δ ^{1}H[b] [ppm]	Δ_1, Δ_2 [ms]	τ_m [ms]	ZQS pulse [ms]
a)	^{1}H{^{19}F} SRI-FESTA	α-*f*-FDAll-3	F3→H3	-196.00	5.05	4.63	0	85
b)	^{1}H{^{19}F} SRI-FESTA	α-*f*-FDAll-3	F3→H3	-196.00	5.05	4.63	0	20

[a] 600 MHz, D_2O referenced at 4.70 ppm, ^{1}H{^{19}F} SRI-FESTA acquired with *ns* = 128 and *ds* = 4; [b] centre of the selected range.

6.2 Table S7. Experimental details for Figure 8.

Label	Experiment[a]	Component	Transfer	δ ^{19}F [ppm]	δ ^{1}H[b] [ppm]	Δ_1, Δ_2 [ms]	τ_m [ms]
a)	^{1}H{^{19}F}	FDMan-2	-	-	-	-	-
b)	^{1}H-^{1}H{^{19}F} sel-TOCSY	β-*p*-FDMan-2	H1	-	4.95	-	200
c)	^{1}H{^{19}F} SRI-FESTA	β-*p*-FDMan-2	F2→H1	-223.15	4.95	1.25	200
d)	^{1}H{^{19}F} SRI-FESTA	β-*p*-FDMan-2	F2→H2	-223.15	4.71	15.15	200
e)	^{1}H{^{19}F} SRI-FESTA	β-*p*-FDMan-2	F2→H3	-223.15	3.79	25.00	200

[a] 600 MHz, D_2O referenced at 4.70 ppm; [b] centre of the selected range.

6.3 Table S8. Experimental details for Figures 9 and 11.

Label	Experiment[a]	Component	Transfer	δ ^{19}F [ppm]	δ ^{1}H[b] [ppm]	Δ_1, Δ_2 [ms]	τ_m [ms]
a)	^{1}H{^{19}F}	FDGal-3	-	-	-	-	-
b)	^{1}H-^{1}H{^{19}F} sel-TOCSY	β-*p*-FDGal-3	H1	-	4.57	-	300
c)	^{1}H{^{19}F} SRI-FESTA	β-*p*-FDGal-3	F3→H4	-199.18	4.15	41.67	300
d)	^{1}H{^{19}F} SRI-FESTA	β-*p*-FDGal-3	F3→H5	-199.18	3.65	125.00	300

e)	^{1}H-^{1}H{^{19}F} sel-TOCSY	α-*p*-FDGal-3	H1	-	5.26	-	300
f)	^{1}H{^{19}F} SRI-FESTA	α-*p*-FDGal-3	F3→H4	-203.29	4.22	35.71	200
g)	^{1}H{^{19}F} SRI-FESTA	α-*p*-FDGal-3	F3→H5	-203.29	4.03	90.00	300

[a] 600 MHz, D_2O referenced at 4.70 ppm; [b] centre of the selected range.

6.4 Table S9. Experimental details for Figure 10.

Label	Experiment[a]	Component	Transfer	δ ^{19}F [ppm]	δ ^{1}H[b] [ppm]	Δ_1, Δ_2 [ms]	τ_m [ms]
a)	^{1}H	FDGlc-2	-	-	-	-	-
b)	^{1}H{^{19}F}	FDGlc-2	-	-	-	-	-
c)	^{1}H{^{19}F} SRI-FESTA	β-*p*-FDGlc-2	F2→H2	-199.34	4.02	5.00	100
d)	^{1}H{^{19}F} SRI-FESTA	α-*p*-FDGlc-2	F2→H2	-199.45	4.34	5.00	100

[a] 500 MHz, D_2O referenced at 4.70 ppm, ^{1}H{^{19}F} acquired with *ns* = 32 and ^{1}H{^{19}F} SRI-FESTA acquired with *ns* = 256 and *ds* = 2; [b] centre of the selected range.

6.5 Table S10. Experimental details for Figure 12.

Label	Experiment[a]	Component	Transfer	δ ^{19}F [ppm]	δ ^{1}H[b] [ppm]	Δ_1, Δ_2 [ms]	τ_m [ms]
a)	^{1}H{^{19}F} SRI-FESTA	β-*p*-FDGal-46	F6→H5	-230.48	4.03	9.00, 16.67	100
b)	^{1}H{^{19}F} SRI-FESTA	β-*p*-FDGal-46	F4→H3	-216.93	3.75	25.00	100

[a] 600 MHz, D_2O referenced at 4.70 ppm; [b] centre of the selected range.

6.6 Table S11. Experimental details for Figure 13.

Label	Experiment[a]	Component	Transfer	δ ^{1}H[b] [ppm]	τ_m [ms]
a)	^{1}H{^{19}F}	FDGal-6	-	-	-
b)	^{1}H-^{1}H{^{19}F} sel-TOCSY	β-*f*-FDGal-6	H1	5.17	200

[a] 600 MHz, D_2O referenced at 4.70 ppm, ^{1}H-^{1}H{^{19}F} sel-TOCSY acquired with *ns* = 128 and *ds* = 4 ; [b] centre of the selected range.

6.1 Table S12. Experimental details for Figure 14.

Label	Experiment[a]	Component	Transfer	δ ^{19}F [ppm]	δ ^{1}H[b] [ppm]	Δ_1, Δ_2 [ms]	τ_m [ms]
a)	$^{1}H\{^{19}F\}$ SRI-FESTA	β-*f*-FDGal-6	F6→H5	-229.26	3.93	15.63	0
b)	$^{1}H\{^{19}F\}$ SRI-FESTA	β-*f*-FDGal-6	F6→H5	-229.26	3.93	15.63	100

[a] 600 MHz, D_2O referenced at 4.70 ppm, $^{1}H\{^{19}F\}$ SRI-FESTA acquired with *ns* = 124 and *ds* = 4; [b] centre of the selected range.

6.2 Table S13. Experimental details for Figure 15.

Label	Experiment[a]	Component	Transfer	δ ^{19}F [ppm]	δ ^{1}H[b] [ppm]	Δ_1, Δ_2 [ms]	τ_m [ms]	ZQS pulse [ms]
a)	$^{1}H\{^{19}F\}$	FDAll-3	-	-	-	-	-	-
b)	$^{1}H\{^{19}F\}$ SRI-FESTA	α-*f*-FDAll-3	F6→H3	-196.00	5.05	4.63	200	20
c)	$^{1}H\{^{19}F\}$ SRI-FESTA	α-*f*-FDAll-3	F6→H4	-196.00	4.34	13.16	200	20
d)	$^{1}H\{^{19}F\}$ SRI-FESTA	β-*f*-FDAll-3	F6→H2+H4	-203.00	4.15	5.21, 10.42	200	20

[a] 600 MHz, D_2O referenced at 4.70 ppm, $^{1}H\{^{19}F\}$ SRI-FESTA acquired with *ns* = 1024 and *ds* = 4; [b]centre of the selected range.

7 Copies of spectra of all compounds and anomers:

7.1 2-Deoxy-2-fluoro-D-glucose (1, FDGlc-2): 46 : 54 α-pyranose / β-pyranose, in D_2O.

7.1.1 FDGlc-2 (**1**): ^{1}**H NMR** (500 MHz, D_2O)

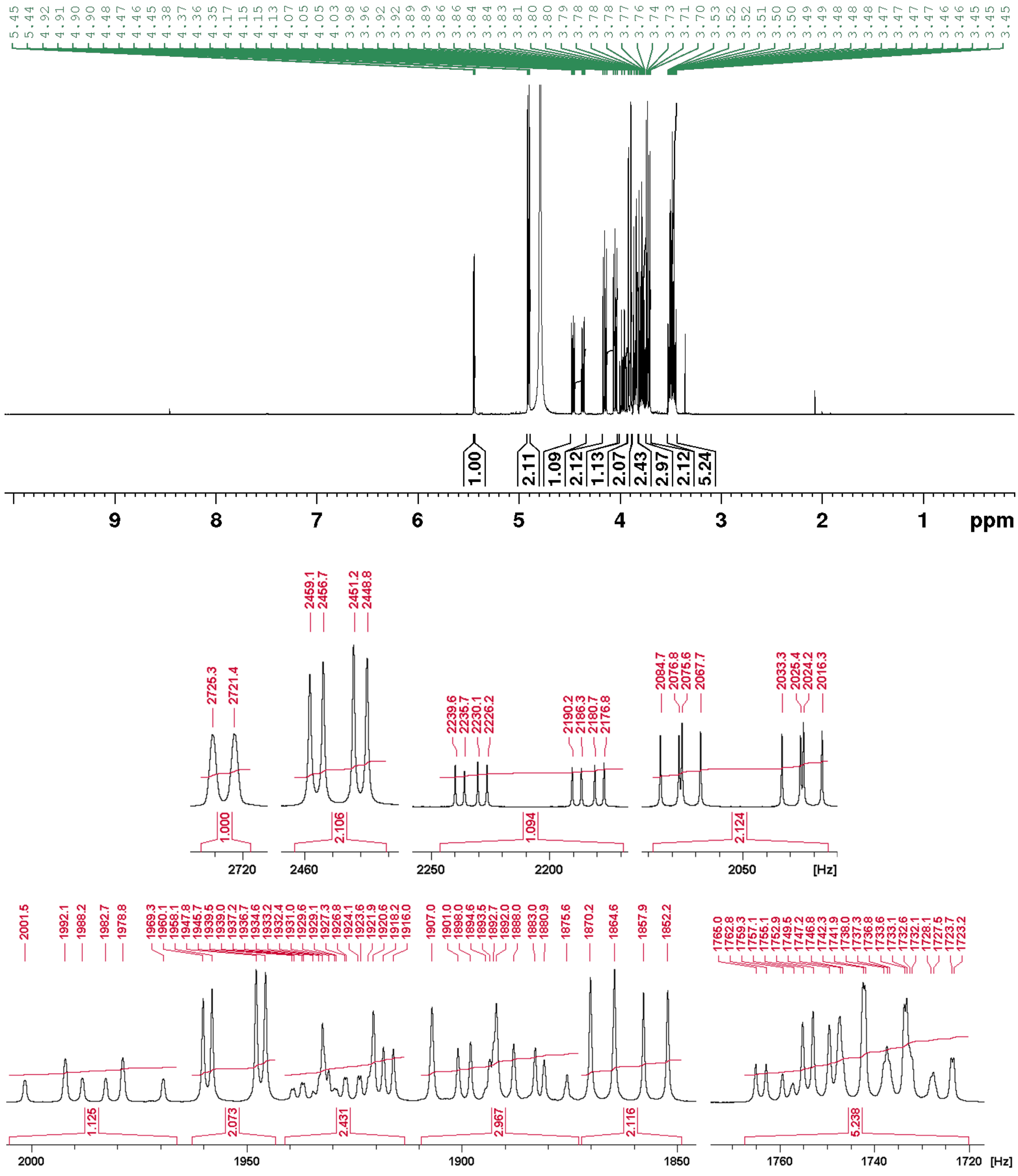

7.1.2 FDGlc-2 (**1**): ^{1}**H{**^{19}**F} NMR** (500 MHz, D_2O)

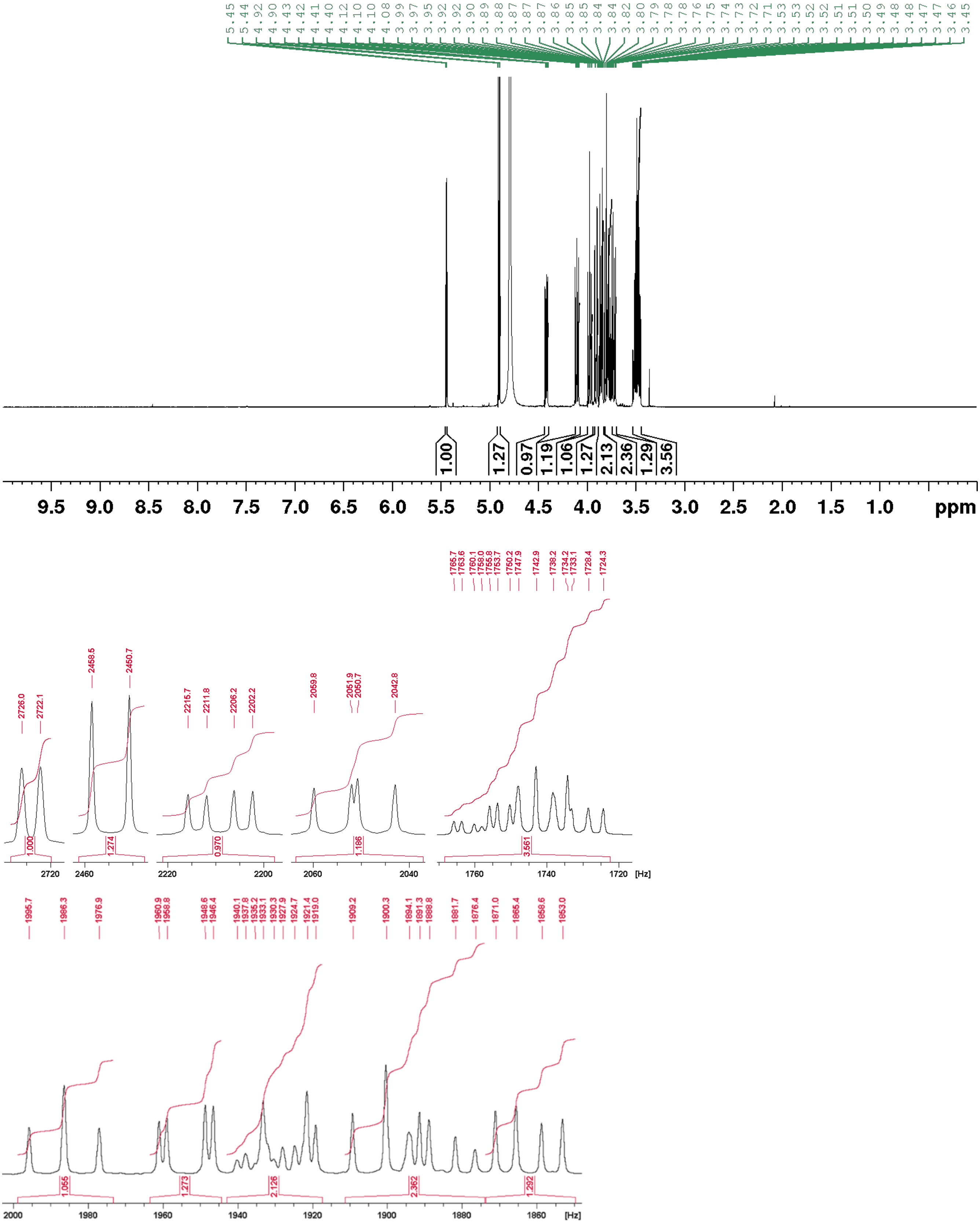

7.1.3 FDGlc-2 (**1**): ^{19}F{^{1}H} NMR (470 MHz, D_2O)

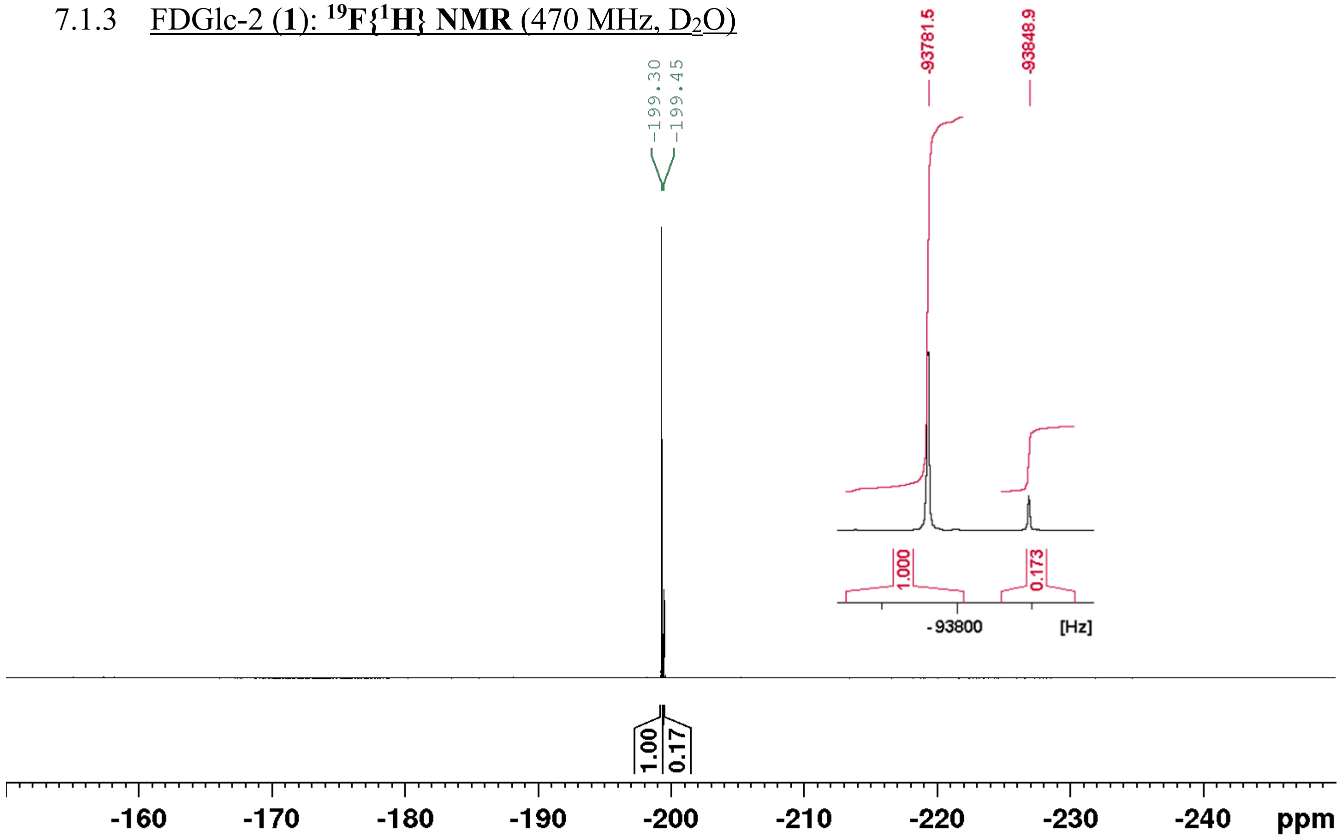

7.1.4 FDGlc-2 (**1**): ^{19}F NMR (470 MHz, D_2O)

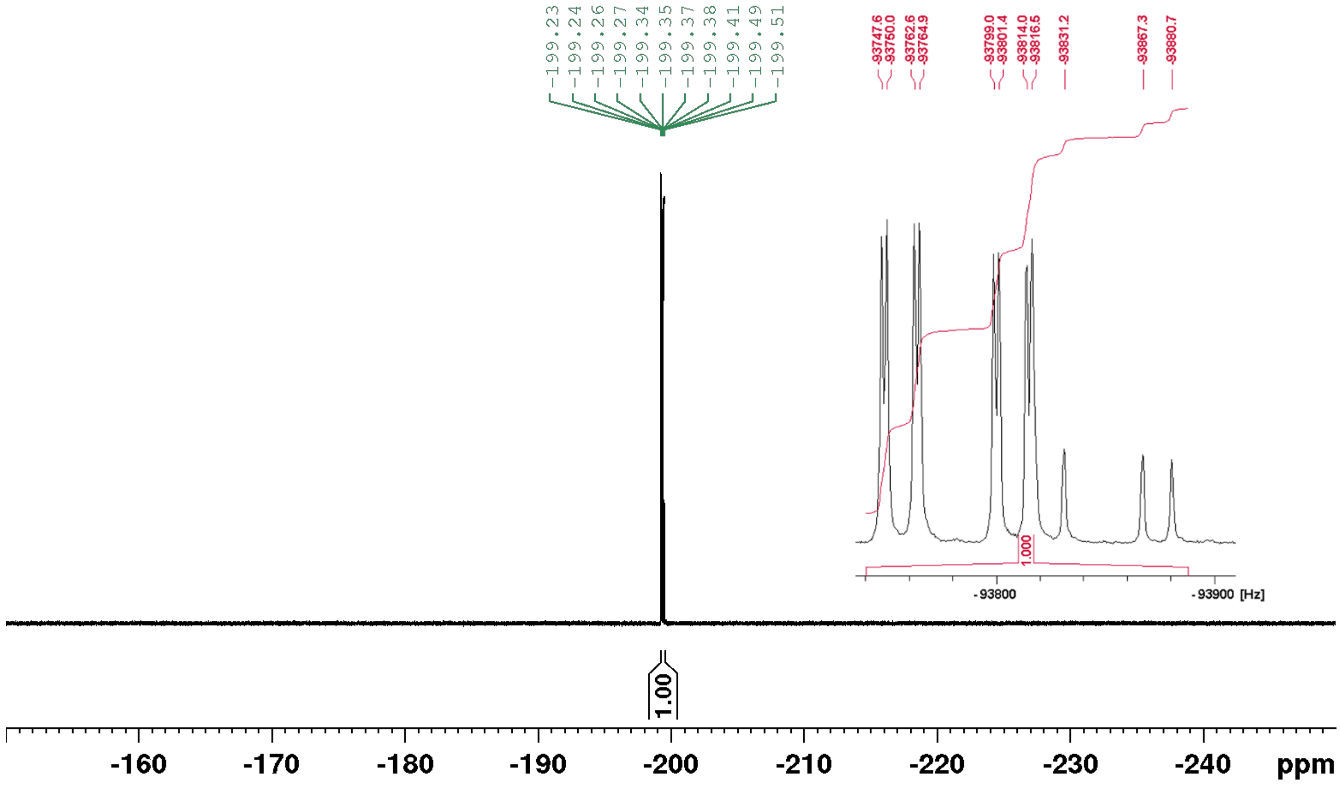

7.1.5 FDGlc-2 (**1**): **^{1}H-^{1}H COSY** (500 MHz, D_2O)

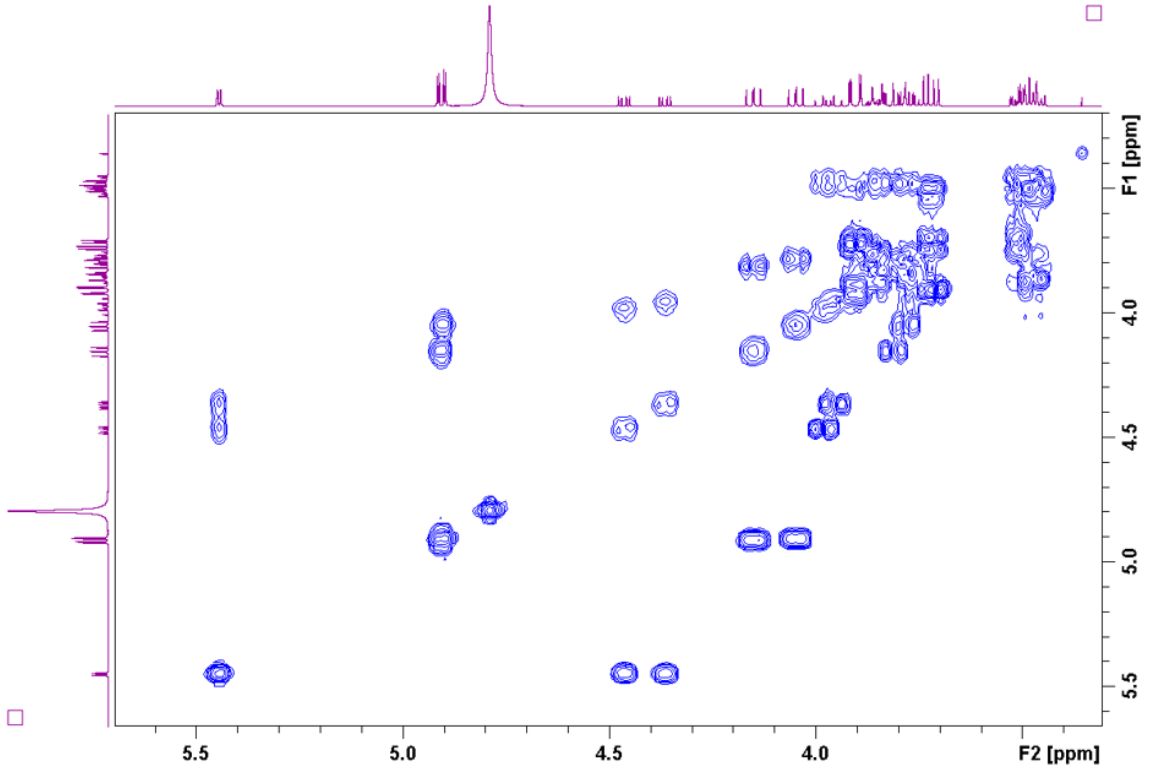

7.1.6 FDGlc-2 (**1**): **^{1}H-^{1}H{^{19}F} COSY** (500 MHz, D_2O)

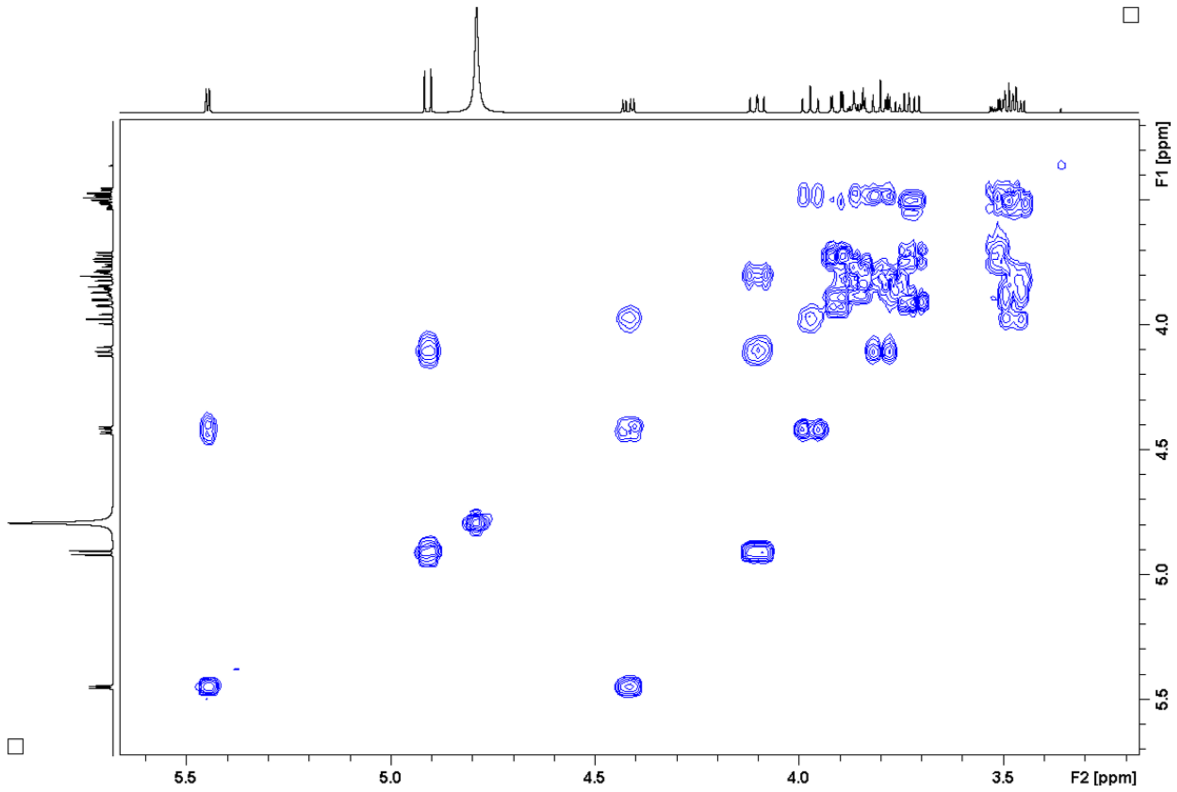

7.1.7 FDGlc-2 (**1**): α-pyranose form (α-*p*-FDGlc-2): **^{1}H{^{19}F} SRI-FESTA NMR** (500 MHz, D_2O, δ^{19}F = -199.45 ppm, δ^1H = 4.41 ppm, τ_m = 120 ms)

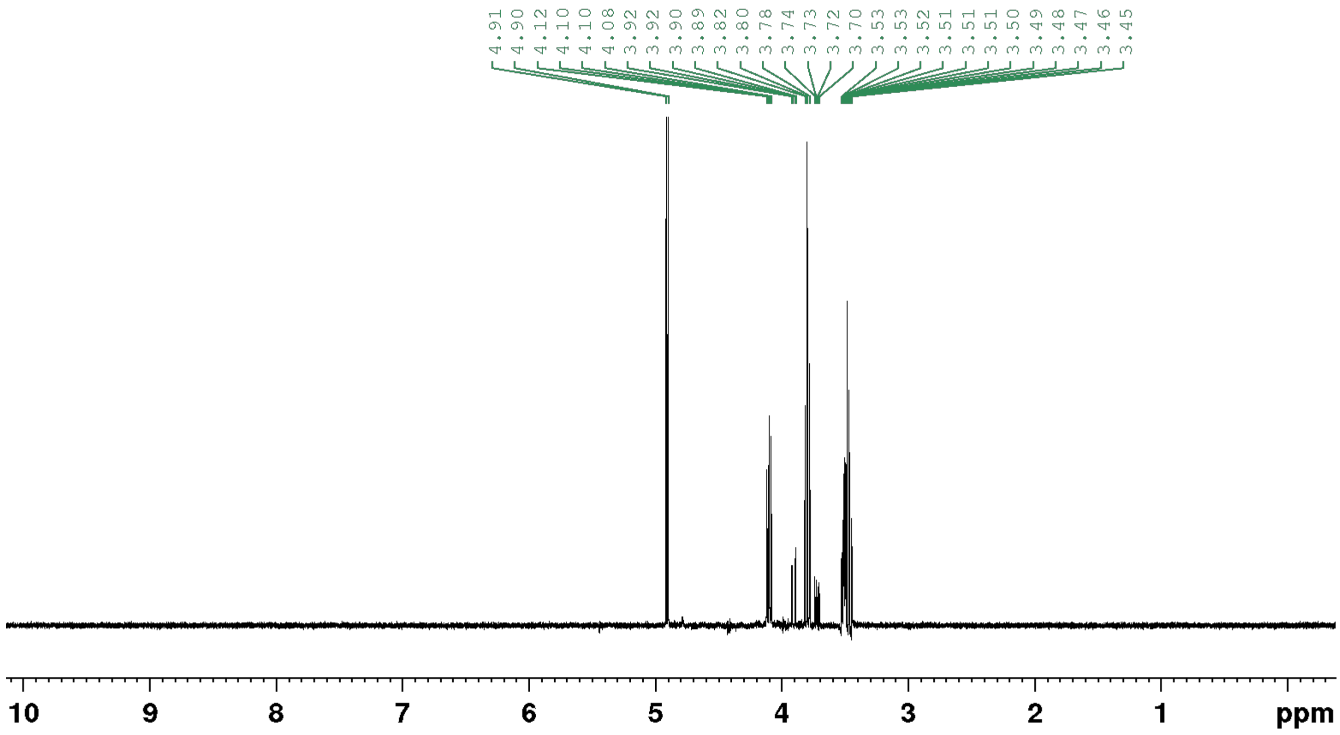

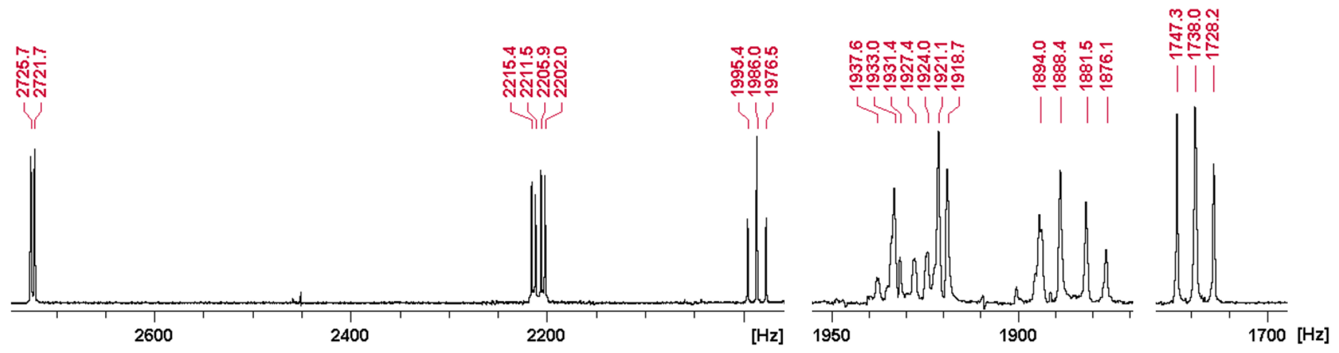

7.1.8 FDGlc-2 (**1**): β-pyranose form (β-*p*-FDGlc-2): **^{1}H{^{19}F} SRI-FESTA NMR** (500 MHz, D_2O, δ^{19}F = -199.30 ppm, δ^{1}H = 4.10 ppm, τ_m = 120 ms)

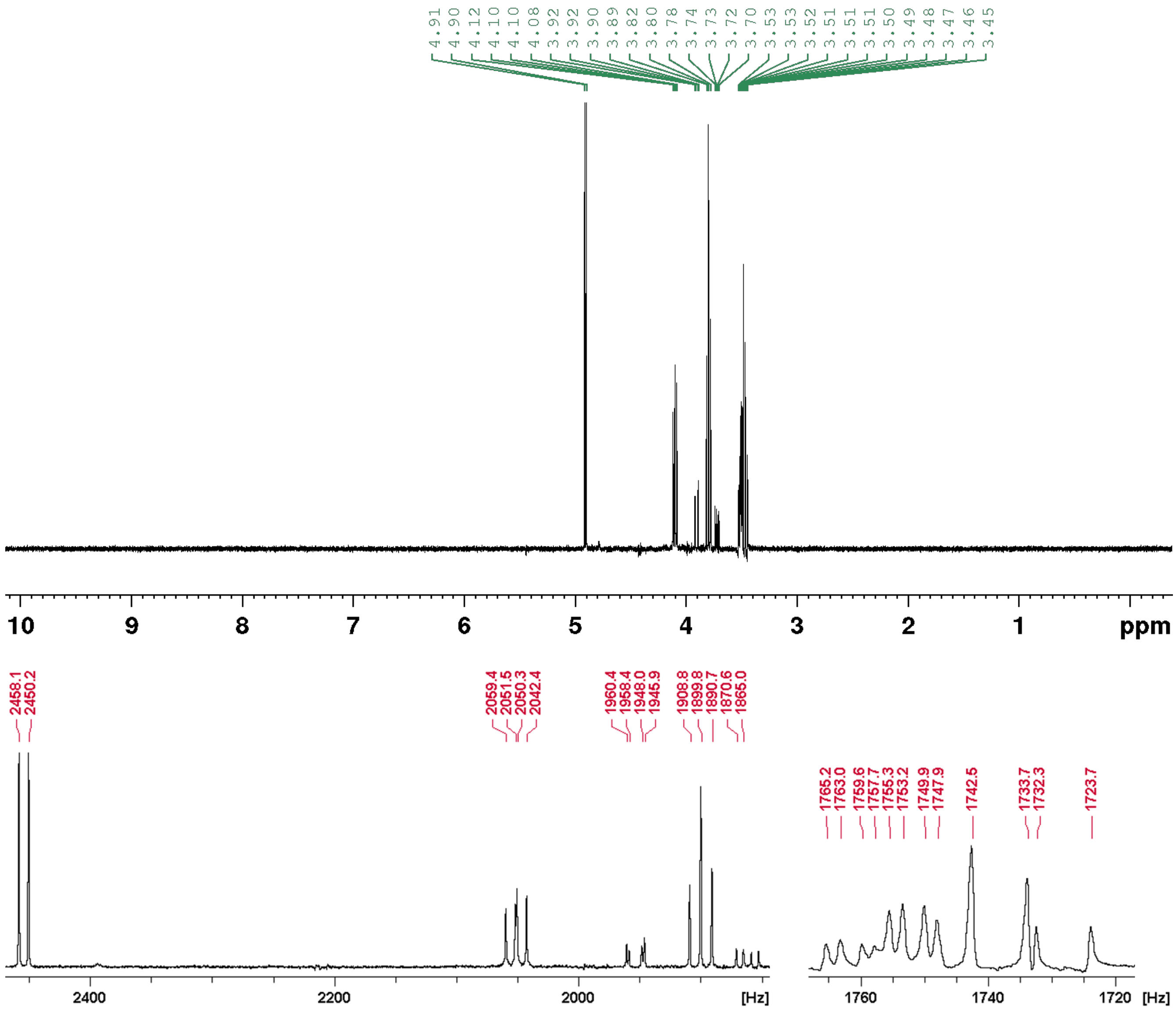

7.2 3-Deoxy-3-fluoro-D-glucose (2, FDGlc-3): 45 : 55 α-pyranose / β-pyranose, in D_2O.

7.2.1 FDGlc-3 (**2**): [1]**H NMR** (500 MHz, D_2O)

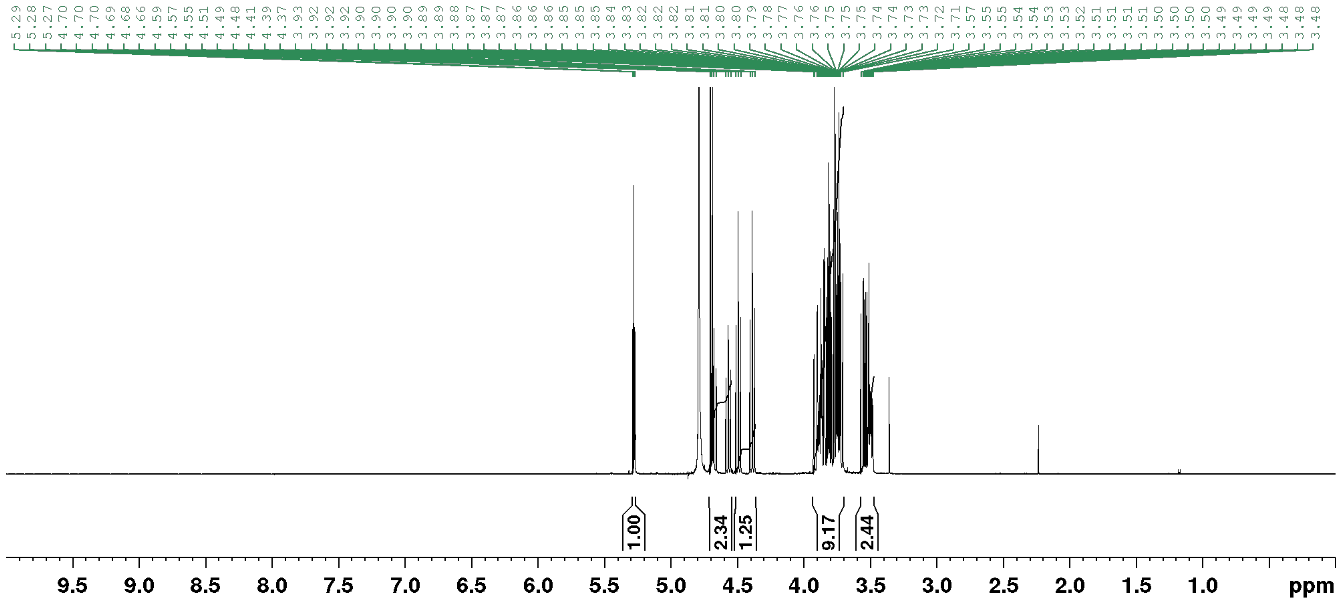

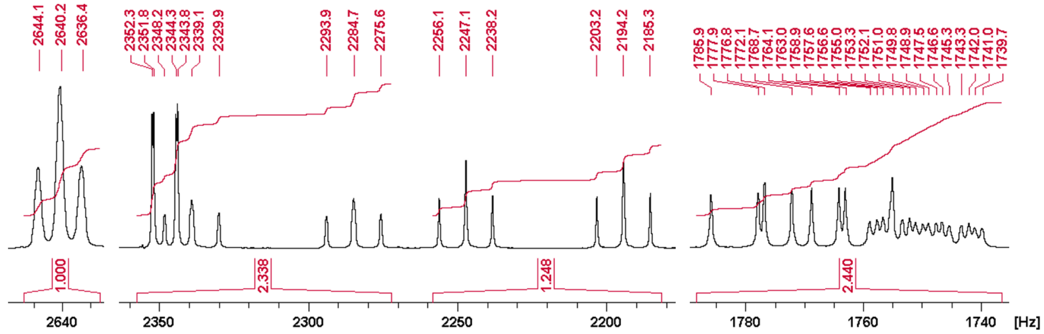

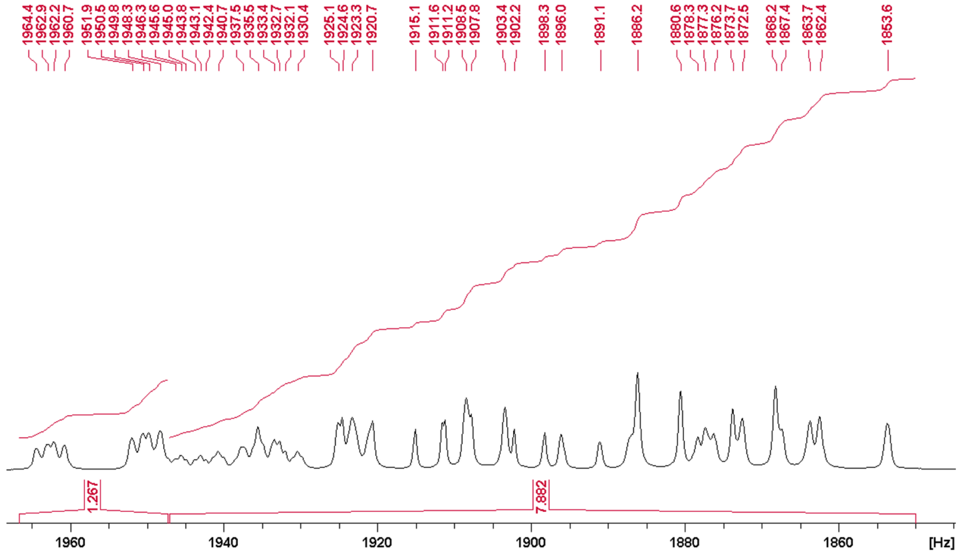

7.2.2 FDGlc-3 (**2**): **^{1}H{^{19}F} NMR** (500 MHz, D_2O)

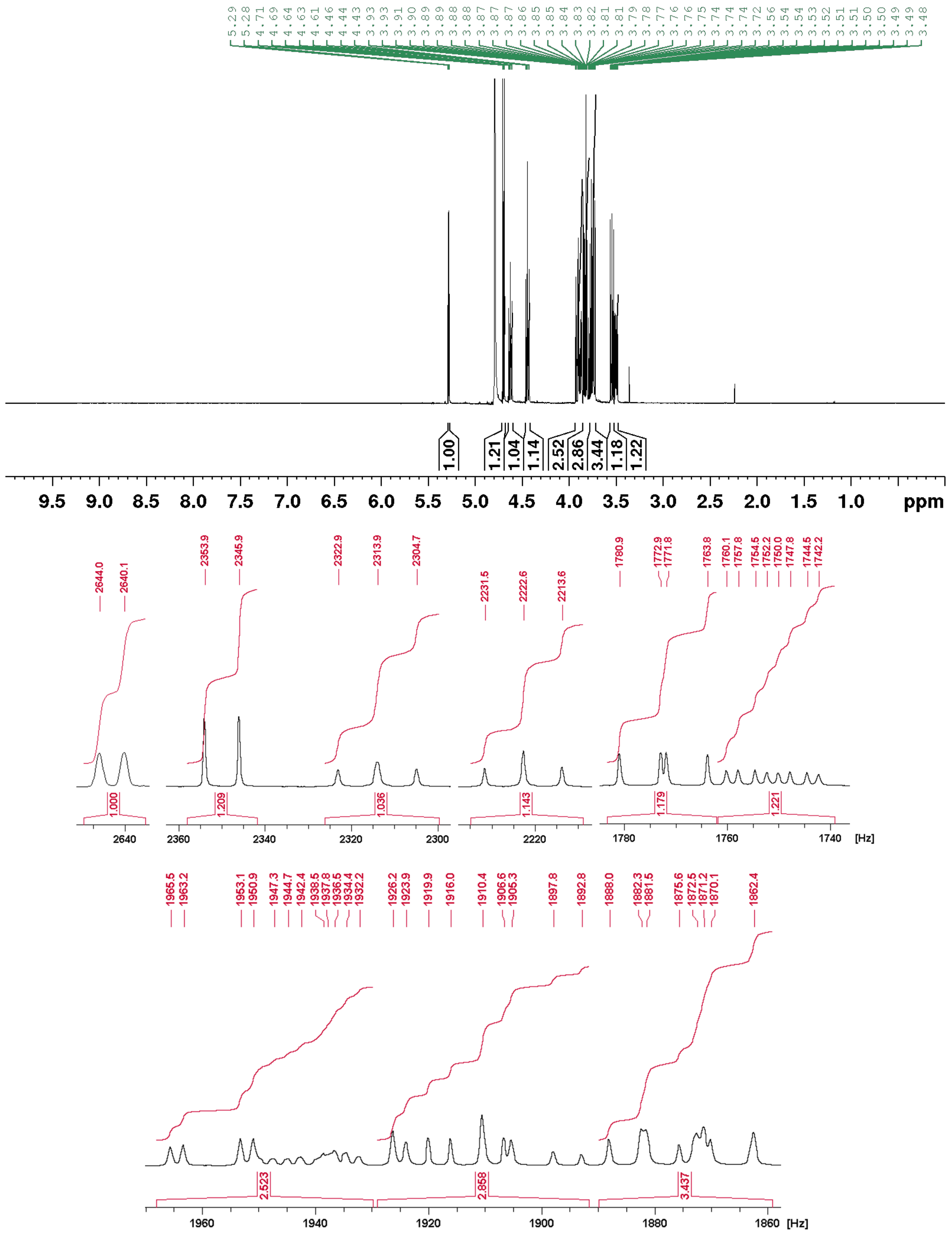

7.2.3 FDGlc-3 (**2**): **$^{19}F\{^1H\}$ NMR** (470 MHz, D_2O)

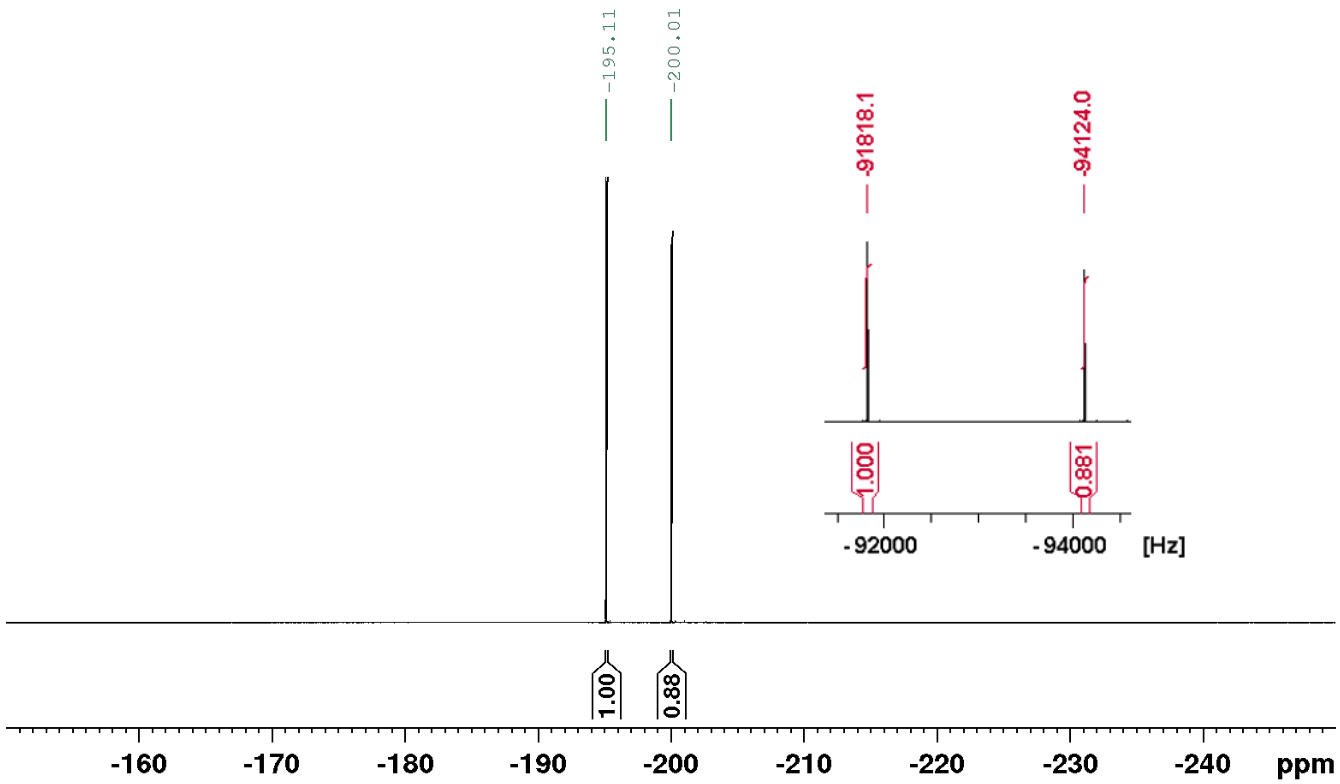

7.2.4 FDGlc-3 (**2**): **^{19}F NMR** (470 MHz, D_2O)

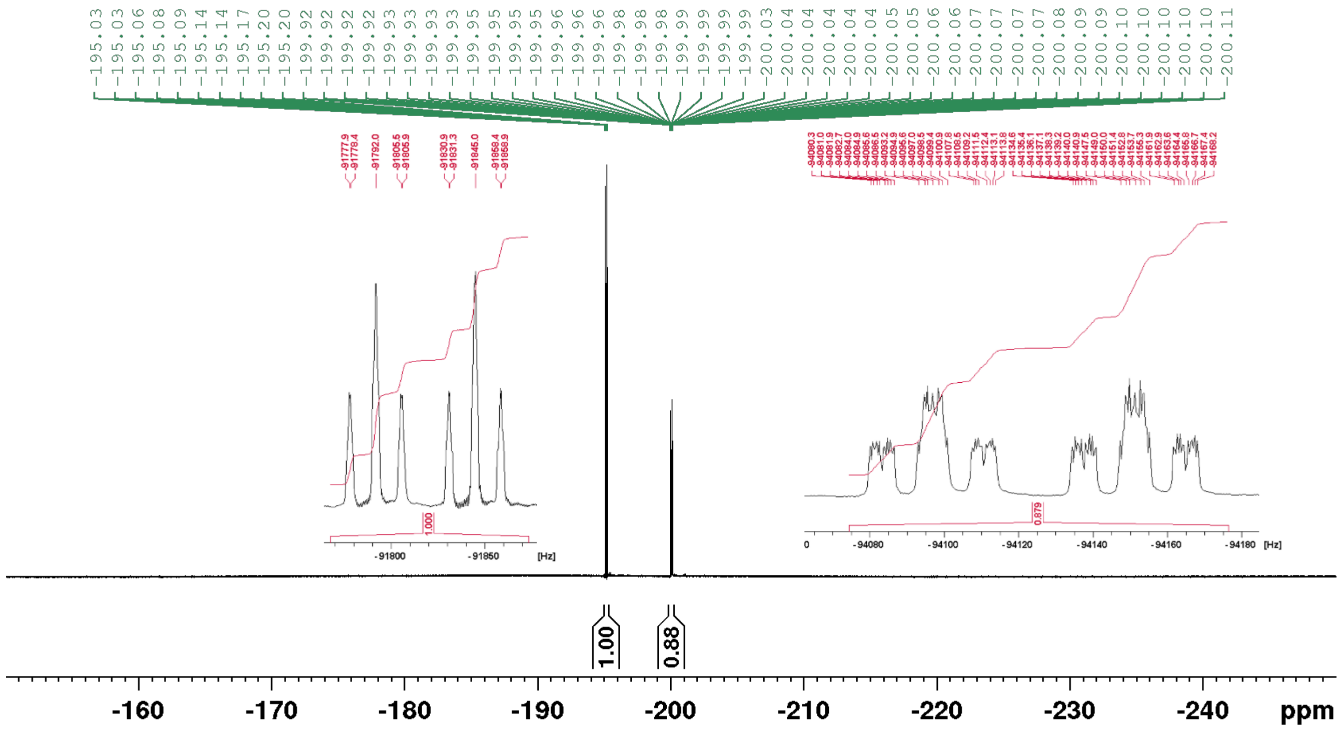

7.2.5 FDGlc-3 (**2**): **^{1}H-^{1}H COSY** (500 MHz, D_2O)

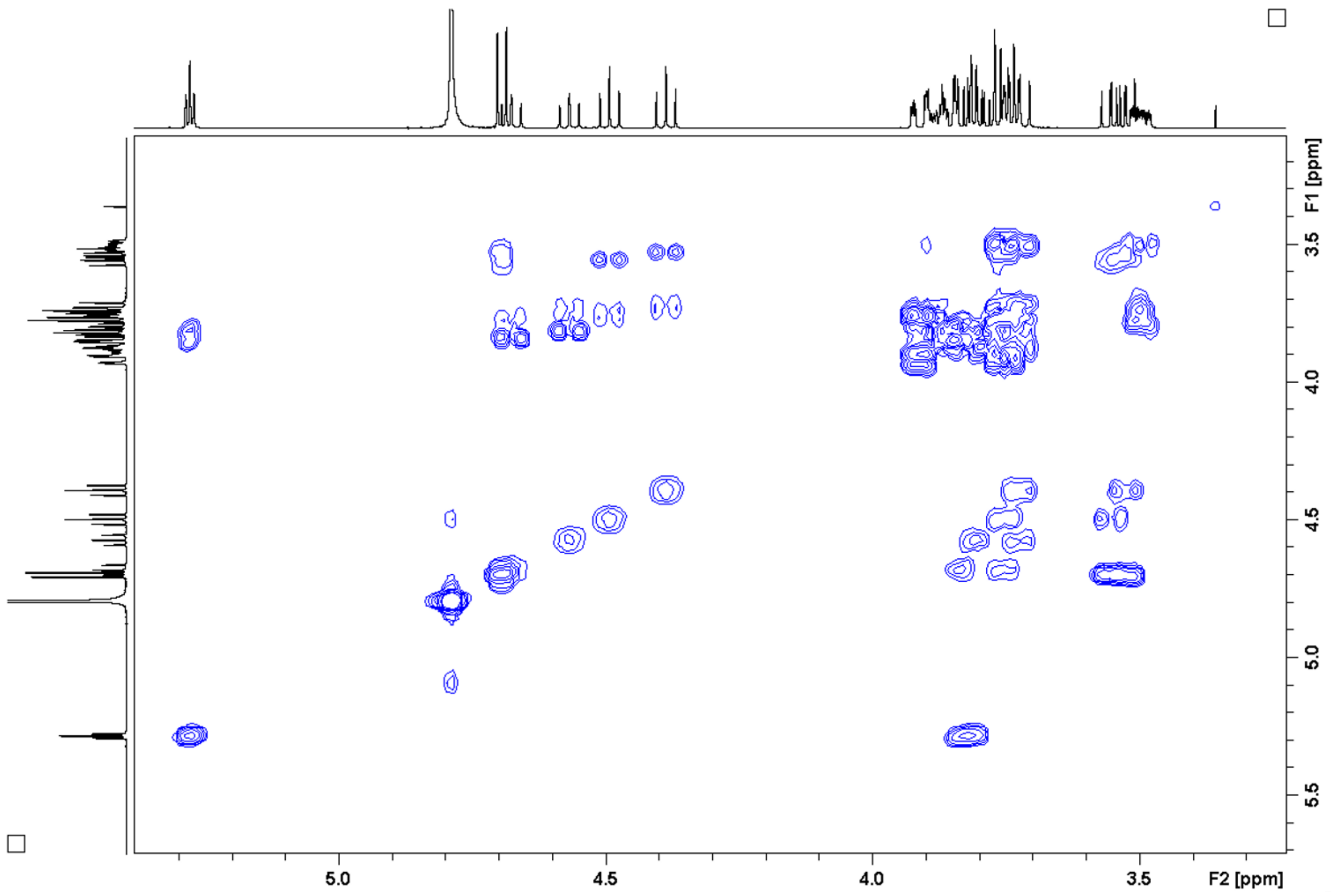

7.2.6 FDGlc-3 (**2**): α-pyranose form (α-*p*-FDGlc-3): **^{1}H{^{19}F} SRI-FESTA NMR** (500 MHz, D_2O, δ^{19}F = -200.01 ppm, δ^1H = 4.62 ppm, τ_m = 100 ms)

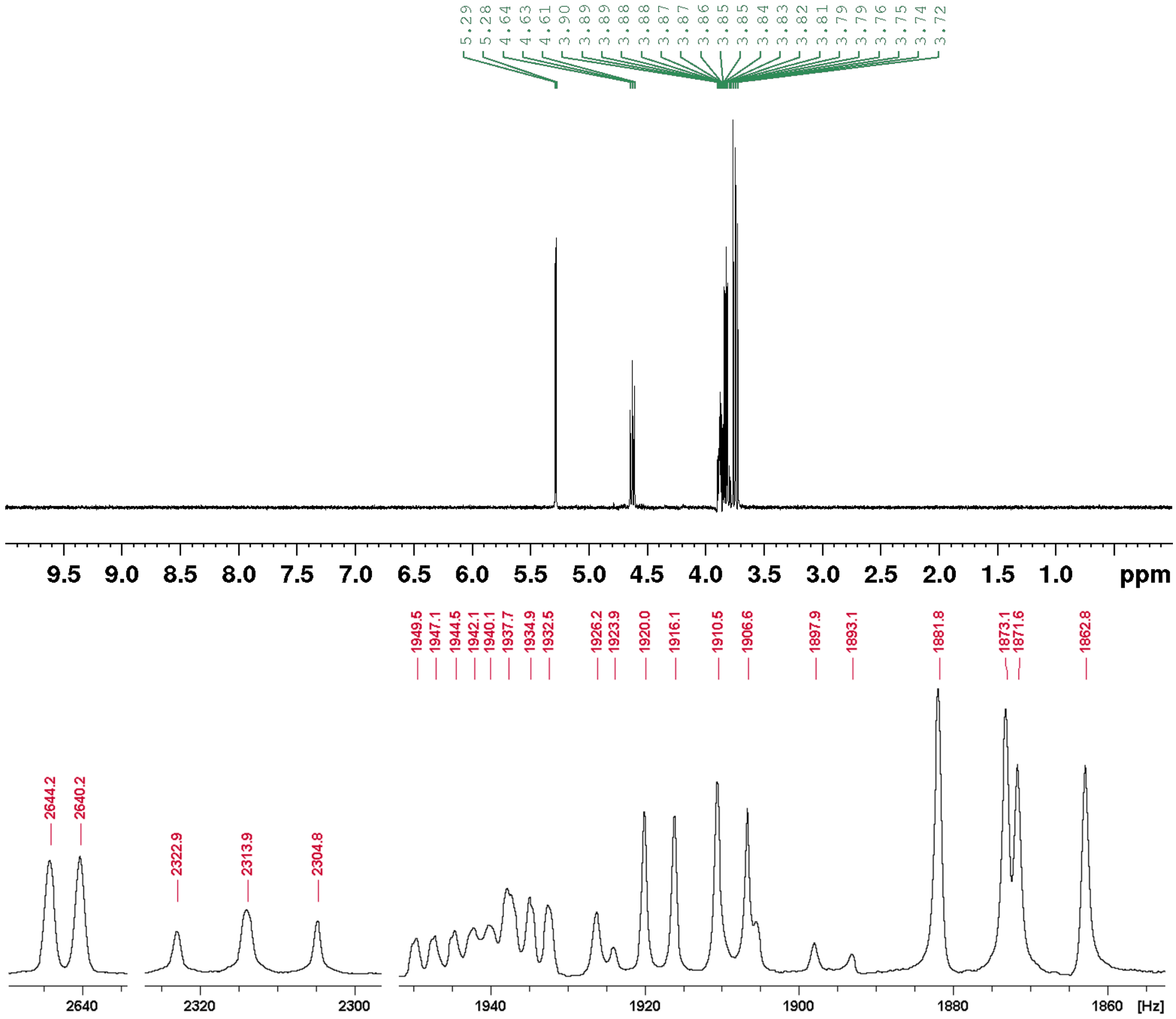

7.2.7 FDGlc-3 (**2**): β-pyranose form (β-*p*-FDGlc-3): **^{1}H{^{19}F} SRI-FESTA NMR** (500 MHz, D_2O, δ^{19}F = -195.11 ppm, δ^{1}H = 4.44 ppm, τ_m = 100 ms)

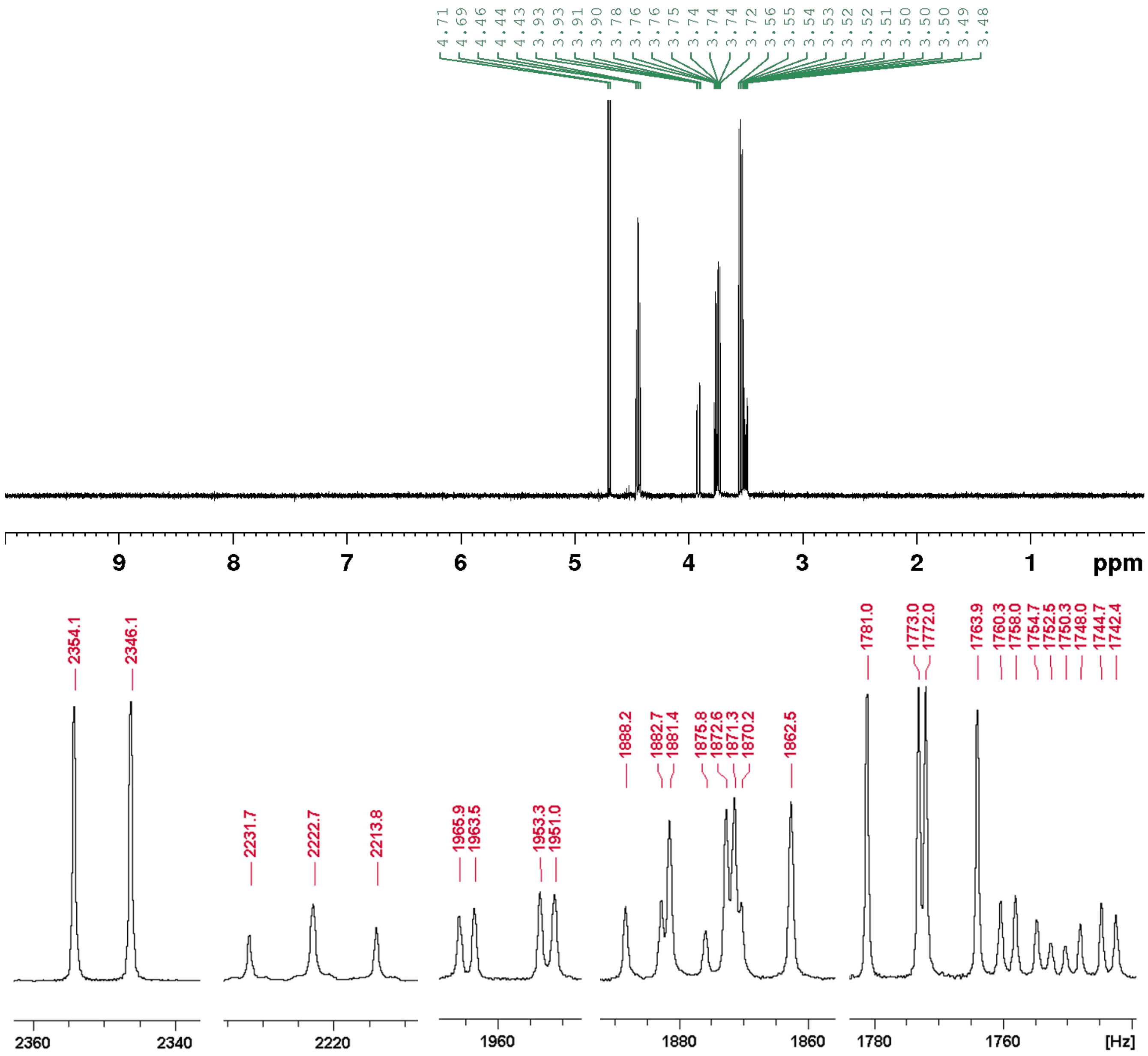

7.3 4-Deoxy-4-fluoro-D-glucose (3, FDGlc-4): 44 : 56 α-pyranose / β-pyranose, in D_2O.

7.3.1 FDGlc-4 (**3**): ^{1}**H NMR** (500 MHz, D_2O)

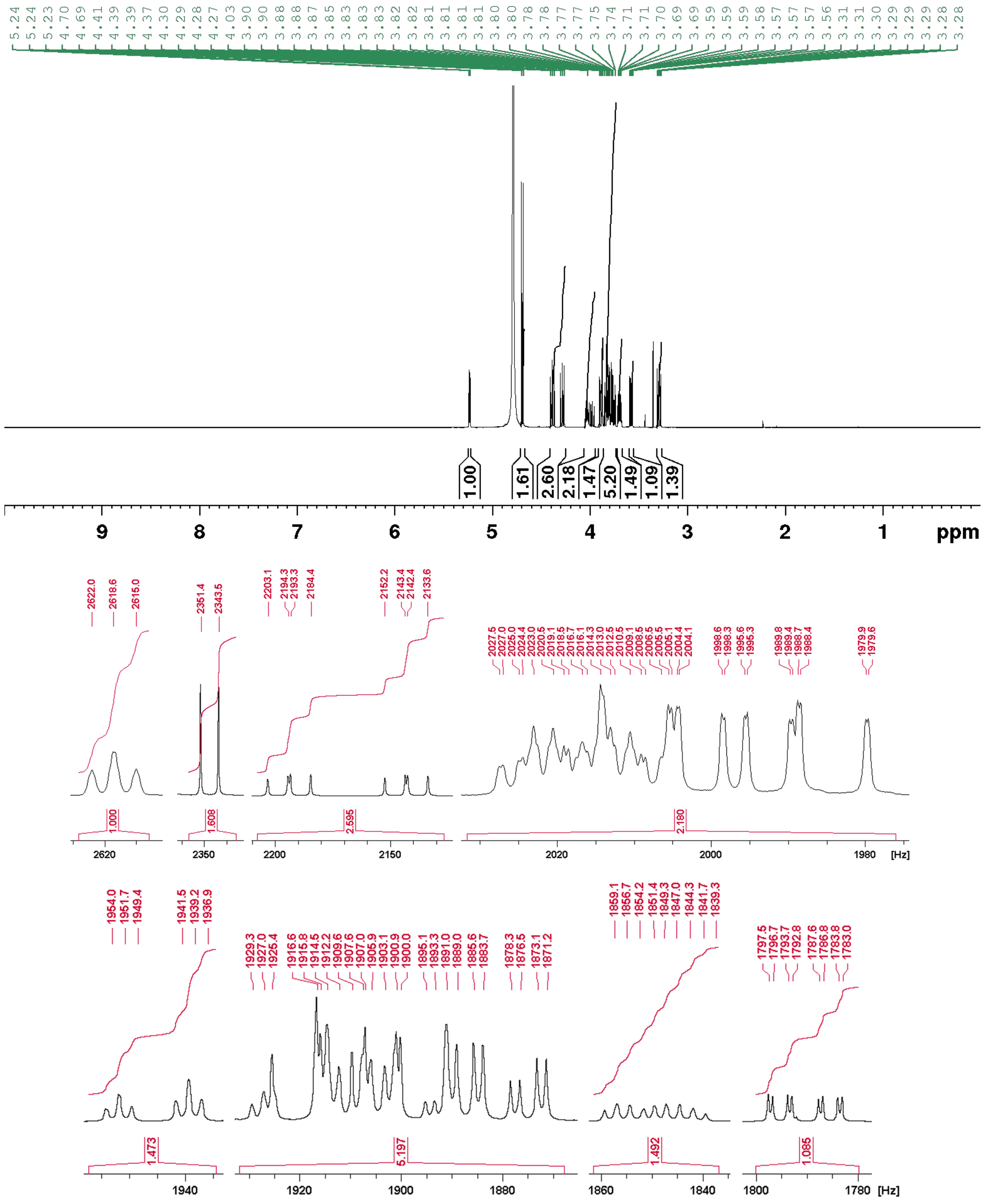

7.3.2 FDGlc-4 (**3**): **$^{1}H\{^{19}F\}$ NMR** (500 MHz, D_2O)

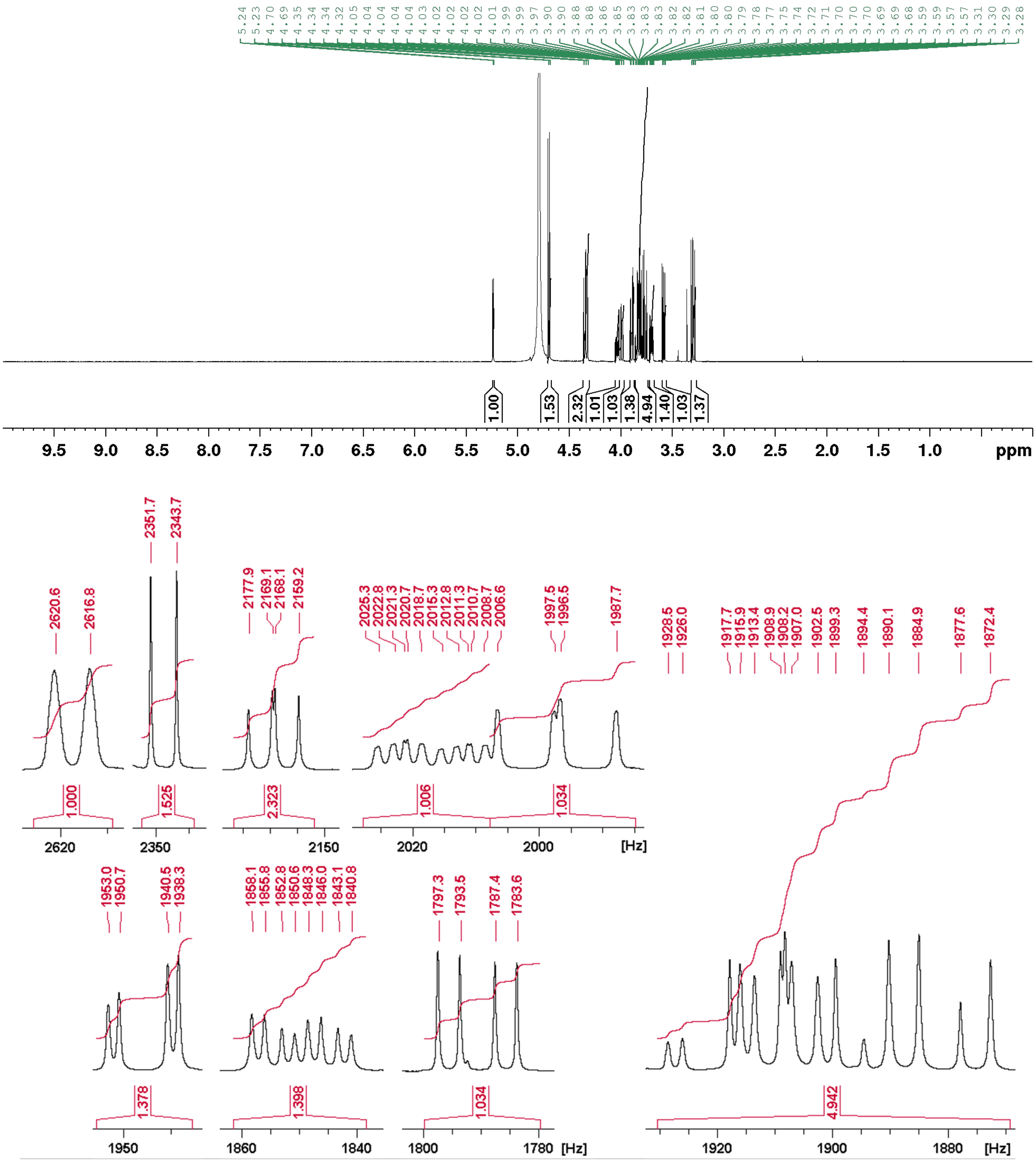

7.3.3 FDGlc-4 (**3**): ^{19}**F{^{1}H} NMR** (470 MHz, D_2O)

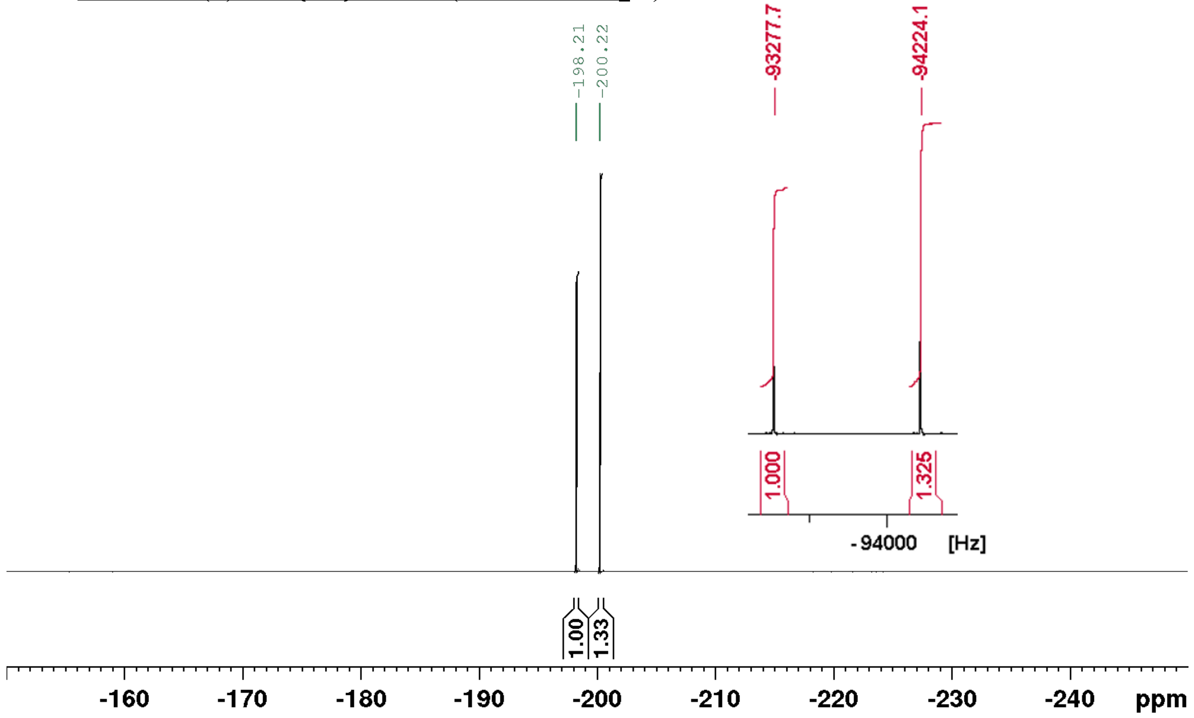

7.3.4 FDGlc-4 (**3**): ^{19}**F NMR** (470 MHz, D_2O)

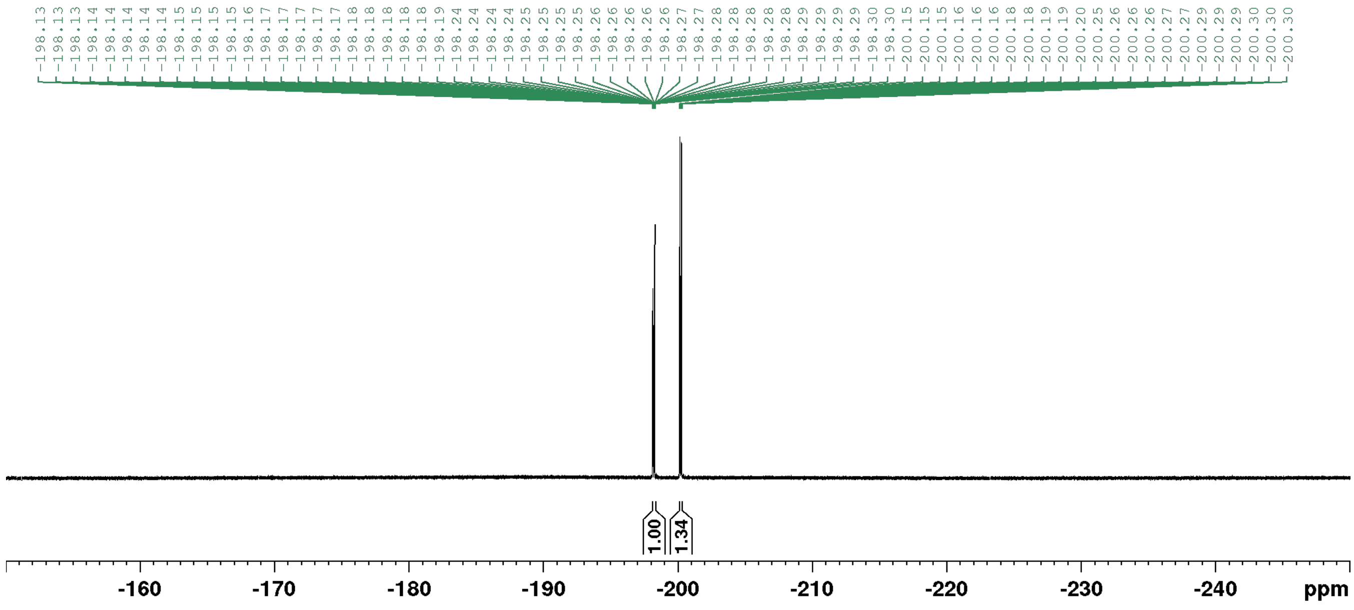

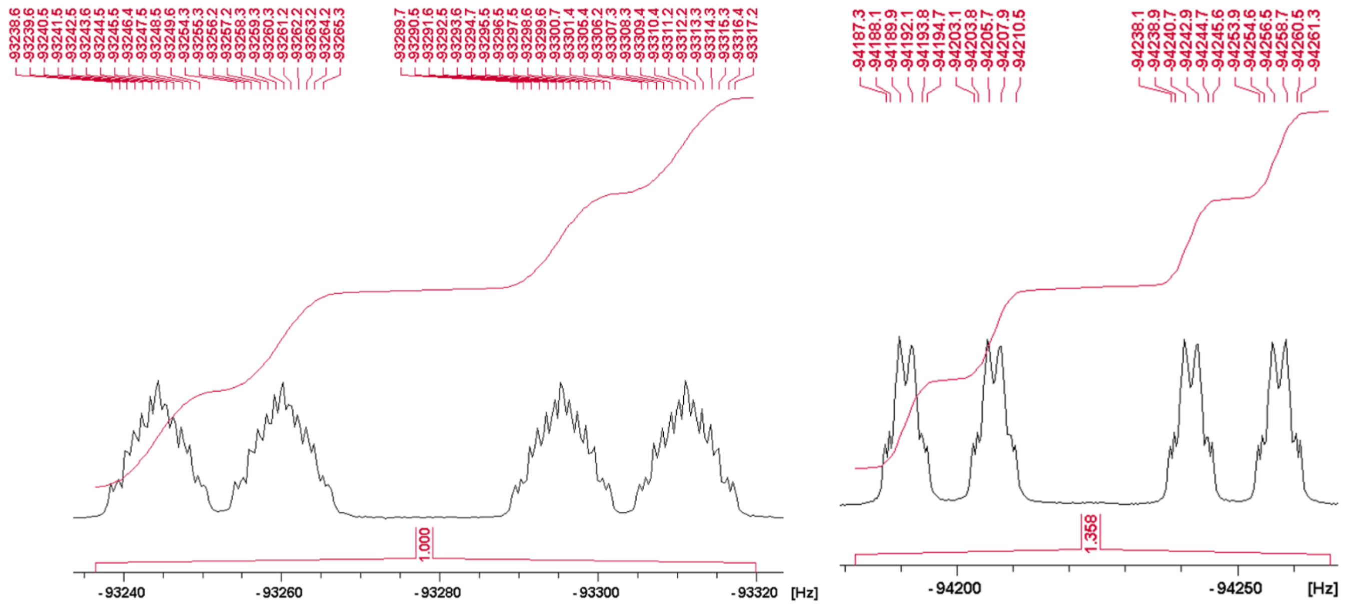

7.3.5 FDGlc-4 (**3**): **^{1}H-^{1}H COSY** (500 MHz, D_2O)

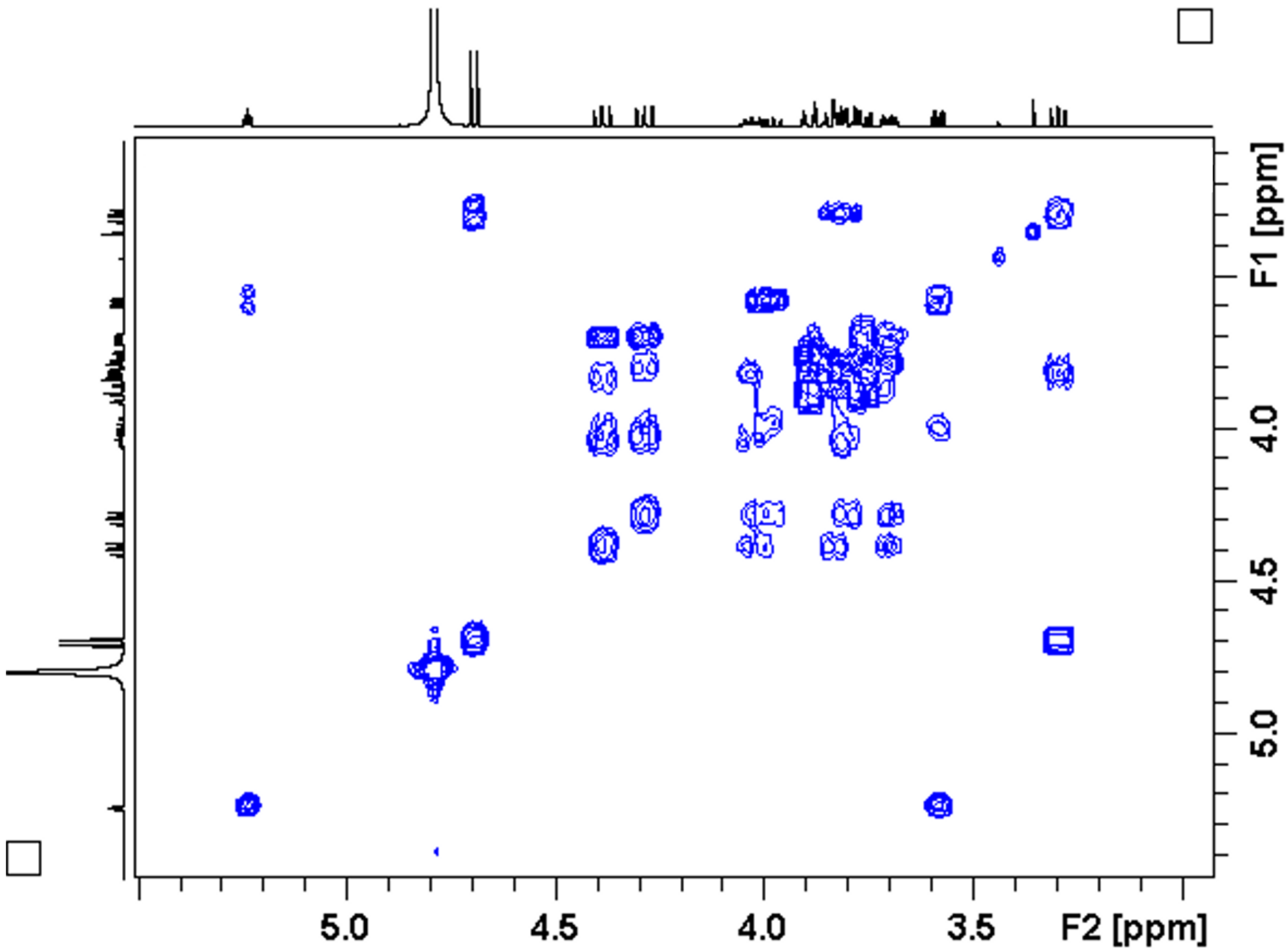

7.3.6 FDGlc-4 (**3**): α-pyranose form (α-*p*-FDGlc-4): **^{1}H{^{19}F} SRI-FESTA NMR** (500 MHz, D_2O, δ^{19}F = -198.21 ppm, δ^1H = 5.24 ppm, τ_m = 120 ms)

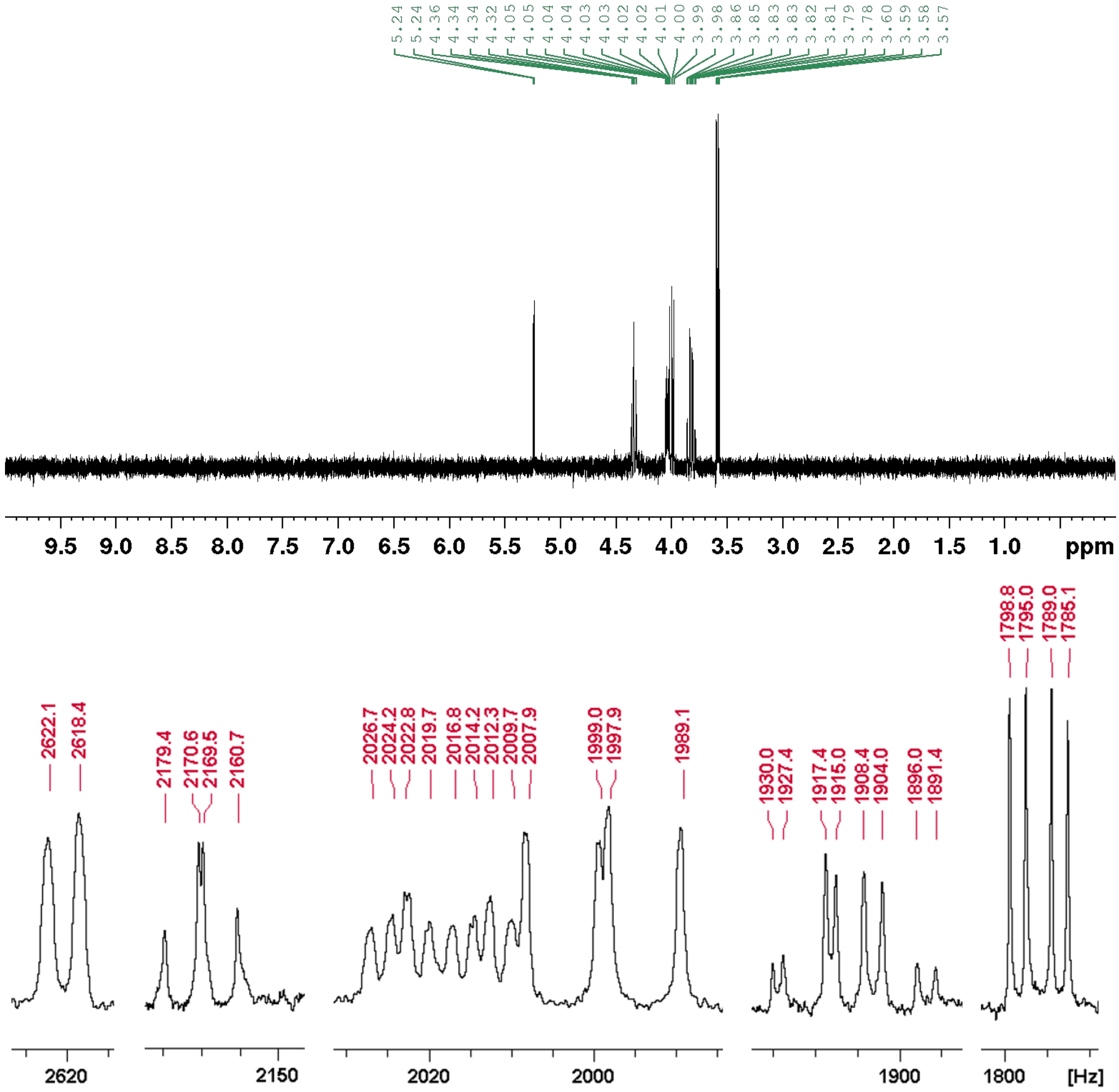

7.3.7 FDGlc-4 (**3**): β-pyranose form (β-*p*-FDGlc-4): **^{1}H{^{19}F} SRI-FESTA NMR** (500 MHz, D_2O, δ^{19}F = -200.22 ppm, δ^{1}H = 3.30 ppm, τ_m = 80 ms)

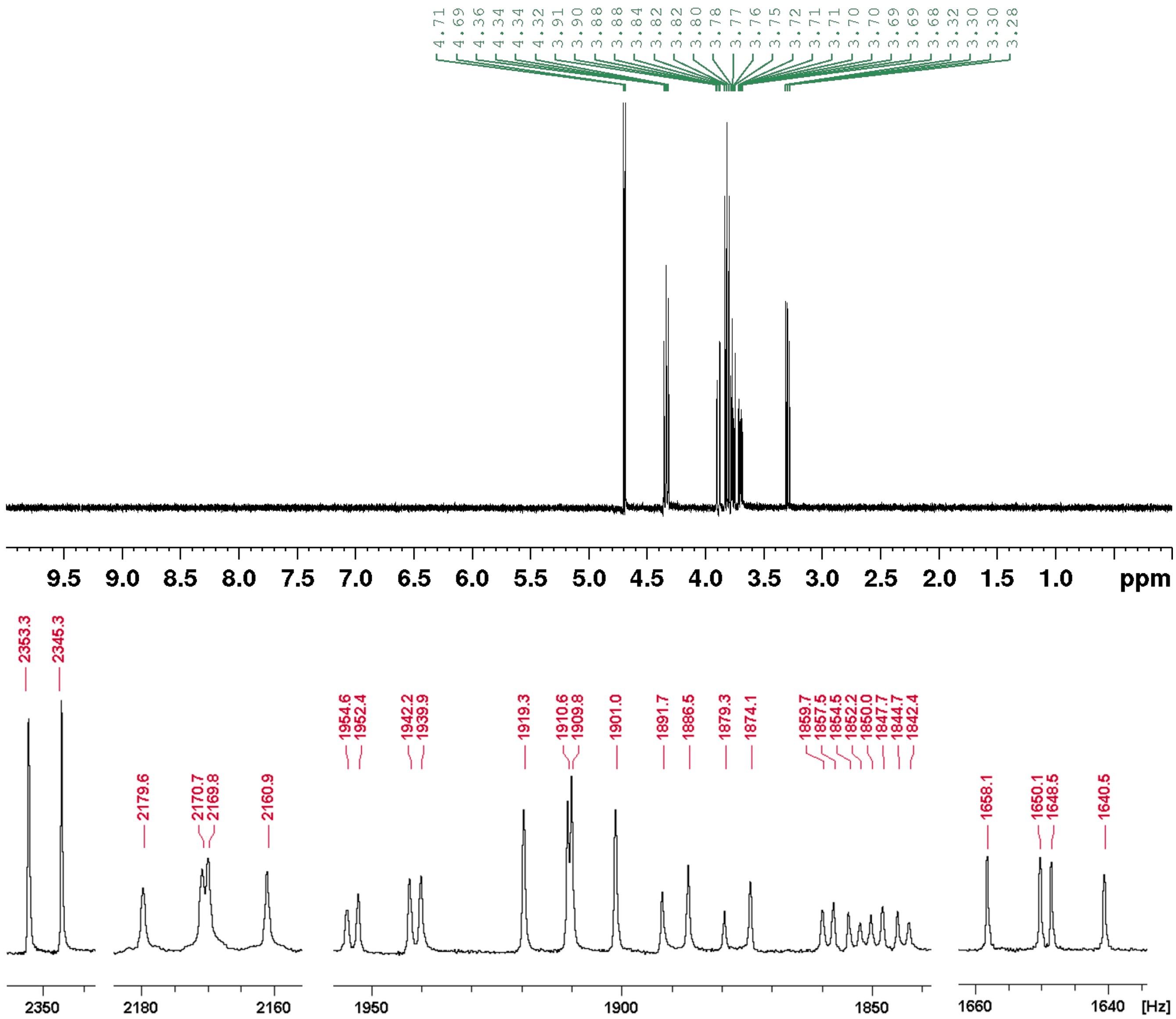

7.4 6-Deoxy-6-fluoro-D-glucose (4, FDGlc-6): 42 : 58 α-pyranose / β-pyranose, in D_2O.

7.4.1 FDGlc-6 (**4**): ^{1}**H NMR** (600 MHz, D_2O)

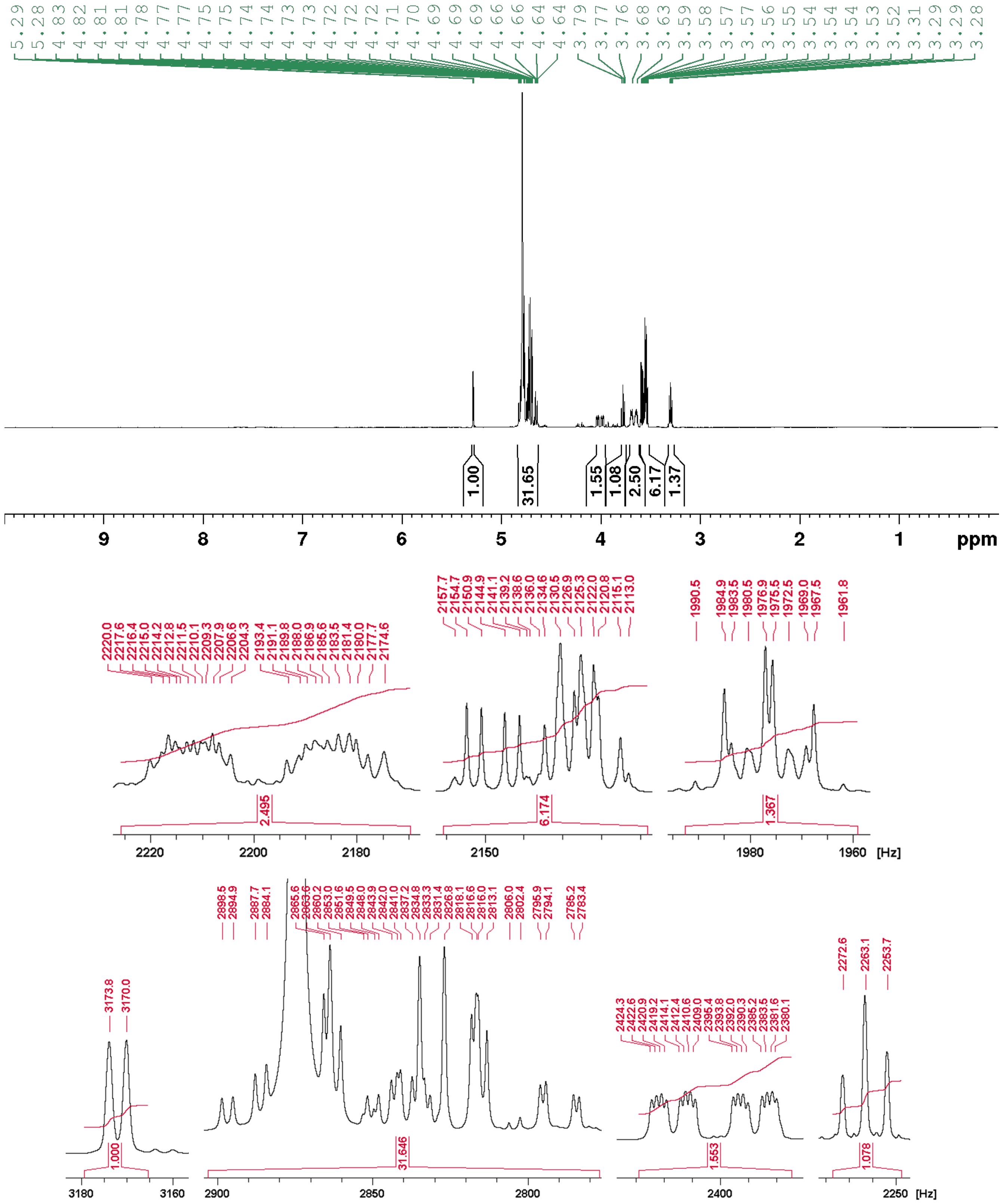

7.4.2 FDGlc-6 (**4**): **^{1}H{^{19}F} NMR** (600 MHz, D_2O)

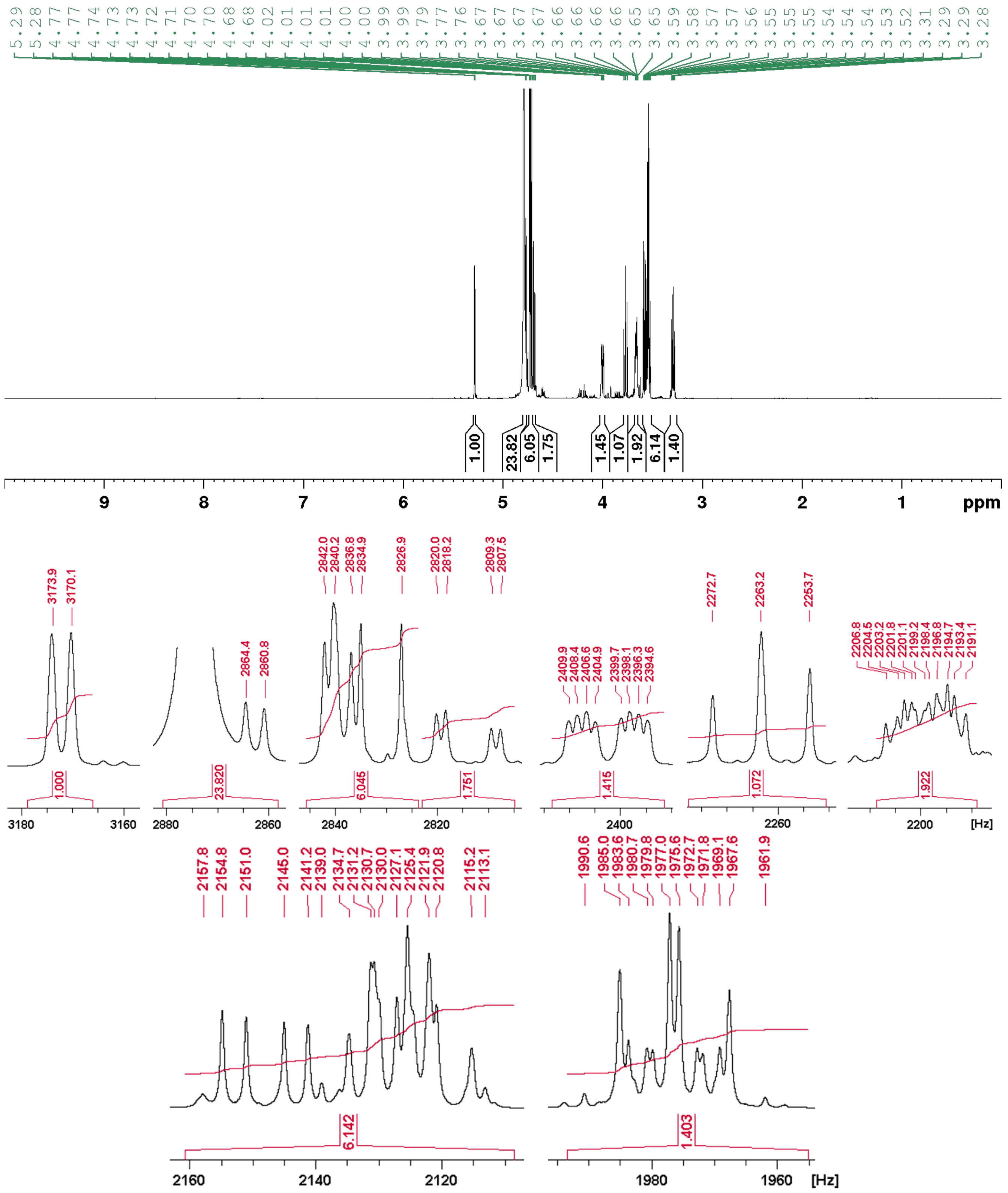

7.4.3 FDGlc-6 (**4**): ^{19}**F{^{1}H} NMR** (565 MHz, D_2O)

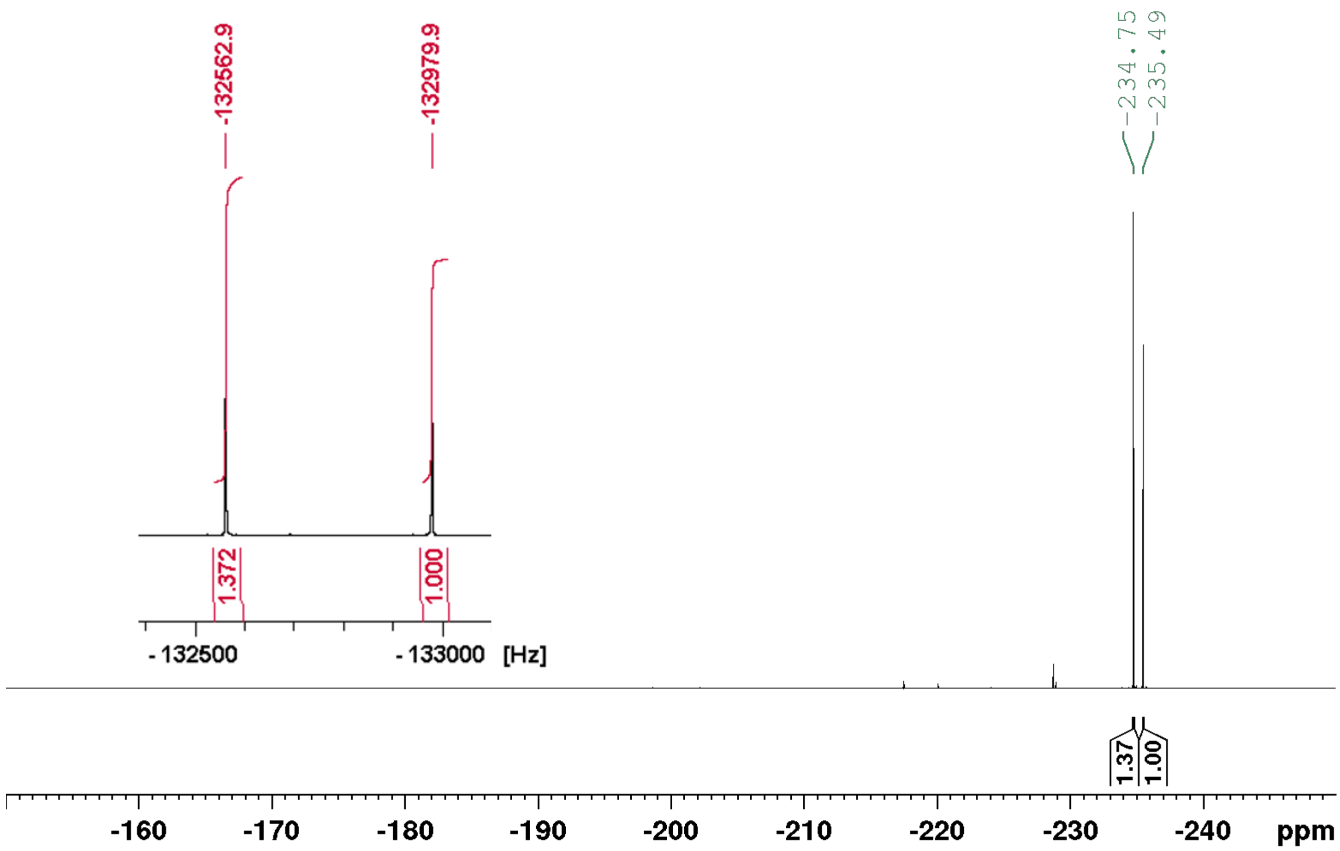

7.4.4 FDGlc-6 (**4**): ^{19}**F NMR** (565 MHz, D_2O)

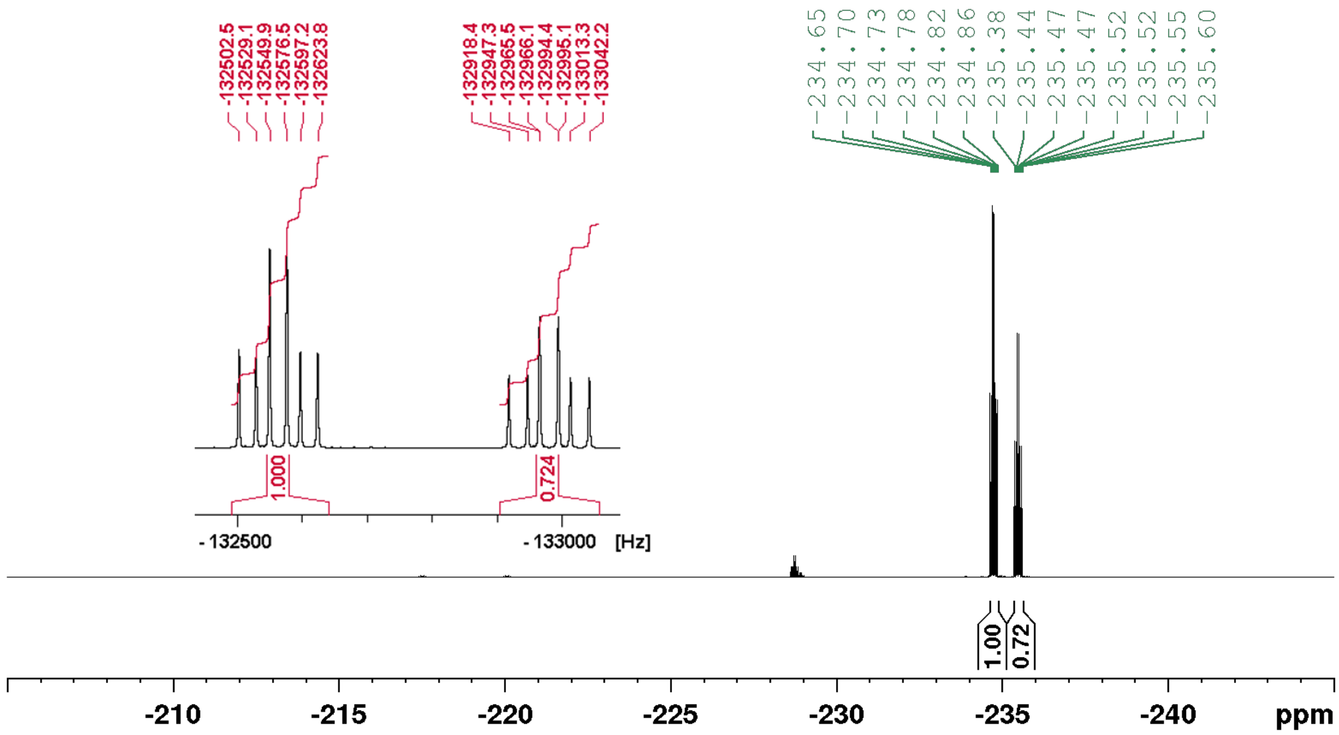

7.4.5 FDGlc-6 (**4**): **^{1}H-$^{1}H\{^{19}F\}$ COSY** (600 MHz, D_2O)

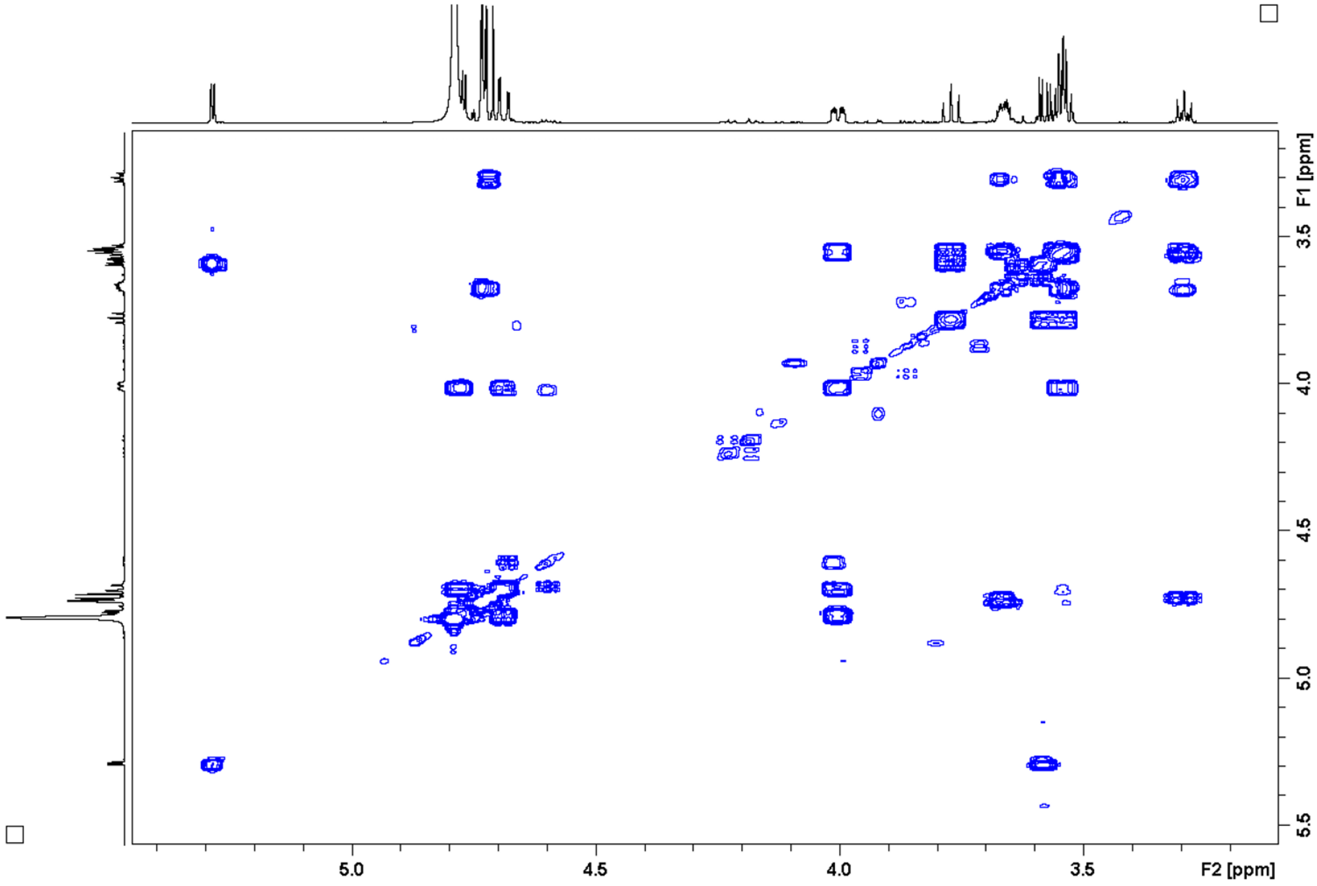

7.4.6 FDGlc-6 (**4**): α-pyranose form (α-*p*-FDGlc-4): **^{1}H{^{19}F} SRI-FESTA NMR** (600 MHz, D_2O, $\delta^{19}F = -235.49$ ppm, $\delta^{1}H = 4.75$ ppm, $\tau_m = 200$ ms)

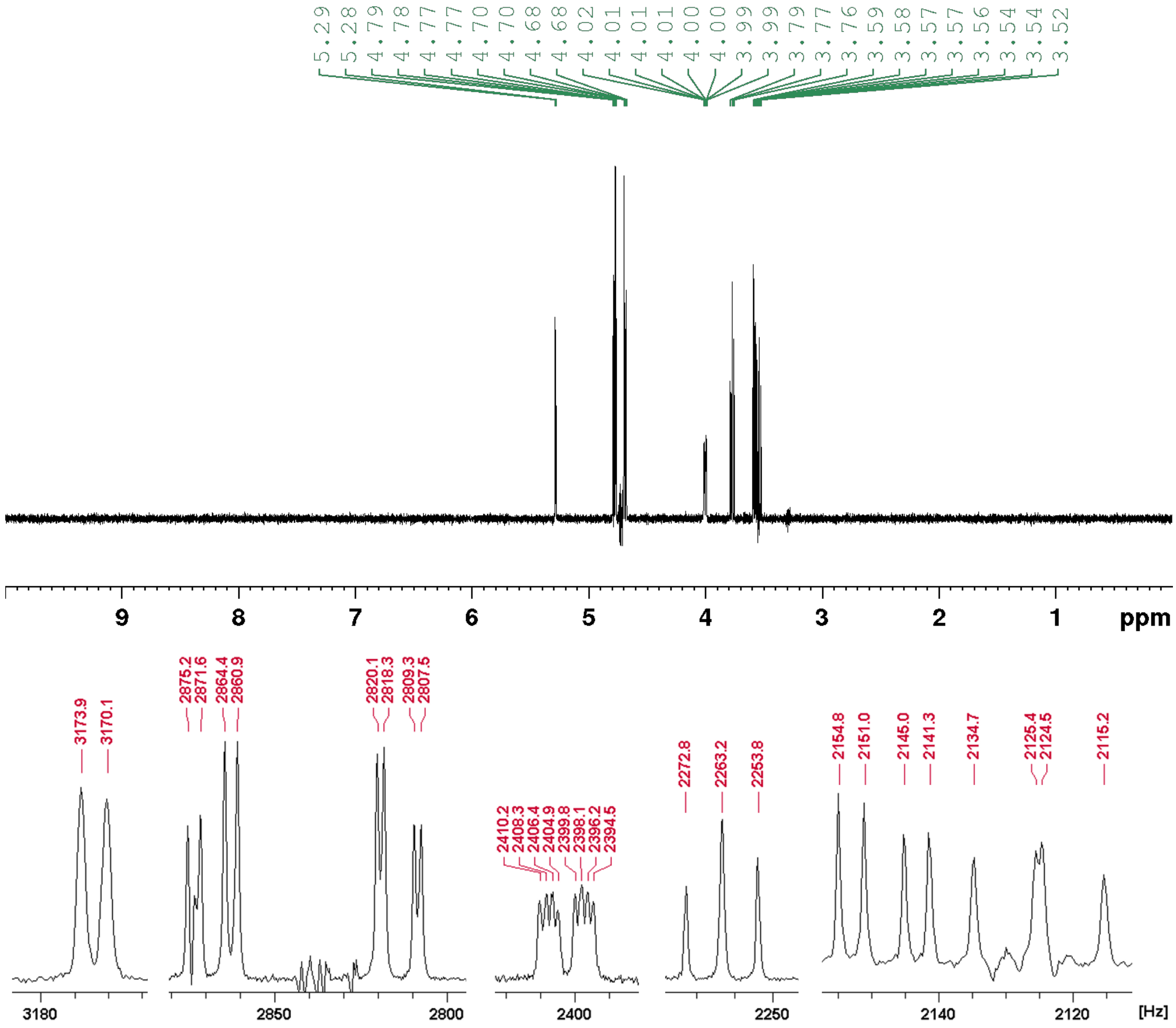

7.4.7 FDGlc-6 (**4**): β-pyranose form (β-*p*-FDGlc-4): **^{1}H{^{19}F} SRI-FESTA NMR** (600 MHz, D_2O, δ^{19}F = -234.75 ppm, δ^{1}H = 3.66 ppm, τ_m = 200 ms)

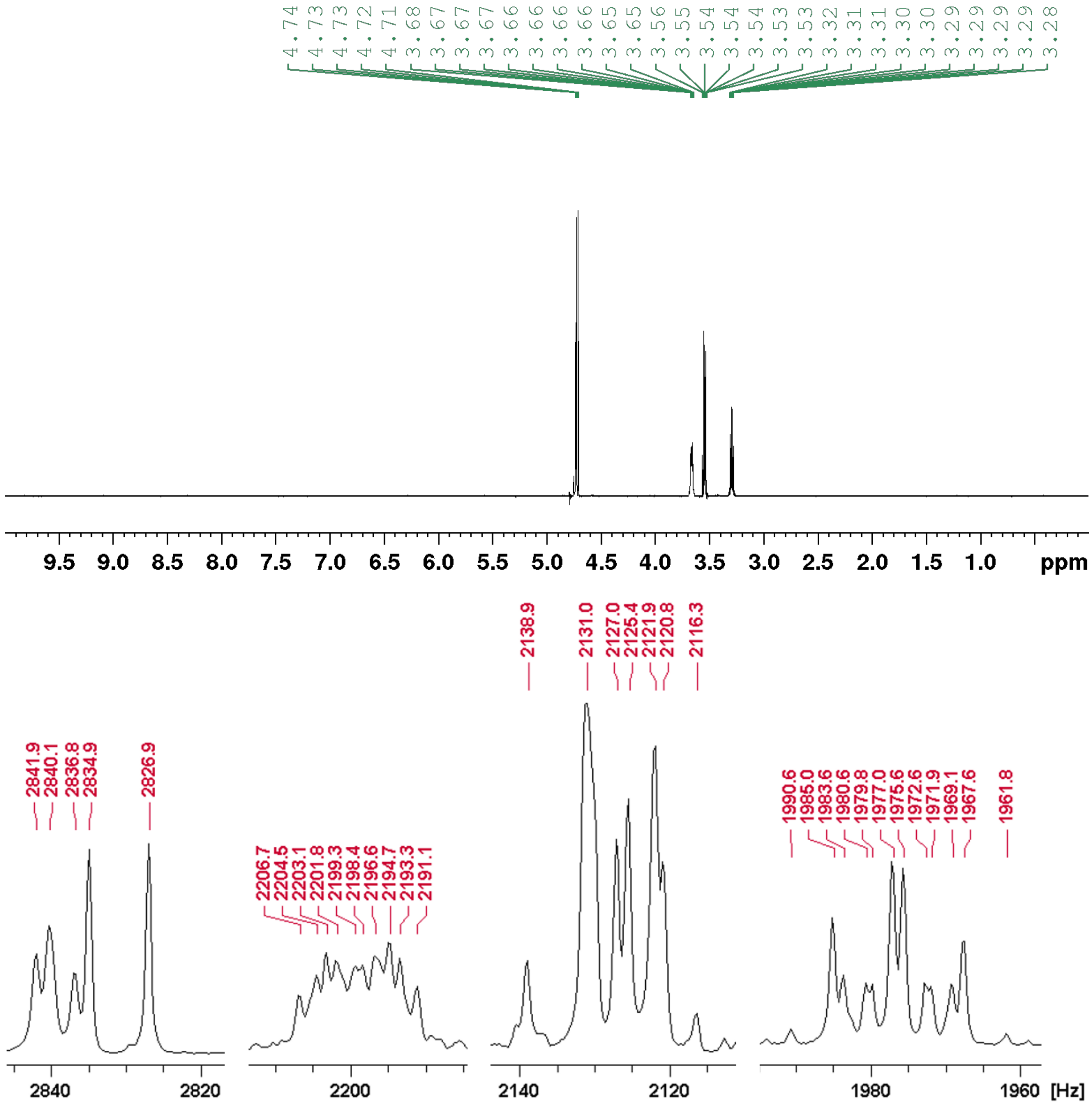

7.5 4,6-Dideoxy-4,6-difluoro-D-glucose (5, FDGlc-46): 45 : 55 α-pyranose / β-pyranose, in D_2O.

7.5.1 FDGlc-46 (**5**): ^{1}**H NMR** (500 MHz, D_2O)

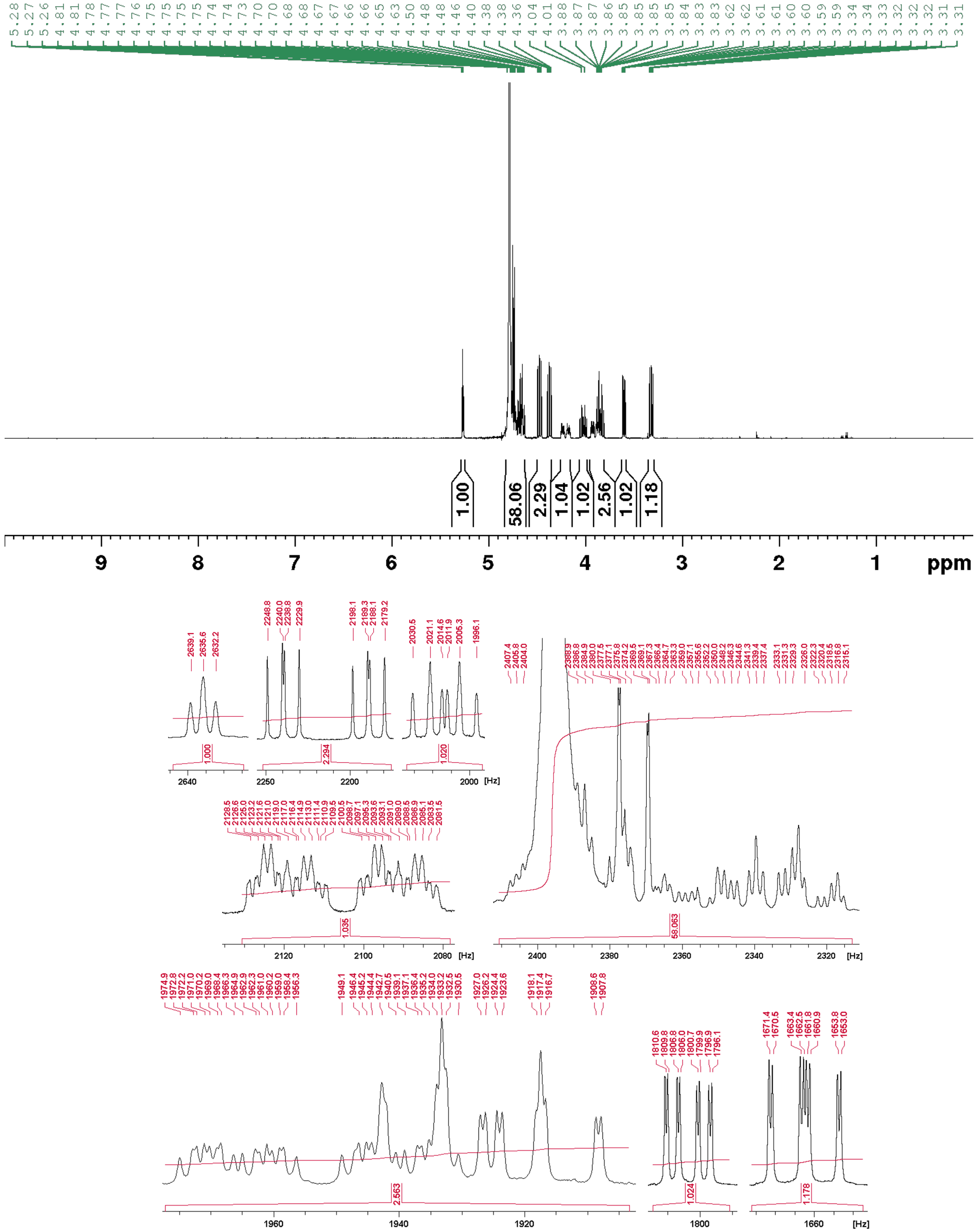

7.5.2 FDGlc-46 (**5**): ^{1}**H{**^{19}**F} NMR** (500 MHz, D_2O)

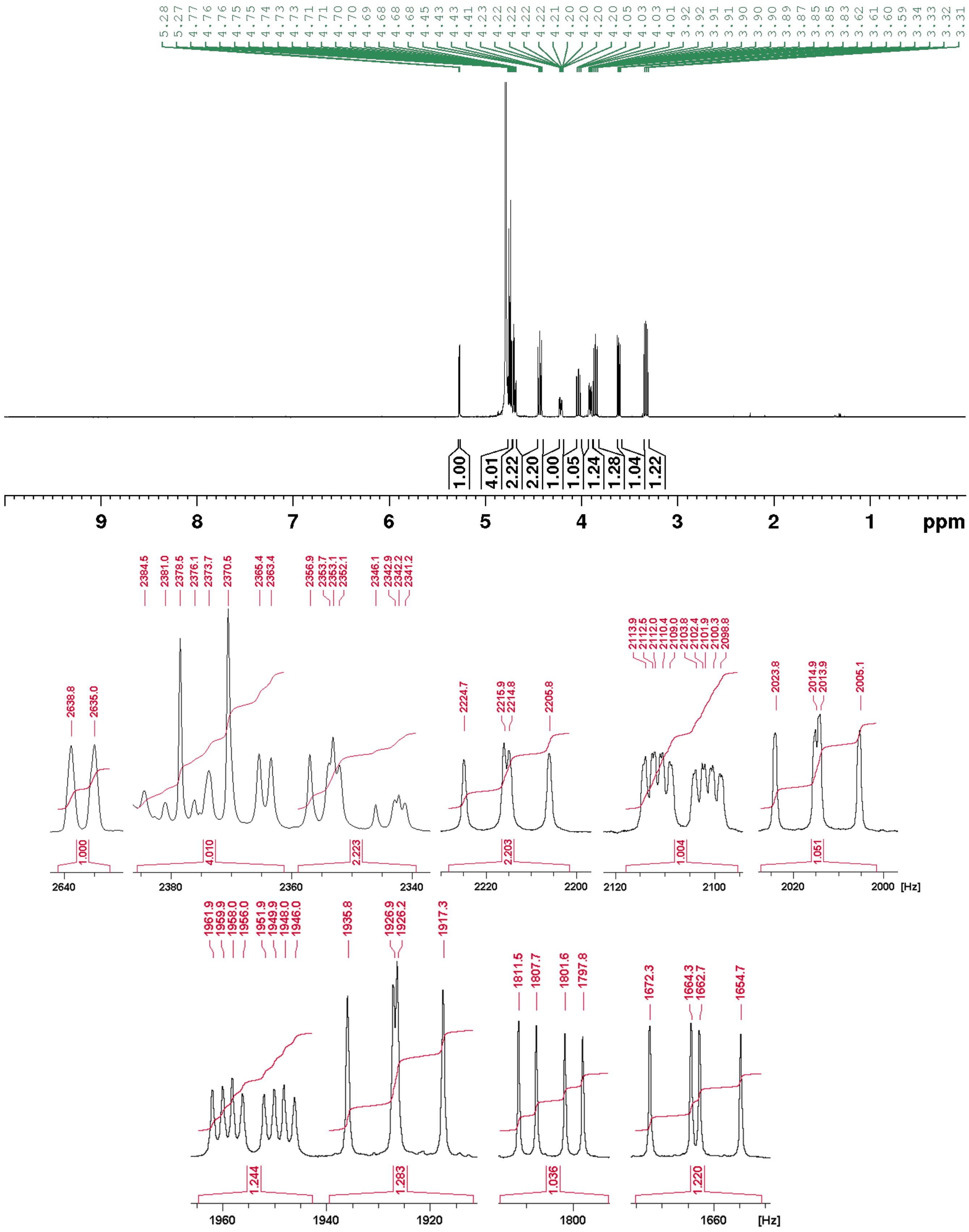

7.5.3 FDGlc-46 (**5**): **^{19}F NMR** (470 MHz, D_2O)

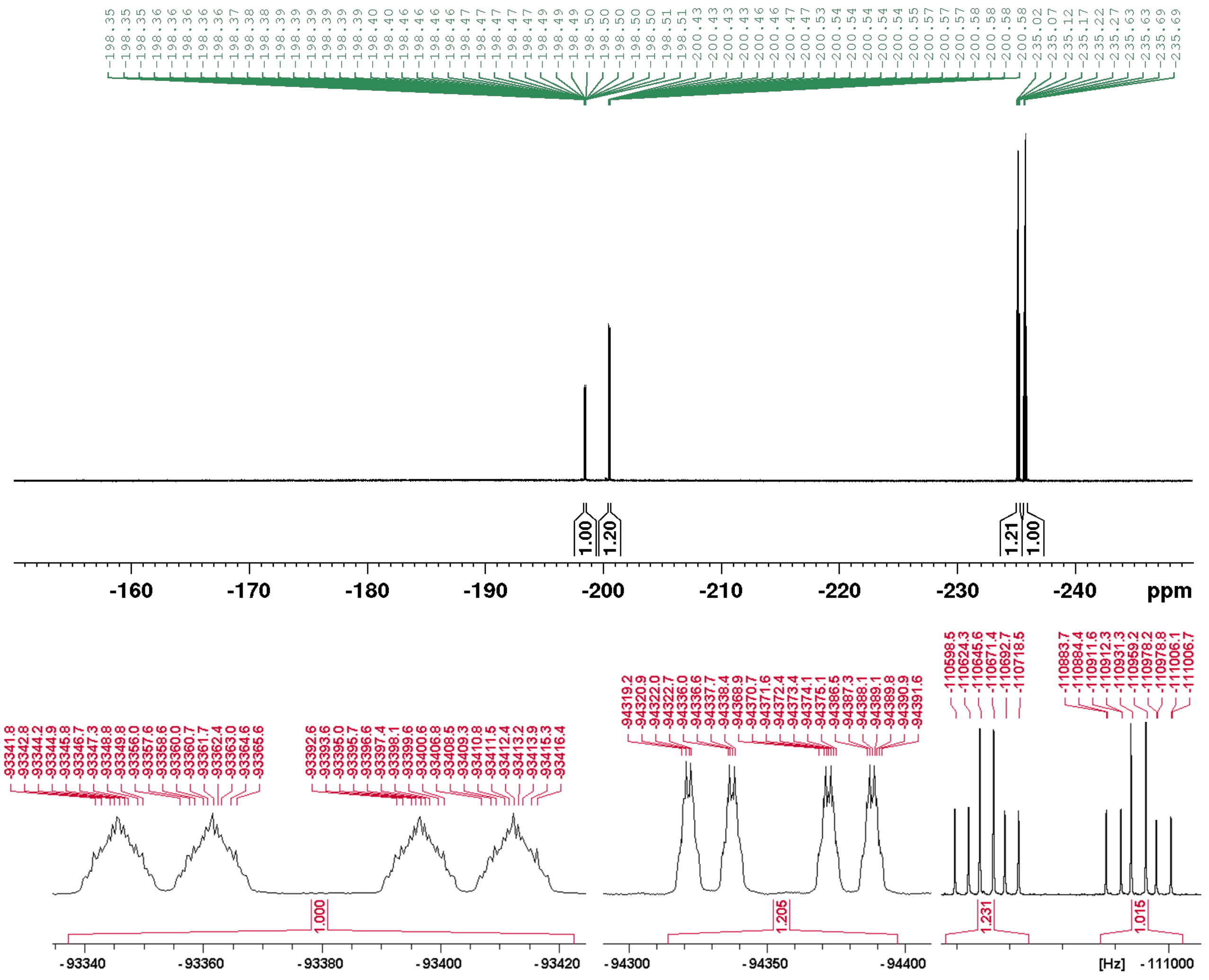

7.5.4 FDGlc-46 (**5**): **^{19}F{^{1}H} NMR** (470 MHz, D_2O)

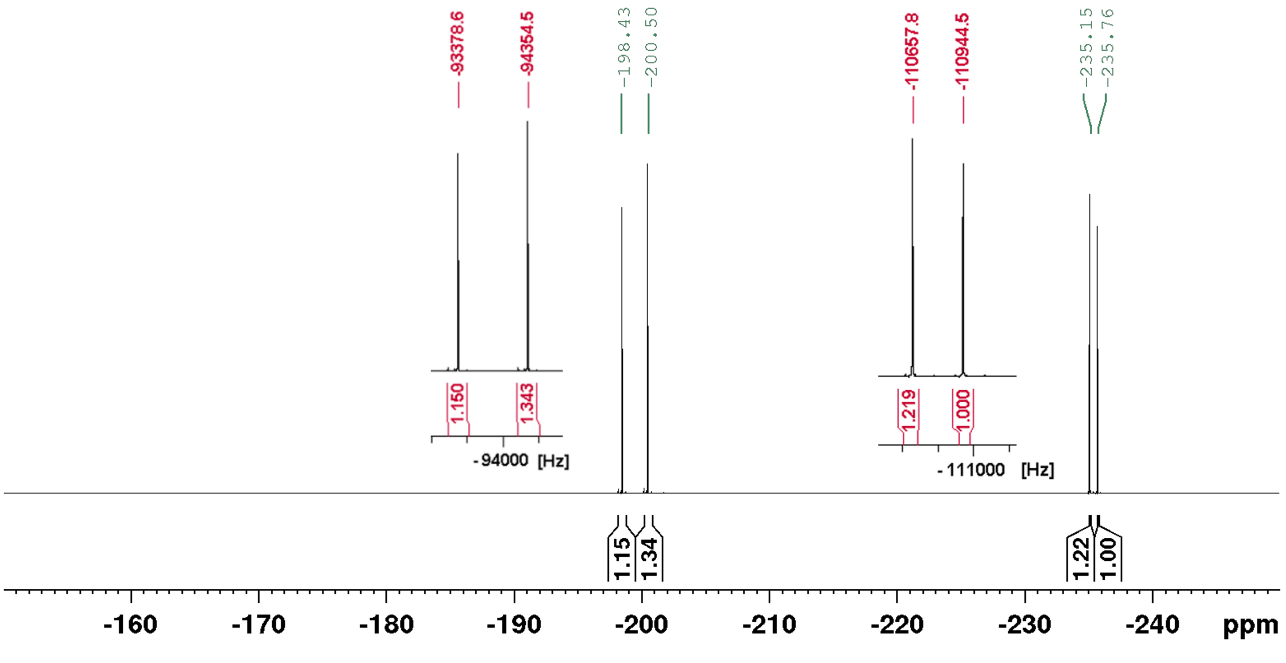

7.5.5 FDGlc-46 (**5**): **^{1}H-^{1}H COSY** (500 MHz, D_2O)

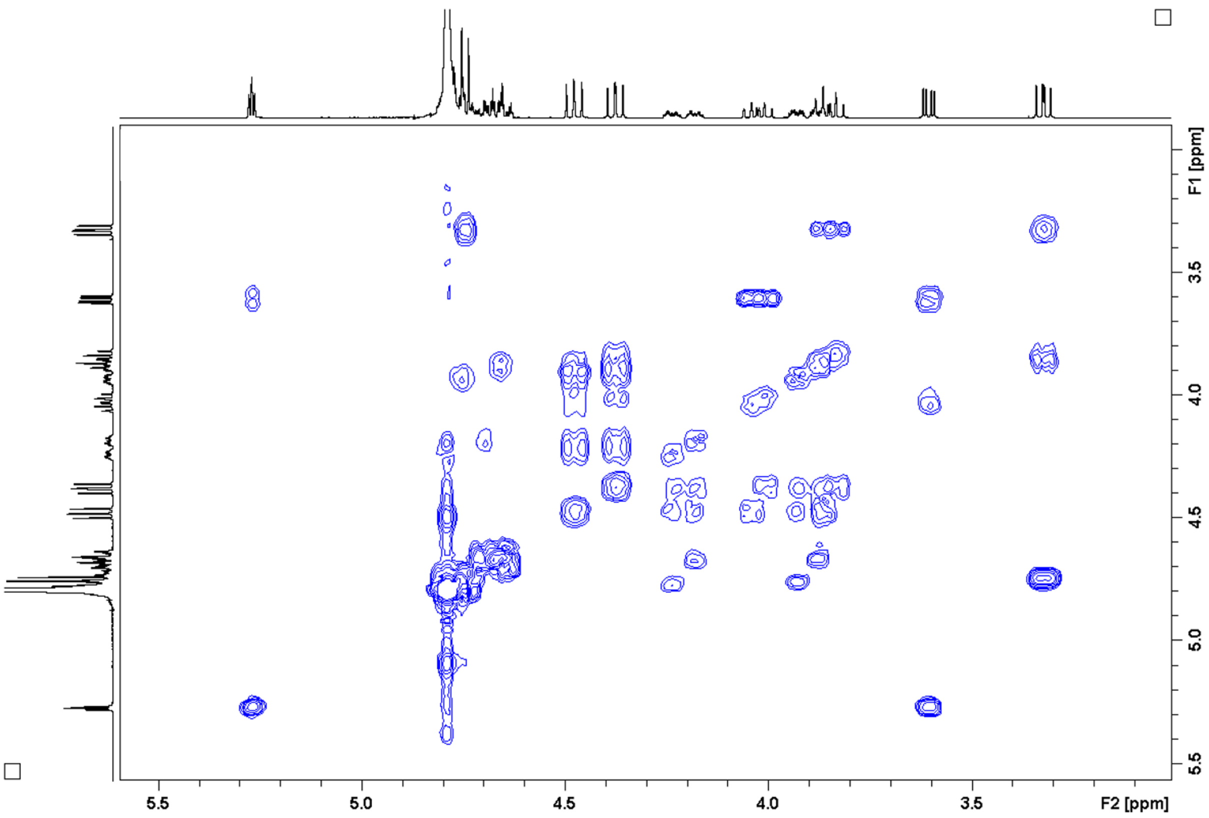

7.5.6 FDGlc-46 (**5**): **^{1}H-^{1}H{^{19}F} COSY** (500 MHz, D_2O)

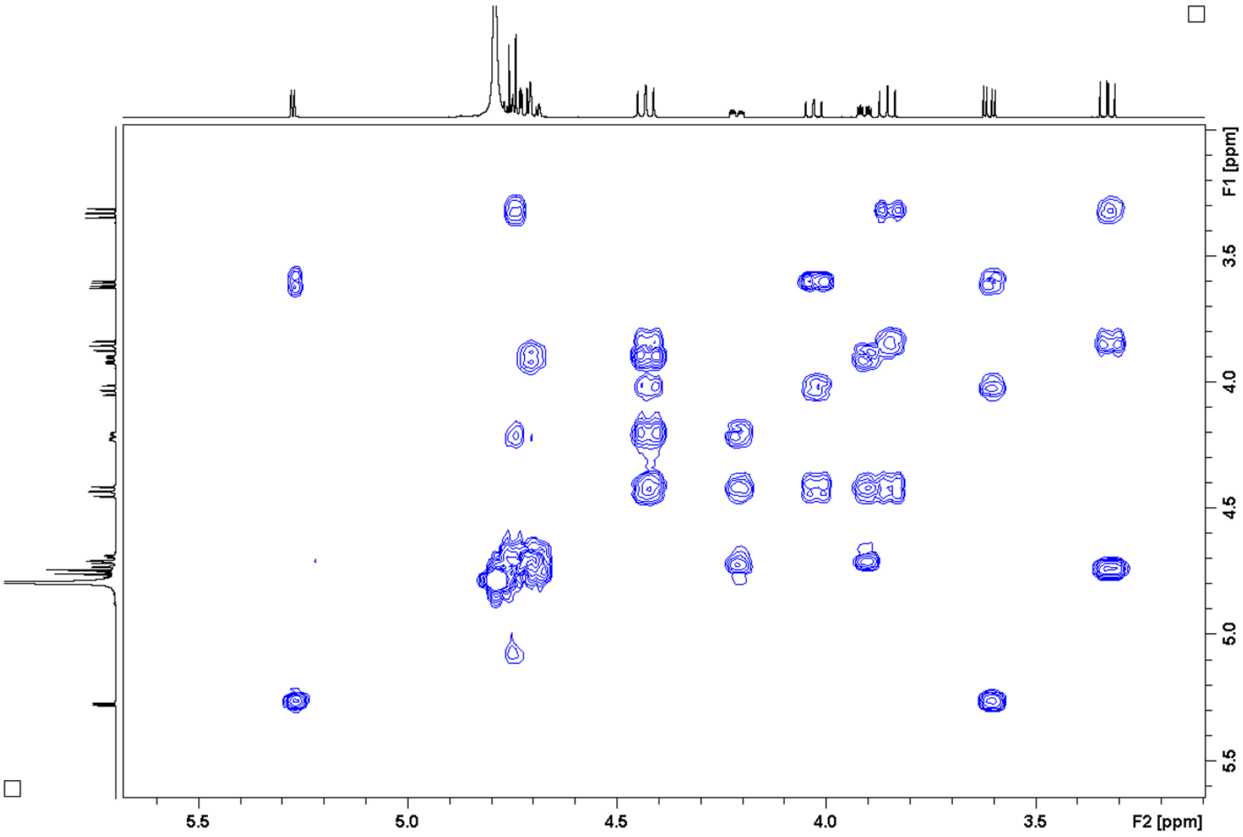

7.5.7 FDGlc-46 (**5**): α-pyranose form (α-*p*-FDGlc-46): **^{1}H{^{19}F} SRI-FESTA NMR** (500 MHz, D_2O, δ^{19}F = -198.43 ppm, δ^{1}H = 4.43 ppm, τ_m = 80 ms)

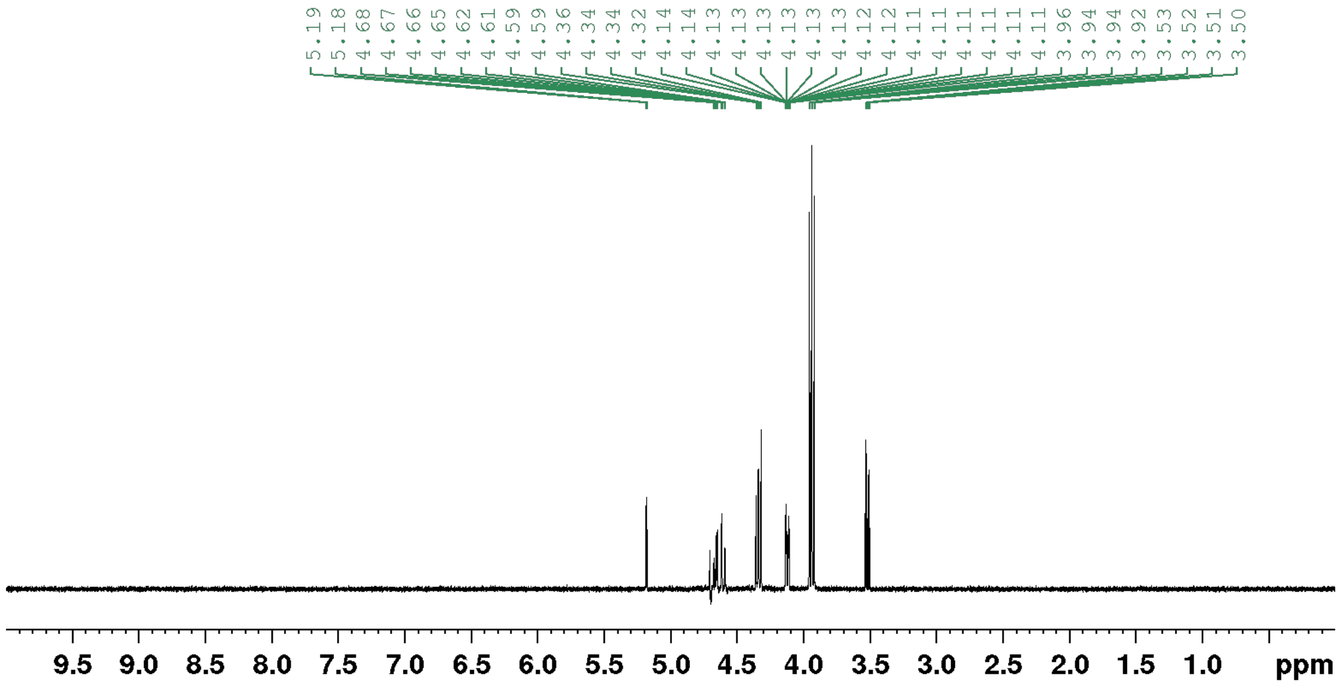

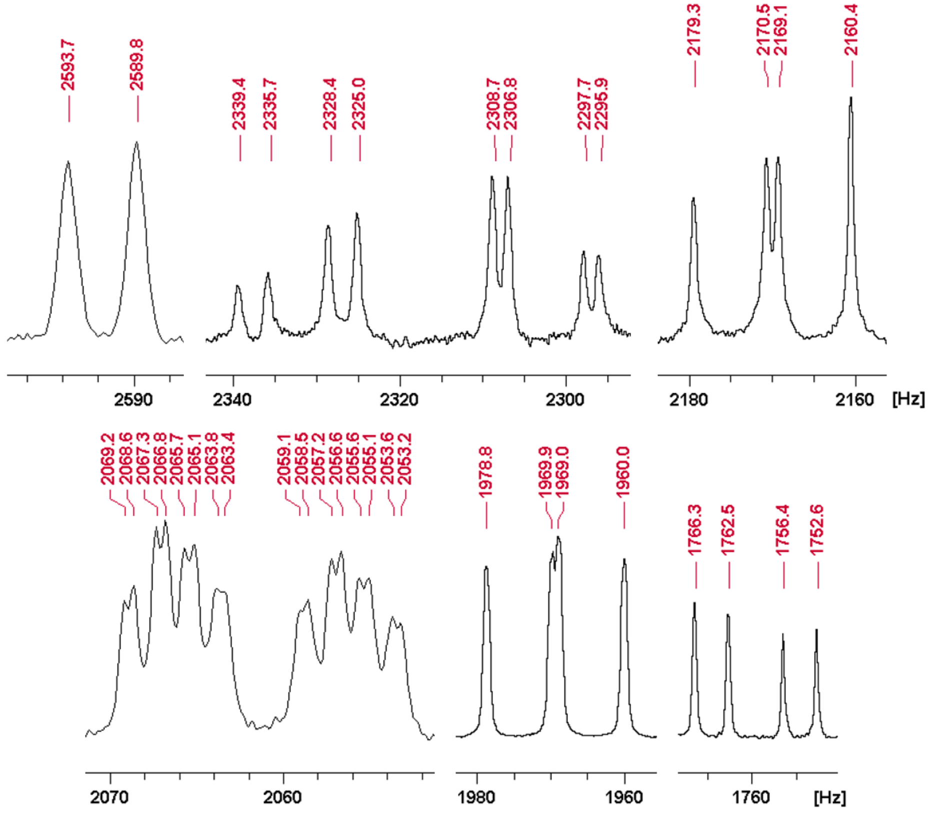

7.5.8 FDGlc-46 (**5**): β-pyranose form (β-*p*-FDGlc-46): **^{1}H{^{19}F} SRI-FESTA NMR** (500 MHz, D_2O, δ^{19}F = -200.50 ppm, δ^{1}H = 4.43 ppm, τ_m = 80 ms)

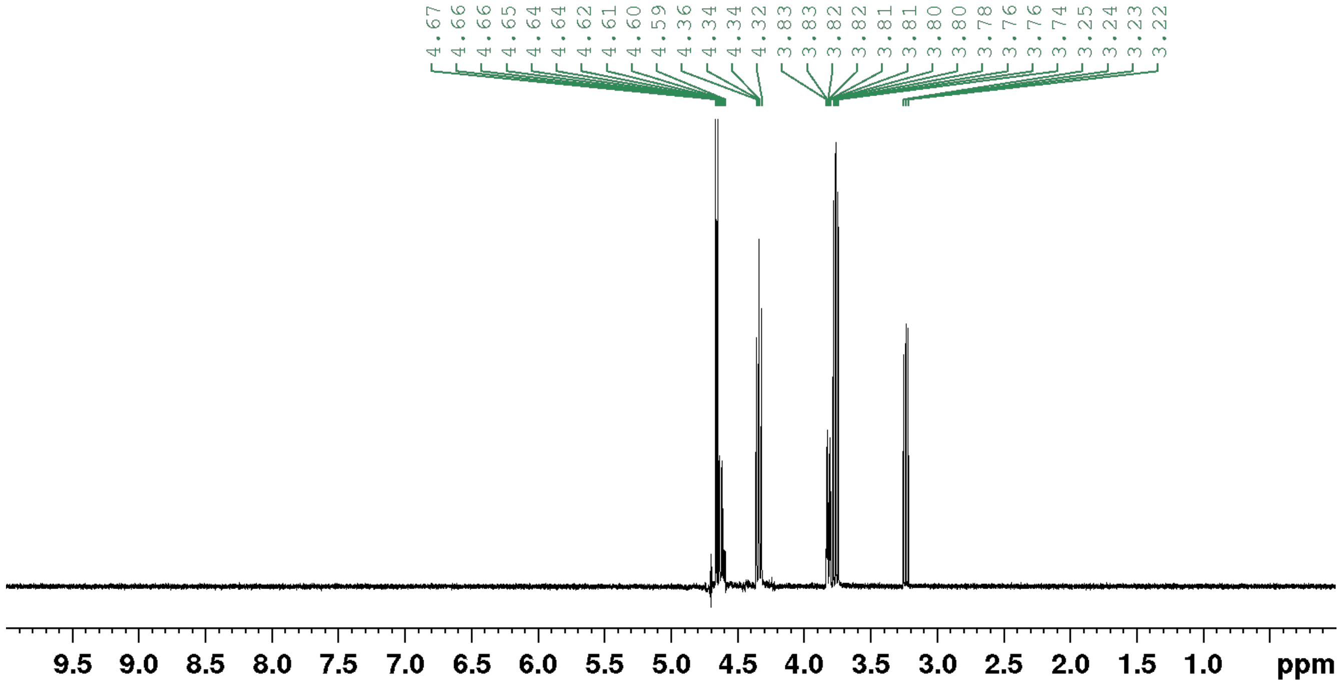

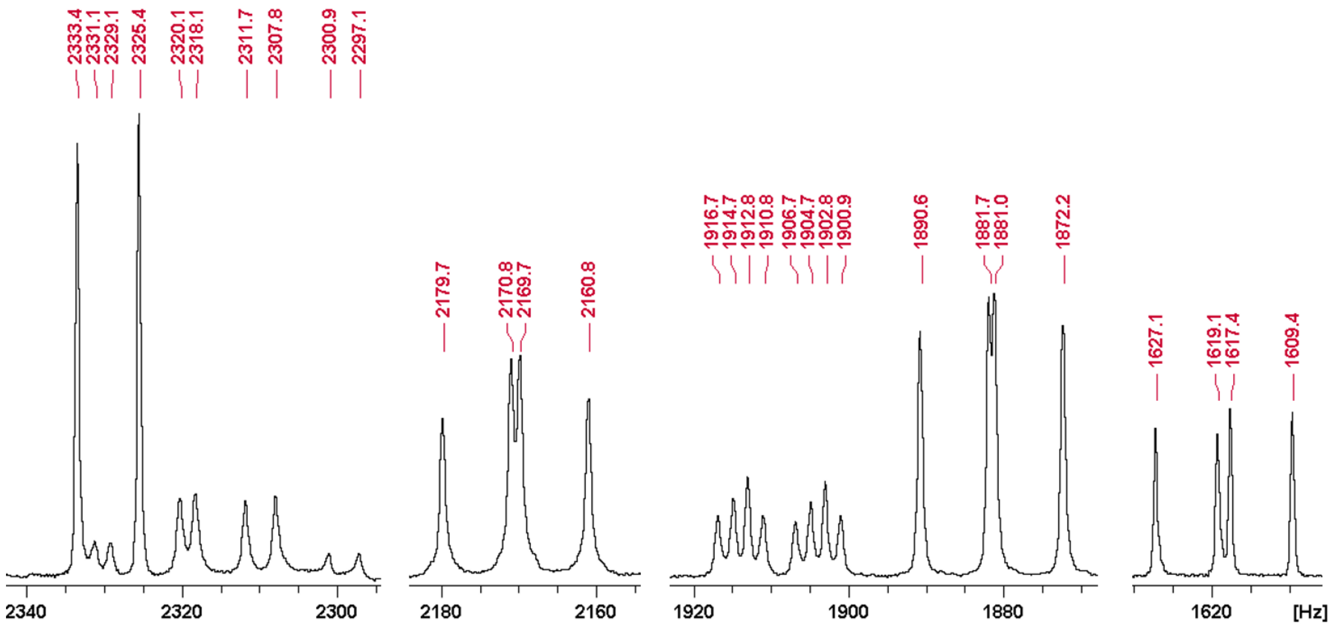

7.6 3-Deoxy-3-fluoro-D-galactose (6, FDGal-3): 35.2 : 63.5 : 0.4: 0.9 α-pyranose / β-pyranose / α-furanose / β-furanose, in D_2O.

7.6.1 FDGal-3 (**6**): ^{1}**H NMR** (600 MHz, D_2O)

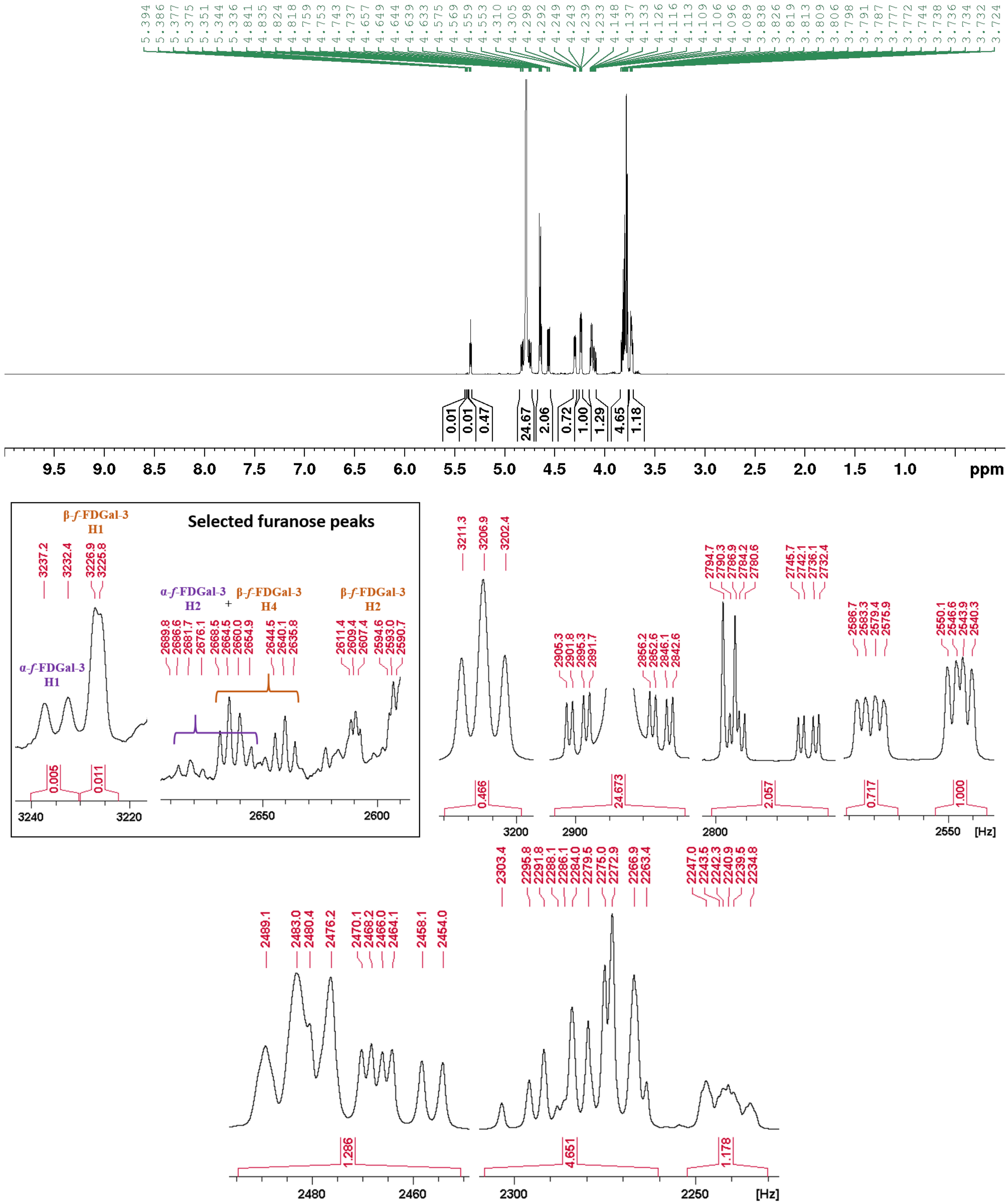

7.6.2 FDGal-3 (**6**): **$^{1}H\{^{19}F\}$ NMR** (600 MHz, D_2O)

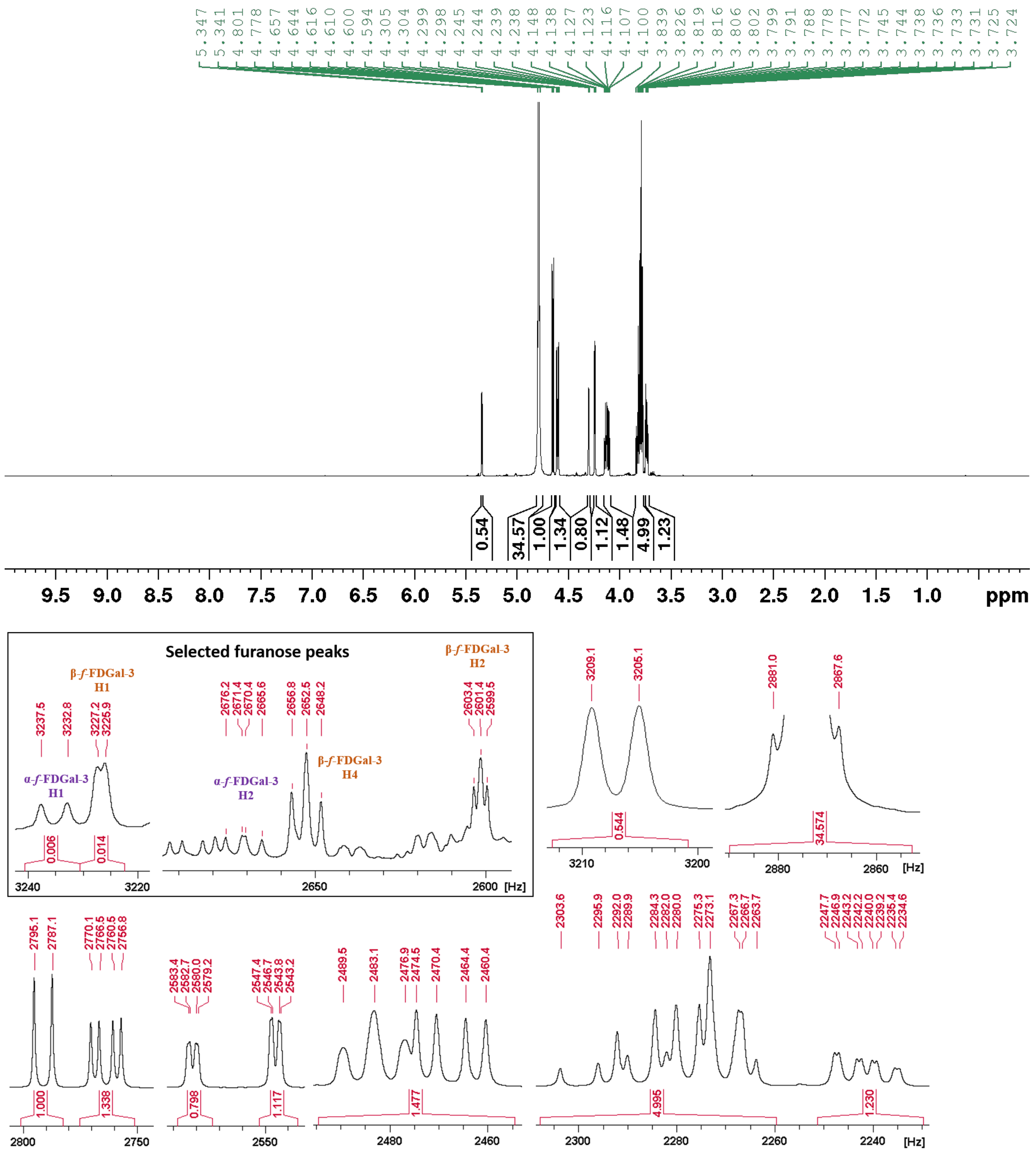

7.6.3 FDGal-3 (**6**): **^{19}F NMR** (565 MHz, D_2O)

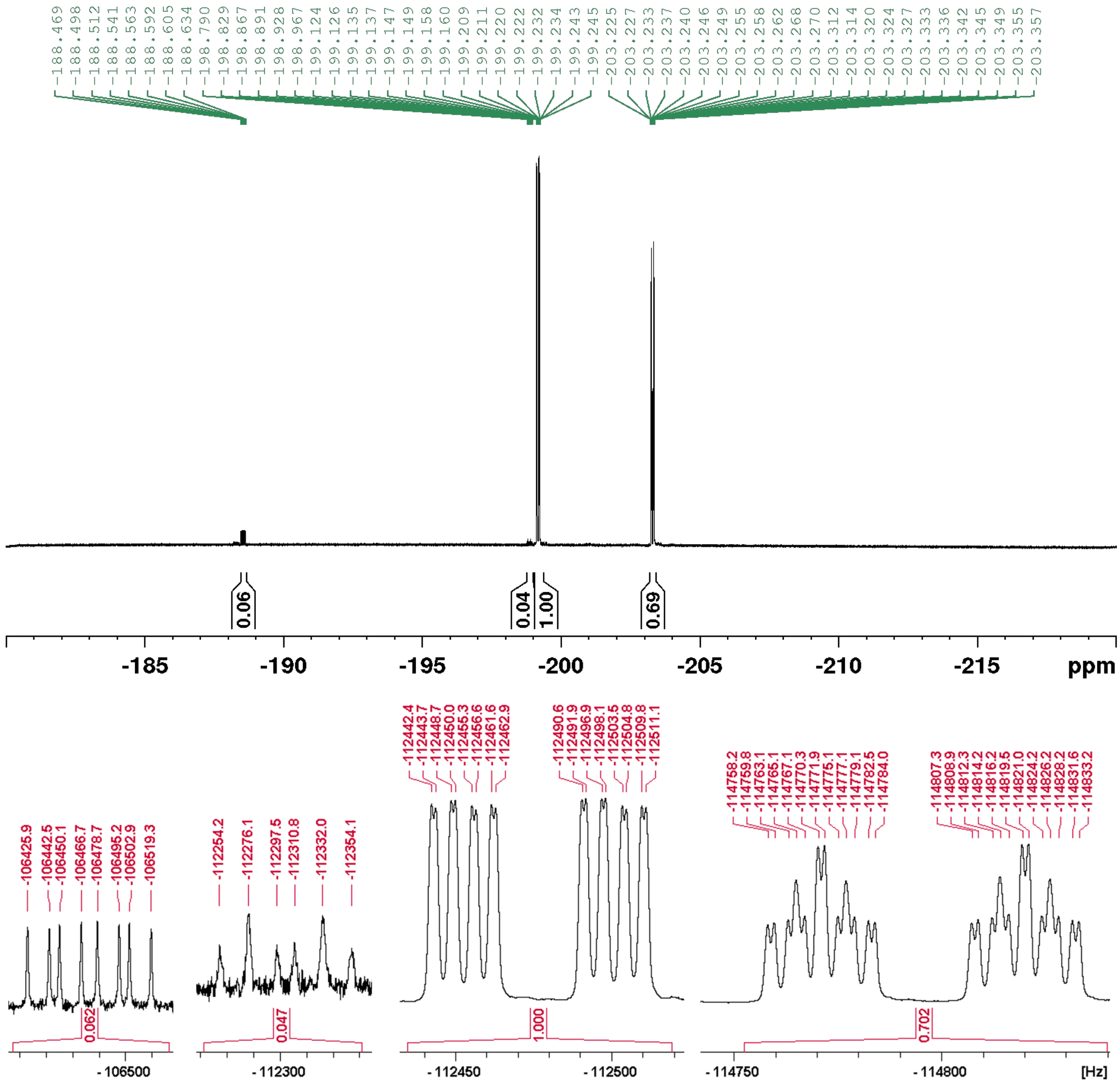

7.6.4 FDGal-3 (**6**): **$^{19}F\{^1H\}$ NMR** (565 MHz, D_2O)

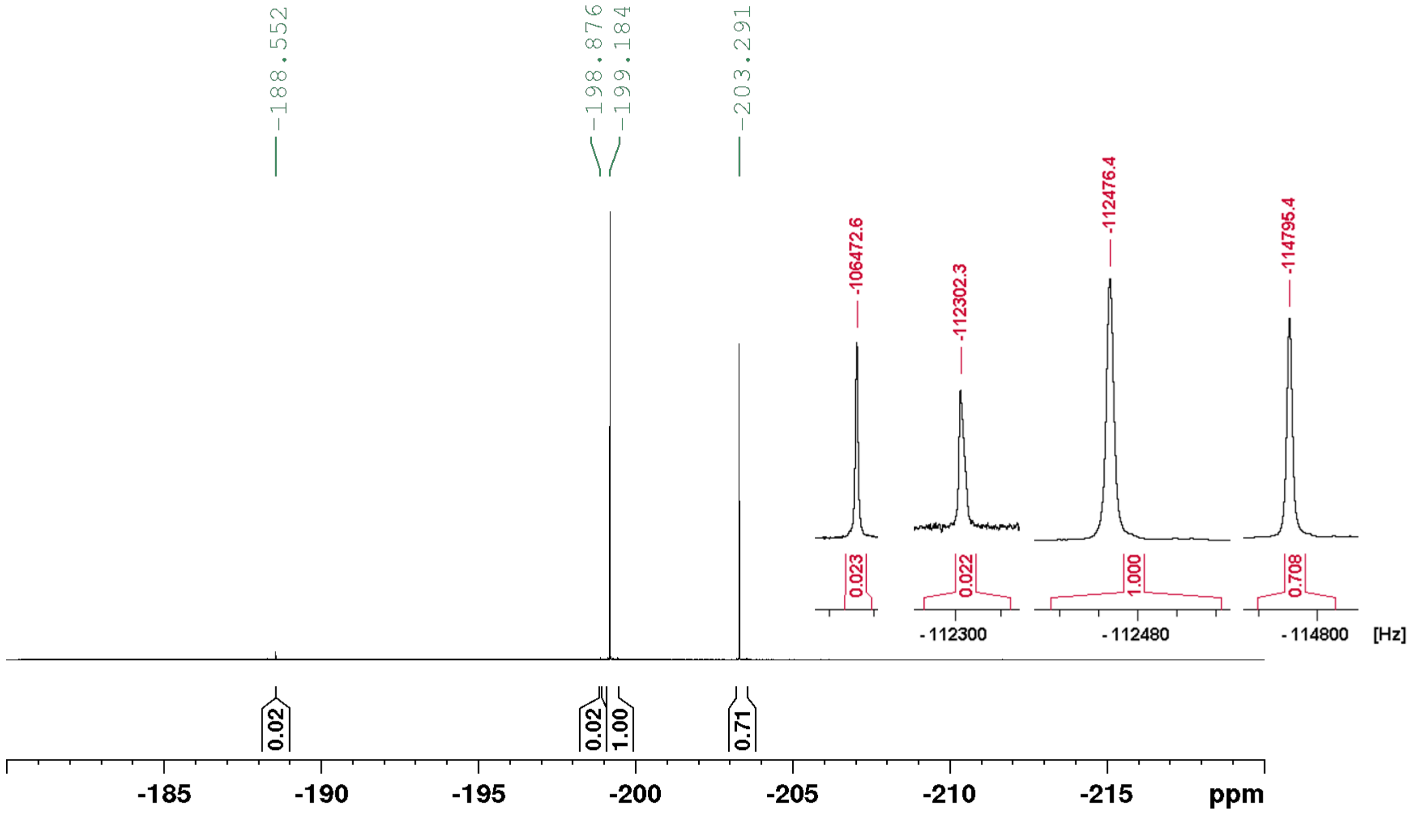

7.6.5 FDGal-3 (**6**): **^1H-$^1H\{^{19}F\}$ COSY** (600 MHz, D_2O)

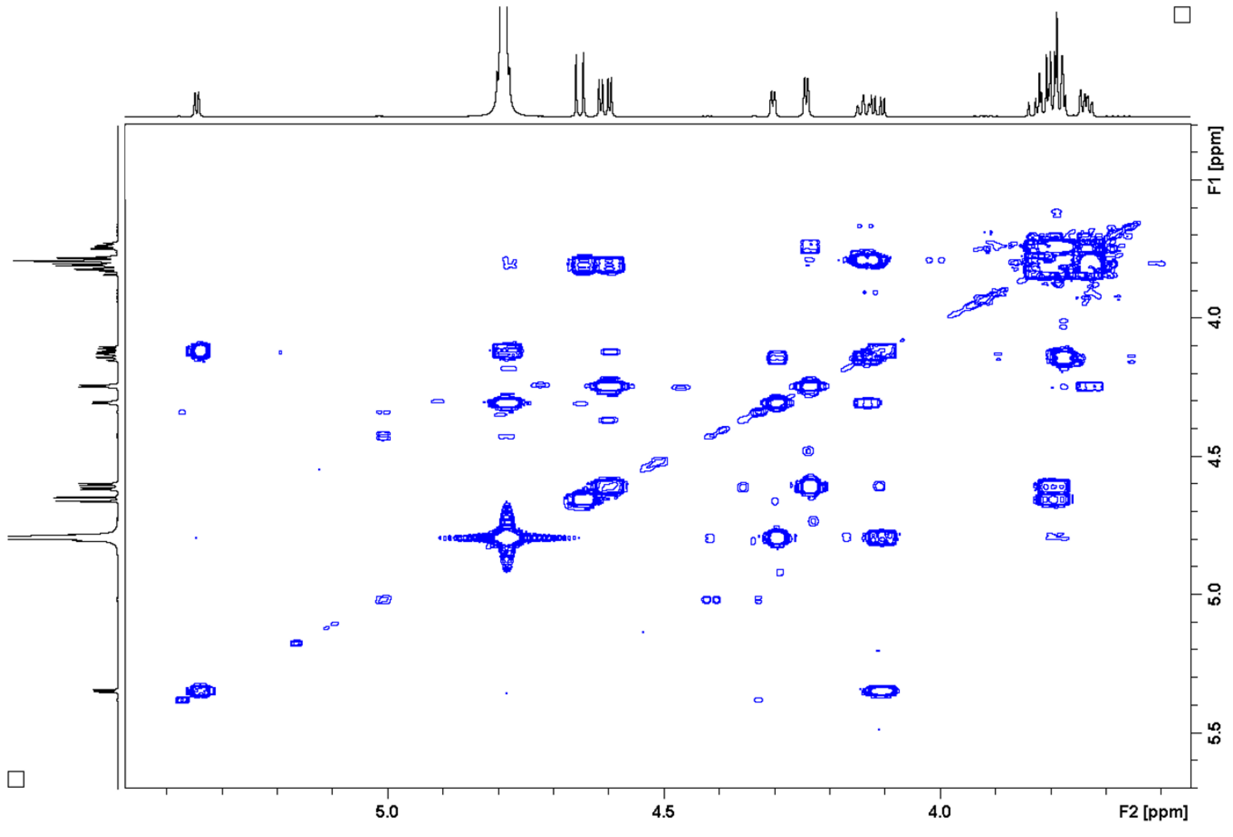

7.6.6 FDGal-3 (**6**): α-pyranose form (α-*p*-FDGal-3): **^{1}H{^{19}F} SRI-FESTA NMR** (600 MHz, D_2O, δ^{19}F = -203.29 ppm, δ^1H = 4.17-4.06 ppm, τ_m = 300 ms)

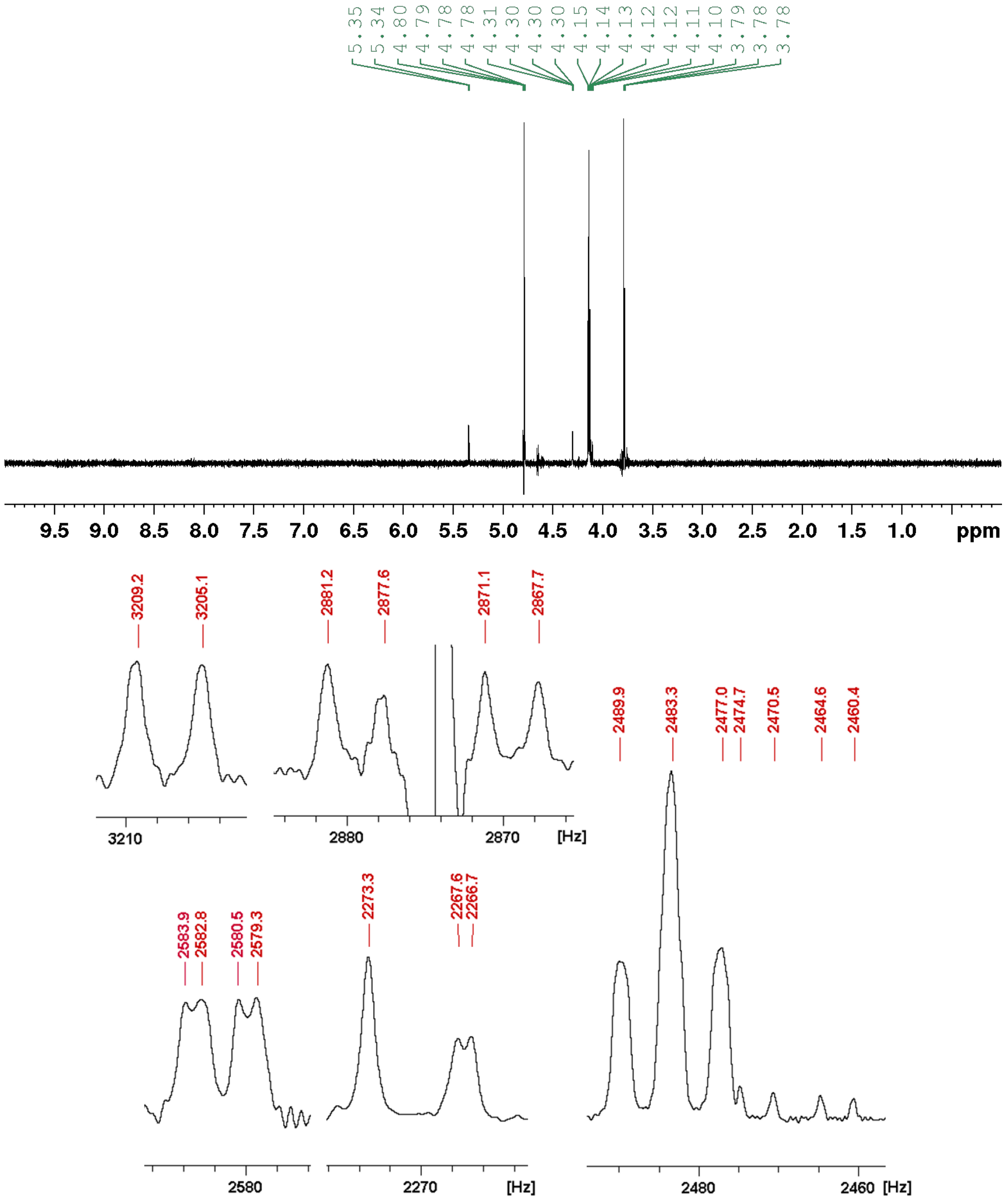

7.6.7 FDGal-3 (**6**): β-pyranose form (β-*p*-FDGal-3): **^{1}H{^{19}F} SRI-FESTA NMR** (600 MHz, D_2O, δ^{19}F = -199.18 ppm, τ_m = 300 ms, a) δ^1H = 4.24 ppm; b) δ^1H = 3.73 ppm)

a)

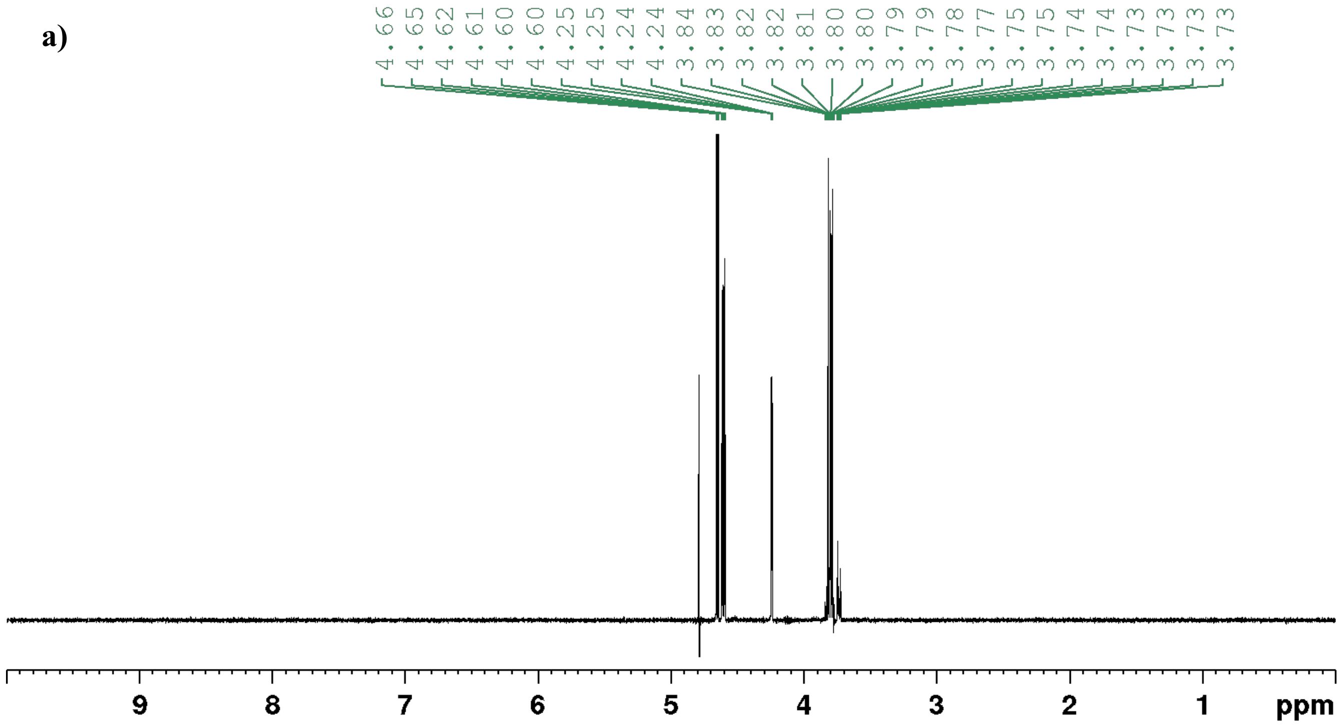

b)

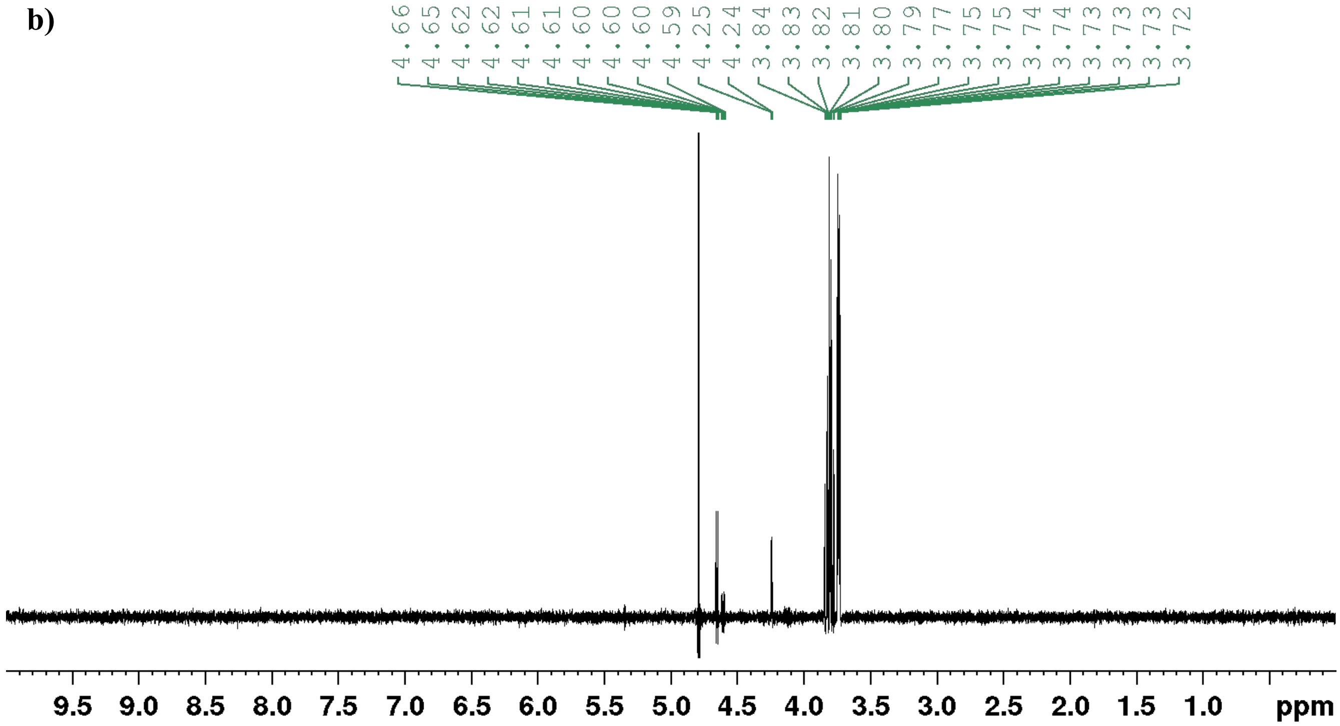

a)

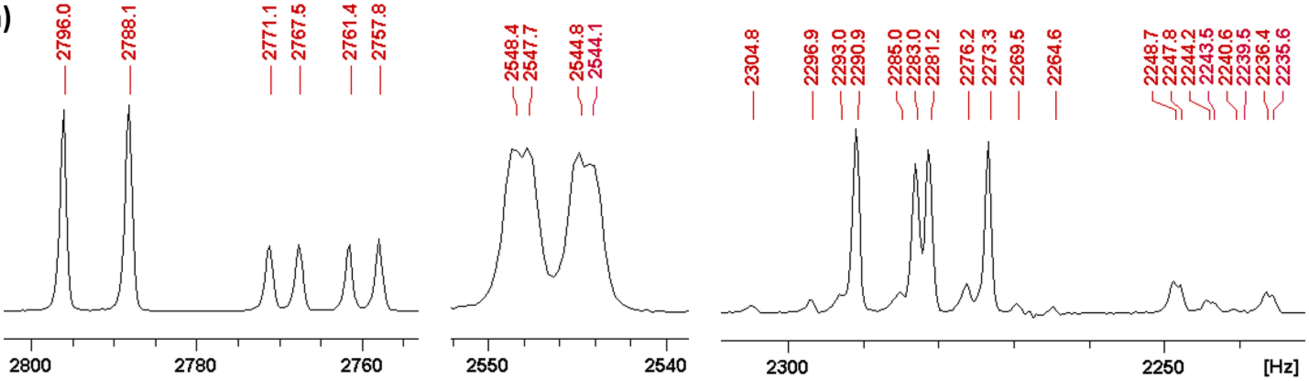

Complimentary peaks from b)

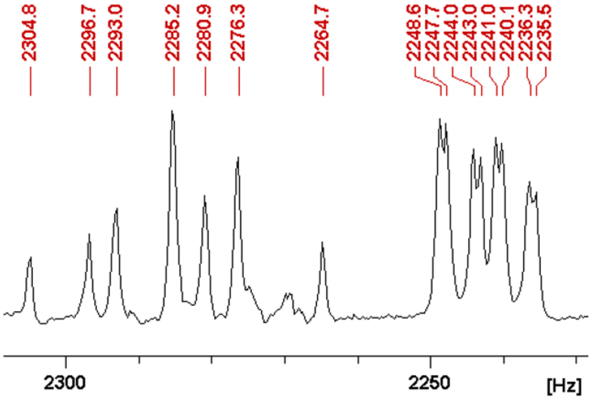

7.6.8 FDGal-3 (**6**): α-furanose form (α-*f*-FDGal-3): **$^{1}H\{^{19}F\}$ SRI-FESTA NMR** (600 MHz, D_2O, $\delta^{19}F$ = -198.88 ppm, $\delta^{1}H$ = 4.45 ppm, τ_m = 200 ms, 4096 ns)

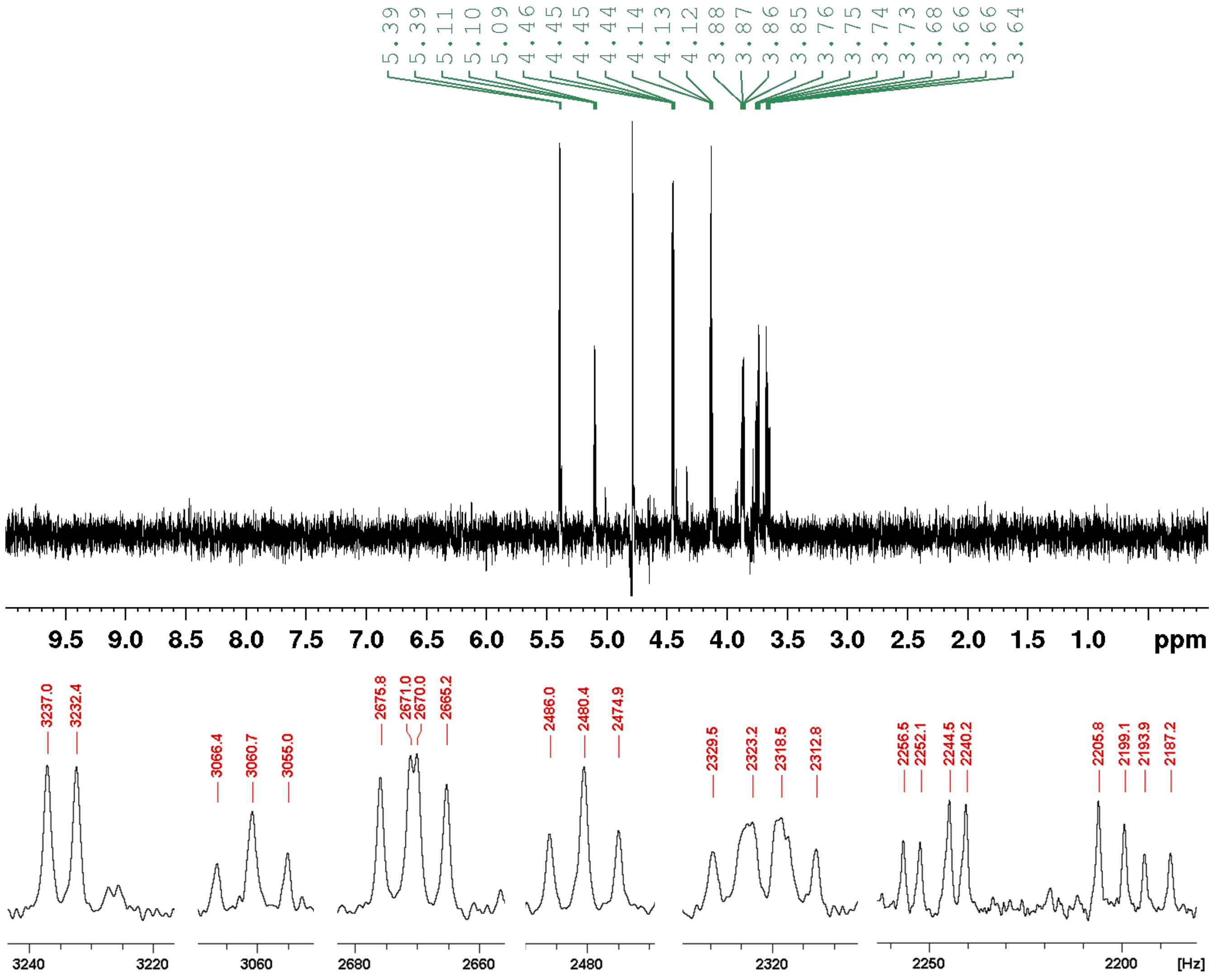

7.6.9 FDGal-3 (**6**): β-furanose form (β-*f*-FDGal-3): **^{1}H{^{19}F} SRI-FESTA NMR** (600 MHz, D_2O, δ^{19}F = -188.55 ppm, δ^1H = 4.42 ppm, τ_m = 300 ms, 4096 ns)

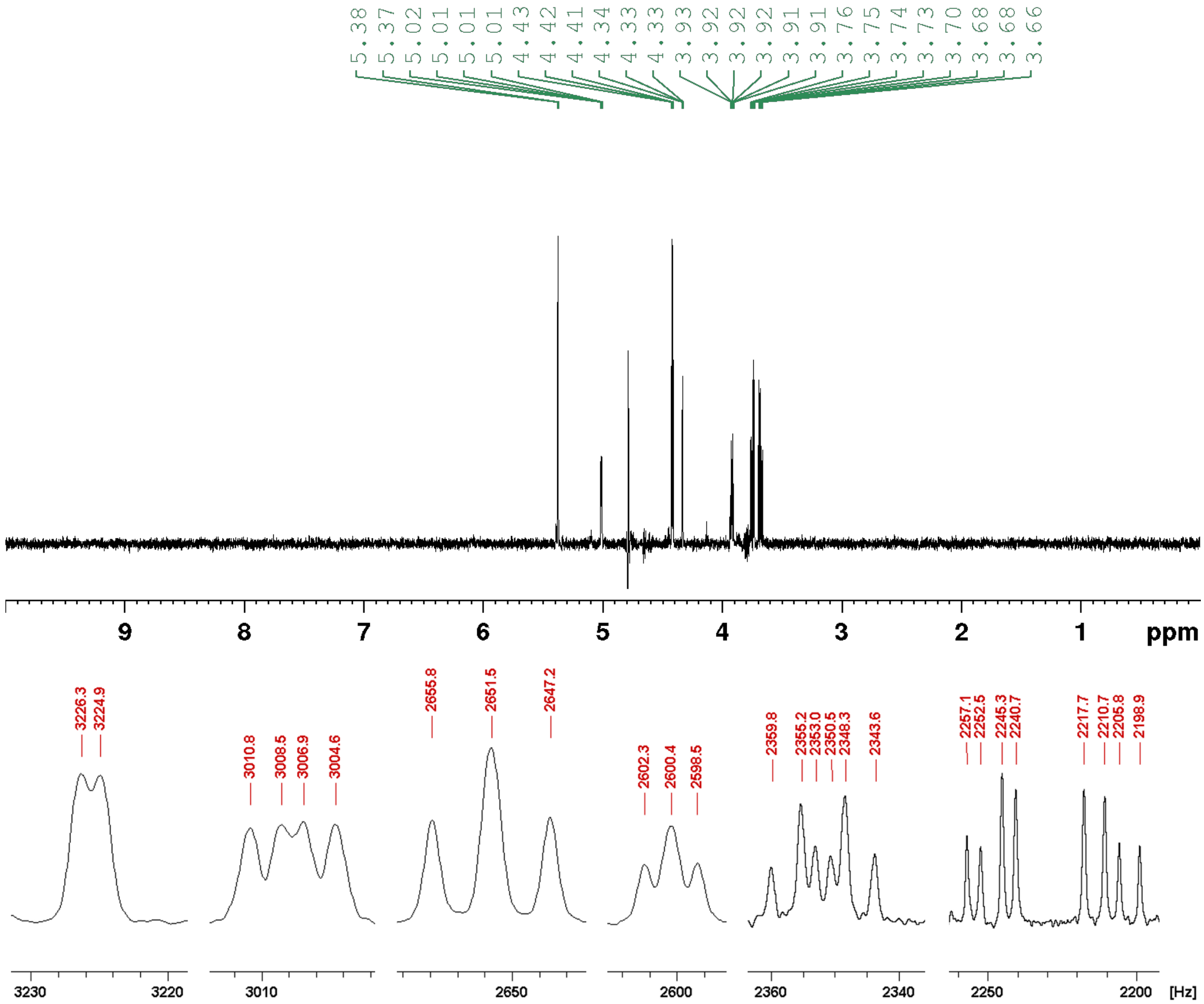

7.7 6-Deoxy-6-fluoro-D-galactose (7, FDGal-6): 32.7 : 58.8 : 3.5 : 5.0 α-pyranose / β-pyranose / α-furanose / β-furanose, in D_2O.

7.7.1 FDGal-6 (7): ^{1}H NMR (600 MHz, D_2O)

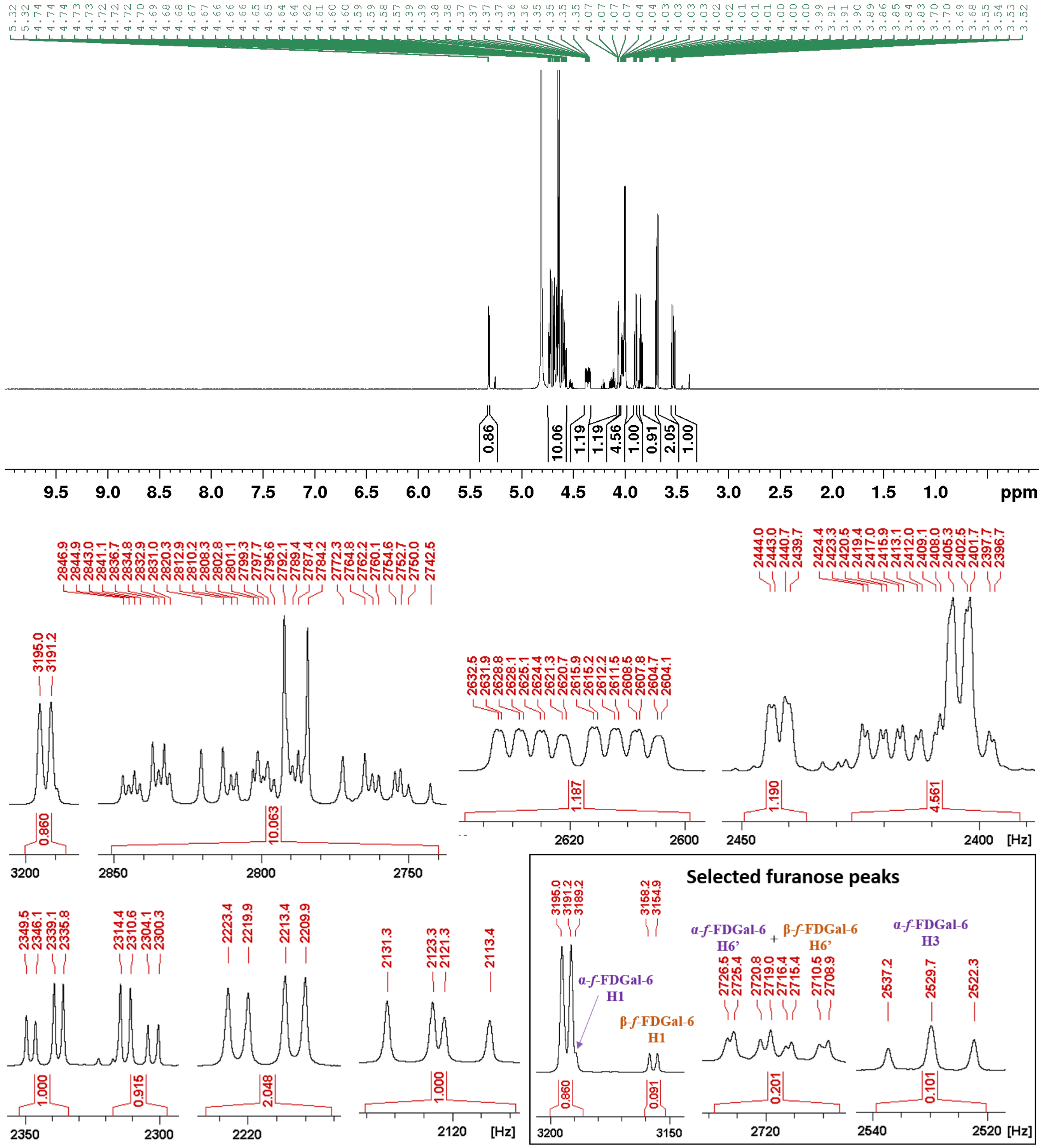

7.7.2 FDGal-6 (**7**): ^{1}**H{**^{19}**F} NMR** (600 MHz, D_2O)

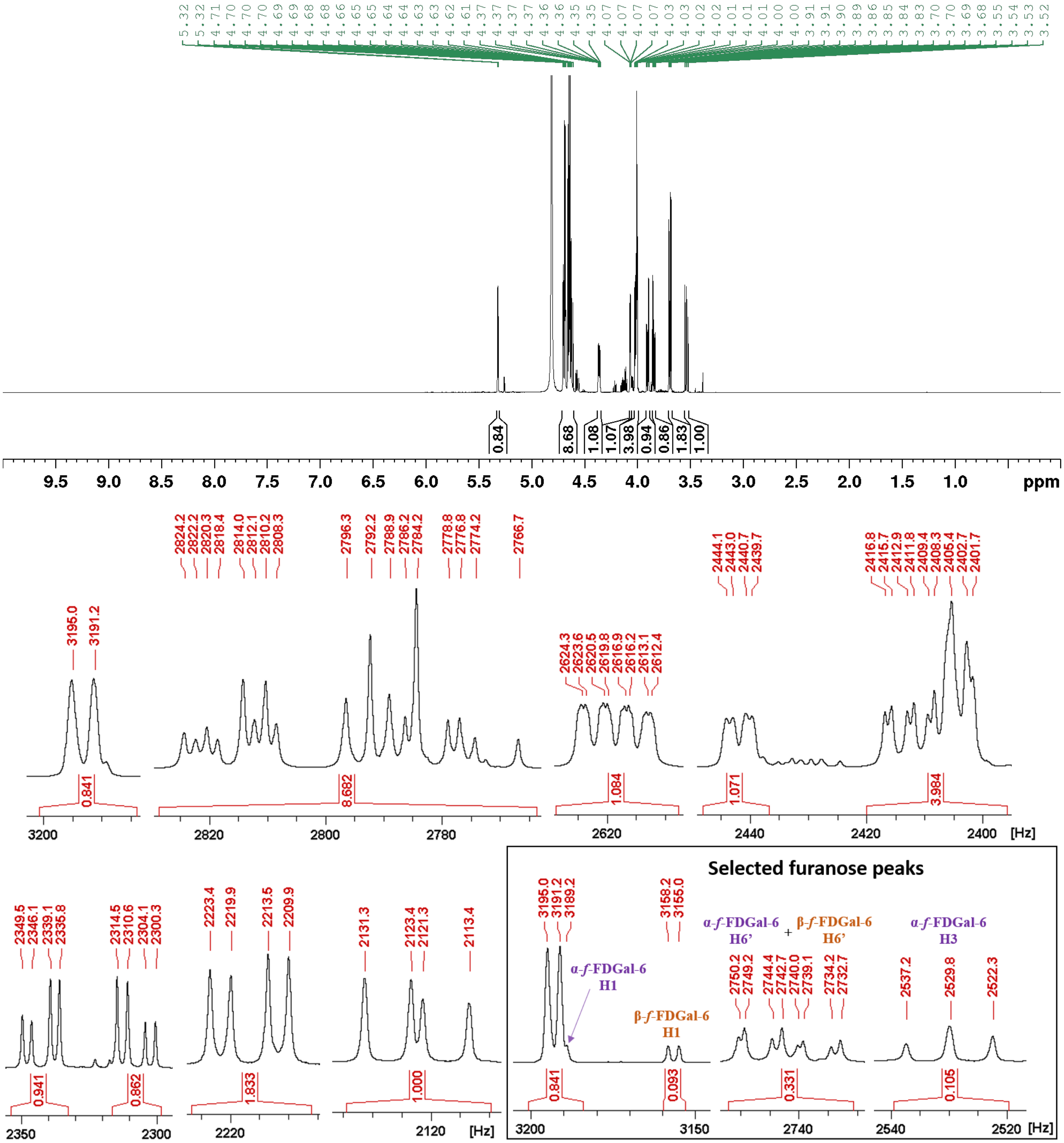

7.7.3 FDGal-6 (**7**): **^{19}F NMR** (565 MHz, D_2O)

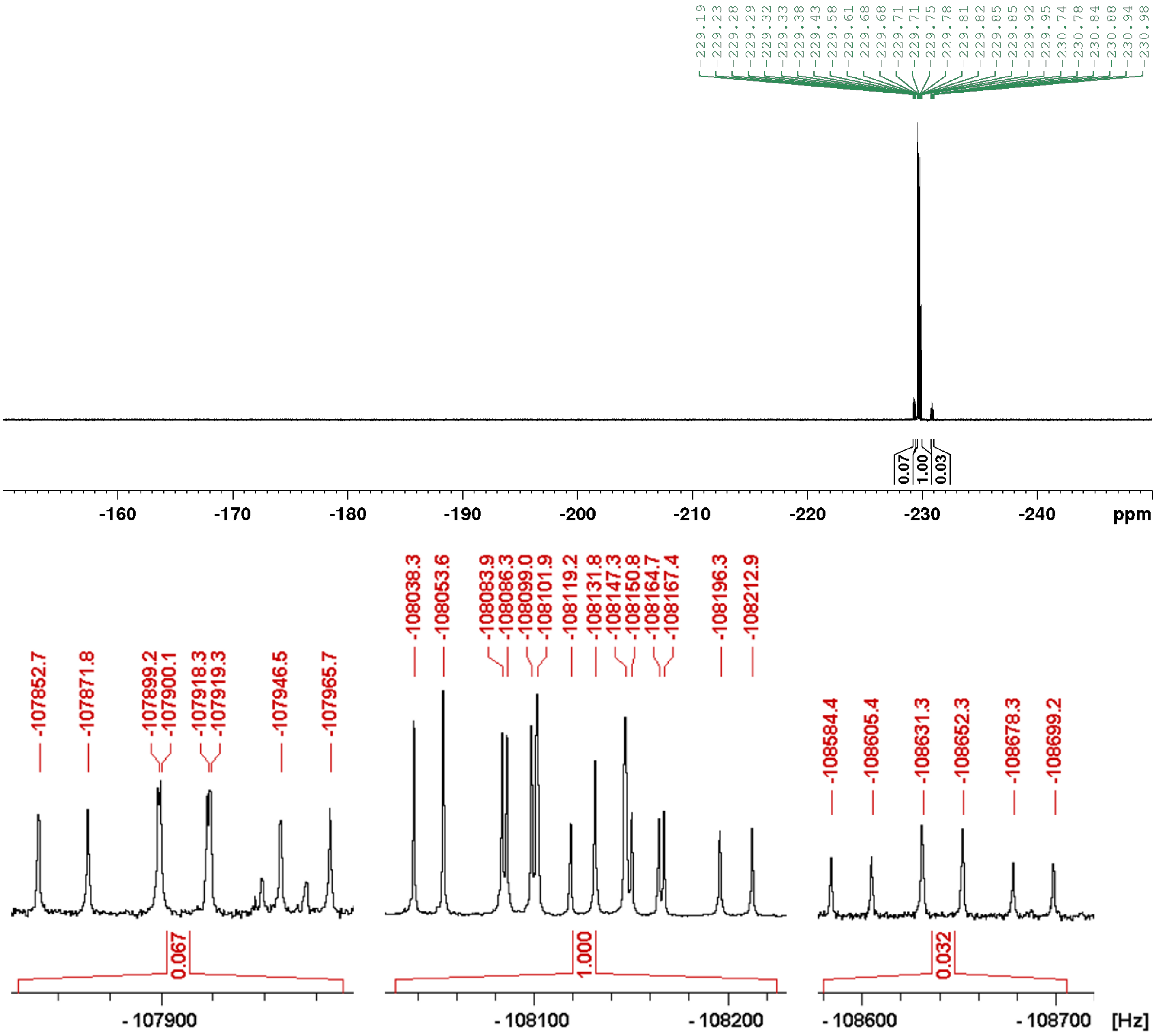

7.7.4 FDGal-6 (**7**): **^{19}F{^{1}H} NMR** (470 MHz, D$_2$O)

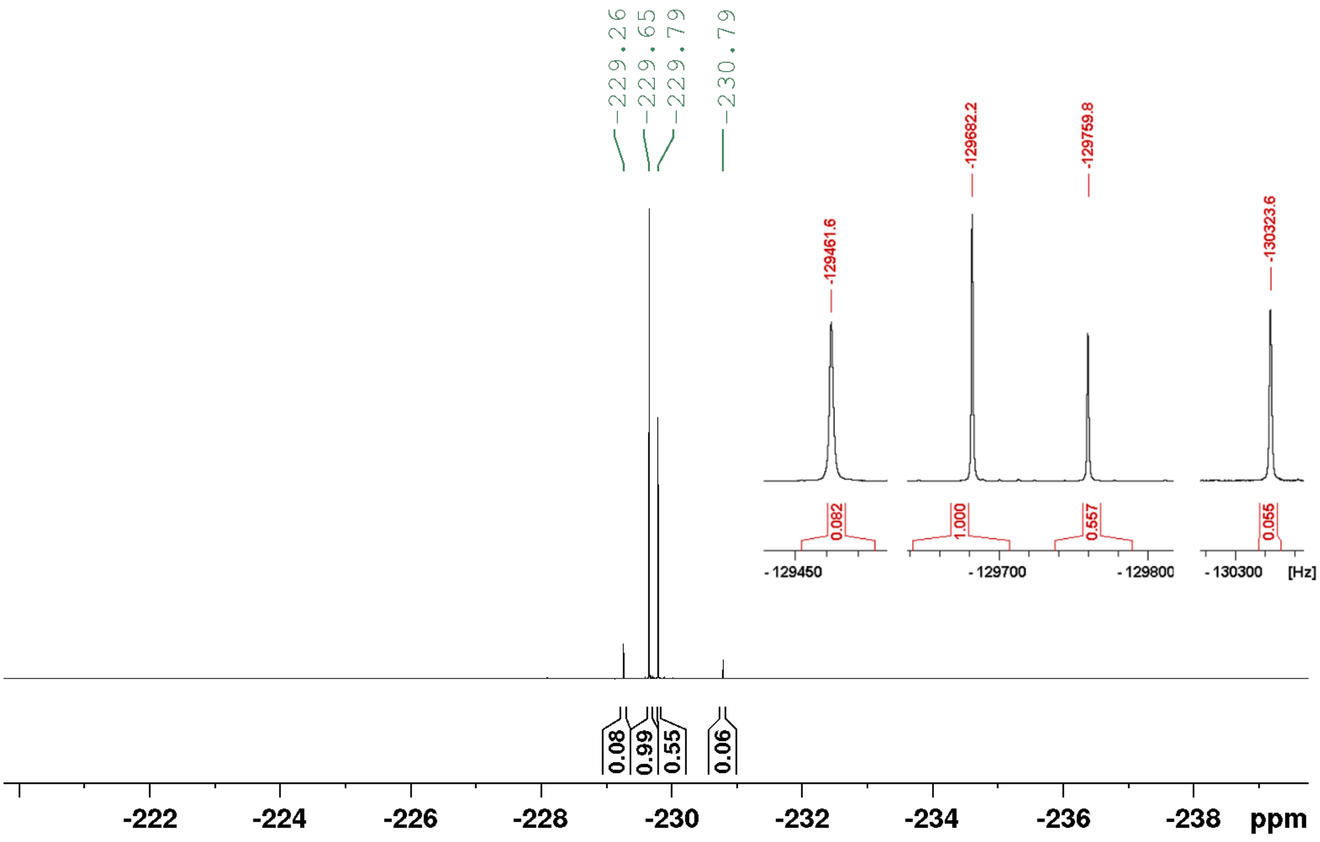

7.7.5 FDGal-6 (**7**): **^{1}H-^{1}H{^{19}F} COSY** (600 MHz, D$_2$O)

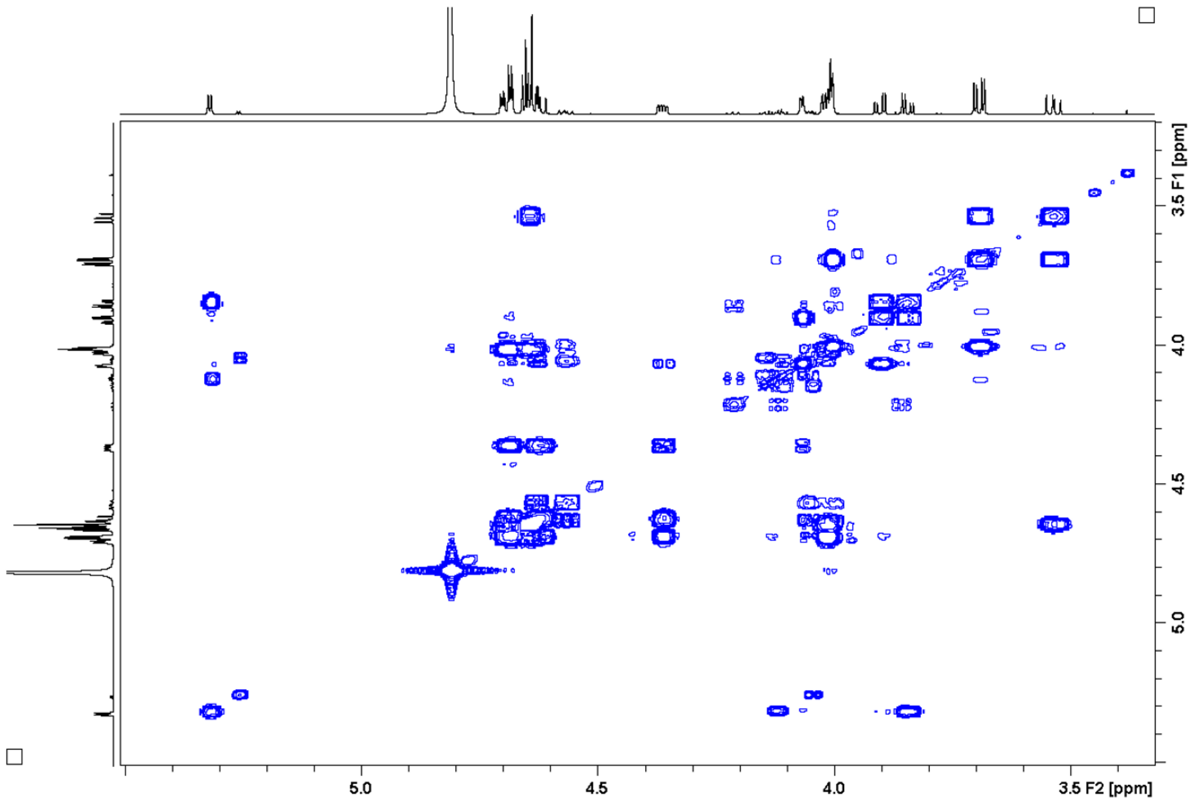

7.7.6 FDGal-6 (**7**): α-pyranose form (α-*p*-FDGal-6): **^{1}H{^{19}F} SRI-FESTA NMR** (600 MHz, D_2O, δ^{19}F = -229.79 ppm, δ^1H = 4.36 ppm, τ_m = 300 ms)

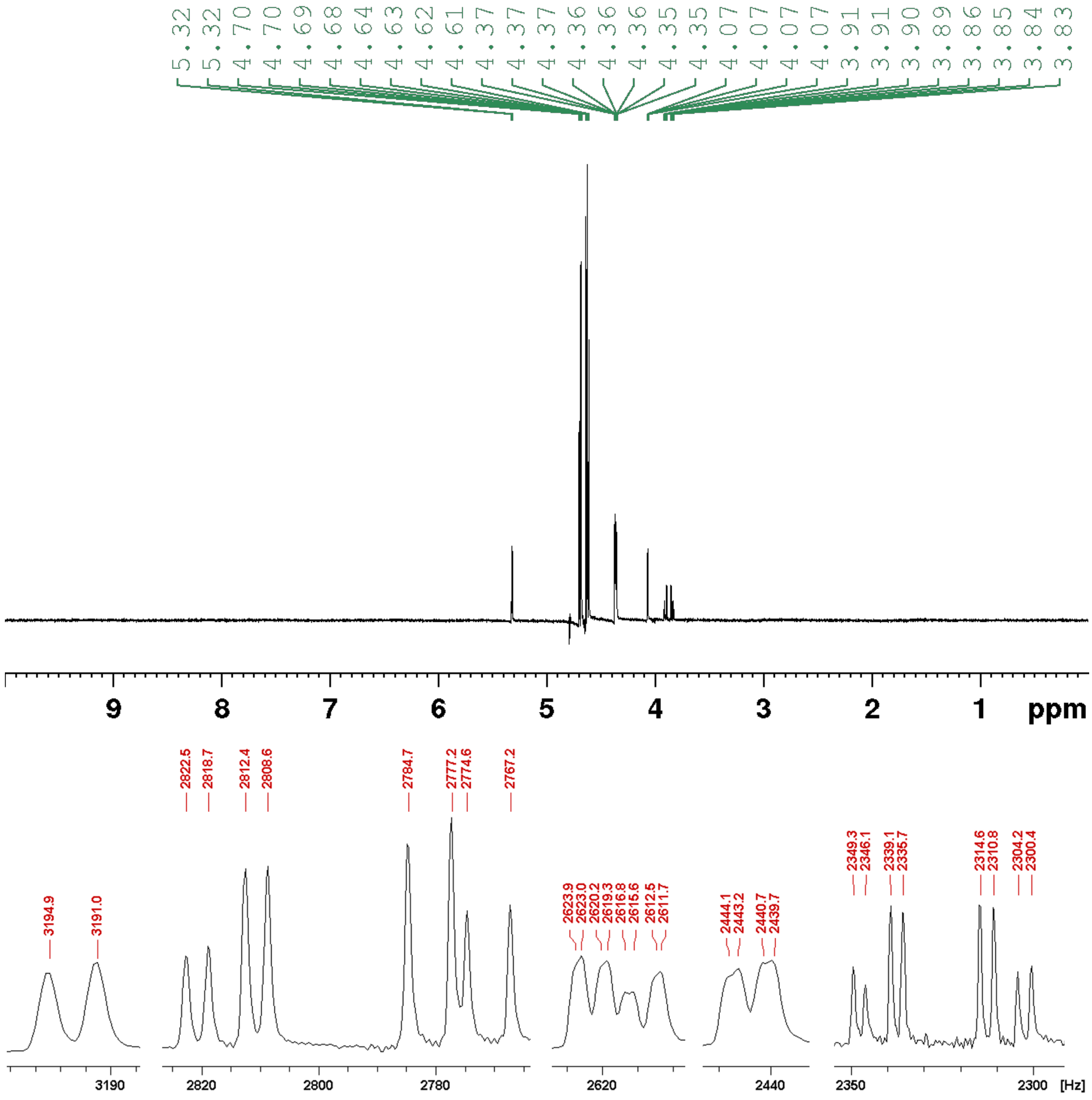

7.7.7 FDGal-6 (**7**): β-pyranose form (β-*p*-FDGal-6): **^{1}H{^{19}F} SRI-FESTA NMR** (600 MHz, D_2O, $\delta^{19}F$ = -229.65 ppm, δ^1H = 4.02 ppm, τ_m = 300 ms)

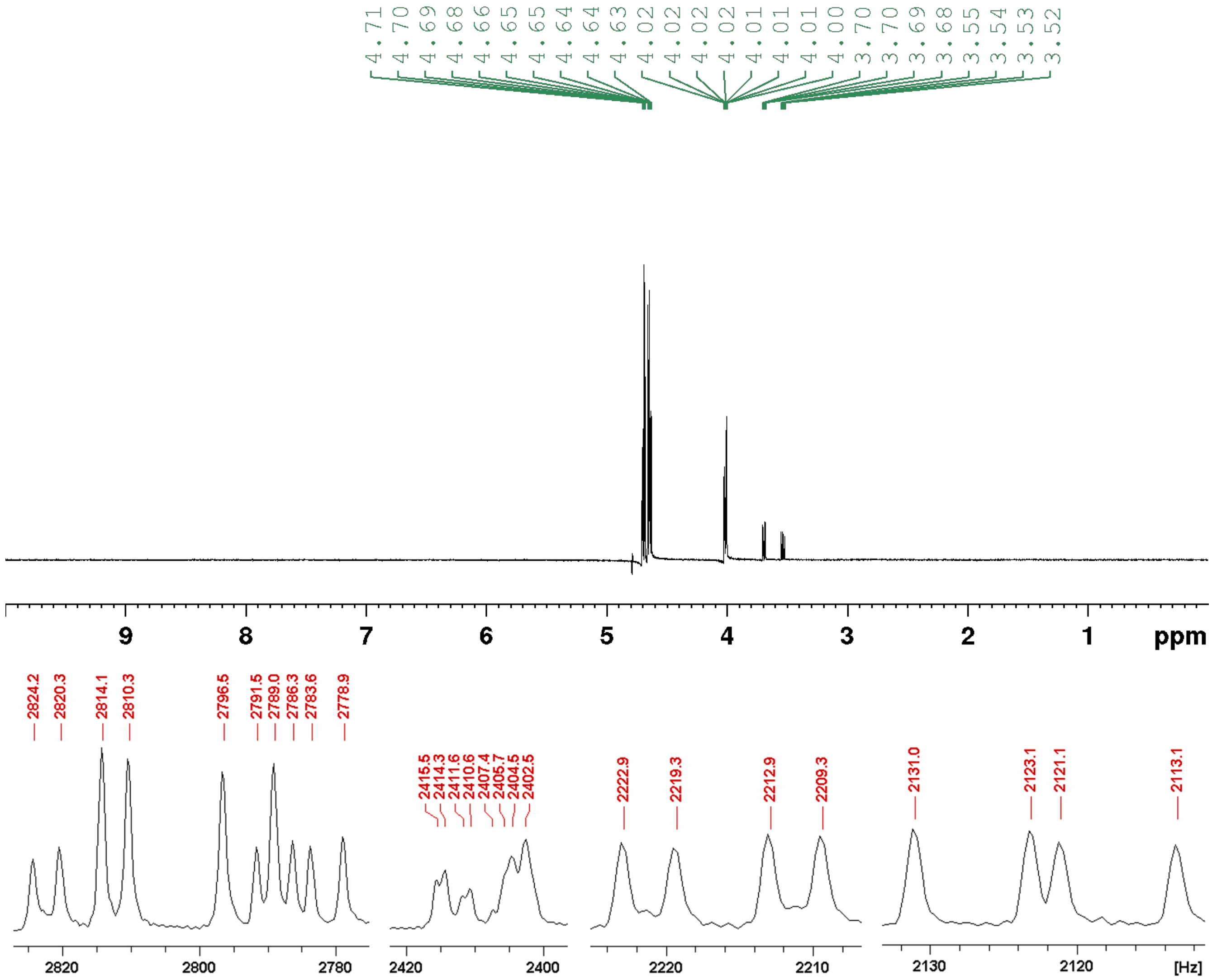

7.7.8 FDGal-6 (**7**): α-furanose form (α-*f*-FDGal-6): **$^{1}H\{^{19}F\}$ SRI-FESTA NMR** (600 MHz, D_2O, $\delta^{19}F$ = -230.79 ppm, $\delta^{1}H$ = 4.00 ppm, τ_m = 100 ms)

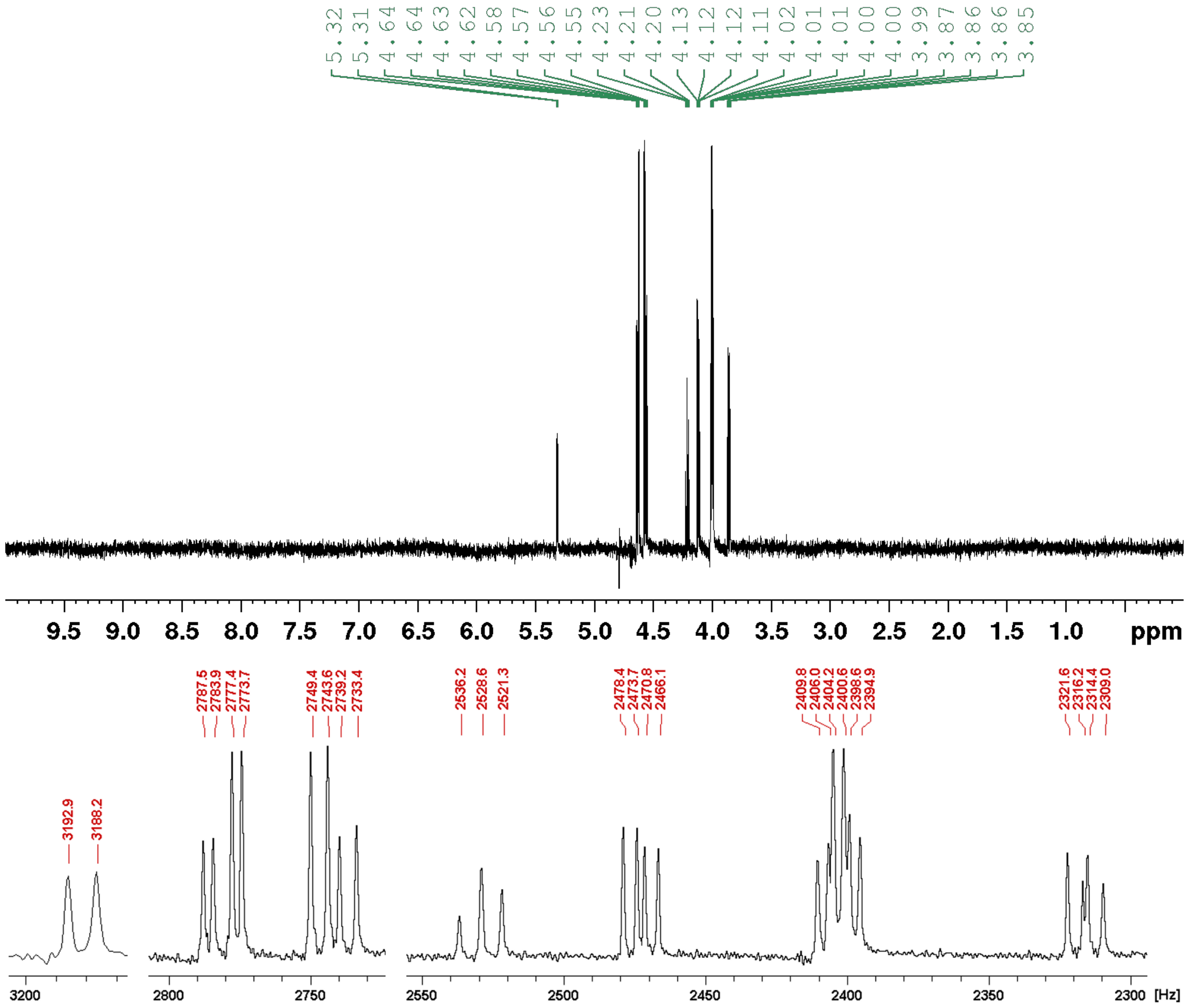

7.7.9 FDGal-6 (**7**): β-furanose form (β-*f*-FDGal-6): **^{1}H{^{19}F} SRI-FESTA NMR** (600 MHz, D_2O, δ^{19}F = -229.26 ppm, δ^1H = 4.07 ppm, τ_m = 200 ms)

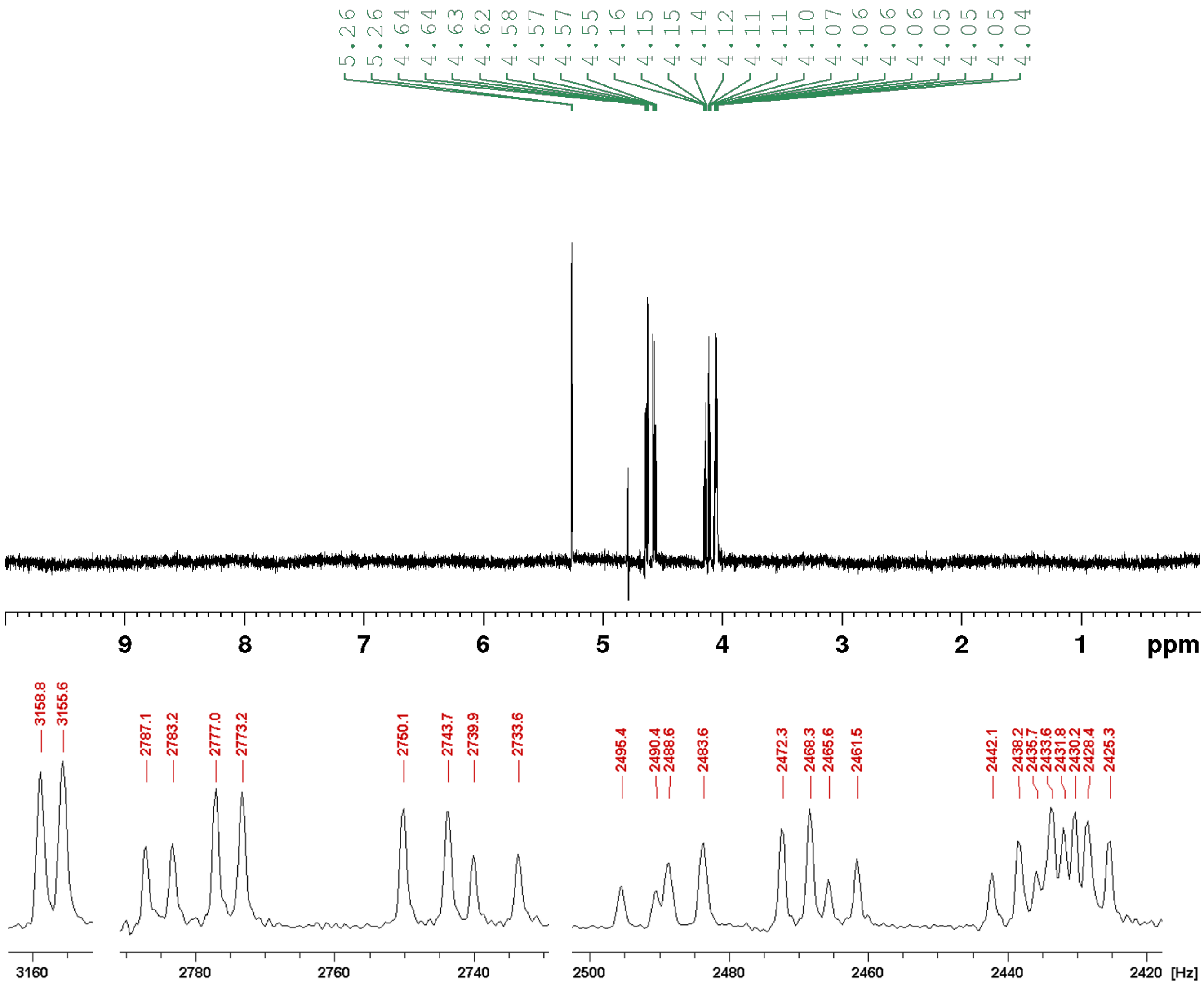

7.8 4,6-Dideoxy-4,6-difluoro-D-galactose (8, FDGal-46): 44 : 56 α-pyranose / β-pyranose, in D_2O.

7.8.1 FDGal-46 (**8**): ^{1}H NMR (600 MHz, D_2O)

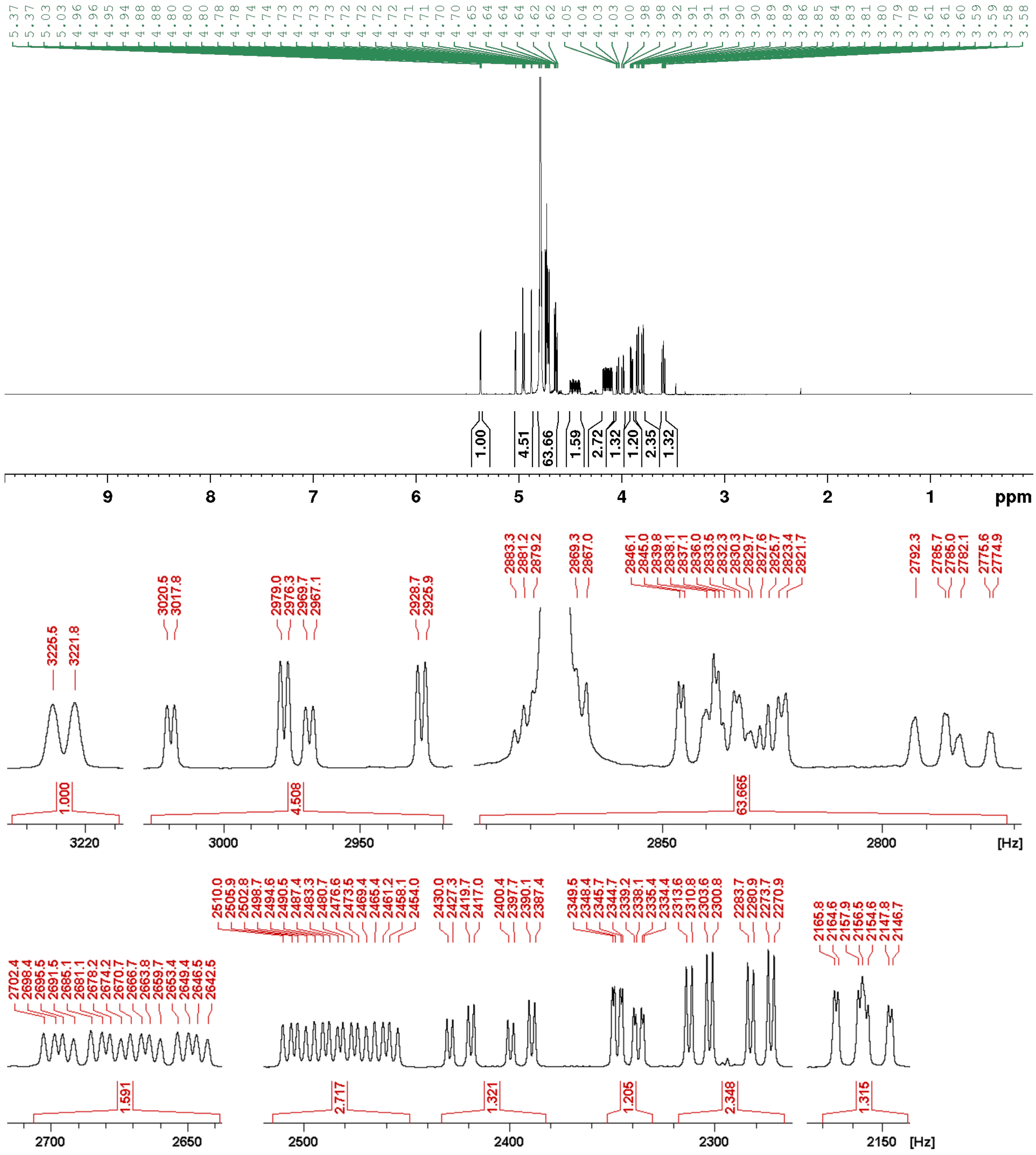

7.8.2 FDGal-46 (**8**): **^{1}H{^{19}F} NMR** (600 MHz, D_2O)

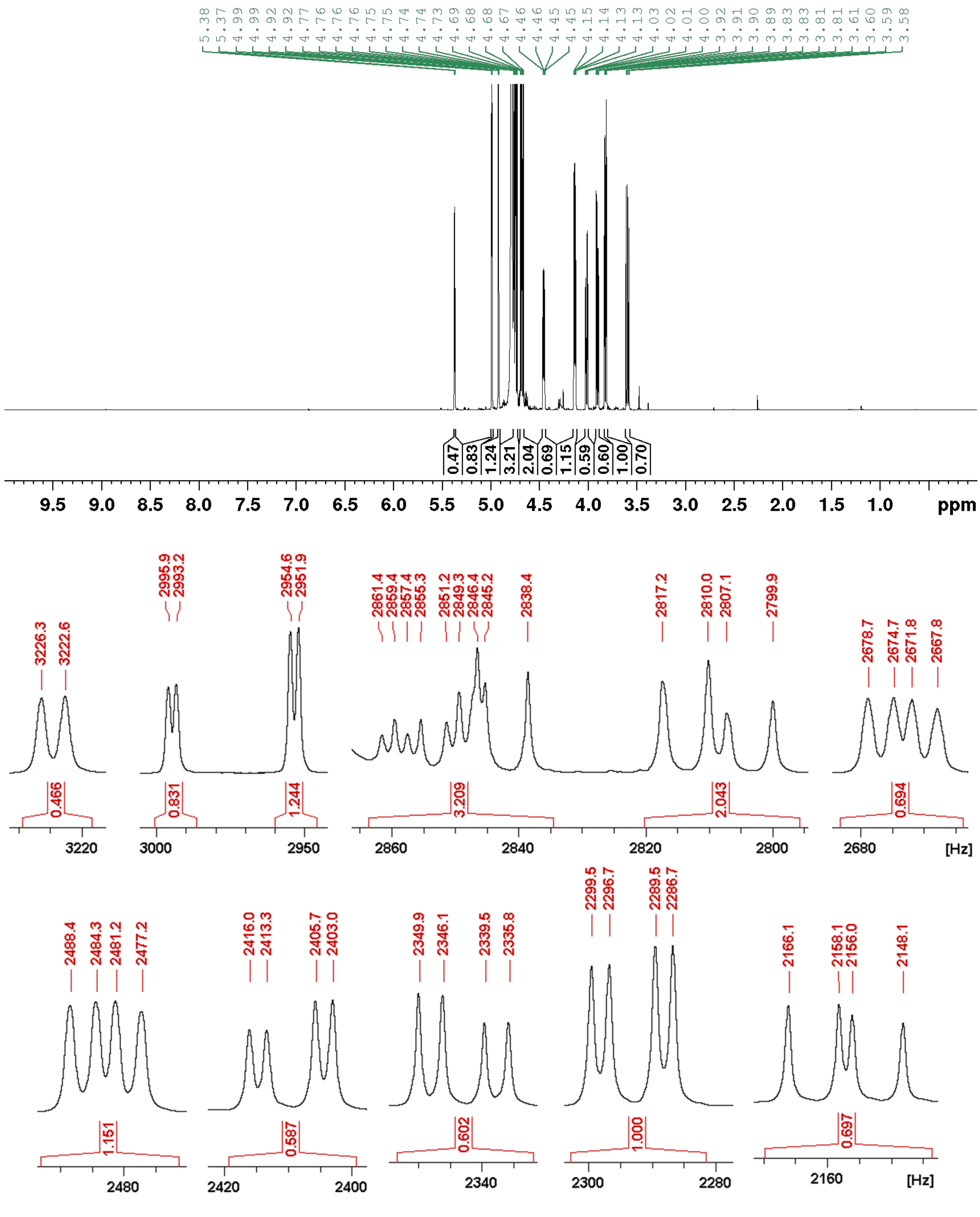

7.8.3 FDGal-46 (**8**): ^{19}**F NMR** (565 MHz, D_2O)

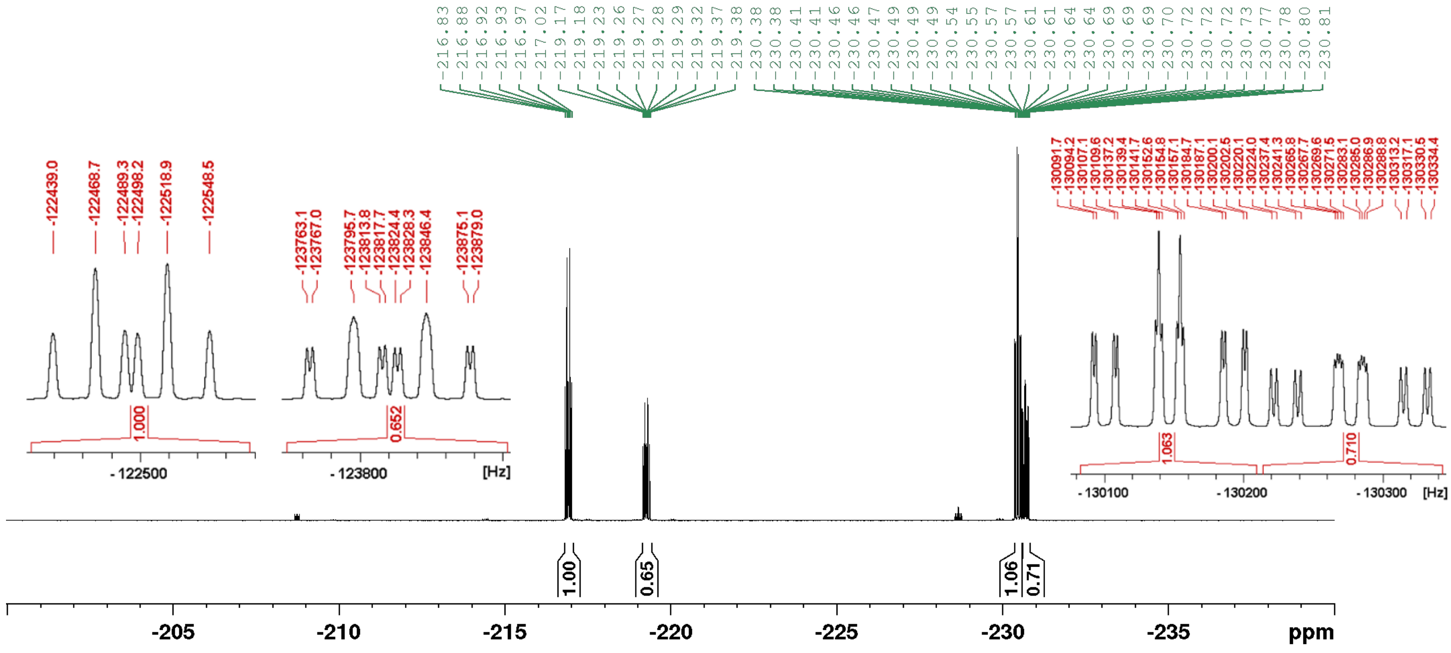

7.8.4 FDGal-46 (**8**): ^{19}**F{^{1}H} NMR** (565 MHz, D_2O)

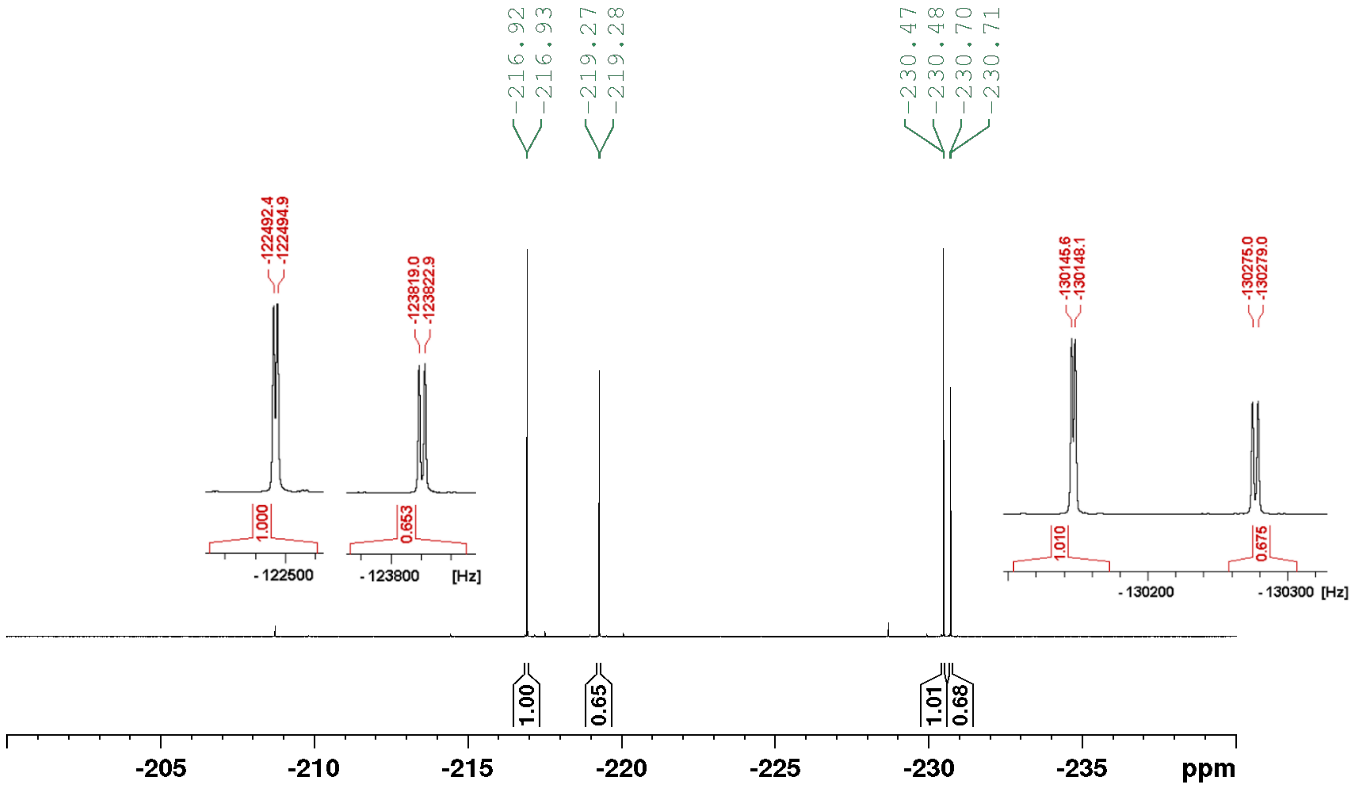

7.8.5 FDGal-46 (**8**): **^{1}H-^{1}H COSY** (500 MHz, D_2O)

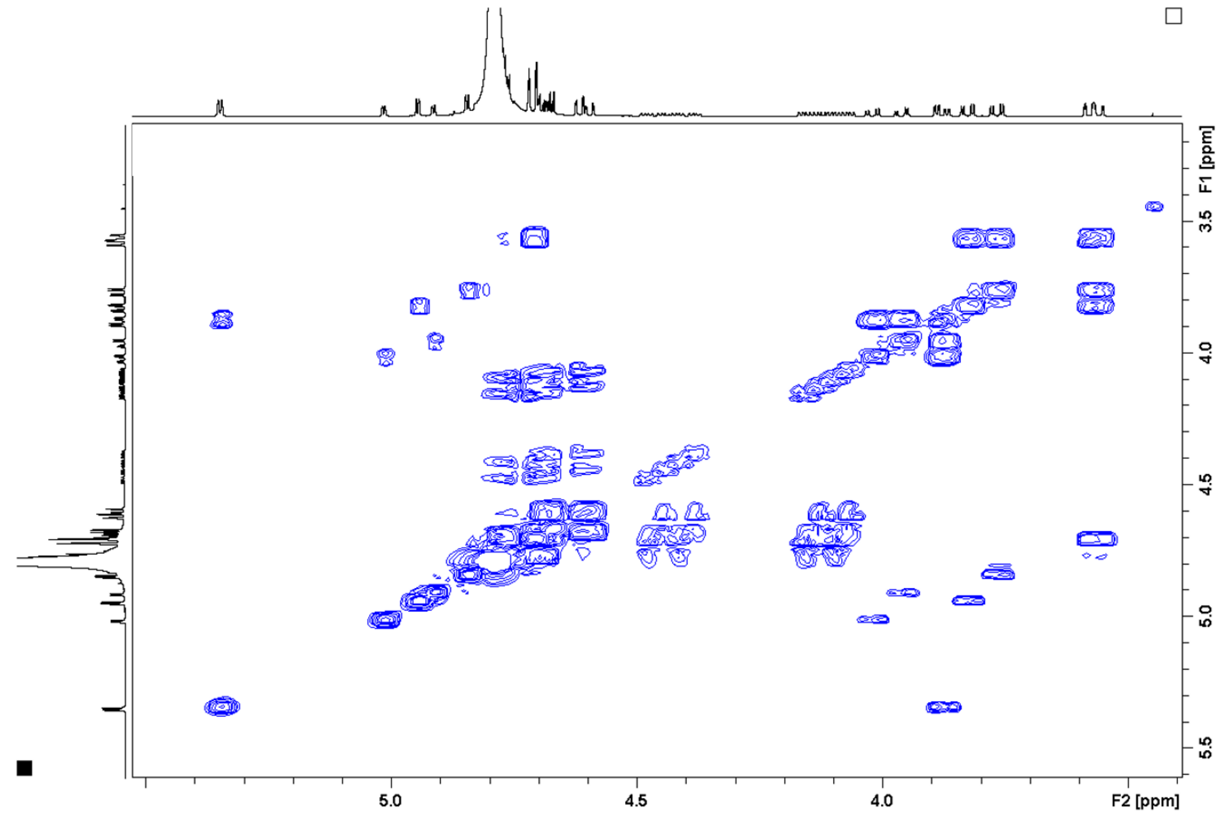

7.8.6 FDGal-46 (**8**): **^{1}H-^{1}H{^{19}F} COSY** (500 MHz, D_2O)

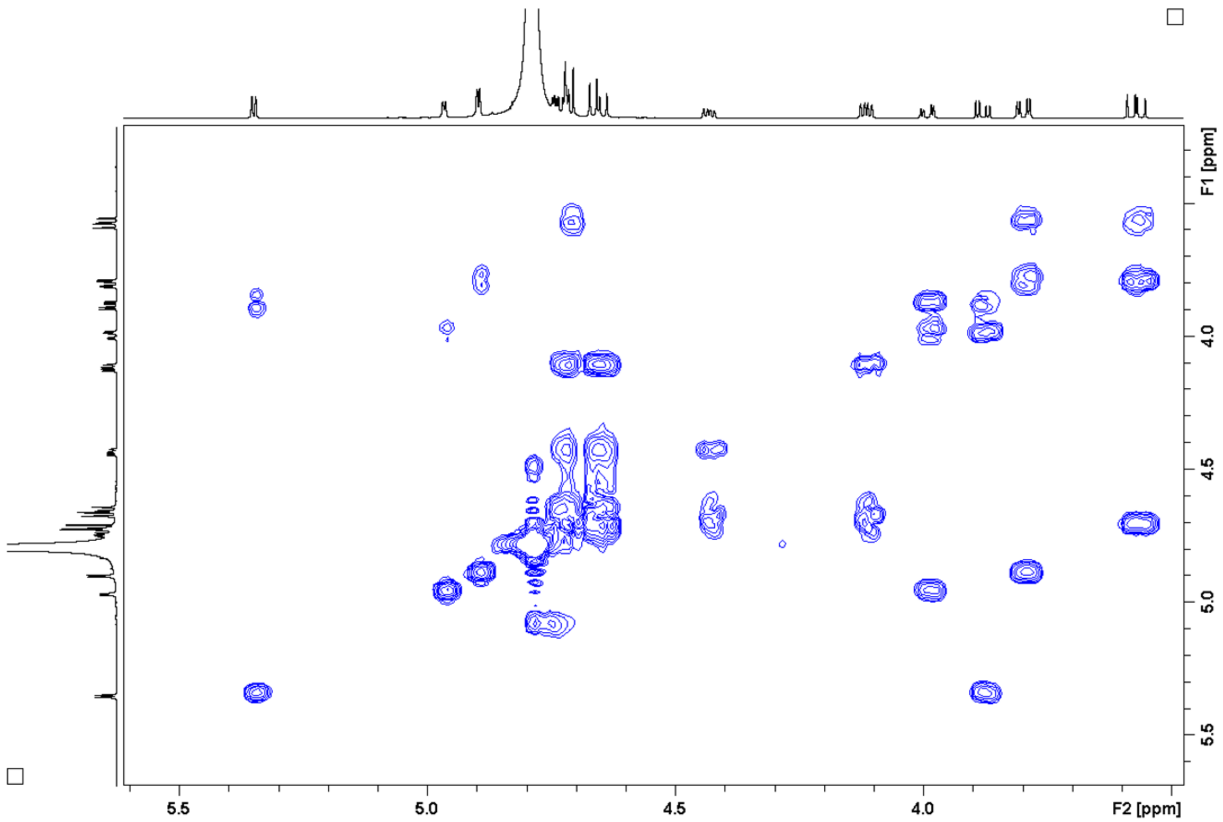

7.8.7 FDGal-46 (**8**): α-pyranose form (α-*p*-FDGal-46): **^{1}H{^{19}F} SRI-FESTA NMR** (600 MHz, D$_2$O, a) δ^{19}F = -219.27 ppm, δ^{1}H = 4.99 ppm, τ_m = 100 ms; b) δ^{19}F = -230.48 ppm, δ^{1}H = 4.45 ppm, τ_m = 100 ms)

a)

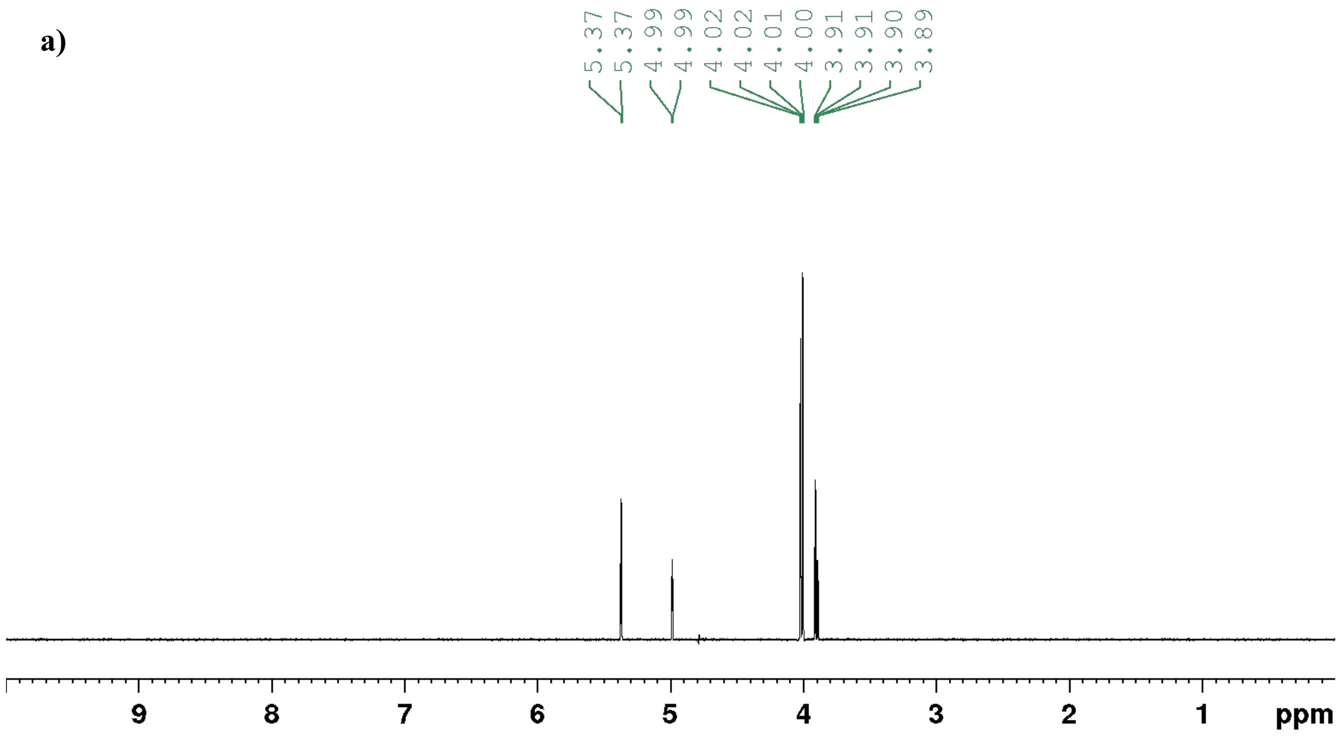

b)

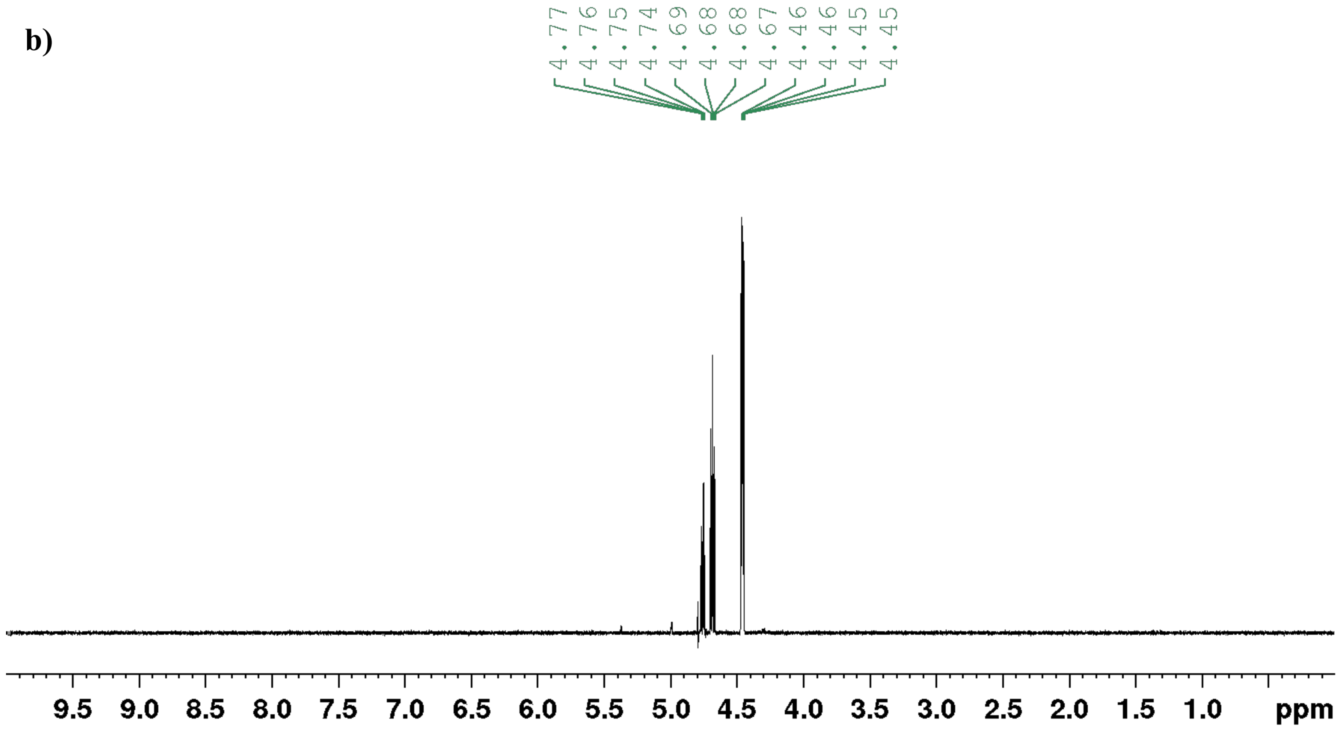

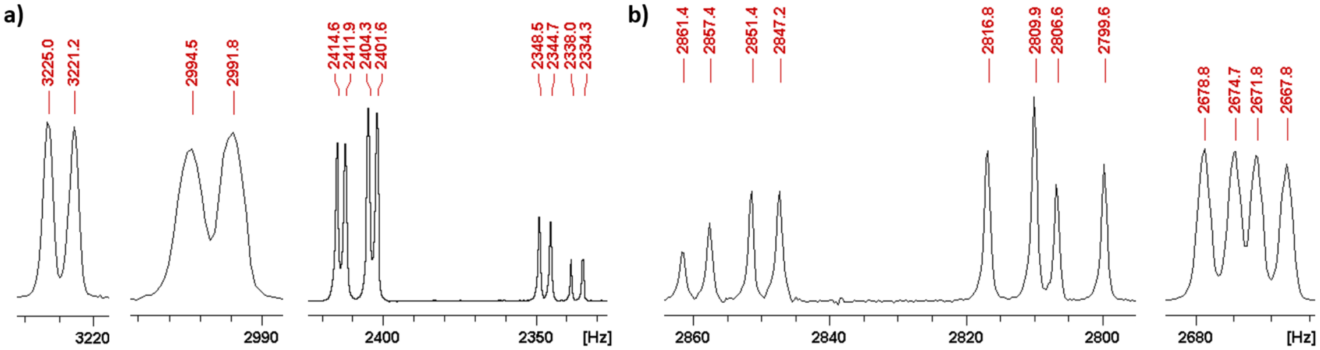

7.8.8 FDGal-46 (**8**): β-pyranose form (β-*p*-FDGal-46): **^{1}H{^{19}F} SRI-FESTA NMR** (600 MHz, D_2O, a) δ^{19}F = -216.93 ppm, δ^{1}H = 3.82 ppm, τ_m = 100 ms; b) δ^{19}F = -230.48 ppm, δ^{1}H = 4.14 ppm, τ_m = 100 ms)

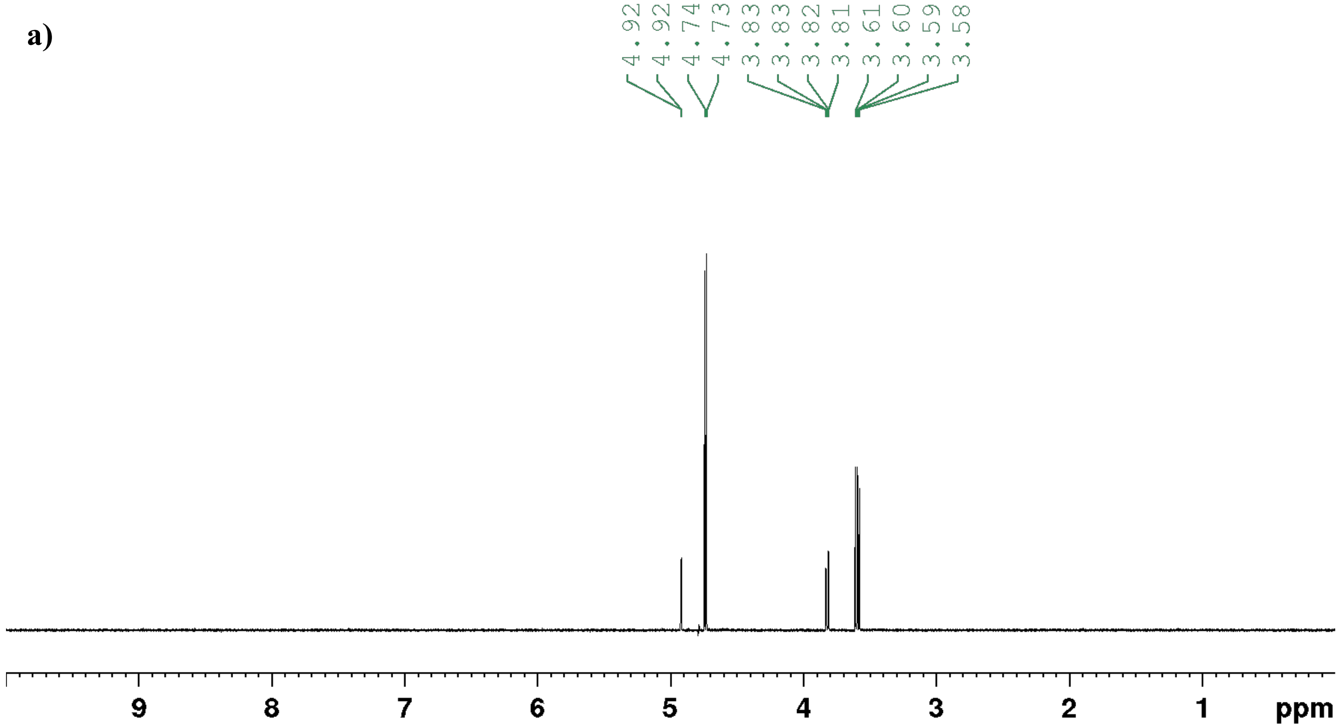

b)

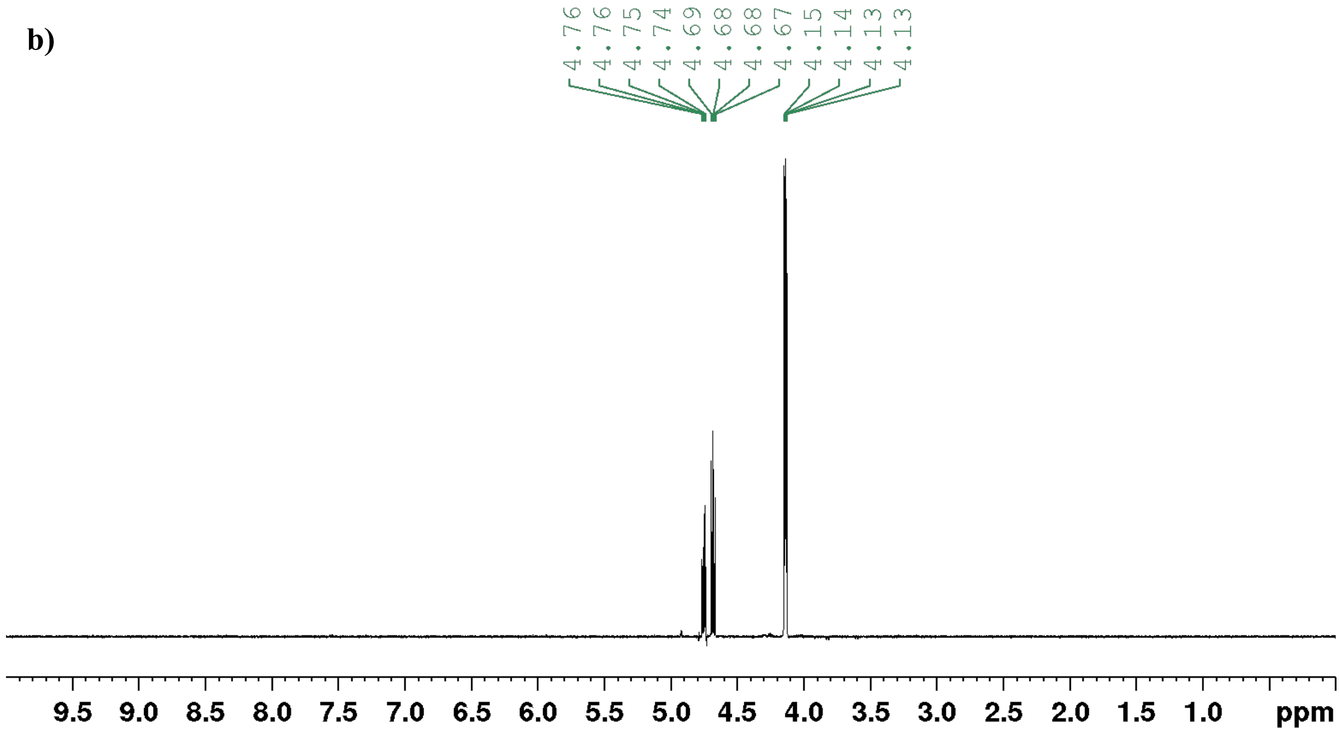

4.76
4.76
4.75
4.74
4.69
4.68
4.68
4.67
4.15
4.14
4.13
4.13
9.5 9.0 8.5 8.0 7.5 7.0 6.5 6.0 5.5 5.0 4.5 4.0 3.5 3.0 2.5 2.0 1.5 1.0 ppm

a)

b)

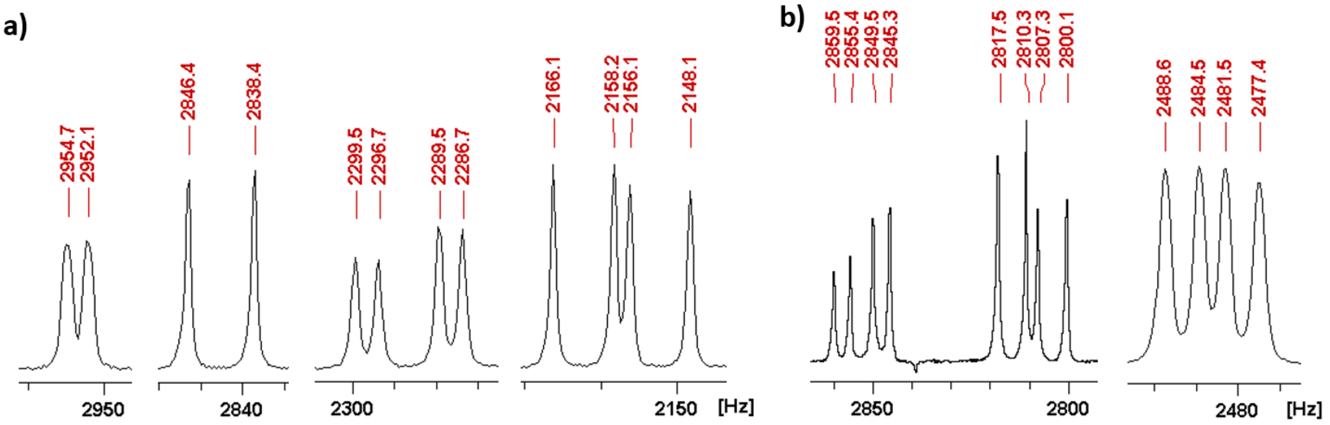

2954.7
2952.1
2846.4
2838.4
2299.5
2296.7
2289.5
2286.7
2166.1
2158.2
2156.1
2148.1
2950
2840
2300
2150
[Hz]
2859.5
2855.4
2849.5
2845.3
2817.5
2810.3
2807.3
2800.1
2488.6
2484.5
2481.5
2477.4
2850
2800
2480
[Hz]

7.9 2-Deoxy-2-fluoro-D-mannose (9, FDMan-2): 66 : 34 α-pyranose / β-pyranose, in D_2O.

7.9.1 FDMan-2 (**9**): **^{1}H NMR** (600 MHz, D_2O)

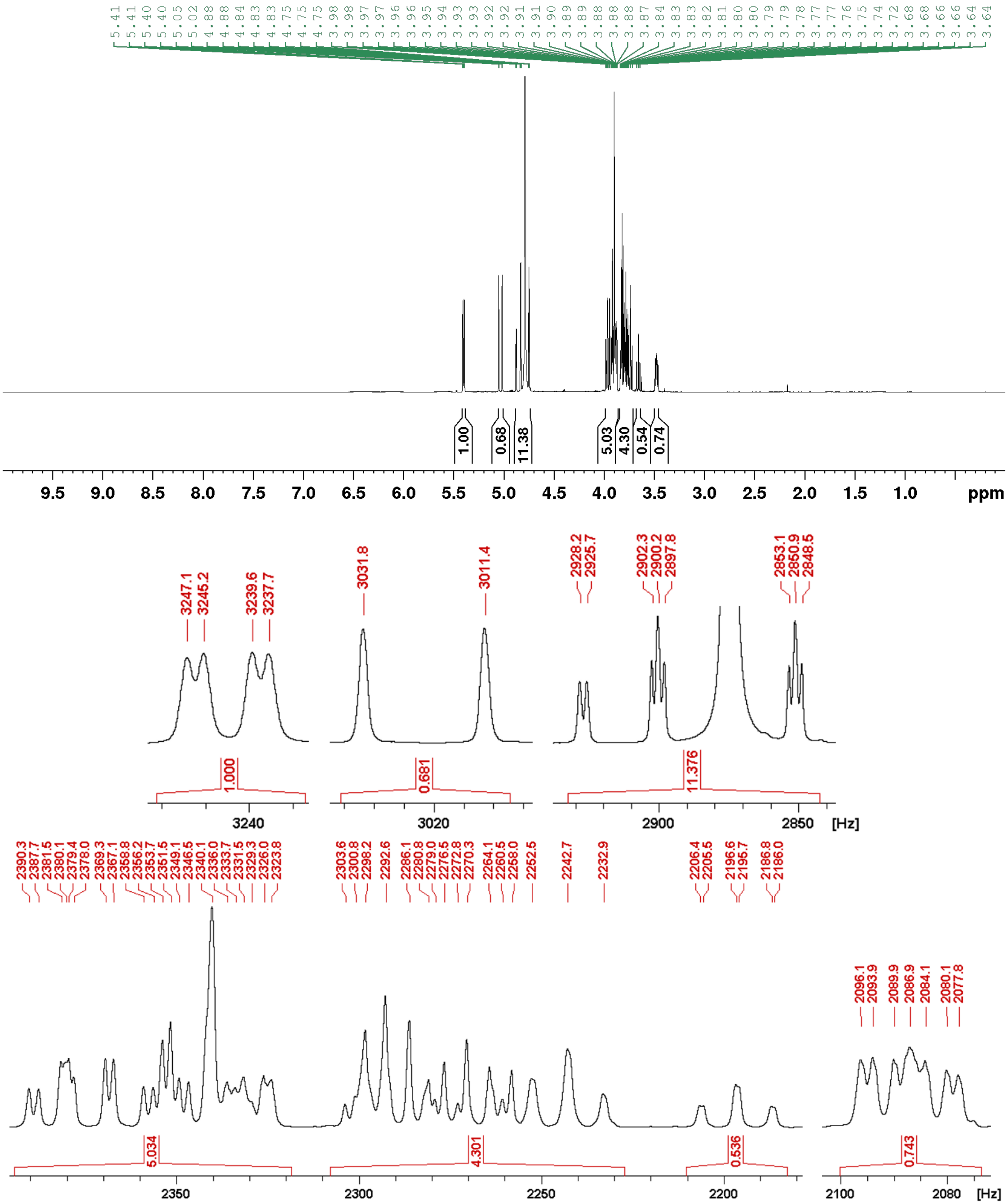

7.9.2 FDMan-2 (**9**): ^{1}**H{^{19}F} NMR** (600 MHz, D_2O)

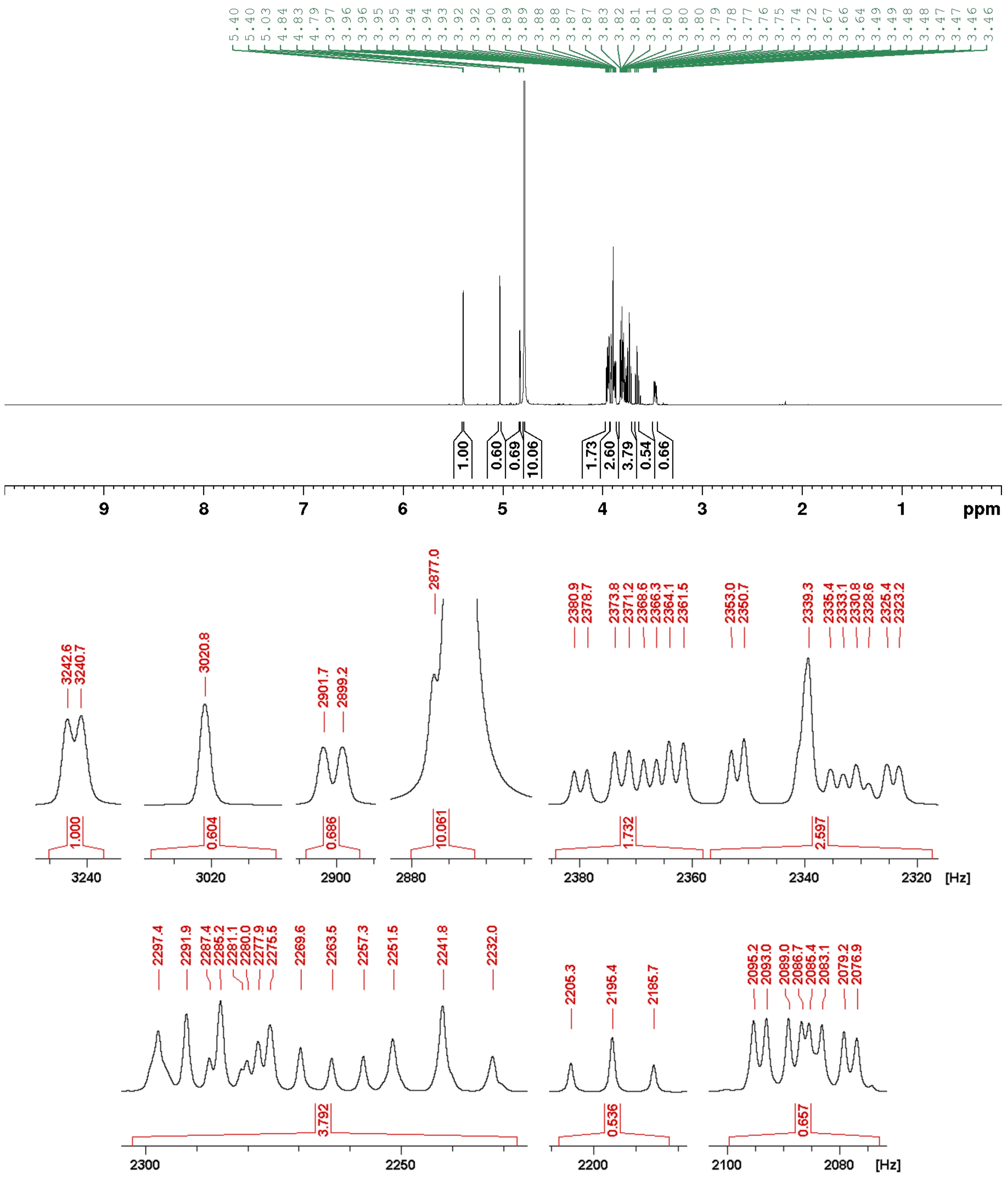

7.9.3 FDMan-2 (**9**): **^{19}F NMR** (565 MHz, D_2O)

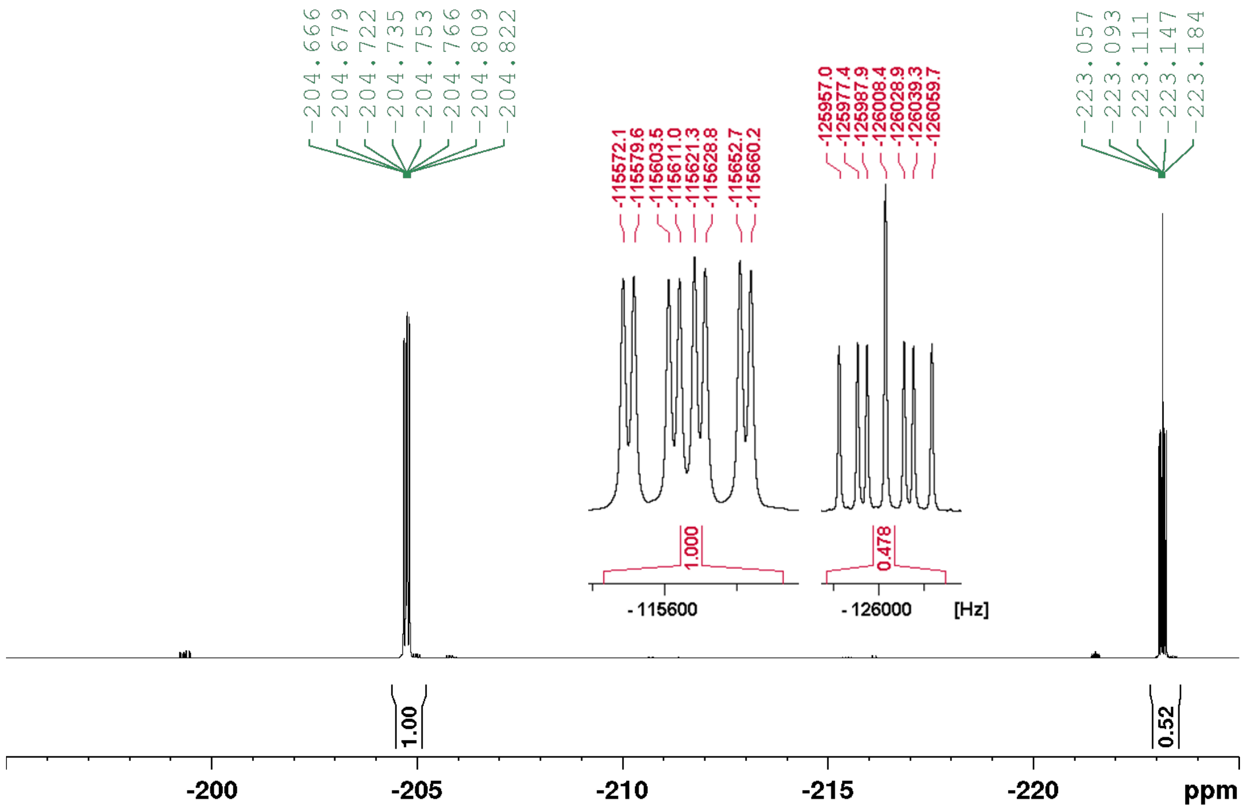

7.9.4 FDMan-2 (**9**): **^{19}F{^{1}H} NMR** (565 MHz, D_2O)

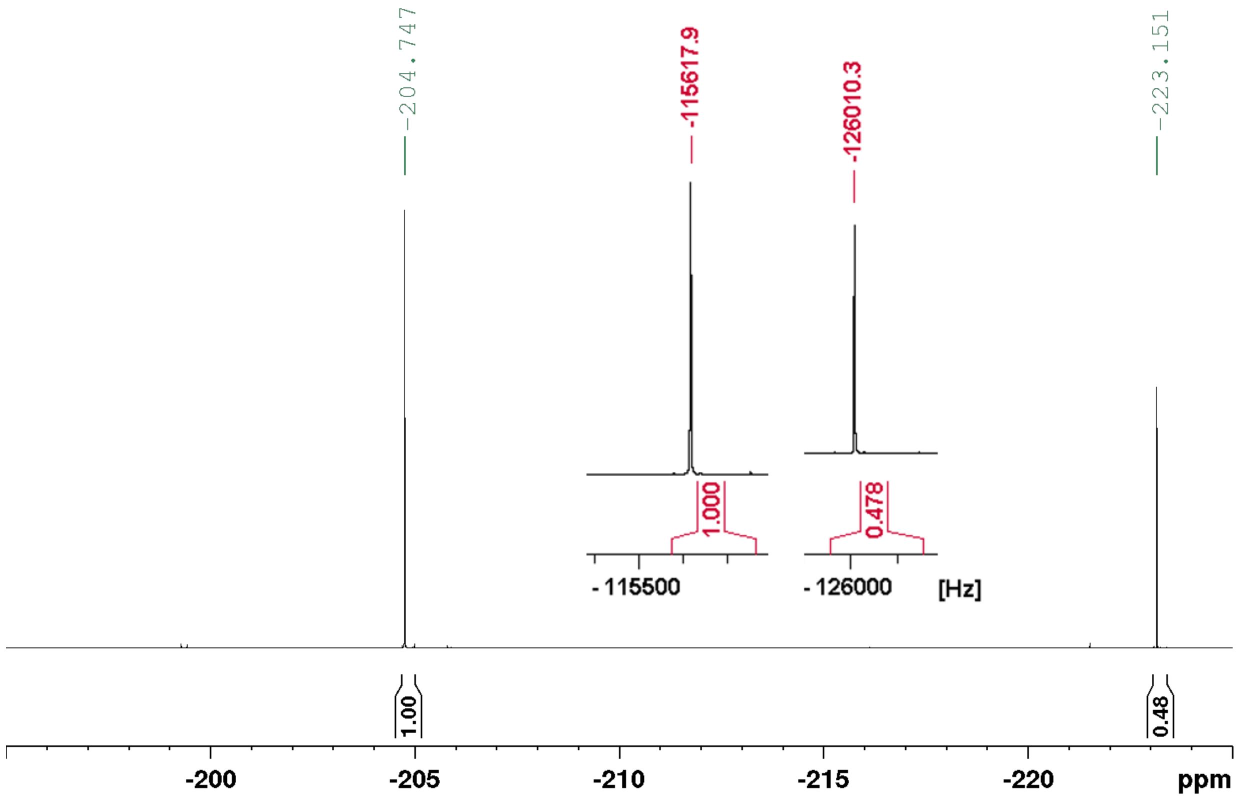

7.9.5 FDMan-2 (**9**): **^{1}H-^{1}H COSY** (500 MHz, D_2O)

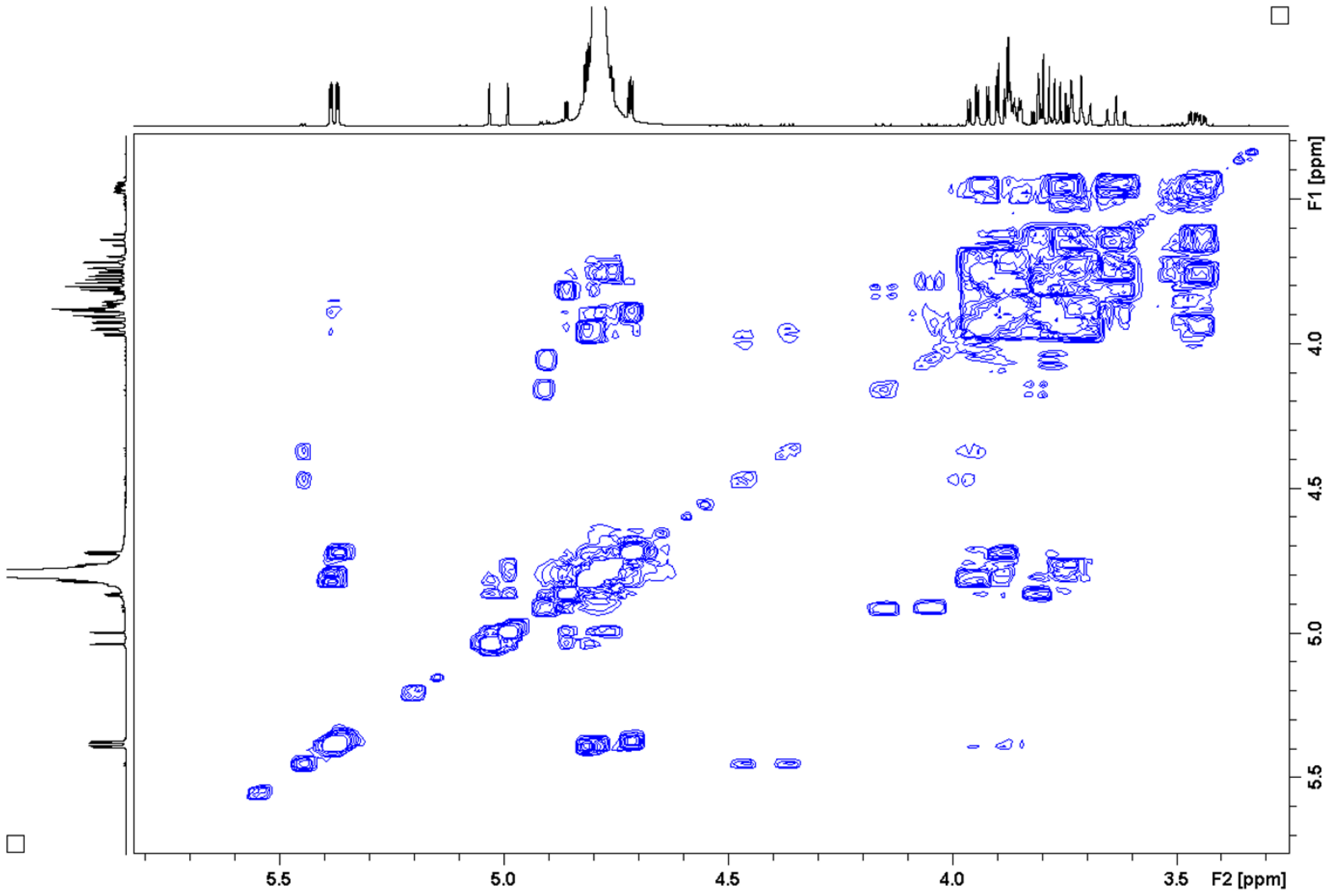

7.9.6 FDMan-2 (**9**): α-pyranose form (α-*p*-FDGMan-2): **^{1}H{^{19}F} SRI-FESTA NMR** (600 MHz, D_2O, δ^{19}F = -204.75 ppm, δ^1H = 3.94 ppm, τ_m = 300 ms)

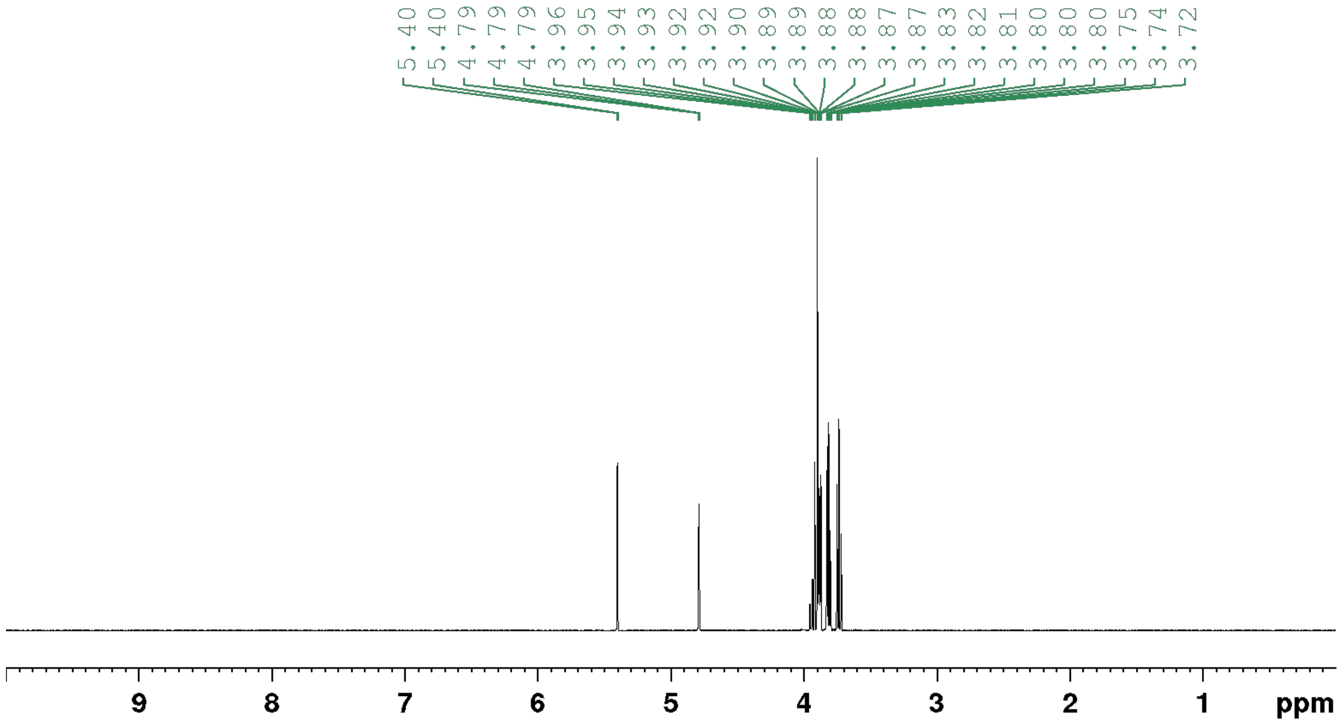

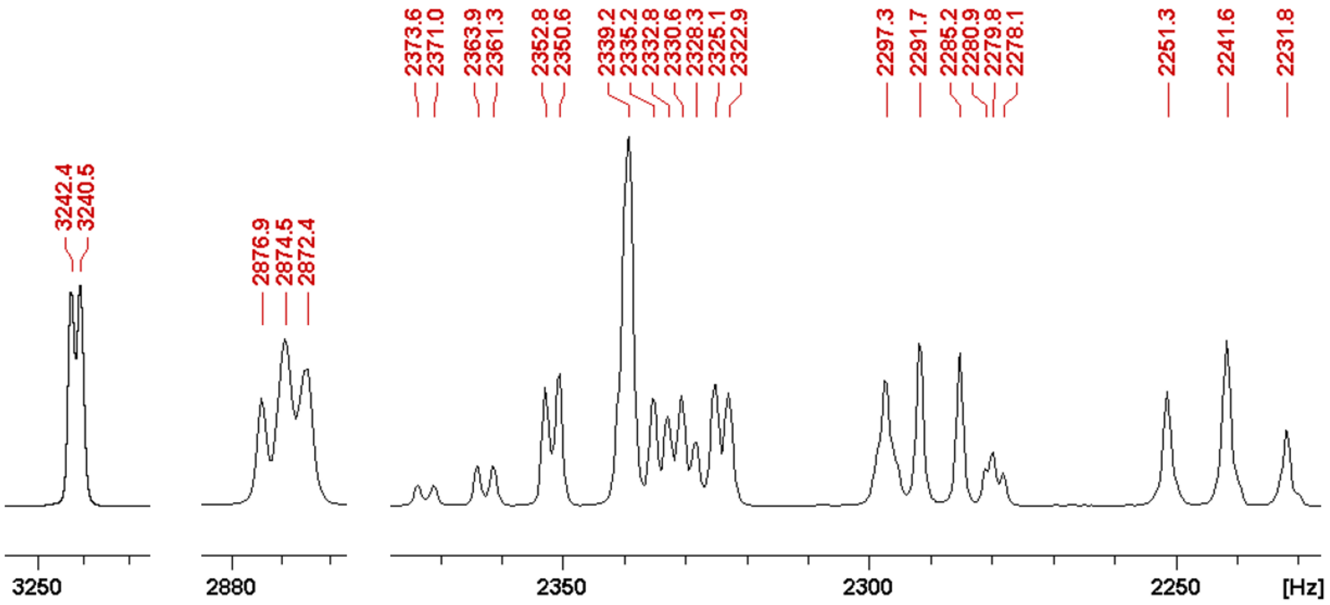

3242.4
3240.5
2876.9
2874.5
2872.4
2373.6
2371.0
2363.9
2361.3
2352.8
2350.6
2339.2
2335.2
2332.8
2330.6
2328.3
2325.1
2322.9
2297.3
2291.7
2285.2
2280.9
2279.8
2278.1
2251.3
2241.6
2231.8
3250
2880
2350
2300
2250
[Hz]

7.9.7 FDMan-2 (**9**): β-pyranose form (β-*p*-FDMan-2): **^{1}H{^{19}F} SRI-FESTA NMR** (600 MHz, D_2O, δ^{19}F = -223.15 ppm, δ^1H = 4.83, τ_m = 200 ms)

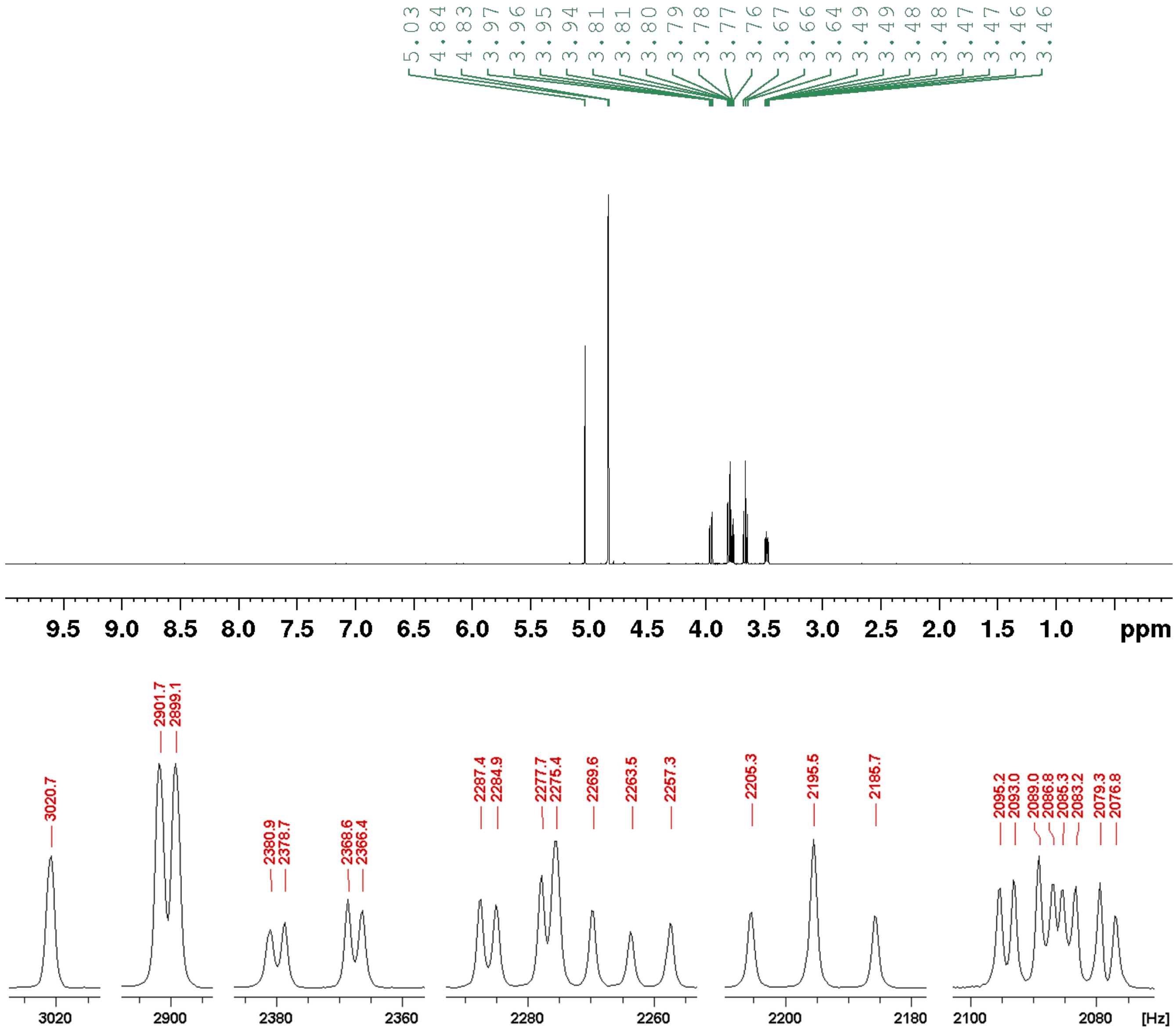

7.10 3-Deoxy-3-fluoro-D-allose (10, FDAll-3): 10.0 : 88.9 : 0.6 : 0.5 α-pyranose / β-pyranose / α-furanose / β-furanose, in D_2O.

7.10.1 FDAll-3 (**10**): **^{1}H NMR** (600 MHz, D_2O)

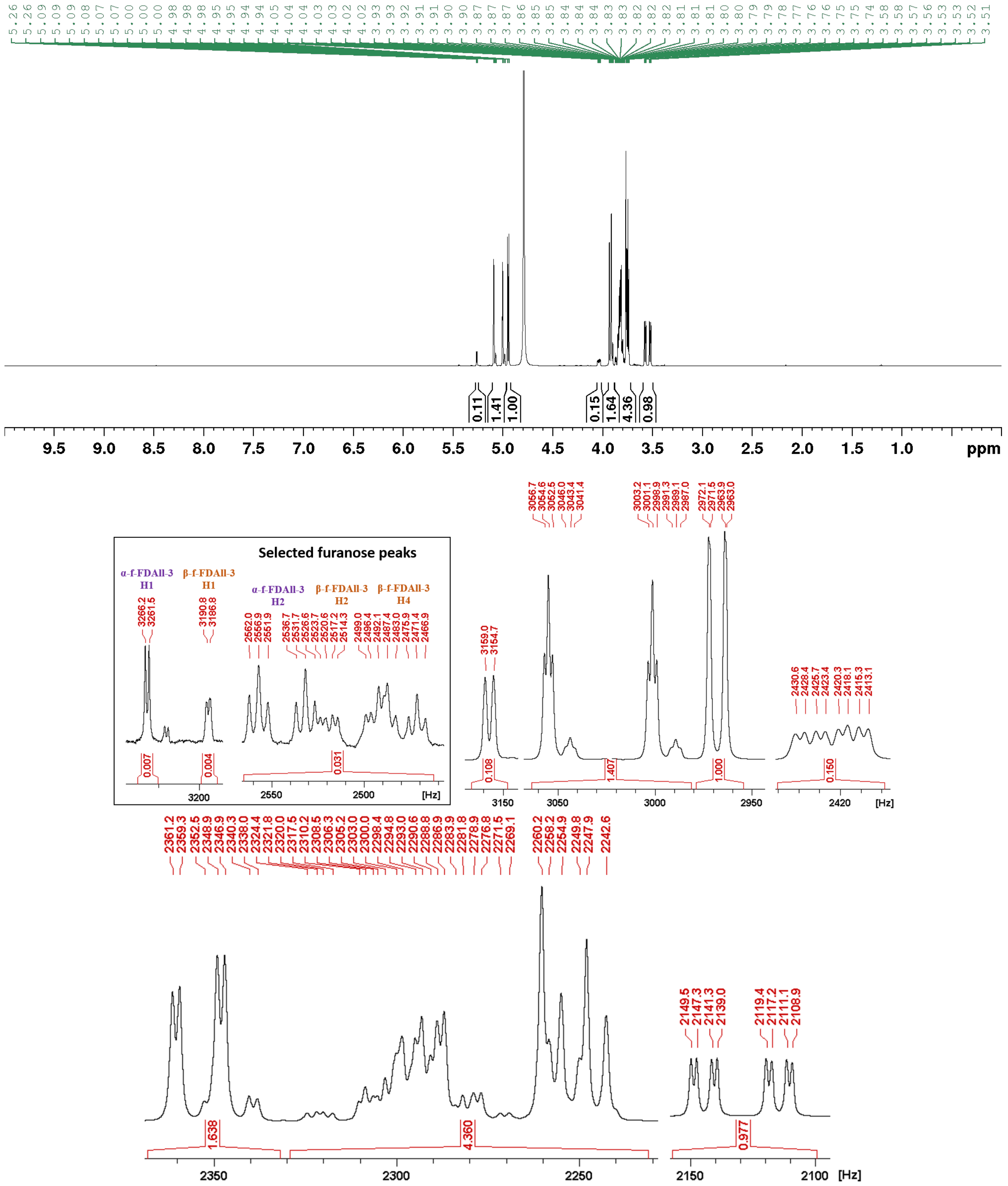

7.10.2 FDAll-3 (**10**): ^{1}**H{^{19}F} NMR** (600 MHz, D_2O)

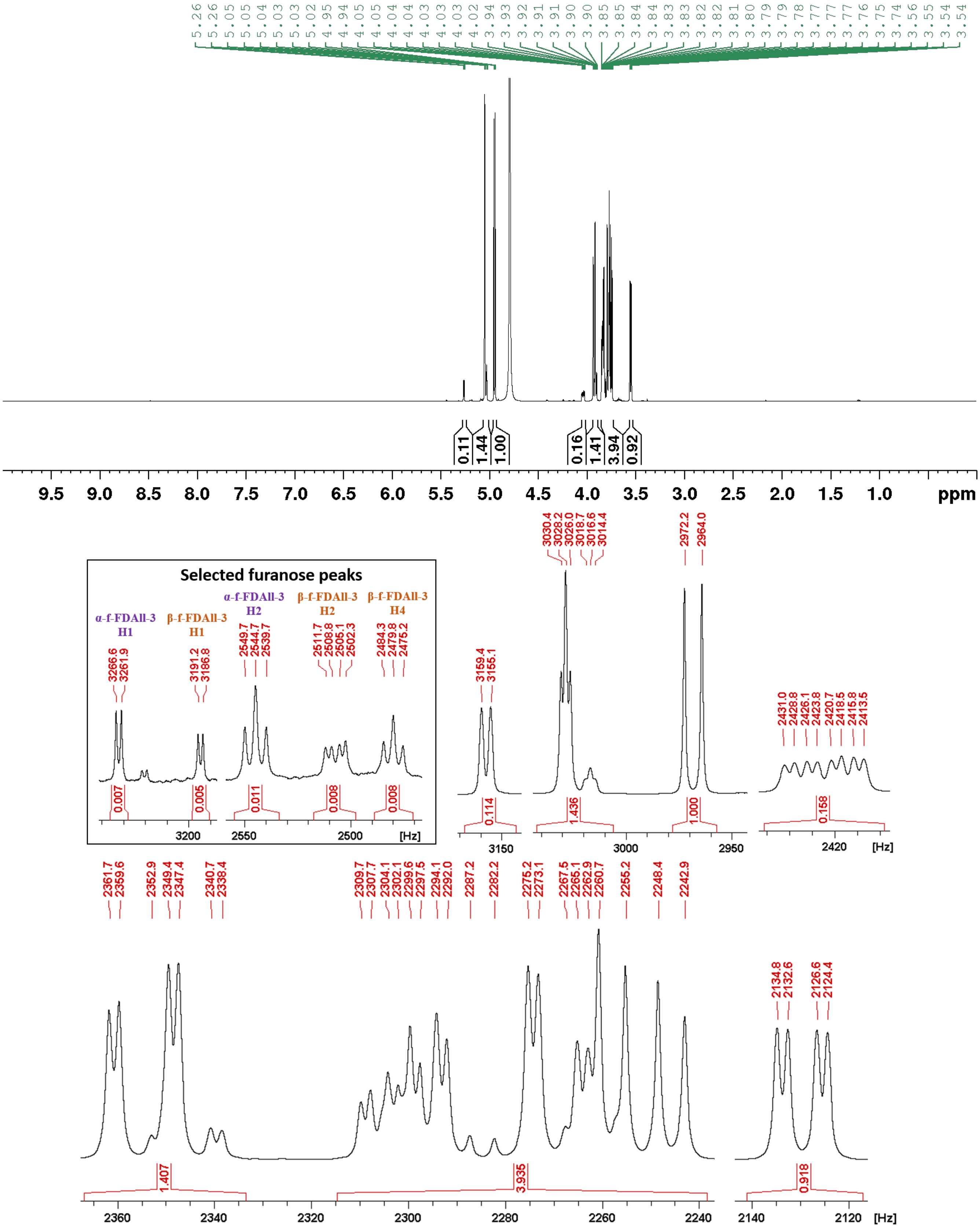

7.10.3 FDAll-3 (**10**): ^{19}**F NMR** (565 MHz, D_2O)

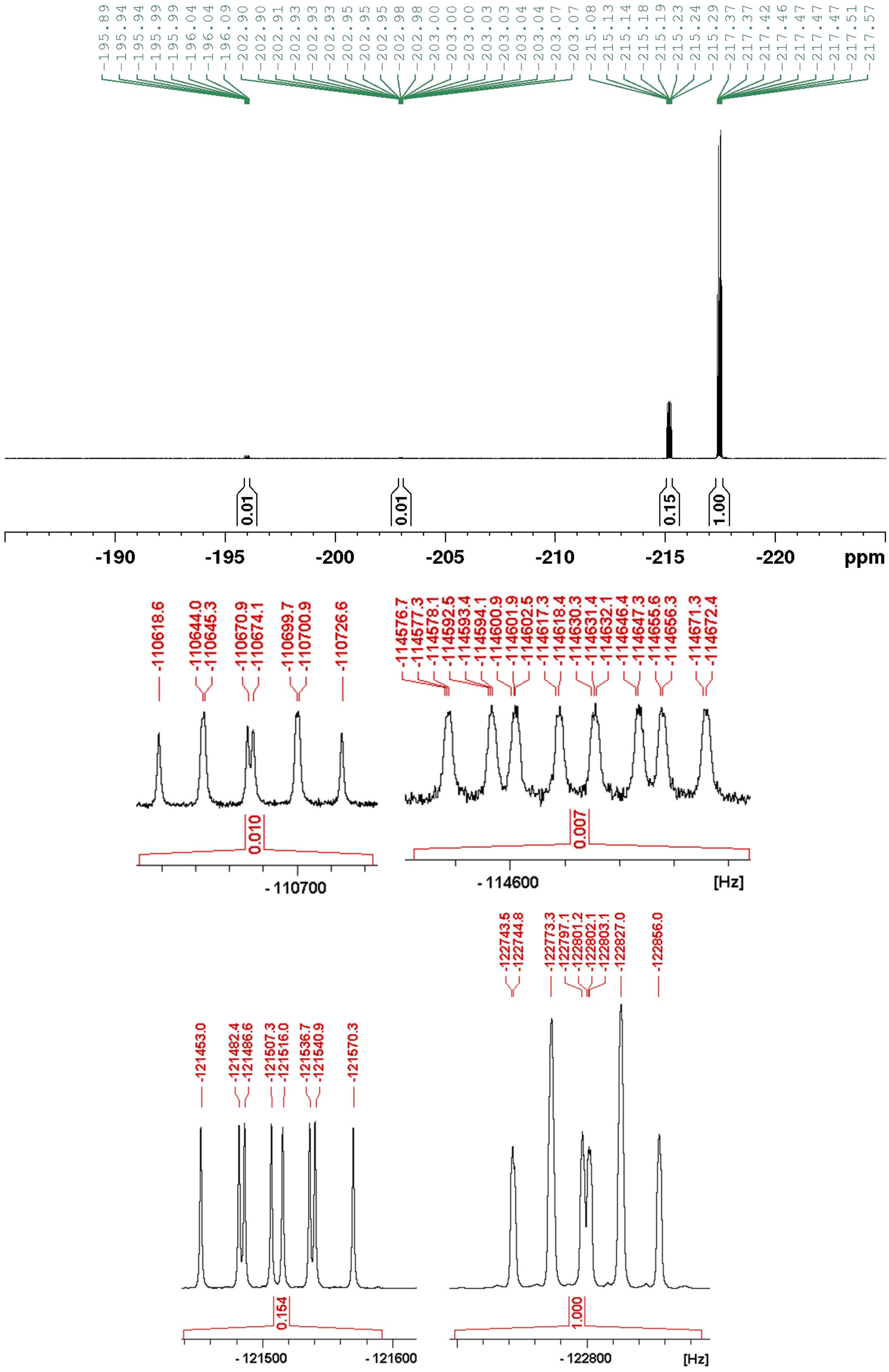

7.10.4 FDAll-3 (**10**): **$^{19}F\{^{1}H\}$ NMR** (565 MHz, D_2O)

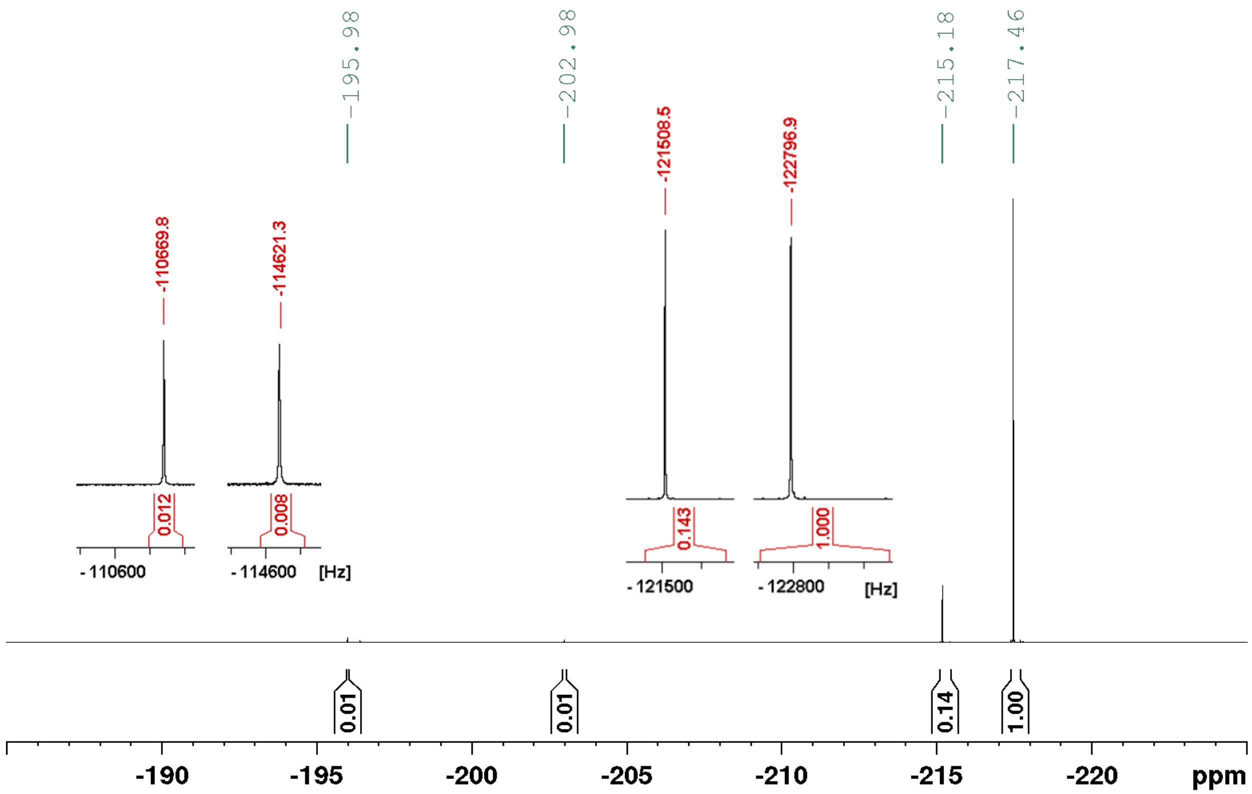

7.10.5 FDAll-3 (**10**): **^{1}H-$^{1}H\{^{19}F\}$ COSY** (600 MHz, D_2O)

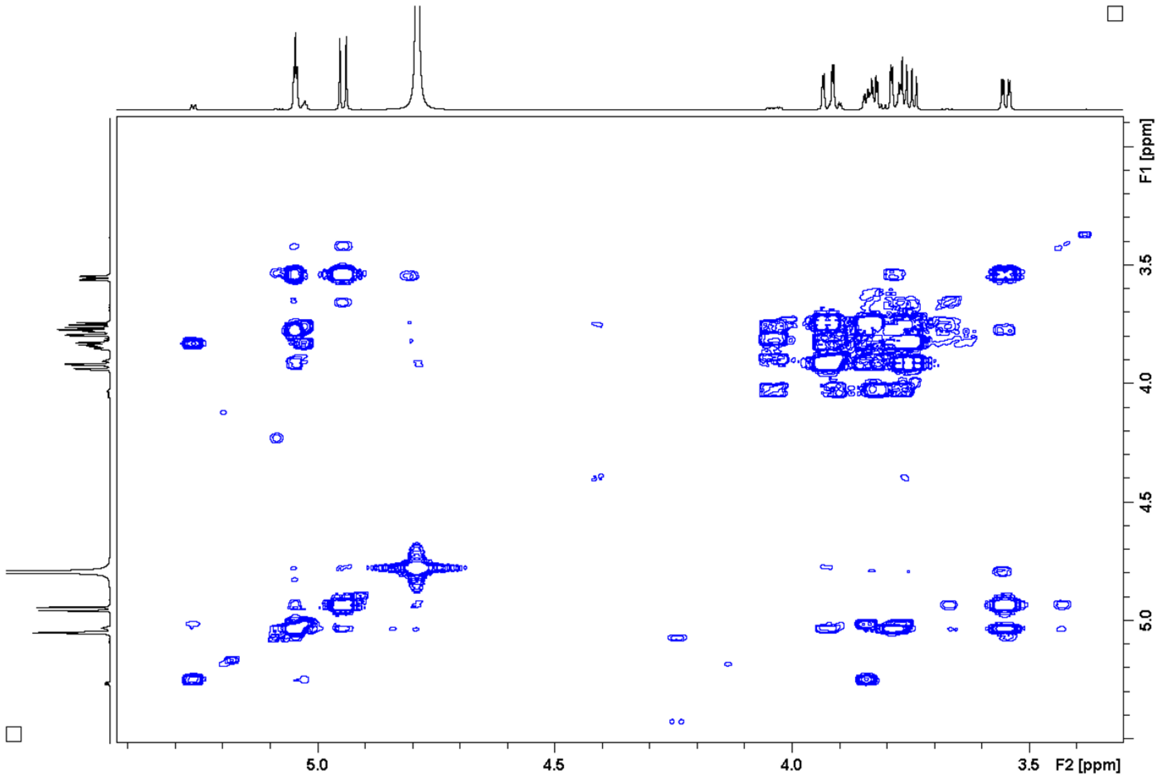

7.10.6 FDAll-3 (**10**): **^{1}H-^{13}C HSQC** (600 MHz, D_2O)

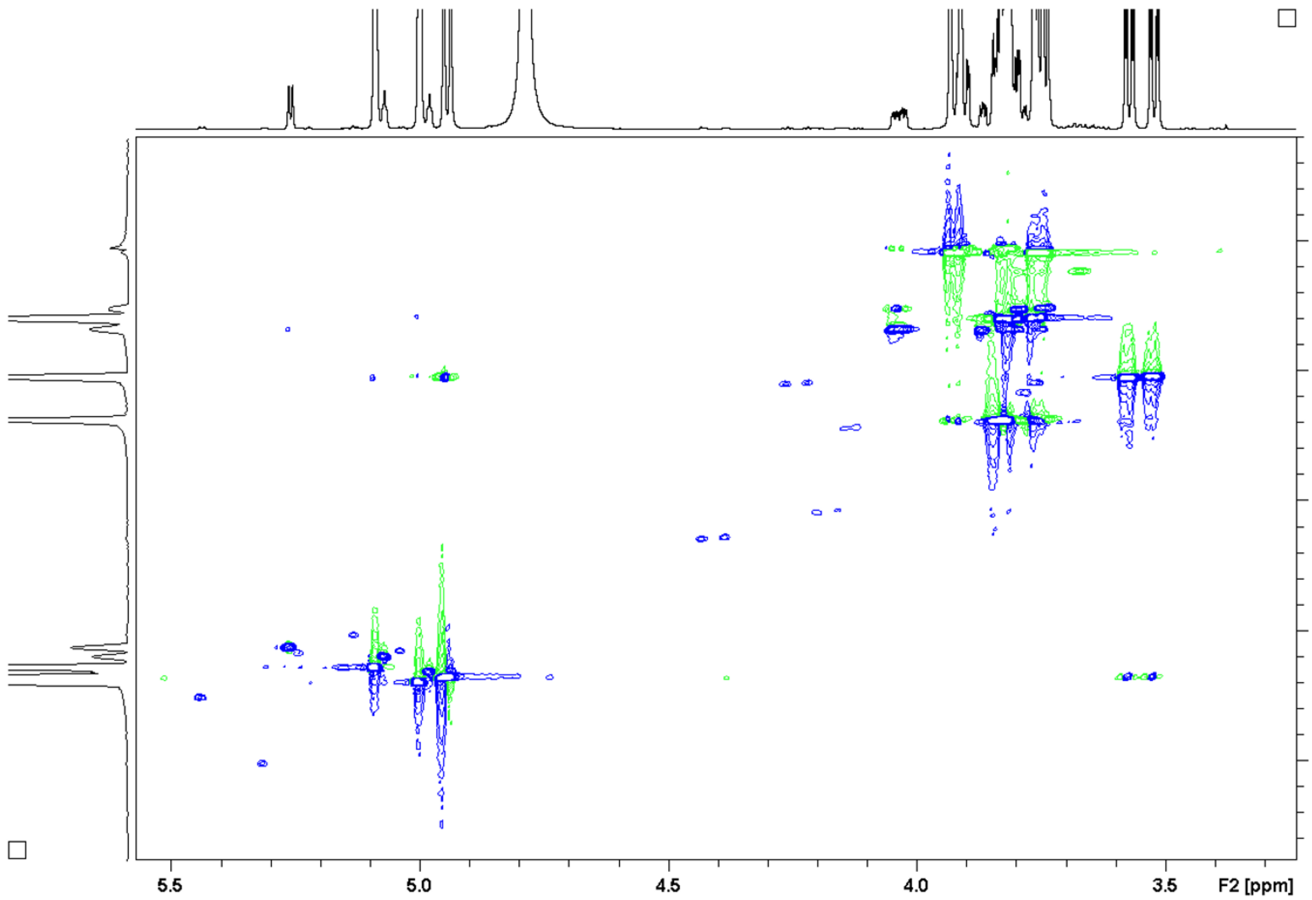

7.10.7 FDAll-3 (**10**): α-pyranose form (α-*p*-FDAll-3): **^{1}H{^{19}F} SRI-FESTA NMR** (600 MHz, D_2O, $\delta^{19}F$ = -215.18 ppm, $\delta^{1}H$ = 5.03 ppm, τ_m = 100 ms)

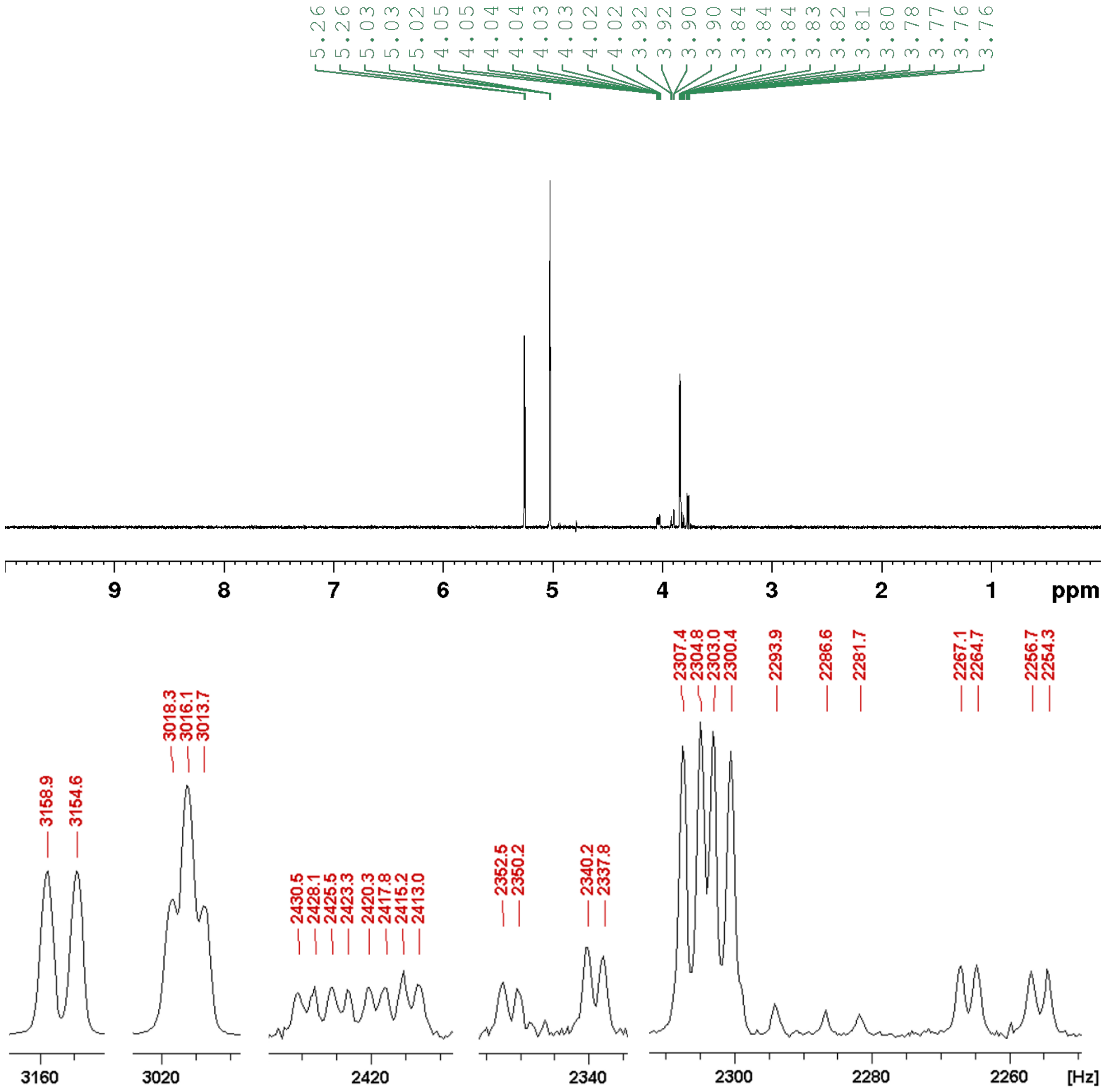

7.10.8 FDAll-3 (**10**): β-pyranose form (β-*p*-FDAll-3): **$^{1}H\{^{19}F\}$ SRI-FESTA NMR** (600 MHz, D_2O, $\delta^{19}F$ = -217.46 ppm, $\delta^{1}H$ = 5.05 ppm, τ_m = 200 ms)

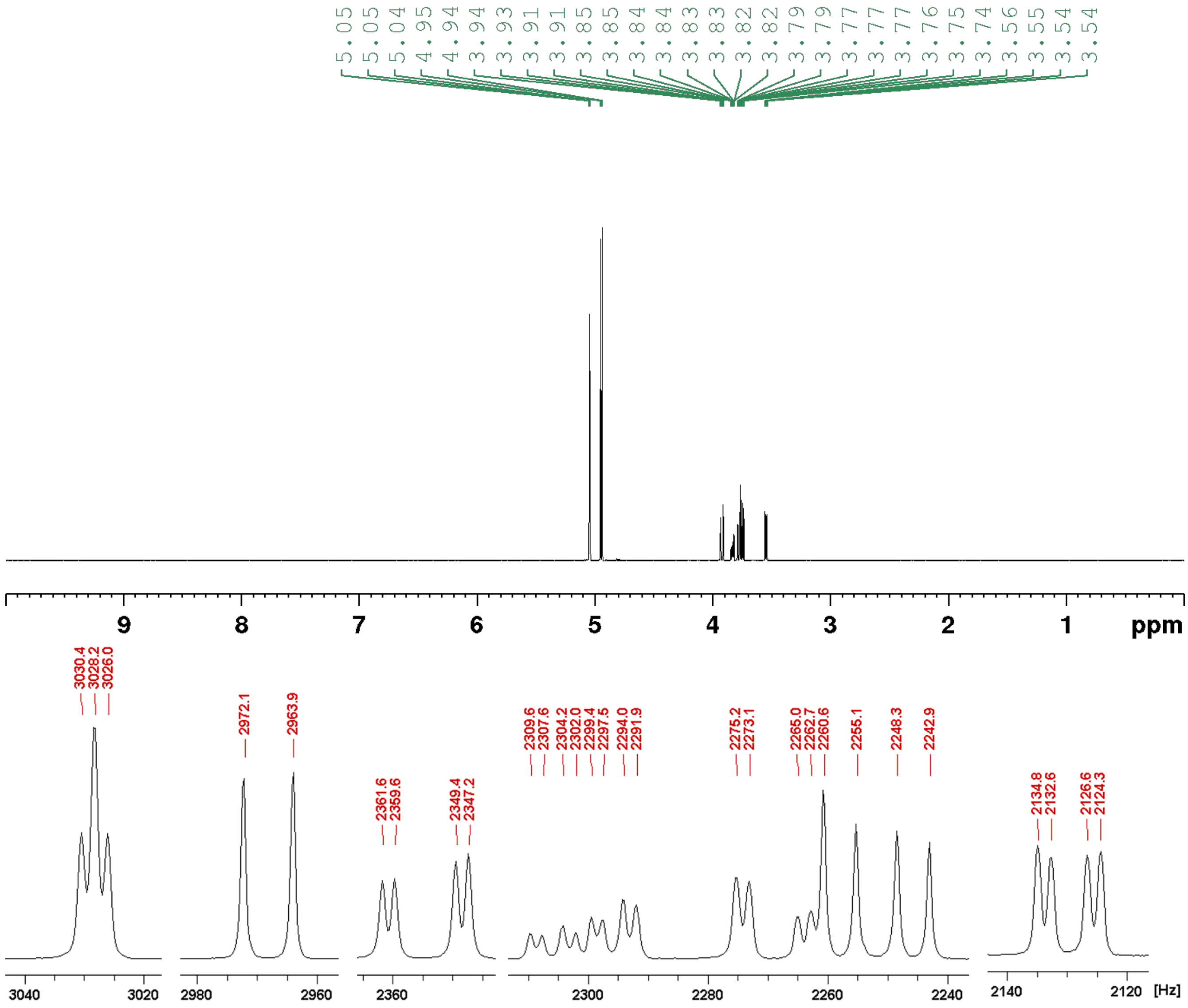

7.10.9 FDAll-3 (**10**): α-furanose form (α-*f*-FDAll-3): **^{1}H{^{19}F} SRI-FESTA NMR** (600 MHz, D$_2$O, δ^{19}F = -195.98 ppm, ZQS = 20 ms, a) δ^{1}H = 5.05 ppm, τ_m = 200 ms; b) δ^{1}H = 4.38 ppm, τ_m = 200 ms)

a)

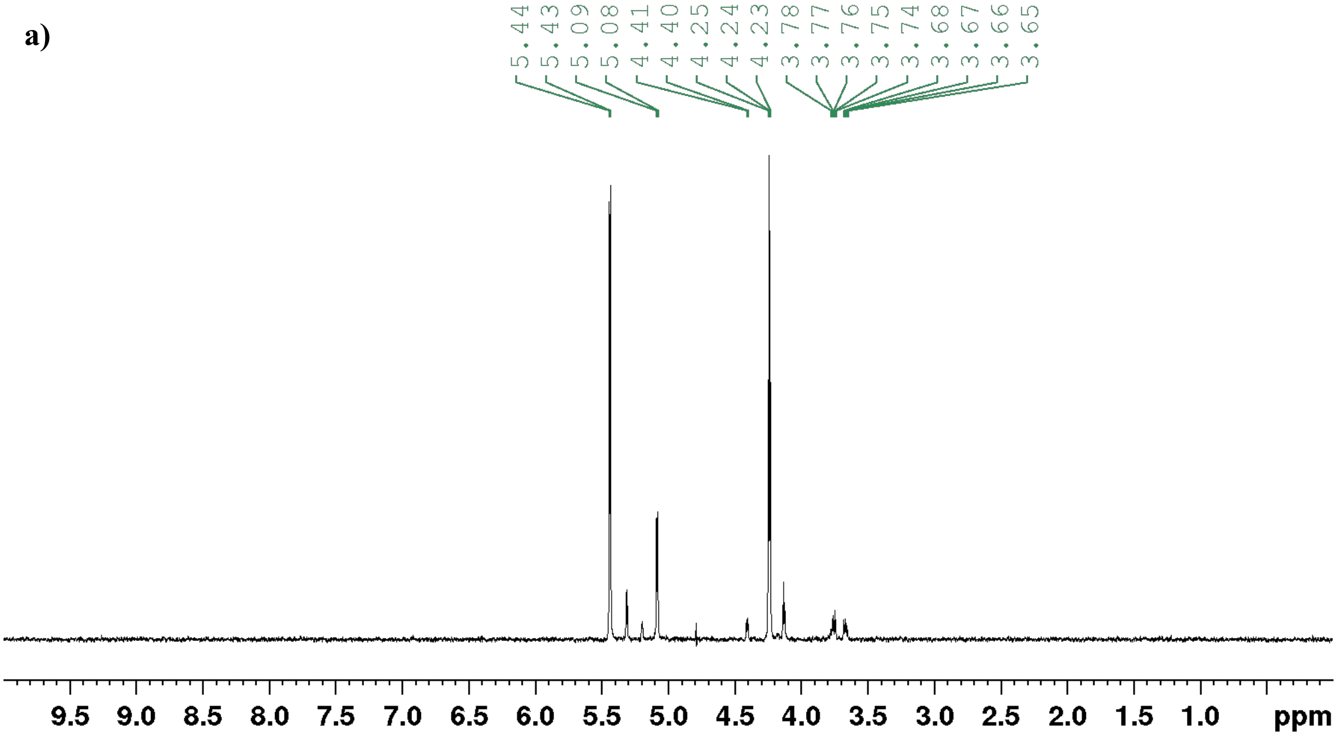

b)

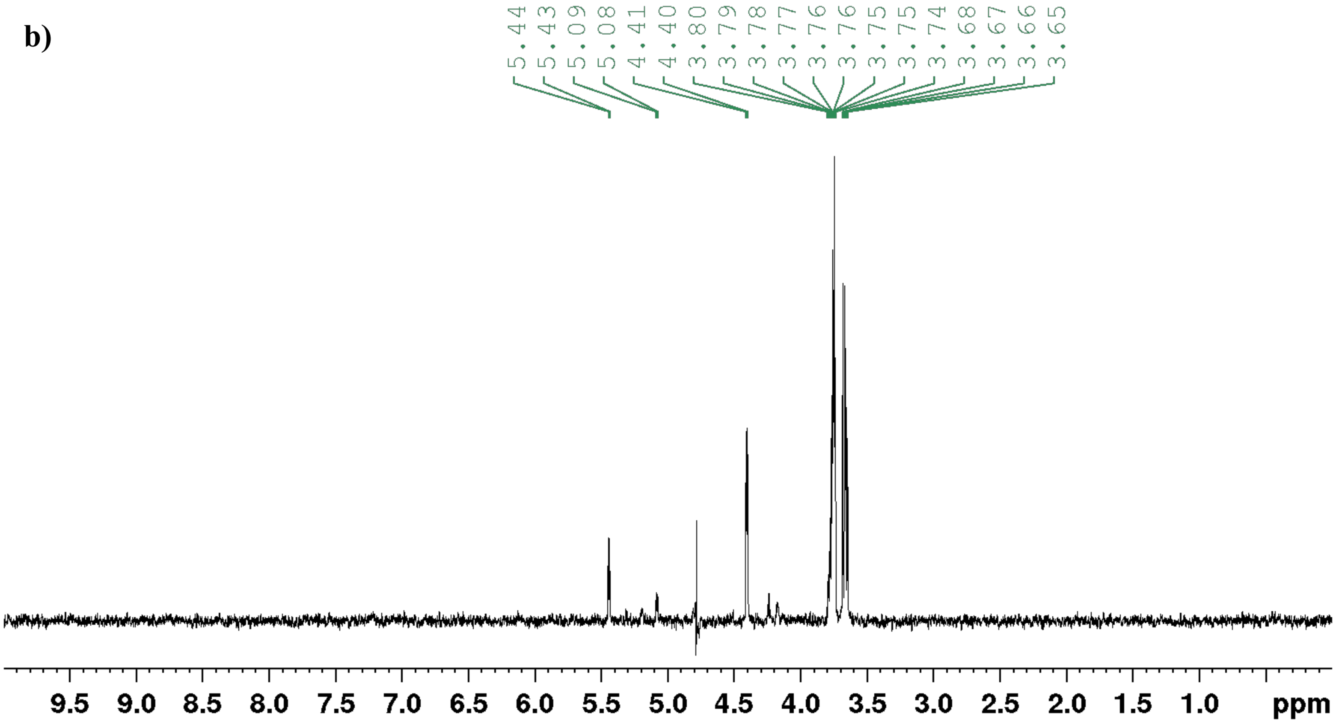

a)

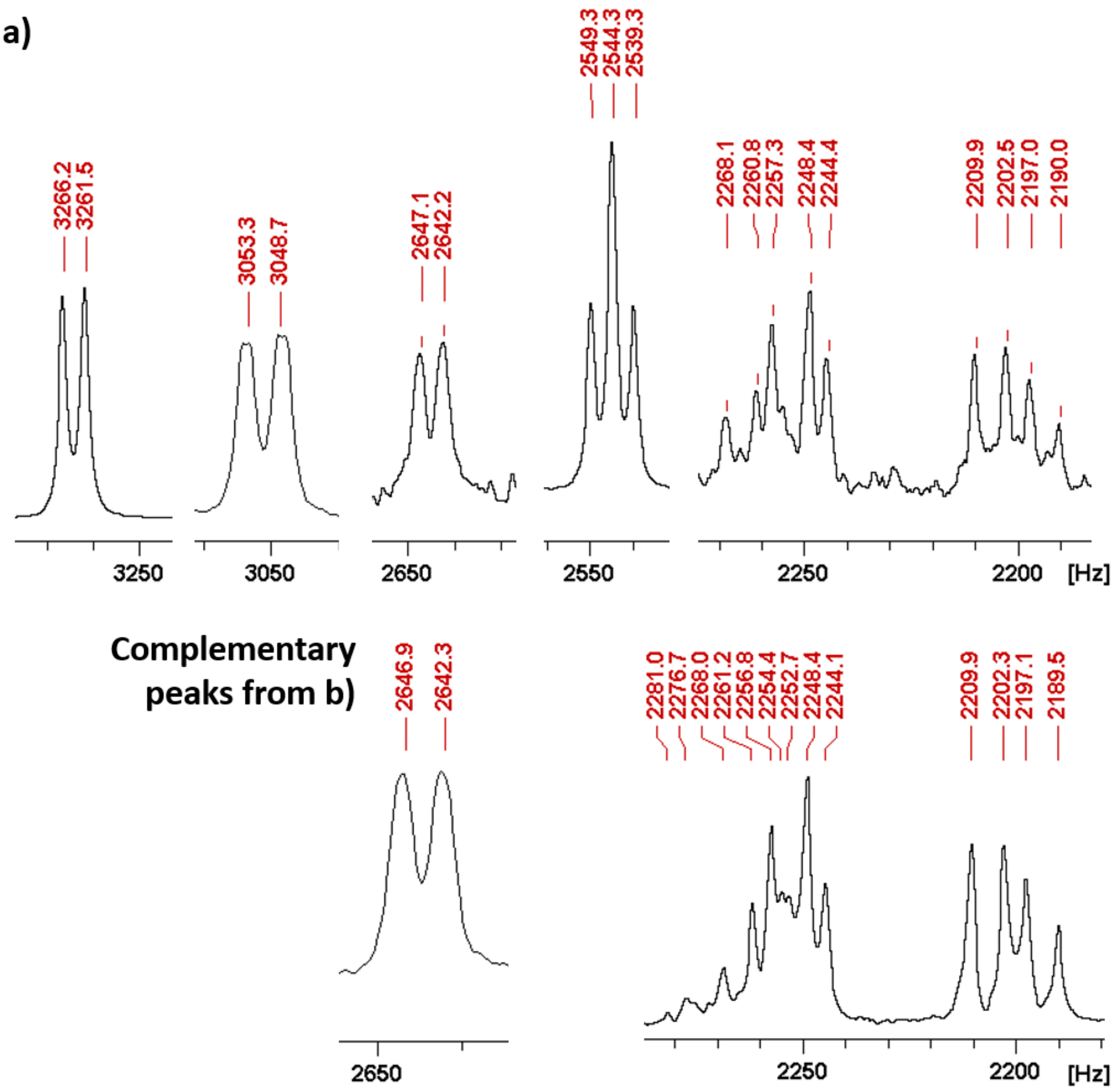
3266.2
3261.5
3053.3
3048.7
2647.1
2642.2
2549.3
2544.3
2539.3
2268.1
2260.8
2257.3
2248.4
2244.4
2209.9
2202.5
2197.0
2190.0
3250
3050
2650
2550
2250
2200
[Hz]
Complementary peaks from b)
2646.9
2642.3
2281.0
2276.7
2268.0
2261.2
2256.8
2254.4
2252.7
2248.4
2244.1
2209.9
2202.3
2197.1
2189.5
2650
2250
2200
[Hz]

7.10.10FDAll-3 (**10**): β-furanose form (β-*f*-FDAll-3): **^{1}H{^{19}F} SRI-FESTA NMR** (600 MHz, D_2O, δ^{19}F = -202.98 ppm, δ^{1}H = 3.89-4.59 ppm, τ_m = 100 ms, ZQS = 20 ms)

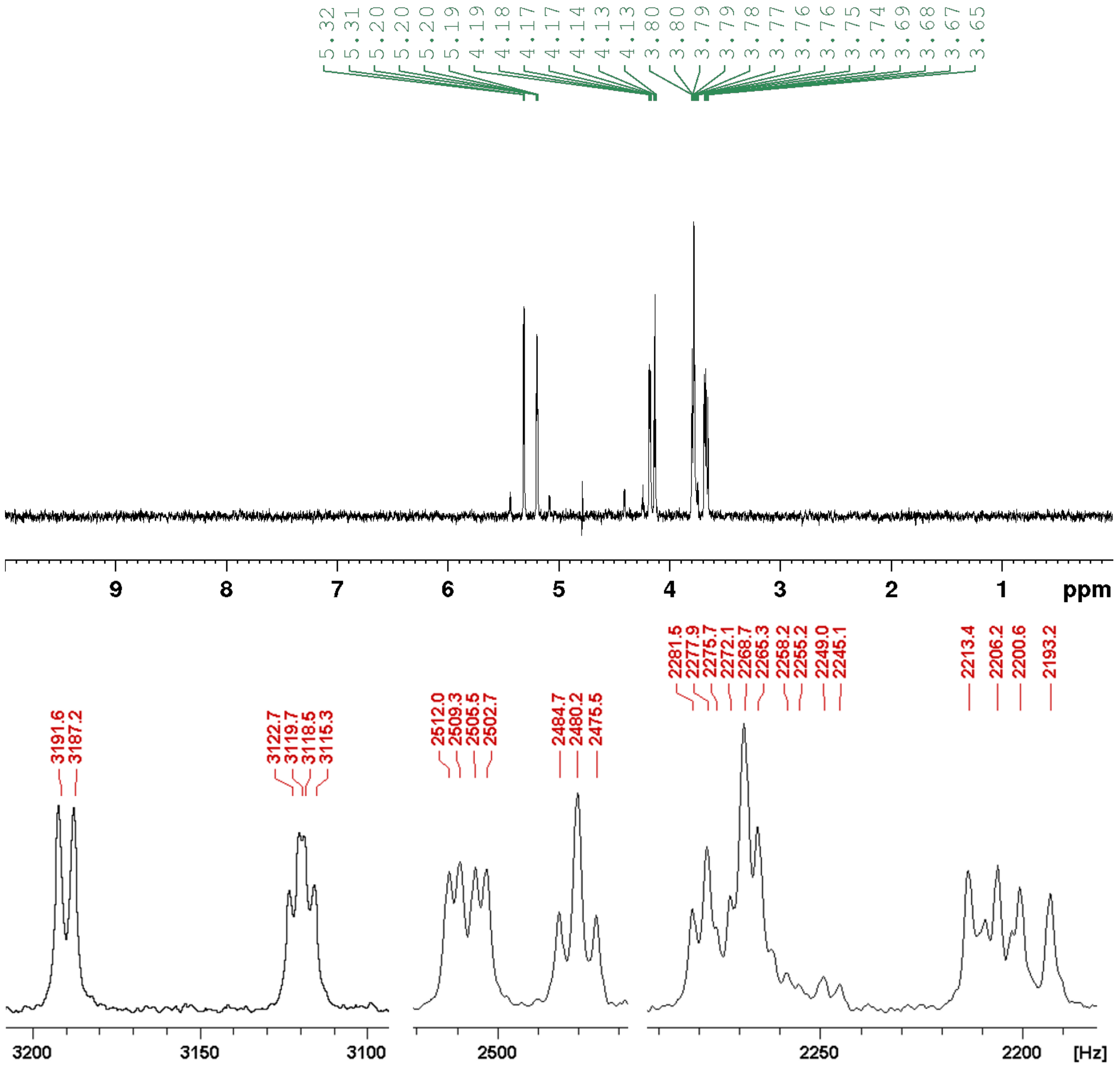

8 References

(1) Castanar, L.; Moutzouri, P.; Barbosa, T. M.; Tormena, C. F.; Rittner, R.; Phillips, A. R.; Coombes, S. R.; Nilsson, M.; Morris, G. A. FESTA: An Efficient Nuclear Magnetic Resonance Approach for the Structural Analysis of Mixtures Containing Fluorinated Species. *Anal. Chem.* **2018**, *90*, 5445-5450.

(2) Bauer, C.; Freeman, R.; Frenkiel, T.; Keeler, J.; Shaka, A. J. Gaussian pulses. *J. Mag. Res.* **1984**, *58*, 442-457.

(3) Kupce, E.; Boyd, J.; Campbell, I. D. Short Selective Pulses for Biochemical Applications. *J. Mag. Res., Series B* **1995**, *106*, 300-303.

(4) Geen, H.; Freeman, R. Band-selective Radiofrequency Pulses. *J. Mag. Res.* **1991**, *93*, 93-141.

(5) Shaka, A. J.; Lee, C. J.; Pines, A. Iterative Schemes for Bilinear Operators; Application to Spin Decoupling. *J. Mag. Res.* **1988**, *77*, 274-293.

(6) Thrippleton, M. J.; Keeler, J. Elimination of Zero-Quantum Interference in Two-Dimensional NMR Spectra. *Angew. Chem. Int. Ed.* **2003**, *42*, 3938-3941.

(7) Barlow, J. N.; Blanchard, J. S. Enzymatic Synthesis of UDP-(3-Deoxy-3-fluoro)-D-galactose and UDP-(2-Deoxy-2-fluoro)-D-galactose and Substrate Activity with UDP-Galactopyranose Mutase. *Carbohydr. Res.* **2000**, *328*, 473-480.

(8) Takahashi, T.; Tanaka, H.; Nakada, T. Method for Producing 18F Labeled Compound and High Molecular Compound to Be Used in the Method. Japan Patent PCT/JP2011/052630.

(9) Li, L.; Liu, Y.; Wan, Y.; Li, Y.; Chen, X.; Zhao, W.; Wang, P. G. Efficient Enzymatic Synthesis of Guanosine 5′-Diphosphate-Sugars and Derivatives. *Org. Lett.* **2013**, *15*, 5528-5530.

(10) Wheatley, D. E.; Fontenelle, C. Q.; Kuppala, R.; Szpera, R.; Briggs, E. L.; Vendeville, J. B.; Wells, N. J.; Light, M. E.; Linclau, B. Synthesis and Structural Characteristics of all Mono- and Difluorinated 4,6-Dideoxy-D-xylo-hexopyranoses. *J. Org. Chem.* **2021**, *86*, 7725-7756.

(11) Yamamoto, H.; Wada, K.; Toyohara, J.; Tago, T.; Ibaraki, M.; Kinoshita, T.; Yamamoto, Y.; Nishiyama, Y.; Kudomi, N. Radiosynthesis of 18F-labeled D-allose. *Carbohydr. Res.* **2019**, *486*, 107827.
