# Supplementary material for: Obtaining Pure 1H NMR Spectra of Individual Pyranose and Furanose Anomers of Reducing Deoxyfluorinated Sugars
Source: J Org Chem. 2023 Sep 27;88(19):13908–25. doi: 10.1021/acs.joc.3c01503 (PMC10563139; doi:10.1021/acs.joc.3c01503)
Supplement: Supplementary file 2 — jo3c01503_si_002.zip [file jo3c01503_si_002.zip › FIDs_Full NMR of all compounds - 1H, 19F, SRI-FESTA/1_FDGlc-2/4/pdata/1/email_bl_2_1.pdf]

2-F-Glu

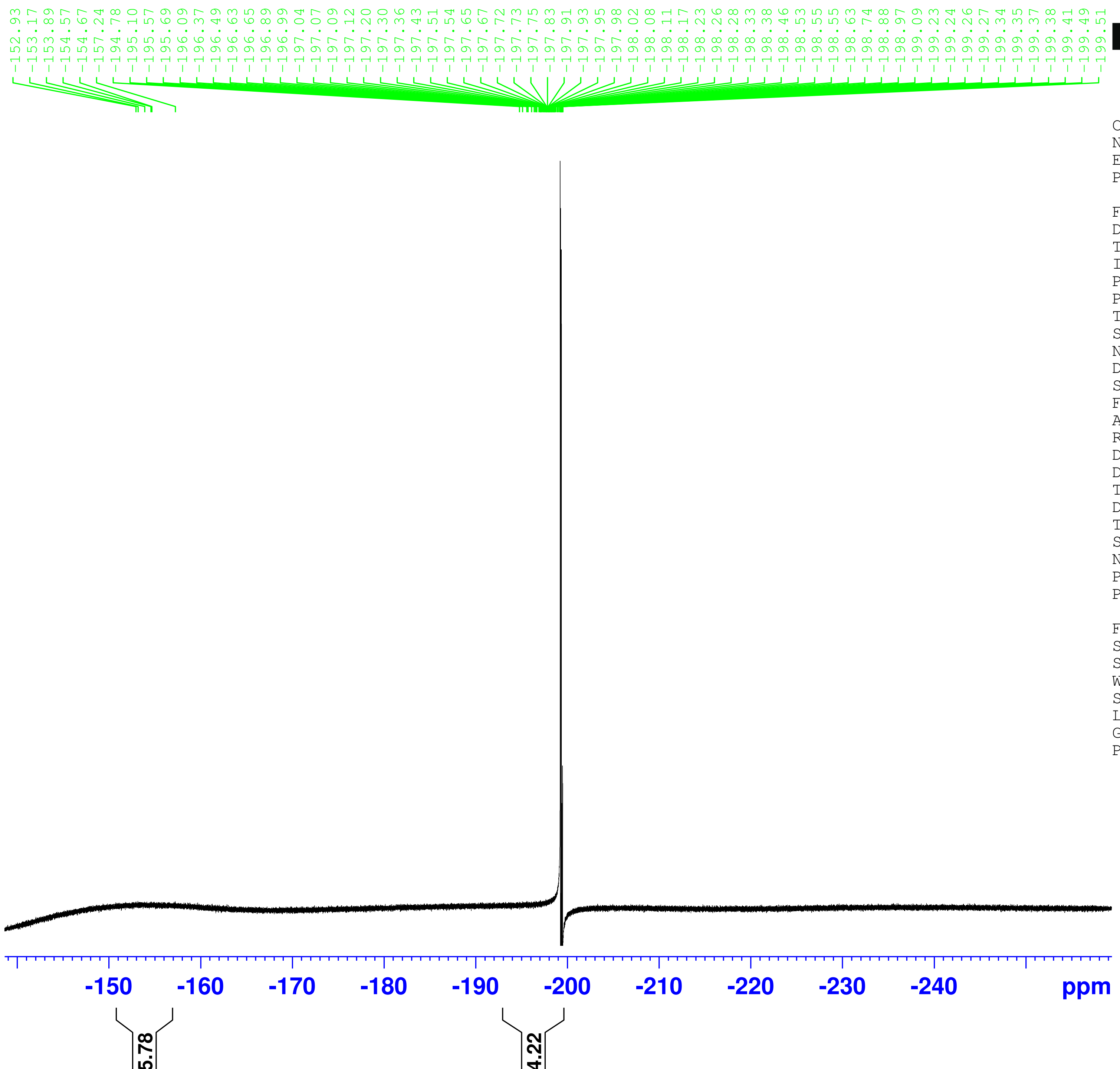

BRUKER

Current Data Parameters

NAME	bl
EXPNO	2
PROCNO	1

F2 - Acquisition Parameters

Date_	20230201
Time	9.58 h
INSTRUM	spect
PROBHD	Z564401_0012 (
PULPROG	zg
TD	131072
SOLVENT	D2O
NS	16
DS	4
SWH	56818.184 Hz
FIDRES	0.866977 Hz
AQ	1.1534336 sec
RG	127.83
DW	8.800 usec
DE	6.50 usec
TE	298.2 K
D1	1.00000000 sec
TD0	1
SFO1	470.4516915 MHz
NUC1	19F
P1	13.00 usec
PLW1	11.26900005 W

F2 - Processing parameters

SI	131072
SF	470.5453300 MHz
WDW	EM
SSB	0
LB	0.30 Hz
GB	0
PC	1.00
